# Supplementary material for: Regio- and stereoselective access to highly substituted vinylphosphine oxides via metal-free electrophilic phosphonoiodination of alkynes
Source: Nat Commun. 2024 Jun 25;15:5385. doi: 10.1038/s41467-024-49640-z (PMC11199708; doi:10.1038/s41467-024-49640-z)
Supplement: Supplementary file 1 — Supplementary Information [file 41467_2024_49640_MOESM1_ESM.pdf]

Supplementary Information

Regio- and Stereoselective Access to Highly Substituted Vinylphosphine Oxides via Metal-Free Electrophilic Phosphonoiodination of Alkynes

Bingbing Dong[a], Fengqian Zhao[a], Wen-Xin Lv[b, c], Ying-Guo Liu[a], Donghui Wei[a]*, Junliang Wu[a]* and Yonggui Robin Chi[b, c]*

[a] *Green Catalysis Center, and College of Chemistry, Zhengzhou University, Zhengzhou 450001, P. R. China*

[b] *National Key Laboratory of Green Pesticide, Key Laboratory of Green Pesticide and Agricultural Bioengineering, Ministry of Education, Guizhou University, Guiyang 550025, P. R. China*

[c] *School of Chemistry, Chemical Engineering, and Biotechnology, Nanyang Technological University, Singapore 637371, Singapore*

Email: robinchi@ntu.edu.sg, wujl@zzu.edu.cn, donghuiwei@zzu.edu.cn

Content

1. General information S2
2. Optimization of reaction conditions S3
3. General procedure for the synthesis of substrates S5
4. General procedure for the phosphonoiodination of unactivated alkynes S8
5. Mechanism experiments S8
6. Analysis of crude reaction mixture by NMR S10
7. DFT calculations S12
8. Synthetic transformations S15
9. Halogen-bond-assisted synthesis of benzo[*b*]phospholes oxides S17
10. Mechanism experiments for the synthesis of benzo[*b*]phospholes oxides S18
11. X-ray crystal structures S20
12. Characteristic data S23
13. NMR spectra S40
14. References S126

1. General information

Unless otherwise noted, materials were purchased from commercial suppliers and used without further purification. All reactions were carried out in sealed tubes filled with argon. All reactions were monitored by Thin Layer Chromatography (TLC) and visualized using UV light. Products were purified by column chromatography by 200-300 mesh silica. NMR spectra (^{1}H, ^{13}C, ^{31}P, ^{19}F) were recorded on 400 or 600 MHz spectrophotometers. Chemical shifts (δ) are reported in ppm from the resonance of the residual solvent peak or tetramethylsilane (TMS) as the internal standard. ^{1}H NMR data are reported as follows: chemical shift (δ ppm), multiplicity (s = singlet, d = doublet, t = triplet, q = quartet, m = multiplet), coupling constants (Hz) and integration. Data for ^{13}C, ^{31}P, and ^{19}F NMR are reported in terms of chemical shifts and multiplicity where appropriate. The high resolution mass spectra (HRMS) were measured on a Thermo-Fisher Scientific Q Exactive MS/MS System ESI spectrometer. X-ray diffraction data was collected on an Oxford Diffraction Xcalibur CCD (X-ray single-crystal diffractometer).

2. Optimization of reaction conditions

Supplementary Table 1. Screening of base[a]

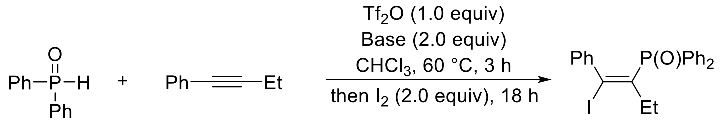

1a, 0.2 mmol **2a**, 0.4 mmol **3a**

Entry	Base	Yield (%)[b]
1	none	50
2[c]	none	trace
3	2,6-Di-*tert*-butylpyridine	69
4	2,4,6-Tri-methylpyridine	32
5	2,6-Di-methylpyridine	trace
6	1,2-Bis(dimethylamino)ethane	48
7	DBN	36
8	Et_3N	trace
9	DBU	trace
10	DABCO	trace
11	TMG	trace
12	Na_2HPO_4	26
13	K_2HPO_4	trace
14	Na_2CO_3	n.d.
15	MeOK	n.d.
16	K_3PO_4	n.d.
17	K_2CO_3	trace

[a]Reaction conditions: **1a** (0.20 mmol, 1.0 equiv), **2a** (0.40 mmol, 2.0 equiv), Tf_2O (0.20 mmol, 1.0 equiv), base (0.40 mmol, 2.0 equiv), $CHCl_3$ (2.0 mL), 60 °C for 3 h, then I_2 (0.40 mmol, 2.0 equiv) was added for 18 h. [b]Isolated yield. "n.d." stands for "not detected". [c]I_2 (0.40 mmol, 2.0 equiv) was added at first. DBN: 1,5-Diazabicyclo[4.3.0]-5-nonene; DBU = 1,8-Diazabicyclo[5.4.0]undec-7-ene; DABCO = 1,8-Diazabicyclo[5.4.0]undec-7-ene; TMG = 1,1,3,3-Tetramethylguanidine.

Supplementary Table 2. Screening of activating species[a]

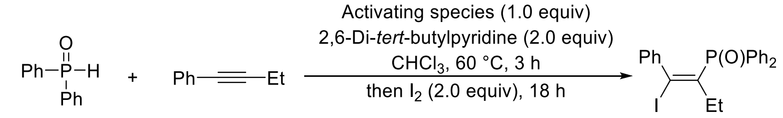

1a, 0.2 mmol **2a**, 0.4 mmol **3a**

Entry	Activating species	Yield (%)[b]
1	Tf_2O	69
2	TMSOTf	n.d.
3	$BF_3 \cdot Et_2O$	n.d.
4	$(CF_3CO)_2O$	n.d.

[a]Reaction conditions: **1a** (0.20 mmol, 1.0 equiv), **2a** (0.40 mmol, 2.0 equiv), activating species (0.20 mmol, 1.0 equiv), 2,6-di-*tert*-butylpyridine (0.40 mmol, 2.0 equiv), $CHCl_3$ (2.0 mL), 60 °C for 3 h, then I_2 (0.40 mmol, 2.0 equiv) was added for 18 h. [b]Isolated yield. "n.d." stands for "not detected".

Supplementary Table 3. Screening of I source[a]

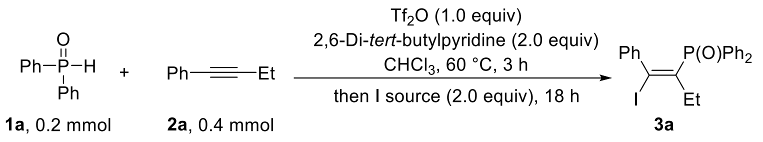

Entry	I source	Yield (%)[b]
1	I_2	69
2	NIS	33
3	ICl	27
4[c]	NIS	trace
5[c]	ICl	n.d.
6	TBAI	n.d.
7	NaI	n.d.

[a]Reaction conditions: **1a** (0.20 mmol, 1.0 equiv), **2a** (0.40 mmol, 2.0 equiv), Tf_2O (0.20 mmol, 1.0 equiv), 2,6-di-*tert*-butylpyridine (0.40 mmol, 2.0 equiv), $CHCl_3$ (2.0 mL), 60 °C for 3 h, then I source (0.40 mmol, 2.0 equiv) was added for 18 h. [b]Isolated yield. "n.d." stands for "not detected". [c]I source was added at first and stirred at 60 °C for 21 h.

Supplementary Table 4. Screening the loading of Tf_2O[a]

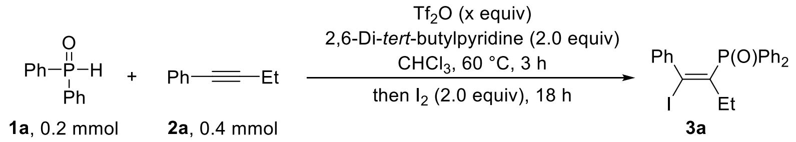

Entry	x equiv	Yield (%)[b]
1	1.0	69
2	1.1	73
3	1.2	47
4	1.5	19
5[c]	1.0	75
6[c]	1.05	83

[a]Reaction conditions: **1a** (0.20 mmol, 1.0 equiv), **2a** (0.40 mmol, 2.0 equiv), Tf_2O (x equiv), 2,6-di-*tert*-butylpyridine (0.40 mmol, 2.0 equiv), $CHCl_3$ (2.0 mL), 60 °C for 3 h, then I_2 (0.40 mmol, 2.0 equiv) was added for 18 h. [b]Isolated yield. [c]$CHCl_3$: 1.0 mL.

3. General procedure for the synthesis of substrates

3.1 General procedure for the synthesis of secondary phosphine oxides

Diaryl phosphine oxides (**1c-1k, 1m-1n**) were synthesized according to the following procedure (**GP1**).[1] Phenyl - alkyl secondary phosphine oxides (**1l**) were synthesized according to the following procedure (**GP2**).[2]

Supplementary Table 5. **Substrates of secondary phosphine oxides**

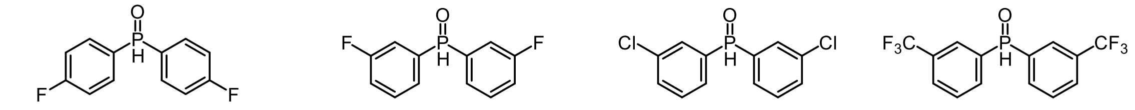

1c **1d** **1e** **1f**

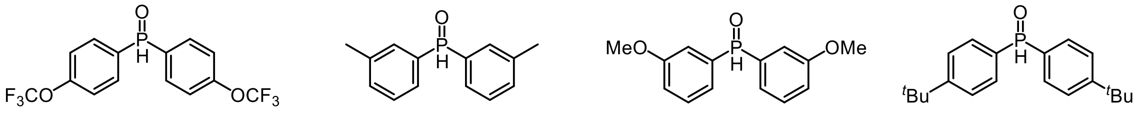

1g **1h** **1i** **1j**

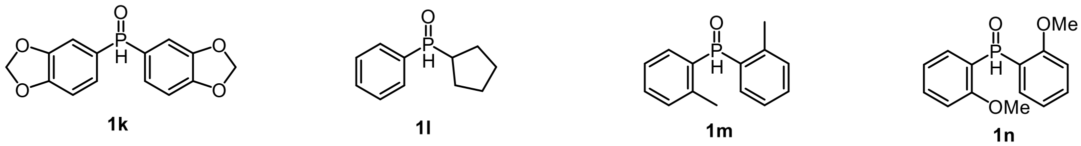

1k **1l** **1m** **1n**

General Procedure 1 (GP1)

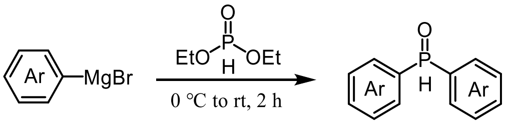

1c-1k, **1m** and **1n**

A 100 mL round-bottomed flask equipped with a magnetic stirrer under argon atmosphere was charged with Grignard reagent (33 mL, 1 M in THF). A mixture of diethyl phosphonate (1.38 g, 10 mmol) and anhydrous THF (5 mL) was added slowly to the Grignard reagent at 0 °C. The mixture was allowed to warm to rt about 2 h and then was quenched with sat. aq. NH_4Cl solution, and extracted with EtOAc. The combined organic layers were dried over $MgSO_4$, filtered, and concentrated in vacuo. The product was obtained after purification by column chromatography on silica gel to provide desired products (60% - 80% yield).

General Procedure 2 (GP2)

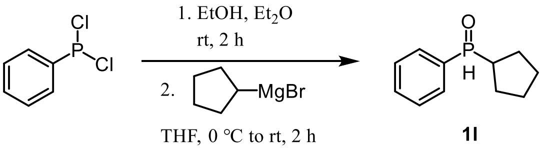

1l

A 50 mL round-bottomed flask equipped with a magnetic stirrer under argon atmosphere was charged with dichlorophenylphosphine (1.35 mL, 10.0 mmol) and Et_2O (25 mL). EtOH (1.45 mL, 25.0 mmol) was added dropwise over 5 mins and the resulting solution was stirred at rt for 2 h. The solvent was removed under vacuum and the resulting crude ethyl phosphinate was dissolved in THF (12 mL) under a nitrogen atmosphere. The cyclopentylmagnesium bromide (2.2 equiv) in THF was cooled to 0 °C under an inert atmosphere and the ethyl phosphinate solution was added dropwise at 0 °C over 30 mins. The reaction was stirred at rt for 2 h, then quenched with sat. aq. NH_4Cl solution.

Water was then added and the aqueous phase was then extracted with EtOAc. The combined organic layers were dried over $MgSO_4$, filtered, and concentrated in vacuo. The product was obtained after purification by column chromatography on silica gel to provide the corresponding product **1l** (65% yield).

3.2 General procedure for the synthesis of alkynes

The alkynes (**2c-2s and 2u**) were synthesized according to the following procedure (**GP3**).[3] The alkyne (**2t**) was synthesized according to the following procedure (**GP4**).[4, 5] The alkyne (**2v**) was synthesized according to the published procedure.[6] The alkyne (**2w**) was synthesized according to the published procedure.[7]

***Supplementary Table 6*. Substrates of alkynes**

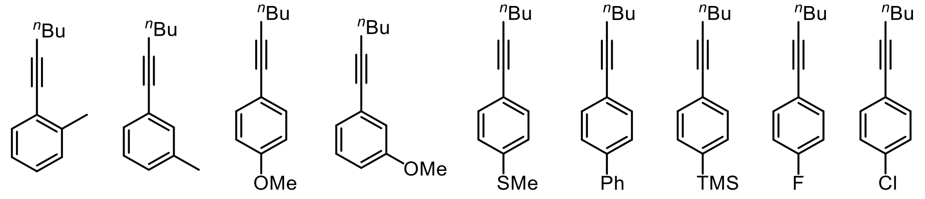

2c **2d** **2e** **2f** **2g** **2h** **2i** **2j** **2k**

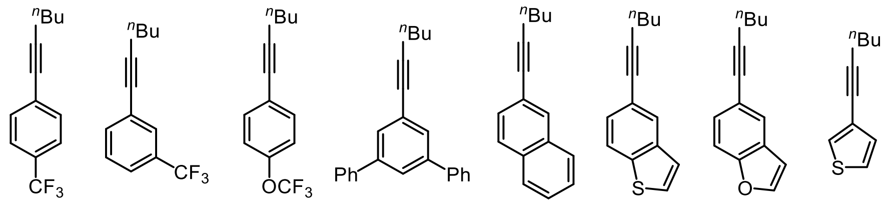

2l **2m** **2n** **2o** **2p** **2q** **2r** **2s**

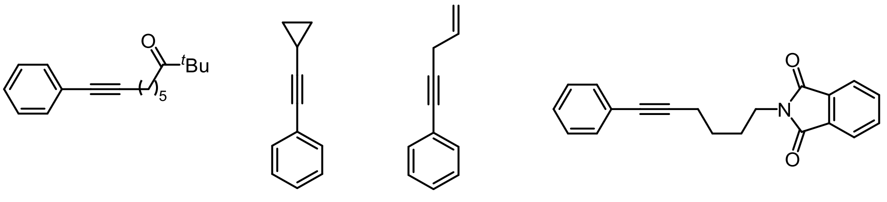

2t **2u** **2v** **2w**

General Procedure 3 (GP3)

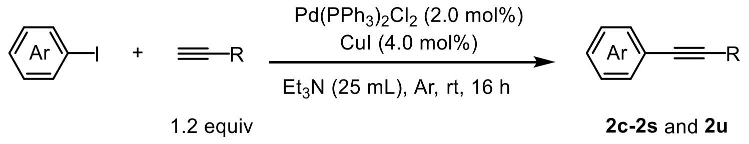

A 100 mL Schlenk flask with a stirring bar were charged with $Pd(PPh_3)_2Cl_2$ (0.2 mmol, 0.02 equiv), CuI (0.4 mmol, 0.04 equiv), aryl iodide (10 mmol, 1.0 equiv) and Et_3N (25 mL) under argon. Terminal alkyne (12 mmol, 1.2 equiv) was added slowly to the reaction mixture. The solution was stirred for 16 h at room temperature. After the reaction, the solvent was removed under reduced pressure and the crude product was purified by column chromatography (80% - 95% overall yield).

General Procedure 4 (GP4)

step 1

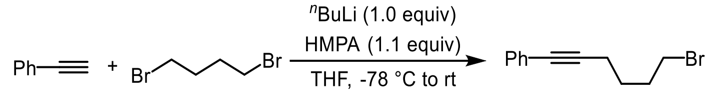

step 2

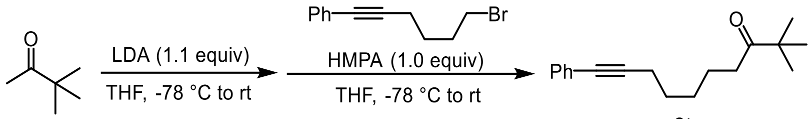

2t

Step 1: To a solution of phenyl acethylene (10 mmol, 1.0 equiv) in THF (15 mL) at -78 °C was added ^{n}BuLi (10 mmol, 1.0 equiv), and the mixture was allowed to warm to rt over 0.5 h. The mixture was then cooled to -78 °C, HMPA (11 mmol, 1.1 equiv) and dibromobutane (10 mmol, 1.0 equiv) were added. The mixture was allowed to warm to rt about 2 h and then was quenched with water, and extracted with Et_2O. The organic layer was washed brine, dried over $MgSO_4$, and concentrated. The residue was purified by column chromatography to provide (6-bromohex-1-yn-1-yl)benzene.

Step 2: To a stirred solution of pinacolone (10 mmol, 1.0 equiv) in THF (20 mL) was slowly added LDA (11 mmol, 1.1 equiv) at -78 °C. The resulting mixture was stirred at -78 °C for 30 min, and then allowed to room temperature over 30 min. The mixture was cooled again to -78 °C, followed by the addition of HMPA (10 mmol, 1.0 equiv) and (6-bromohex-1-yn-1-yl)benzene (10 mmol, 1.0 equiv). The reaction mixture was allowed to room temperature, stirred overnight, and poured into brine (50 mL). The layers were separated, and the aqueous phase was extracted with Et_2O. The combined organic layer was dried over $MgSO_4$ and concentrated under reduced pressure. The residue was purified by column chromatography to provide the corresponding product **2t** (53% yield).

4. General procedure for the phosphonoiodination of unactivated alkynes

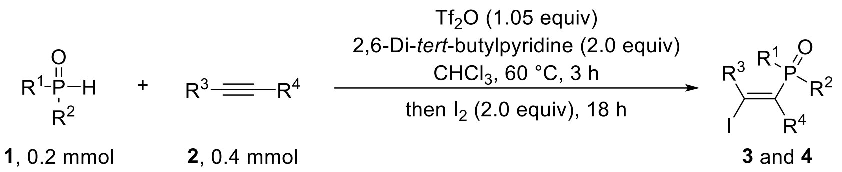

General Procedure 5 (GP5)

A 10 mL oven-dried sealed tube equipped with a magnetic stir bar was charged with secondary phosphine oxides (0.2 mmol, 1.0 equiv), alkynes (0.4 mmol, 2.0 equiv, if solid). The tube was evacuated and backfilled with argon (three times) and then $CHCl_3$ (1.0 mL) was added sequentially via a syringe, followed by alkynes (0.4 mmol, 2.0 equiv, if oil), 2,6-di-*tert*-butylpyridine (0.4 mmol, 2.0 equiv) and Tf_2O (0.21 mmol, 1.05 equiv) were added by a syringe. The resulting mixture was stirred for 3 h at 60 °C, and I_2 (0.4 mmol, 2.0 equiv) was added for 18 h at 60 °C. After cooled to ambient temperature, sat. $NaHCO_3$ aq (5.0 mL) was added and the resulting mixture was extracted with DCM (3 x 10 mL). The organic layer was washed with sat. $Na_2S_2O_3$ aq and brine, followed by dried over $MgSO_4$, and volatiles were removed under reduced pressure. The residue was purified by flash column chromatography on silica gel to give the desired products.

5. Mechanism experiments

5.1 Control experiment using ^{18}O-labeled diphenylphosphine oxide

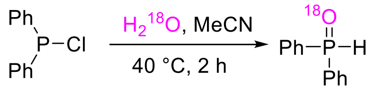

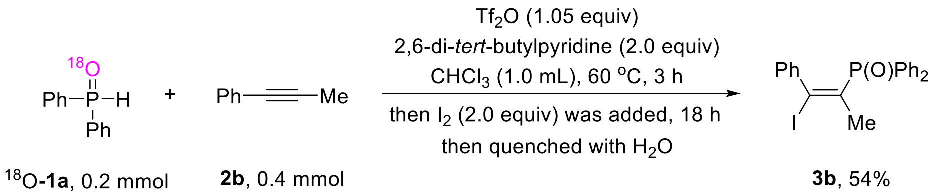

Step 1: A 10 mL oven-dried sealed tube equipped with a magnetic stir bar was charged with chlorodiphenylphosphine (36 μL, 0.2 mmol). The tube was evacuated and backfilled with argon (three times) and then dry acetonitrile (0.5 mL) was added via a syringe. Cooled to 0 °C, 0.1 mL of $H_2{}^{18}O$ was added slowly and the reaction mixture was allowed to stir at 40 °C for 2 h. After cooling down to room temperature, solvent was removed under reduced pressure and the resulting crude diphenylphosphine oxide (^{18}O) was used in next step without further purification.

Step 2: A 10 mL oven-dried sealed tube equipped with a magnetic stir bar was charged with ^{18}O-labeled diphenylphosphine oxide (0.2 mmol, 1.0 equiv) prepared in the previous step. The tube was evacuated and backfilled with argon (three times) and then $CHCl_3$ (1.0 mL) was added via a syringe, followed by **2b** (0.4 mmol, 2.0 equiv), 2,6-di-*tert*-butylpyridine (0.4 mmol, 2.0 equiv) and Tf_2O (0.21 mmol, 1.05 equiv) was added by a syringe. The resulting mixture was stirred for 3 h at 60 °C, and I_2 (0.4 mmol, 2.0 equiv) was added for 18 h at 60 °C. After cooled to ambient temperature, sat. $NaHCO_3$ aq (5.0 mL) was added and the resulting mixture was extracted with DCM (3 x 10 mL).

The organic layer was washed with sat. $Na_2S_2O_3$ aq and brine, followed by dried over $MgSO_4$, and volatiles were removed under reduced pressure. The residue was purified by flash column chromatography on silica gel to give the desired product ^{16}O-**3b** (54% yield), which indicated that the oxygen of **3b** did not come from diphenylphosphine oxide.

5.2 Control experiment using $H_2{}^{18}O$

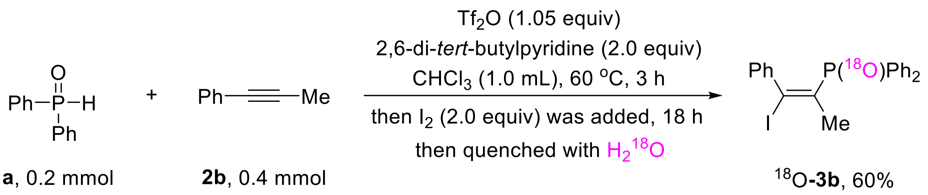

A 10 mL oven-dried sealed tube equipped with a magnetic stir bar was charged with diphenylphosphine oxide **1a** (0.2 mmol, 1.0 equiv). The tube was evacuated and backfilled with argon (three times) and then $CHCl_3$ (1.0 mL) was added via a syringe, followed by **2b** (0.4 mmol, 2.0 equiv), 2,6-di-*tert*-butylpyridine (0.4 mmol, 2.0 equiv) and Tf_2O (0.21 mmol, 1.05 equiv) were added by a syringe. The resulting mixture was stirred for 3 h at 60 °C, and I_2 (0.4 mmol, 2.0 equiv) was added for 18 h at 60 °C. After cooled to ambient temperature, $H_2{}^{18}O$ (40 µL) was added to the mixture and stirred for additional 2 h at 60 °C. Then sat. $NaHCO_3$ aq (5.0 mL) was added and the resulting mixture was extracted with DCM (3 x 10 mL). The organic layer was washed with sat. $Na_2S_2O_3$ aq and brine, followed by dried over $MgSO_4$, and volatiles were removed under reduced pressure. The residue was purified by flash column chromatography on silica gel to give corresponding product ^{18}O-**3b** (HRMS calcd for $C_{21}H_{19}I^{18}OP^+$ $[M+H]^+$ 447.0255, Found 447.0279, 60% yield), which indicated that the oxygen did come from outside water.

5.3 Control experiment using 4-methyl-*N*-phenyl-*N*-(3-phenylprop-2-yn-1-yl) benzenesulfonamide

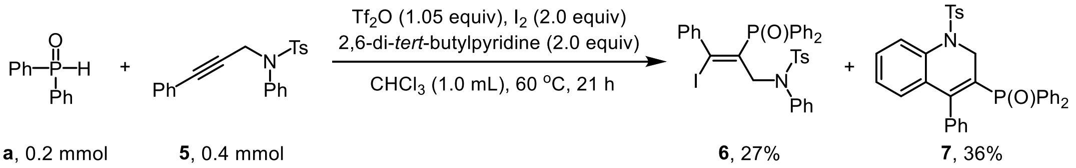

A 10 mL oven-dried sealed tube equipped with a magnetic stir bar was charged with secondary phosphine oxides **1a** (0.2 mmol, 1.0 equiv) and alkyne **5** (0.4 mmol, 2.0 equiv). The tube was evacuated and backfilled with argon (three times) and then $CHCl_3$ (1.0 mL) was added sequentially via a syringe, followed by 2,6-di-*tert*-butylpyridine (0.4 mmol, 2.0 equiv), Tf_2O (0.21 mmol, 1.05 equiv) and I_2 (0.4 mmol, 2.0 equiv) were added. The resulting mixture was stirred for 21 h at 60 °C. After cooled to ambient temperature, sat. $NaHCO_3$ aq (5.0 mL) was added and the resulting mixture was extracted with DCM (3 x 10 mL). The organic layer was washed with sat. $Na_2S_2O_3$ aq and brine, followed by dried over $MgSO_4$, and volatiles were removed under reduced pressure. The residue was purified by flash column chromatography on silica gel to give the phosphonoiodination product **6** in 27% yield and cyclization product **7** in 36% yield, which indicated that phosphonoiodination of unactivated alkynes may undergo a three-membered phosphirenium cation intermediate. Spectroscopic data of **7** in agreement with that reported previously.[8]

5.4 *In situ* ^{31}P NMR experiment

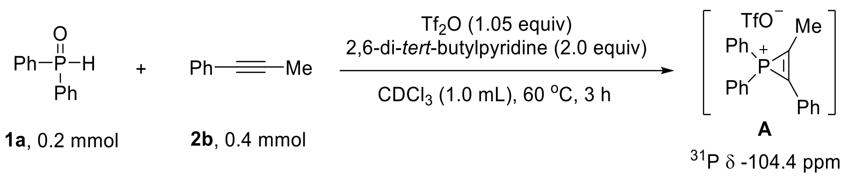

A 10 mL oven-dried sealed tube equipped with a magnetic stir bar was charged with secondary phosphine oxides **1a** (0.2 mmol, 1.0 equiv) and alkyne **2b** (0.4 mmol, 2.0 equiv). The tube was evacuated and backfilled with argon (three times) and then $CDCl_3$ (1.0 mL) was added sequentially via a syringe, followed by 2,6-di-*tert*-butylpyridine (0.4 mmol, 2.0 equiv), and Tf_2O (0.21 mmol, 1.05 equiv) were added. The resulting mixture was stirred for 3 h at 60 °C. After cooled to ambient temperature, the reaction solution was transferred to an NMR tube under argon and detected by ^{31}P NMR, and we observed a peak of -104.4 ppm (**Supplementary Figure 1**) which is assigned to the characteristic shift of phosphirenium ions. [7]

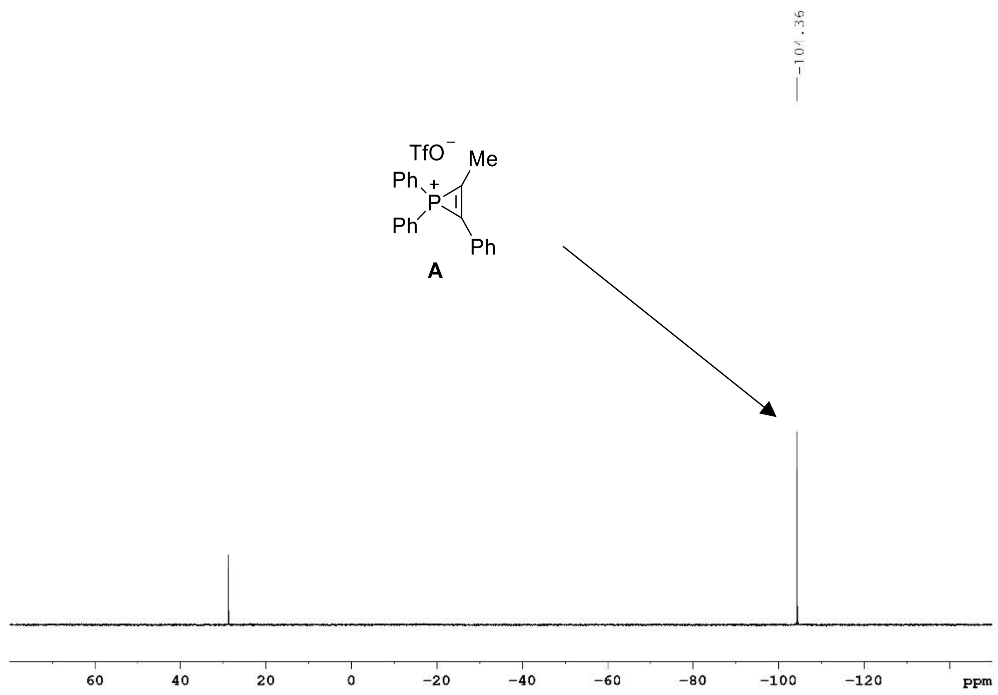

Supplementary Figure 1. *In-situ* ^{31}P NMR

6. Analysis of crude reaction mixture by NMR

6.1 Analysis of crude reaction mixture by ^1H NMR

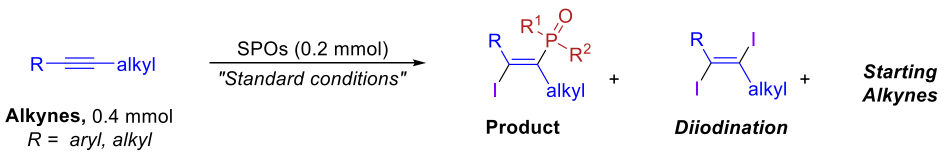

A 10 mL oven-dried sealed tube equipped with a magnetic stir bar was charged with SPOs (0.2

mmol, 1.0 equiv). The tube was evacuated and backfilled with argon (three times) and then $CHCl_3$ (1.0 mL) was added sequentially via a syringe, followed by Tf_2O (0.21 mmol, 1.05 equiv), 2,6-di-*tert*-butylpyridine (0.4 mmol, 2.0 equiv) and alkynes (0.4 mmol, 2.0 equiv) were added by a syringe. The resulting mixture was stirred for 3 h at 60 °C, and then I_2 (0.4 mmol, 2.0 equiv) was added and stirred for an additional 18 h at 60 °C. After cooling to ambient temperature, sat. $NaHCO_3$ aq (5.0 mL) was added and the resulting mixture was extracted with DCM (3 x 10 mL). The organic layer was washed with sat. $Na_2S_2O_3$ aq and brine, dried over $MgSO_4$, and then the volatiles were removed under reduced pressure. 1,3,5-Trimethoxybenzene (0.2 mmol) was added to the residue as an internal standard, dissolved with $CDCl_3$, and then the solution was transferred to an NMR tube and analyzed by ^{1}H NMR.

***Supplementary Table 7*. Alkynes' conversions and residuals**

Entry	Product	*Diiodination*	*Starting Alkynes*	Total alkynes *converted and remaining*
1	**3a**, 0.164 mmol	0.13 mmol	0.1 mmol	0.394 mmol, 99%
2	**3h**, 0.1 mmol	0.25 mmol	0.04 mmol	0.39 mmol, 98%
3	**4j**, 0.024 mmol	0.24 mmol	0.13 mmol	0.394 mmol, 99%
4	**4k**, 0.028 mmol	0.26 mmol	0.11 mmol	0.398 mmol, > 99%
5	**4l**, 0.09 mmol	0.2 mmol	0.11 mmol	0.4 mmol, > 99%
6	**4v**, 0.042 mmol	0.35 mmol	/	0.392 mmol, 98%

In the analysis the crude ^{1}H NMR spectra, we observed the formation of diiodination product and the remaining of starting alkynes and we analyzed the transformations and residuals of alkynes with examples of ***3a*, *3h*, *4j*, *4k*, *4l*** *and* ***4v***. *As seen in* ***Supplementary Table*** *7, the sum of the conversions and residues of the alkynes after the reaction essentially matches the initially added amount of alkynes.*

6.2 Analysis of crude reaction mixture by ^{31}P NMR

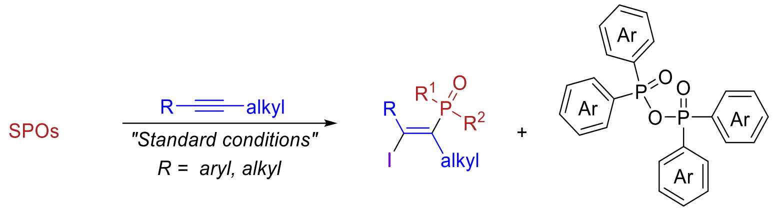

A 10 mL oven-dried sealed tube equipped with a magnetic stir bar was charged with SPOs (0.2 mmol, 1.0 equiv). The tube was evacuated and backfilled with argon (three times) and then $CHCl_3$ (1.0 mL) was added sequentially via a syringe, followed by Tf_2O (0.21 mmol, 1.05 equiv), 2,6-di-*tert*-butylpyridine (0.4 mmol, 2.0 equiv) and alkynes (0.4 mmol, 2.0 equiv) were added by a syringe. The resulting mixture was stirred for 3 h at 60 °C, and then I_2 (0.4 mmol, 2.0 equiv) was added and stirred for an additional 18 h at 60 °C. After cooling to ambient temperature, sat. $NaHCO_3$ aq (5.0 mL) was added and the resulting mixture was extracted with DCM (3 x 10 mL). The organic layer was washed with sat. $Na_2S_2O_3$ aq and brine, dried over $MgSO_4$, and then the volatiles were removed under reduced pressure. Ph_3PO was added to the residue as a reference, dissolved with $CDCl_3$, and then the solution was transferred to an NMR tube and analyzed by ^{31}P NMR.

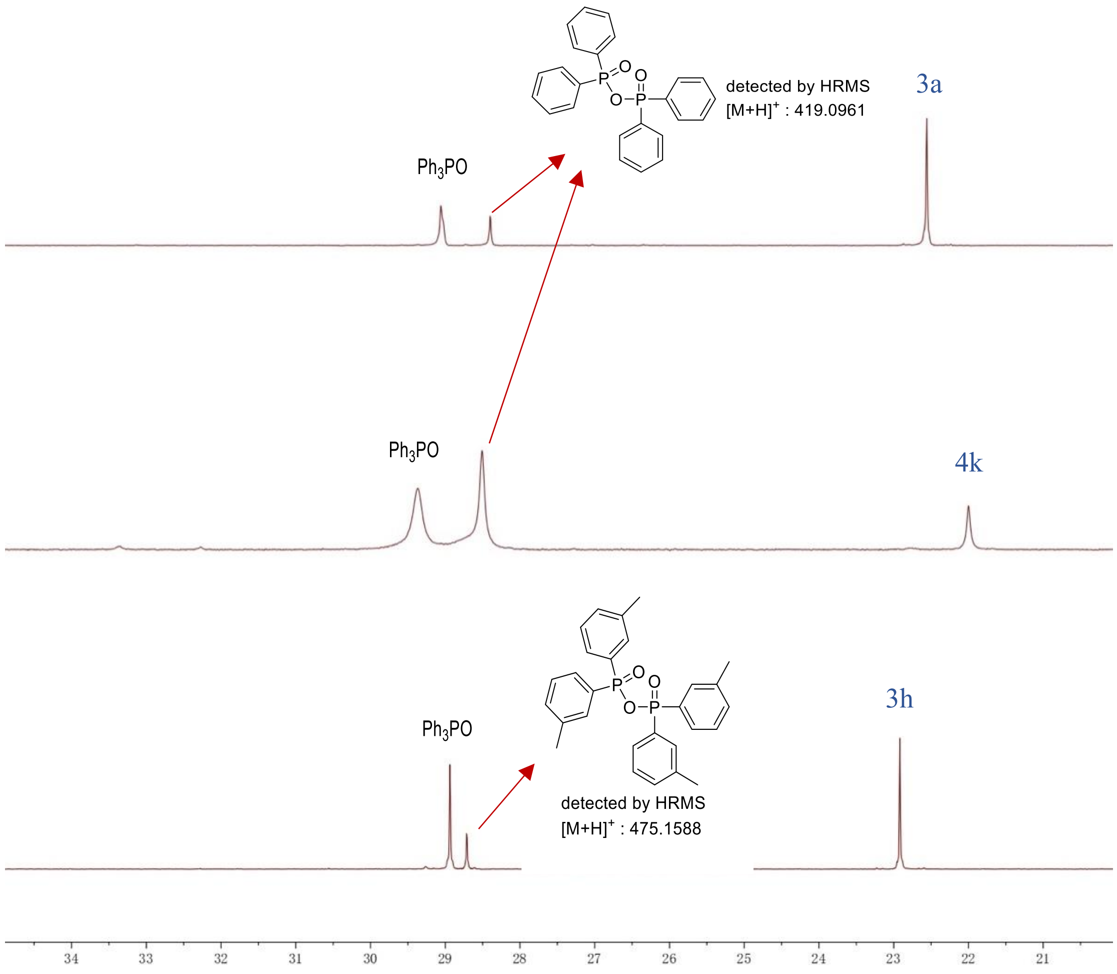

Supplementary Figure 2: Crude ^{31}P NMR spectra of reaction

*By analyzing the ^{31}P NMR spectra of the crude reaction mixture (**Supplementary Figure 2**), we observed the phosphoric anhydrides[9] (~28.5 ppm) and products with the examples of **3a**, **3h** and **4k**, and the phosphoric anhydrides were also detected by HRMS. **No starting SPOs were detected.** Additionally, the hydrophosphorylated product (HRMS (ESI) calcd for $C_{24}H_{26}OP^+$ $[M+H]^+$ 361.1716, found 361.1716) and di-m-tolylphosphinic acid (HRMS (ESI) calcd for $C_{14}H_{16}O_2P^+$ $[M+H]^+$ 247.0882, found 247.0883) were also detected by HRMS in the crude mixture of **3h**. However, their amounts are so small that it is difficult to observe them in the ^{31}P NMR spectrum.*

7. DFT calculations

Computational Methods

Gaussian09 program[10] was employed for the theoretical calculations. The geometry optimization was carried out by using the M06-2X method[11, 12]. The 6-31G (d, p) basis set[13, 14] was used for C, H, O, and P atoms, whereas the LANL2DZ pseudopotential basis set[15, 16] was used for the I atom. Solvent effects were considered using the integral equation formalism polarizable continuum model (IEF-PCM)[17] with chloroform as the solvent.

7.1 DFT calculations based on phosphirenium iodide

DFT calculations were employed to elucidate the mechanism and chemoselectivity of the ring-opening process. As shown in **Supplementary Figure 3**, the cleavage of C-P and I-I bonds is coupled with formation of C-I and P-I bonds during the ring-opening process, and the energy barriers of the four possible chemoselective pathways associated with the concerted transition states **A-TS1**, **B-TS1**, **C-TS1**, and **D-TS1** are 16.1 kcal/mol, 21.6 kcal/mol, 17.5 kcal/mol, and 18.2 kcal/mol, respectively. Meanwhile, intermediate **A-M1** has the lowest energy among the four intermediates (vs. **B-M1**, **C-M1**, and **D-M1**). Therefore, the chemoselective pathway associated with **A-TS1** and **A-M1** is the most energetically favourable pathway in both kinetics and thermodynamics. The calculated results are in agreement with the experimental observations.

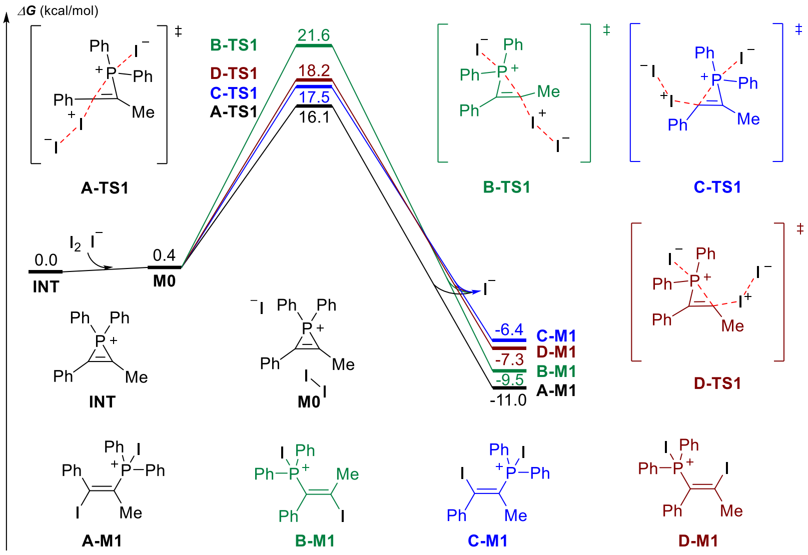

Supplementary Figure 3. The relative Gibbs energy profiles of possible chemoselective ring-opening pathways without the presence of TfO$^-$.

7.2 DFT calculations based on phosphirenium triflate

Moreover, we have investigated the four possible chemoselective ring-opening pathways via transition states A/B/C/D-TS2 with the presence of TfO$^-$. The corresponding energy barriers of these pathways, depicted in **Supplementary Figure 4**, are significantly higher than those of the pathways shown in **Supplementary Figure 3**, so we think that the presence of the iodide increases the driving force for the ring-opening process.

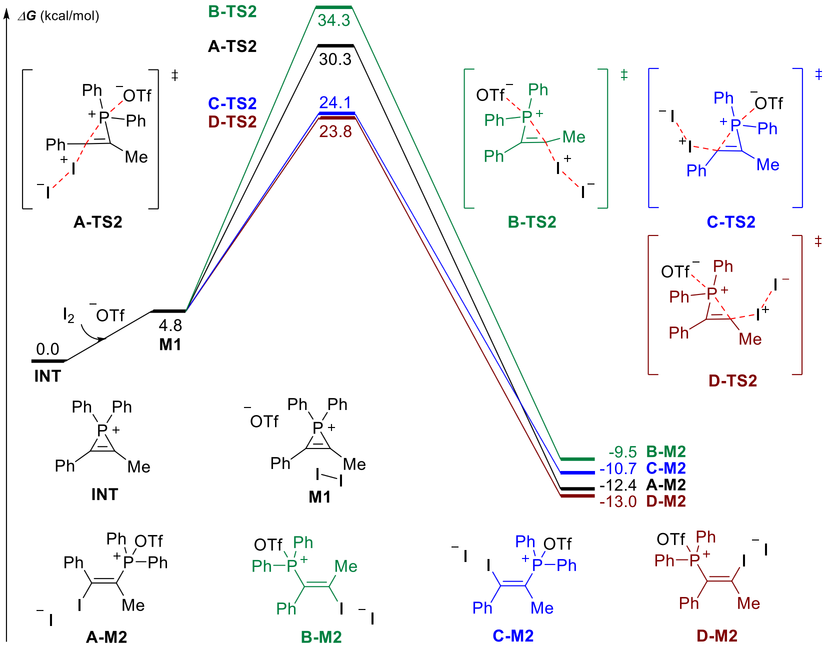

Supplementary Figure 4. The relative Gibbs energy profiles of other possible ring-opening pathways with the presence of TfO$^-$.

7.3 DFT calculations with the direct cleavage of I-I bond

Furthermore, we considered another possible pathway involving the direct cleavage of the I-I bond and computed a concerted transition state, **A-TS3**, in which the cleavage of the C-P bond is coupled with the formation of C-I and P-I bonds. However, the Gibbs energy profile depicted in **Supplementary Figure 5** indicates an extremely high energy barrier. Therefore, this pathway, characterized by the direct cleavage of the I-I bond, can also be safely excluded.

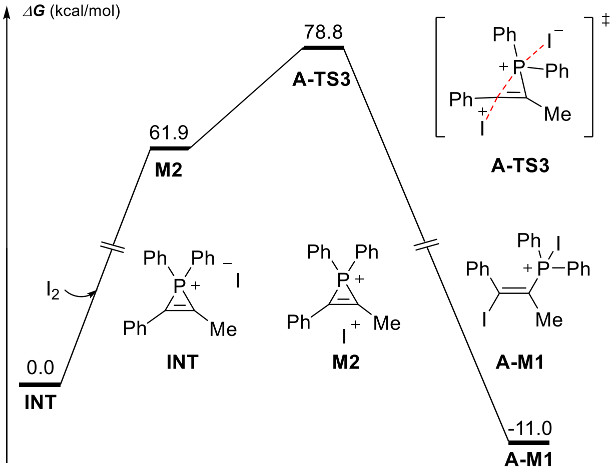

Supplementary Figure 5. The relative Gibbs energy profile of another possible pathway with the direct cleavage of I-I bond.

7.4 Comparison of Δ*G* for A-TS1, B-TS1, C-TS1, and D-TS1 optimized with different methods

To justify the chemoselectivity of the reported reactivity, various methods, including M06-2X, B3LYP-D3 and DSD-PBEP86 were additionally employed for geometry optimization of the key transition states **A-TS1**, **B-TS1**, **C-TS1** and **D-TS1**. It should be noted that **A-TS1**, **B-TS1**, **C-TS1** and **D-TS1** represent the optimized structures by using M06-2X method, while **A-TS1(B3LYP-D3)**,

B-TS1(B3LYP-D3), **C-TS1(B3LYP-D3)** and **D-TS1(B3LYP-D3)** represent the optimized structures by using B3LYP-D3 method, and **A-TS1(DSD-PBEP86)**, **B-TS1(DSD-PBEP86)**, **C-TS1(DSD-PBEP86)** and **D-TS1(DSD-PBEP86)** represent the optimized structures by using DSD-PBEP86 method. *As presented in **Supplementary Table 8**, the calculated results reveal that the pathway associated with **A-TS1** represents the most energetically favourable pathway across various computational methods.*

Supplementary Table 8. Comparison of ΔG for **A-TS1**, **B-TS1**, **C-TS1**, and **D-TS1** optimized with different methods (unit: kcal/mol)

Method	M06-2X	B3LYP-D3	DSD-PBEP86
$\Delta G_{\mathbf{(A\text{-}TS1)}}$	0.0	0.0	0.0
$\Delta G_{\mathbf{(B\text{-}TS1)}}$	5.5	2.1	3.7
$\Delta G_{\mathbf{(C\text{-}TS1)}}$	1.4	2.0	3.0
$\Delta G_{\mathbf{(D\text{-}TS1)}}$	2.1	3.9	5.2

8. Synthetic transformations

8.1 Synthesis of 8

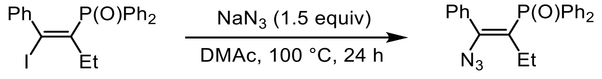

3a **8**, 90% yield

A 10 mL oven-dried sealed tube equipped with a magnetic stir bar was charged with **3a** (0.2 mmol, 1.0 equiv) and NaN_3 (0.3 mmol, 1.5 equiv). The tube was evacuated and backfilled with argon (three times) and DMAc (1.0 mL) was added sequentially via a syringe. The resulting mixture was stirred for 24 h at 100 °C. After cooled to room temperature, the reaction was quenched with water and extracted with diethyl ether (3 x 10 mL). The organic layer was washed with saturated NH_4Cl for 5 times, dried over Na_2SO_4, and volatiles were removed under reduced pressure. The residue was purified by flash column chromatography on silica gel to give the desired product **8** (95% yield).

8.2 Synthesis of 9

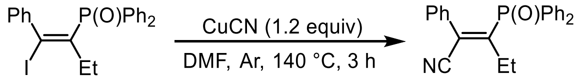

3a **9**, 99% yield

A 10 mL oven-dried sealed tube equipped with a magnetic stir bar was charged with **3a** (0.2 mmol, 1.0 equiv) and CuCN (0.24 mmol, 1.2 equiv). The tube was evacuated and backfilled with argon (three times) and then DMF (1.0 mL) was added, and the resulting mixture was stirred for 3 h at 140 °C. After cooled to ambient temperature, the reaction was quenched with water and extracted with EA (3 x 10 mL). The organic layer was washed with brine, dried over $MgSO_4$, and volatiles were removed under reduced pressure. The residue was purified by flash column chromatography

on silica gel to give the desired product **9** (99% yield).

8.3 Synthesis of 10

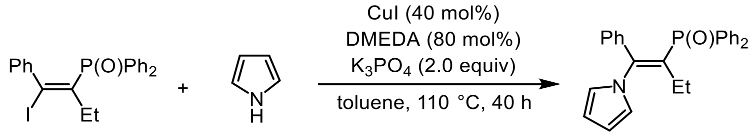

3a **10**, 90% yield

A 10 mL oven-dried sealed tube equipped with a magnetic stir bar was charged with **3a** (0.2 mmol, 1.0 equiv), CuI (0.08 mmol, 0.4 equiv) and K_3PO_4 (0.4 mmol, 2.0 equiv). The tube was evacuated and backfilled with argon (three times) and then toluene (2.0 mL) was added, followed by pyrrole (0.8 mmol, 4.0 equiv) and *N,N'*-dimethyl-1,2-ethanediamine (0.16 mmol, 0.8 equiv) was added. The resulting mixture was stirred for 40 h at 110 °C. After cooled to ambient temperature, volatiles were removed under reduced pressure and the residue was purified by flash column chromatography on silica gel to give the desired product **10** (90% yield).

8.4 Synthesis of 11

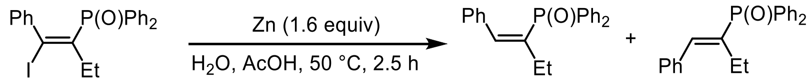

3a **11-1**, 61% yield **11-2**, 30% yield

91% total yield, *Z* / *E* = 2 : 1

A 10 mL oven-dried sealed tube equipped with a magnetic stir bar was charged with **3a** (0.2 mmol, 1.0 equiv) and Zn (0.32 mmol, 1.6 equiv). The tube was evacuated and backfilled with argon (three times) and then H_2O (0.5 mL) and AcOH (0.5 mL) were added sequentially via a syringe. The resulting mixture was stirred for 2.5 h at 50 °C. After cooled to room temperature, the resulting mixture was extracted with ethyl acetate (3 x 10 mL). The organic layer was washed with water, followed by brine, dried over $MgSO_4$, and volatiles were removed under reduced pressure. The residue was purified by flash column chromatography on silica gel to give the desired product **11-1** (61% yield) and the isomer **11-2** (30% yield).

8.5 Synthesis of 12

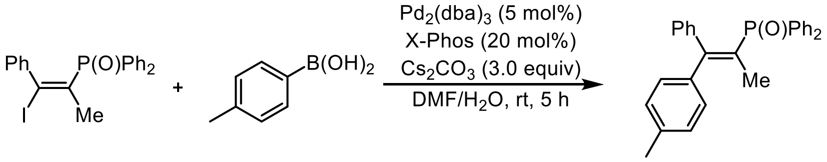

3b **12**, 93% yield

A 10 mL oven-dried sealed tube equipped with a magnetic stir bar was charged with **3b** (0.2 mmol, 1.0 equiv), 4-methylphenylboronic acid (0.4 mmol, 2.0 equiv), $Pd_2(dba)_3$ (0.01 mmol, 5 mol%), X-Phos (0.04 mmol, 20 mol%) and Cs_2CO_3 (0.6 mmol, 3.0 equiv). The tube was evacuated and backfilled with argon (three times) and H_2O (0.4 mL) and DMF (1.6 mL) were added sequentially via a syringe. The resulting mixture was stirred for 5 h at room temperature. After the completion, H_2O was added to the mixture and the resulting mixture was extracted with EA (3 x 10 mL). The organic layer was washed with brine, dried over $MgSO_4$, and volatiles were removed under reduced pressure. The residue was purified by flash column chromatography on silica gel to give the desired

product **12** (93% yield).

8.6 Synthesis of 13

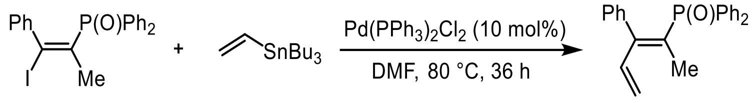

3b **13**, 95% yield

A 10 mL oven-dried sealed tube equipped with a magnetic stir bar was charged with **3b** (0.2 mmol, 1.0 equiv) and $Pd(PPh_3)_2Cl_2$ (0.02 mmol, 10 mol%). The tube was evacuated and backfilled with argon (three times) and DMF (1.0 mL) was added, followed by tributyl(ethenyl)stannane (0.4 mmol, 2.0 equiv) was added via a syringe. The resulting mixture was stirred for 36 h at 80 °C. After cooled to room temperature, the reaction was quenched with water and extracted with EA (3 x 10 mL). The organic layer was washed with brine, dried over $MgSO_4$, and volatiles were removed under reduced pressure. The residue was purified by flash column chromatography on silica gel to give the desired product **13** (95% yield).

8.7 Synthesis of 14

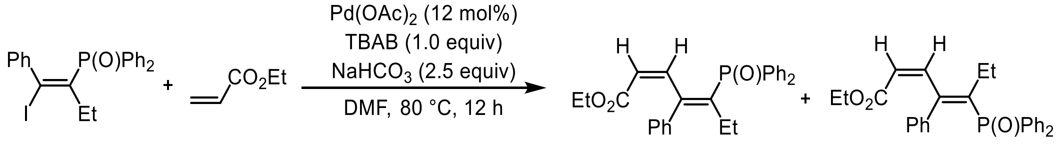

3a **14**, 88% yield
Z,E / *Z,Z* = 5 : 4

A 10 mL oven-dried sealed tube equipped with a magnetic stir bar was charged with **3a** (0.2 mmol, 1.0 equiv), $Pd(OAc)_2$ (0.024 mmol, 0.12 equiv), Bu_4NBr (0.2 mmol, 1.0 equiv) and $NaHCO_3$ (0.5 mmol, 2.5 equiv). The tube was evacuated and backfilled with argon (three times) and then DMF (3.0 mL) was added via a syringe, followed by methyl acrylate (0.66 mmol, 3.3 equiv) was added. The resulting mixture was stirred for 12 h at 80 °C. After cooled to room temperature, the reaction was quenched with aqueous NH_4Cl (5.0 mL), and the resulting mixture was extracted with ethyl acetate (3 x 10 mL). The organic layer was washed with brine, and dried over Na_2SO_4 and volatiles were removed under reduced pressure. The residue was purified by flash column chromatography on silica gel to give the desired product **14** (88% yield, *Z,E*/*Z,Z* = 5:4).

9. Halogen-bond-assisted synthesis of benzo[*b*]phospholes oxides

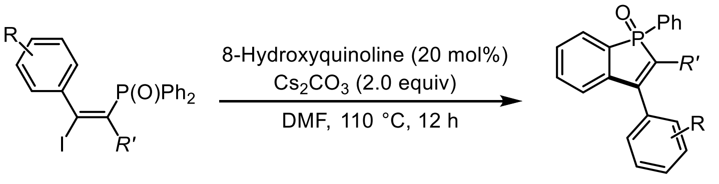

3 or **4** **15**

A 10 mL oven-dried sealed tube equipped with a magnetic stir bar was charged with **3** or **4** (0.10 mmol, 1.0 equiv), 8-hydroxyquinoline (0.02 mmol, 0.2 equiv) and Cs_2CO_3 (0.2 mmol, 2.0 equiv). The tube was evacuated and backfilled with argon (three times) and then DMF (2.0 mL) was added

sequentially via a syringe. The resulting mixture was stirred for 12 h at 110 °C. After cooled to ambient temperature, H_2O was added and the resulting mixture was extracted with EA (3 x 10 mL). The organic layer was washed with brine, and dried over $MgSO_4$ and volatiles were removed under reduced pressure. The residue was purified by flash column chromatography on silica gel to give the desired products **15**.

10. Mechanism experiments for the synthesis of benzo[*b*]phospholes oxides

10.1 Control experiment using TEMPO

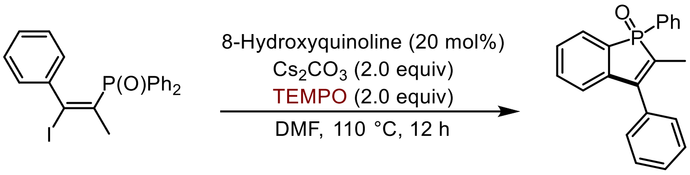

3b **15a**, trace

A 10 mL oven-dried sealed tube equipped with a magnetic stir bar was charged with **3b** (0.10 mmol, 1.0 equiv), 8-hydroxyquinoline (0.02 mmol, 0.2 equiv), Cs_2CO_3 (0.2 mmol, 2.0 equiv) and TEMPO (0.2 mmol, 2.0 equiv). The tube was evacuated and backfilled with argon (three times) and then DMF (2.0 mL) was added sequentially via a syringe. The resulting mixture was stirred at 110 °C for 12 h. After cooled to ambient temperature, H_2O was added and the resulting mixture was extracted with EA (3 x 10 mL). The organic layer was washed with brine, and dried over $MgSO_4$ and volatiles were removed under reduced pressure. Trace of **15a** was detected based on crude ^{1}H-NMR spectrum with 1,3,5-trimethoxybenzene as an internal standard. *This result suggests that the reaction was almost completely inhibited by TEMPO and the cyclization reaction may undergo a free radical pathway.*

10.2 Control experiment without 8-hydroxyquinoline

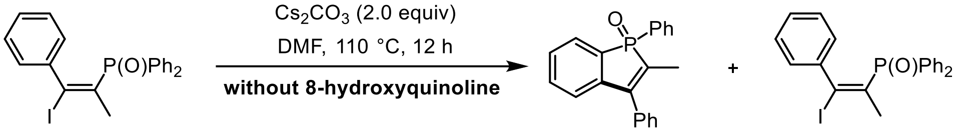

3b **15a**, 45% **3b**, 45% (recovery rate)

A 10 mL oven-dried sealed tube equipped with a magnetic stir bar was charged with **3b** (0.10 mmol, 1.0 equiv) and Cs_2CO_3 (0.2 mmol, 2.0 equiv). The tube was evacuated and backfilled with argon (three times) and then DMF (2.0 mL) was added sequentially via a syringe. The resulting mixture was stirred at 110 °C for 12 h. After cooled to ambient temperature, H_2O was added and the resulting mixture was extracted with EA (3 x 10 mL). The organic layer was washed with brine, and dried over $MgSO_4$ and volatiles were removed under reduced pressure. 45% of **15a** was detected based on crude ^{1}H-NMR spectrum with 1,3,5-trimethoxybenzene as an internal standard. *This result suggests that the addition of 8-hydroxyquinoline may reduce the homogeneous cleavage energy of the C-I bond.*

10.3 Control experiment without Cs_2CO_3

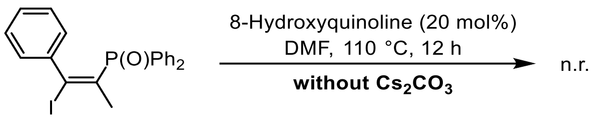

3b

A 10 mL oven-dried sealed tube equipped with a magnetic stir bar was charged with **3b** (0.10 mmol, 1.0 equiv) and 8-hydroxyquinoline (0.02 mmol, 0.2 equiv). The tube was evacuated and backfilled with argon (three times) and then DMF (2.0 mL) was added sequentially via a syringe. The resulting mixture was stirred for 12 h at 110 °C. After cooled to ambient temperature, H_2O was added and the resulting mixture was extracted with EA (3 x 10 mL). The organic layer was washed with brine, and dried over $MgSO_4$ and volatiles were removed under reduced pressure. No desired product **15a** was detected by crude ^{1}H-NMR spectrum. *This result suggests that the addition of base contributes to the deprotonation process.*

10.4 Control experiment using Blue LED and without 8-hydroxyquinoline

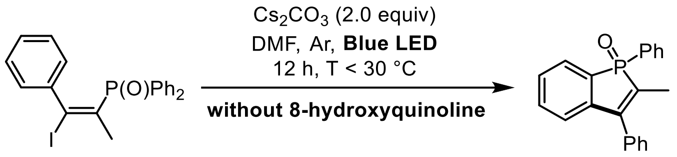

3b **15a**, 70%

A 10 mL oven-dried sealed tube equipped with a magnetic stir bar was charged with **3b** (0.10 mmol, 1.0 equiv) and Cs_2CO_3 (0.2 mmol, 2.0 equiv). The tube was evacuated and backfilled with argon (three times) and then DMF (2.0 mL) was added sequentially via a syringe. The resulting mixture was stirred for 12 h under Blue LED and the reaction temperature was below 30 °C. After the reaction, H_2O was added and the resulting mixture was extracted with EA (3 x 10 mL). The organic layer was washed with brine, and dried over $MgSO_4$ and volatiles were removed under reduced pressure. 70% of **15a** was detected based on crude ^{1}H-NMR spectrum with 1,3,5-trimethoxybenzene as an internal standard. *This result suggests that the cyclization reaction may undergo a free radical pathway.*

10.5 Possible mechanism

Based on the above mechanism experiments, we speculate that the cyclization reaction undergoes a radical process (**Supplementary Figure 6**). First, a halo-bonded complex **A** (XB complex) is formed between 8-hydroxyquinoline and β-iodo vinylphosphine oxides, followed by homogeneous cleavage of the C-I bond to produce a vinyl radical intermediate **B** and an iodine radical. **B** undergoes intramolecular radical cyclization to give **C**, which finally removes the hydrogen atoms in the presence of iodine radicals and base to give the target compound **15**.

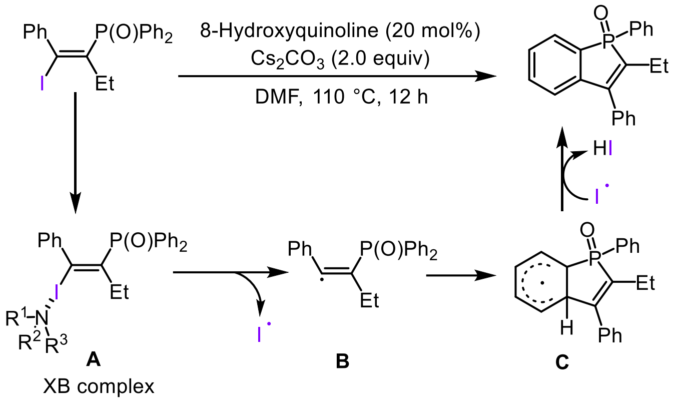

Supplementary Figure 6 Possible mechanism

11. X-ray crystal structures

11.1 X-ray crystal structures analysis of 3a

The structure of **3a** was confirmed X-ray analysis (**Supplementary Figure 7**). The crystal data of compound **3a** was collected at 293 (2) K with graphite monochromatic Cu Kα radiation (λ = 1.54184 Å). The crystal structures were solved and refined by full matrix least-squares methods against F^2 by using Olex2 programs. Crystallographic data for the structure has been deposited to the Cambridge Crystallographic Data Center (CCDC 2240040).

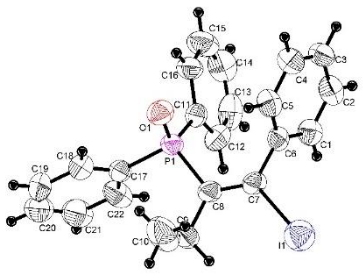

Supplementary Figure 7 ORTEP drawing of **3a** at 50% probability

***Supplementary Table 9*. Crystal data and structure refinement for 3a**

CCDC	2240040
Identification code	20210115
Empirical formula	$C_{22}H_{20}IOP$
Formula weight	458.25
Temperature/K	293(2)
Crystal system	monoclinic
Space group	$P2_1/c$
a/Å	14.9205(4)
b/Å	6.2703(3)
c/Å	21.3259(6)

α/°	90
β/°	91.342(3)
γ/°	90
Volume/Å^3	1994.62(12)
Z	4
ρ_{calc}g/cm^3	1.526
μ/mm^{-1}	13.411
F(000)	912.0
Crystal size/mm^3	0.253 × 0.225 × 0.173
Radiation	CuKα (λ = 1.54184)
2Θ range for data collection/°	8.294 to 134.122
Index ranges	-11 ≤ h ≤ 17, -4 ≤ k ≤ 7, -25 ≤ l ≤ 24
Reflections collected	7239
Independent reflections	3569 [R_{int} = 0.0392, R_{sigma} = 0.0536]
Data/restraints/parameters	3569/0/227
Goodness-of-fit on F^2	1.030
Final R indexes [I>=2σ (I)]	R_1 = 0.0468, wR_2 = 0.1106
Final R indexes [all data]	R_1 = 0.0636, wR_2 = 0.1243
Largest diff. peak/hole / e Å^{-3}	0.50/-0.68

11.2 X-ray crystal structures analysis of 3m

The structure of **3m** was confirmed X-ray analysis (**Supplementary Figure 8**). The crystal data of compound **3m** was collected at 292 K with graphite monochromatic Mo Kα radiation (λ = 0.71073 Å). The crystal structures were solved and refined by full matrix least-squares methods against F^2 by using Olex2 programs. Crystallographic data for the structure has been deposited to the Cambridge Crystallographic Data Center (CCDC 2300801).

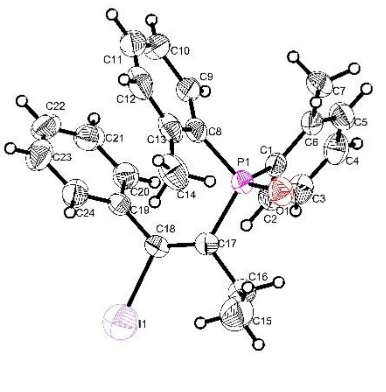

Supplementary Figure 8 ORTEP drawing of **3m** at 50% probability

***Supplementary Table 10*. Crystal data and structure refinement for 3m**

CCDC	2300801
Identification code	3m

Empirical formula	$C_{24}H_{24}IOP$
Formula weight	486.30
Temperature/K	292.00
Crystal system	triclinic
Space group	P-1
a/Å	10.2375(3)
b/Å	10.3407 (3)
c/Å	10.6496(3)
α/°	109.0510(10)
β/°	92.8910(10)
γ/°	93.4060(10)
Volume/Å^3	1060.85(5)
Z	2
ρ_{calc}g/cm^3	1.522
μ/mm^{-1}	1.596
F(000)	488
Crystal size/mm^3	0.3×0.04×0.03
Radiation	MoKα (λ=0.71073 Å)
2Θ range for data collection/°	3.996 to 55.216
Index ranges	$-13 \le h \le 13$, $-13\le k \le 13$, $-13 \le l \le 13$
Reflections collected	32714
Independent reflections	4896 [R_{int} = 0.0600, R_{sigma} = 0.0362]
Data/restraints/parameters	4896/0/247
Goodness-of-fit on F^2	1.033
Final R indexes [I>=2σ (I)]	R_1 = 0.0344, wR_2 = 0.0821
Final R indexes [all data]	R_1 = 0.0466, wR_2 = 0.0875
Largest diff. peak/hole / e Å^{-3}	0.83/-0.61

12. Characteristic data

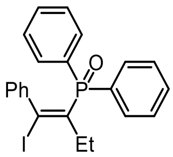

(*E*)-(1-Iodo-1-phenylbut-1-en-2-yl)diphenylphosphine oxide (3a): Light yellow solid (m.p.: 115 - 116 °C); 83% yield; **^{1}H NMR** (400 MHz, $CDCl_3$) δ 7.59 – 7.55 (m, 4H), 7.40 – 7.37 (m, 2H), 7.31 (td, J = 7.6, 2.8 Hz, 4H), 7.09 – 7.07 (m, 2H), 6.96 – 6.89 (m, 3H), 2.70 – 2.61 (m, 2H), 1.05 (t, J = 7.4 Hz, 3H); **^{13}C NMR** (101 MHz, $CDCl_3$) δ 144.1 (d, J = 6.2 Hz), 143.0 (d, J = 79.7 Hz), 133.0 (d, J = 103.6 Hz), 131.36 (d, J = 9.6 Hz), 131.37 (d, J = 2.8 Hz), 128.5 (d, J = 1.0 Hz), 128.2 (d, J = 12.2 Hz), 128.2, 127.3, 120.5 (d, J = 12.6 Hz), 36.9 (d, J = 11.4 Hz), 12.7; **^{31}P NMR** (162 MHz, $CDCl_3$) δ 22.6. HRMS (ESI) calcd for $C_{22}H_{21}IOP^+$ $[M+H]^+$ 459.0369, found 459.0379.

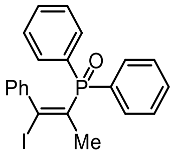

(*E*)-(1-Iodo-1-phenylprop-1-en-2-yl)diphenylphosphine oxide (3b): Colorless oil; 83% yield; **^{1}H NMR** (400 MHz, $CDCl_3$) δ 7.58 – 7.53 (m, 4H), 7.38 – 7.34 (m, 2H), 7.31 – 7.26 (m, 4H), 7.12 – 7.09 (m, 2H), 6.96 – 6.89 (m, 3H), 2.21 (d, J = 11.6 Hz, 3H); **^{13}C NMR** (101 MHz, $CDCl_3$) δ 143.9 (d, J = 6.2 Hz), 137.2 (d, J = 82.5 Hz), 132.9 (d, J = 104.2 Hz), 131.4 (d, J = 2.6 Hz), 131.2 (d, J = 9.7 Hz), 128.6 (d, J = 1.0 Hz), 128.4, 128.3 (d, J = 12.3 Hz), 127.5, 121.8 (d, J = 11.7 Hz), 31.7 (d, J = 12.3 Hz); **^{31}P NMR** (162 MHz, $CDCl_3$) δ 22.8. HRMS (ESI) calcd for $C_{21}H_{19}IOP^+$ $[M+H]^+$ 445.0213, found 445.0216.

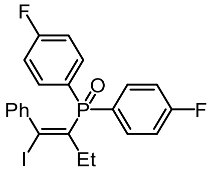

(*E*)-Bis(4-fluorophenyl)(1-iodo-1-phenylbut-1-en-2-yl)phosphine oxide (3c): White solid (m.p.: 98 - 100 °C); 70% yield; **^{1}H NMR** (400 MHz, $CDCl_3$) δ 7.54 – 7.48 (m, 4H), 7.03 – 6.90 (m, 9H), 2.67 – 2.58 (m, 2H), 1.05 (t, J = 7.3 Hz, 3H); **^{13}C NMR** (101 MHz, $CDCl_3$) δ 164.6 (dd, J = 254.4, 2.9 Hz), 144.0 (d, J = 6.3 Hz), 142.7 (d, J = 81.9 Hz), 133.7 (dd, J = 11.0, 8.9 Hz), 128.8 (d, J = 104.1 Hz), 128.4, 127.4, 120.8 (d, J = 13.1 Hz), 115.7 (dd, J = 21.5, 13.4 Hz), 36.9 (d, J = 11.5 Hz), 12.8; **^{31}P NMR** (162 MHz, $CDCl_3$) δ 21.0; **^{19}F NMR** (376 MHz, $CDCl_3$) δ -107.0. HRMS (ESI) calcd for $C_{22}H_{19}F_2IOP^+$ $[M+H]^+$ 495.0181, found 495.0184.

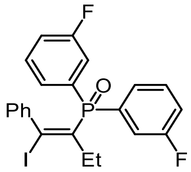

(*E*)-Bis(3-fluorophenyl)(1-iodo-1-phenylbut-1-en-2-yl)phosphine oxide (3d): White solid (m.p.: 112 - 113 °C); 59% yield; **^{1}H NMR** (400 MHz, $CDCl_3$) δ 7.37 – 7.27 (m, 4H), 7.21 – 7.16 (m, 2H), 7.11 – 7.03 (m, 4H), 6.98 – 6.91 (m, 3H), 2.68 – 2.59 (m, 2H), 1.08 (t, J = 7.4 Hz, 3H); **^{13}C NMR** (101 MHz, $CDCl_3$) δ 162.2 (dd, J = 251.0, 17.0 Hz), 143.8 (d, J = 6.5 Hz), 142.2 (d, J = 82.8 Hz),

135.4 (dd, *J* = 103.0, 5.4 Hz), 130.3 (dd, *J* = 14.2, 7.4 Hz), 128.6 – 128.5 (m), 127.4, 126.9 (dd, *J* = 9.2, 3.2 Hz), 121.5 (d, *J* = 13.1 Hz), 118.8 (dd, *J* = 21.2, 2.0 Hz), 118.2 (dd, *J* = 22.7, 10.6 Hz), 36.8 (d, *J* = 11.6 Hz), 12.9; **^{31}P NMR** (162 MHz, $CDCl_3$) δ 19.8; **^{19}F NMR** (376 MHz, $CDCl_3$) δ -111.1 (d, *J* = 5.7 Hz). HRMS (ESI) calcd for $C_{22}H_{19}F_2IOP^+$ $[M+H]^+$ 495.0181, found 495.0182.

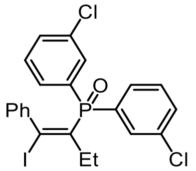

(*E*)-Bis(3-chlorophenyl)(1-iodo-1-phenylbut-1-en-2-yl)phosphine oxide (3e): White solid (m.p.: 119 - 121 °C); 60% yield; **^{1}H NMR** (400 MHz, $CDCl_3$) δ 7.46 – 7.41 (m, 4H), 7.32 (d, *J* = 7.8 Hz, 2H), 7.26 – 7.21 (m, 2H), 7.08 – 7.06 (m, 2H), 6.98 – 6.91 (m, 3H), 2.68 – 2.59 (m, 2H), 1.09 (t, *J* = 7.4 Hz, 3H); **^{13}C NMR** (101 MHz, $CDCl_3$) δ 143.7 (d, *J* = 6.6 Hz), 142.0 (d, *J* = 82.4 Hz), 135.0 (d, *J* = 101.7 Hz), 134.8 (d, *J* = 15.9 Hz), 131.7 (d, *J* = 2.2 Hz), 131.2 (d, *J* = 10.4 Hz), 129.8 (d, *J* = 13.2 Hz), 129.1 (d, *J* = 9.3 Hz), 128.8, 128.5, 127.5, 121.6 (d, *J* = 13.2 Hz), 36.8 (d, *J* = 11.6 Hz), 13.0; **^{31}P NMR** (162 MHz, $CDCl_3$) δ 19.7. HRMS (ESI) calcd for $C_{22}H_{19}Cl_2IOP^+$ $[M+H]^+$ 526.9590, found 526.9594.

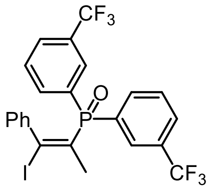

(*E*)-(1-Iodo-1-phenylbut-1-en-2-yl)bis(3-(trifluoromethyl)phenyl)phosphine oxide (3f): White solid (m.p.: 126 - 128 °C); 31% yield; **^{1}H NMR** (400 MHz, $CDCl_3$) δ 7.82 (d, *J* = 12.0 Hz, 2H), 7.75 (dd, *J* = 11.4, 7.8 Hz, 2H), 7.60 (d, *J* = 7.8 Hz, 2H), 7.44 (td, *J* = 7.8, 2.8 Hz, 2H), 7.16 – 7.11 (m, 2H), 6.93 – 6.91 (m, 3H), 2.21 (d, *J* = 12.0 Hz, 3H); **^{13}C NMR** (101 MHz, $CDCl_3$) δ 142.4 (d, *J* = 6.8 Hz), 134.8 (d, *J* = 86.4 Hz), 133.0 (d, *J* = 103.9 Hz), 132.9 (d, *J* = 8.7 Hz), 130.0 (qd, *J* = 33.0, 12.6 Hz), 128.1 (d, *J* = 4.2 Hz), 128.0, 127.8 (d, *J* = 1.5 Hz), 127.4 – 127.2 (m), 126.9 – 126.7 (m), 122.8 (d, *J* = 12.0 Hz), 122.4 (qd, *J* = 273.8, 1.6 Hz), 30.4 (d, *J* = 12.9 Hz); **^{31}P NMR** (162 MHz, $CDCl_3$) δ 18.9; **^{19}F NMR** (377 MHz, $CDCl_3$) δ -62.9. HRMS (ESI) calcd for $C_{23}H_{17}F_6IOP^+$ $[M+H]^+$ 580.9960, found 580.9962.

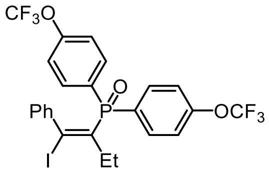

(*E*)-(1-Iodo-1-phenylbut-1-en-2-yl)bis(4-(trifluoromethoxy)phenyl)phosphine oxide (3g): White solid (m.p.: 83 - 85 °C); 51% yield; **^{1}H NMR** (400 MHz, $CDCl_3$) δ 7.57 (dd, *J* = 10.6, 8.7 Hz, 4H), 7.12 (d, *J* = 8.0 Hz, 4H), 7.04 – 7.02 (m, 2H), 6.96 – 6.88 (m, 3H), 2.70 – 2.61 (m, 2H), 1.10 (t, *J* = 7.3 Hz, 3H); **^{13}C NMR** (101 MHz, $CDCl_3$) δ 151.5, 143.8 (d, *J* = 6.2 Hz), 142.4 (d, *J* = 82.6 Hz), 133.2 (d, *J* = 10.6 Hz), 131.4 (d, *J* = 105.7 Hz), 128.6 (d, *J* = 7.2 Hz), 128.5 (overlap one peak of 128.6), 127.5, 121.2 (d, *J* = 13.0 Hz), 120.4 (d, *J* = 12.7 Hz), 120.3 (q, *J* = 259.7 Hz), 36.9 (d, *J* = 11.3 Hz), 12.9; **^{31}P NMR** (162 MHz, $CDCl_3$) δ 20.0; **^{19}F NMR** (376 MHz, $CDCl_3$) δ -57.6. HRMS (ESI) calcd for $C_{24}H_{19}F_6IO_3P^+$ $[M+H]^+$ 627.0015, found 627.0023.

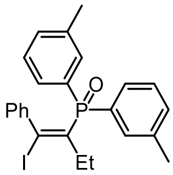

(*E*)-(1-Iodo-1-phenylbut-1-en-2-yl)di-*m*-tolylphosphine oxide (3h): Colorless oil; 32% yield; **^{1}H NMR** (400 MHz, $CDCl_3$) δ 7.36 – 7.31 (m, 4H), 7.21 – 7.16 (m, 4H), 7.05 – 7.03 (m, 2H), 6.94 – 6.88 (m, 3H), 2.69 – 2.59 (m, 2H), 2.27 (s, 6H), 1.05 (t, *J* = 7.4 Hz, 3H); **^{13}C NMR** (101 MHz, $CDCl_3$) δ 144.1 (d, *J* = 6.1 Hz), 143.2 (d, *J* = 79.4 Hz), 138.0 (d, *J* = 12.2 Hz), 132.9 (d, *J* = 103.0 Hz), 132.2 (d, *J* = 2.8 Hz), 132.0 (d, *J* = 9.4 Hz), 128.4 – 128.0 (m), 127.1, 120.1 (d, *J* = 12.9 Hz), 36.9 (d, *J* = 11.3 Hz), 21.4, 12.8; **^{31}P NMR** (162 MHz, $CDCl_3$) δ 23.2. HRMS (ESI) calcd for $C_{24}H_{25}IOP^+$ $[M+H]^+$ 487.0682, found 487.0687.

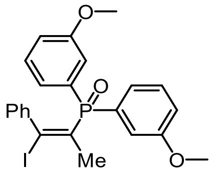

(*E*)-(1-Iodo-1-phenylprop-1-en-2-yl)bis(3-methoxyphenyl)phosphine oxide (3i): White solid (m.p.: 166 - 168 °C); 33% yield; **^{1}H NMR** (400 MHz, $CDCl_3$) δ 7.24 – 7.19 (m, 2H), 7.15 – 7.06 (m, 6H), 6.95 – 6.87 (m, 5H), 3.74 (s, 6H), 2.22 (d, *J* = 11.6 Hz, 3H); **^{13}C NMR** (101 MHz, $CDCl_3$) δ 159.2 (d, *J* = 15.1 Hz), 143.9 (d, *J* = 6.2 Hz), 137.4 (d, *J* = 82.8 Hz), 134.2 (d, *J* = 103.4 Hz), 129.5 (d, *J* = 14.6 Hz), 128.7, 128.3, 127.3, 123.3 (d, *J* = 10.0 Hz), 121.6 (d, *J* = 11.7 Hz), 117.7 (d, *J* = 2.3 Hz), 116.0 (d, *J* = 10.4 Hz), 55.4, 31.6 (d, *J* = 12.3 Hz); **^{31}P NMR** (162 MHz, $CDCl_3$) δ 22.7. HRMS (ESI) calcd for $C_{23}H_{23}IO_3P^+$ $[M+H]^+$ 505.0424, found 505.0419.

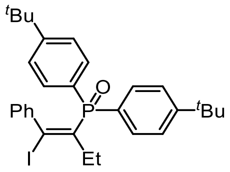

(*E*)-Bis(4-(*tert*-butyl)phenyl)(1-iodo-1-phenylbut-1-en-2-yl)phosphine oxide (3j): White solid (m.p.: 203 - 205 °C); 70% yield; **^{1}H NMR** (400 MHz, $CDCl_3$) δ 7.49 (dd, *J* = 11.6, 8.3 Hz, 4H), 7.30 (dd, *J* = 8.3, 2.6 Hz, 4H), 7.05 – 7.03 (m, 2H), 6.89 – 6.85 (m, 3H), 2.73 – 2.64 (m, 2H), 1.29 (s, 18H), 1.08 (t, *J* = 7.3 Hz, 3H); **^{13}C NMR** (101 MHz, $CDCl_3$) δ 154.5 (d, *J* = 2.7 Hz), 144.2 (d, *J* = 6.1 Hz), 143.7 (d, *J* = 79.6 Hz), 131.3 (d, *J* = 10.1 Hz), 129.8 (d, *J* = 105.9 Hz), 128.5, 128.0, 127.2, 125.2 (d, *J* = 12.4 Hz), 119.6 (d, *J* = 13.0 Hz), 37.0 (d, *J* = 11.2 Hz), 34.9, 31.1, 12.8; **^{31}P NMR** (162 MHz, $CDCl_3$) δ 22.8. HRMS (ESI) calcd for $C_{30}H_{37}IOP^+$ $[M+H]^+$ 571.1621, found 571.1628.

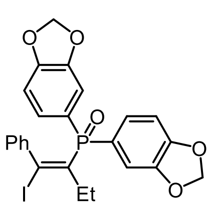

(*E*)-Bis(benzo[*d*][1,3]dioxol-5-yl)(1-iodo-1-phenylbut-1-en-2-yl)phosphine oxide (3k): Colorless oil; 50% yield; **^{1}H NMR** (400 MHz, $CDCl_3$) δ 7.12 – 7.06 (m, 2H), 7.04 – 7.01 (m, 2H),

6.97 – 6.94 (m, 3H), 6.84 (dd, *J* = 11.5, 1.3 Hz, 2H), 6.72 (dd, *J* = 8.0, 2.4 Hz, 2H), 5.94 (s, 4H), 2.70 – 2.60 (m, 2H), 1.08 (t, *J* = 7.4 Hz, 3H); **^{13}C NMR** (101 MHz, $CDCl_3$) δ 150.4 (d, *J* = 2.9 Hz), 147.5 (d, *J* = 18.5 Hz), 144.3 (d, *J* = 6.4 Hz), 143.1 (d, *J* = 81.6 Hz), 128.3 (d, *J* = 1.1 Hz), 128.0, 127.3, 126.8 (d, *J* = 10.9 Hz), 126.1 (d, *J* = 108.7 Hz), 119.8 (d, *J* = 13.1 Hz), 110.9 (d, *J* = 13.0 Hz), 108.5 (d, *J* = 15.4 Hz), 101.5, 37.0 (d, *J* = 11.2 Hz), 12.9; **^{31}P NMR** (162 MHz, $CDCl_3$) δ 23.2. HRMS (ESI) calcd for $C_{24}H_{21}IO_5P^+$ $[M+H]^+$ 547.0166, found 547.0172.

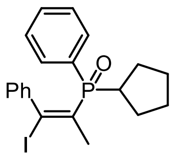

(*E*)-Cyclopentyl(1-iodo-1-phenylprop-1-en-2-yl)(phenyl)phosphine oxide (3l): Colorless oil; 45% yield; **^{1}H NMR** (400 MHz, $CDCl_3$) δ 7.42 – 7.36 (m, 3H), 7.32 – 7.28 (m, 2H), 7.17 – 7.09 (m, 3H), 7.02 – 7.01 (m, 2H), 2.39 (d, *J* = 10.4 Hz, 3H), 2.32 – 2.19 (m, 1H), 2.01 – 1.83 (m, 2H), 1.72 – 1.45 (m, 6H); **^{13}C NMR** (101 MHz, $CDCl_3$) δ 144.6 (d, *J* = 5.5 Hz), 138.9 (d, *J* = 73.0 Hz), 133.8 (d, *J* = 96.0 Hz), 131.1 (d, *J* = 2.2 Hz), 130.4 (d, *J* = 9.2 Hz), 128.3, 128.2 (d, *J* = 11.5 Hz), 128.1, 127.5, 118.7 (d, *J* = 10.0 Hz), 37.0 (d, *J* = 74.6 Hz), 30.3 (d, *J* = 10.6 Hz), 27.0, 26.8 - 26.6 (m); **^{31}P NMR** (162 MHz, $CDCl_3$) δ 34.0. HRMS (ESI) calcd for $C_{20}H_{23}IOP^+$ $[M+H]^+$ 437.0526, found 437.0529.

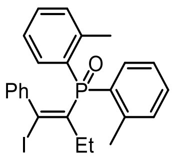

(*E*)-(1-Iodo-1-phenylbut-1-en-2-yl)di-*o*-tolylphosphine oxide (3m): White solid (m.p.: 155 - 157 °C); 6 % NMR yield; **^{1}H NMR** (400 MHz, $CDCl_3$) δ 7.32 – 7.26 (m, 2H), 7.19 – 7.03 (m, 6H), 6.89 – 6.80 (m, 5H), 2.86 – 2.77 (m, 2H), 2.52 (s, 6H), 1.17 (t, *J* = 7.3 Hz, 3H); **^{13}C NMR** (101 MHz, $CDCl_3$) δ 144.5 (d, *J* = 5.8 Hz), 143.3 (d, *J* = 7.8 Hz), 141.4 (d, *J* = 77.3 Hz), 132.3 (d, *J* = 13.6 Hz), 131.9 (d, *J* = 10.5 Hz), 131.6 (d, *J* = 2.2 Hz), 130.3 (d, *J* = 102.4 Hz), 127.9, 127.4, 126.8, 125.2 (d, *J* = 13.1 Hz), 118.0 (d, *J* = 12.3 Hz), 37.2 (d, *J* = 9.8 Hz), 21.9 (d, *J* = 4.3 Hz), 12.5; **^{31}P NMR** (162 MHz, $CDCl_3$) δ 31.6. HRMS (ESI) calcd for $C_{24}H_{25}IOP^+$ $[M+H]^+$ 487.0682, found 487.0685.

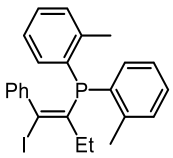

(*E*)-(1-iodo-1-phenylbut-1-en-2-yl)di-*o*-tolylphosphane (3m'): White solid (m.p.: 136 - 138 °C); 59% yield; **^{1}H NMR** (400 MHz, $CDCl_3$) δ 7.31 – 7.11 (m, 13H), 2.56 (qd, *J* = 7.4, 3.2 Hz, 2H), 2.24 (s, 6H), 0.54 (t, *J* = 7.4 Hz, 3H); **^{13}C NMR** (101 MHz, $CDCl_3$) δ 145.6 (d, *J* = 10.2 Hz), 144.1 (d, *J* = 29.1 Hz), 142.2 (d, *J* = 26.6 Hz), 134.8 (d, *J* = 18.2 Hz), 132.3, 130.1 (d, *J* = 4.2 Hz), 128.6, 127.9 (d, *J* = 9.6 Hz), 127.9, 127.7, 126.1, 114.0 (d, *J* = 44.6 Hz), 36.5, 21.2 (d, *J* = 21.5 Hz), 12.0; **^{31}P NMR** (162 MHz, $CDCl_3$) δ -22.2. HRMS (ESI) calcd for $C_{24}H_{25}IP^+$ $[M+H]^+$ 471.0733, found 471.0735.

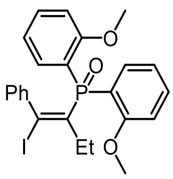

(*E*)-(1-Iodo-1-phenylbut-1-en-2-yl)bis(2-methoxyphenyl)phosphine oxide (3n): Oil; 30 % yield; **^{1}H NMR** (400 MHz, $CDCl_3$) δ 7.33 (t, *J* = 7.7 Hz, 2H), 7.28 – 7.23 (m, 2H), 7.00 – 6.98 (m, 2H), 6.85 – 6.75 (m, 7H), 3.76 (s, 6H), 2.78 – 2.69 (m, 2H), 1.12 (t, *J* = 7.3 Hz, 3H); **^{13}C NMR** (101 MHz, $CDCl_3$) δ 160.8 (d, *J* = 2.4 Hz), 144.0 (d, *J* = 6.5 Hz), 143.6 (d, *J* = 86.1 Hz), 134.1 (d, *J* = 8.5 Hz), 133.3 (d, *J* = 1.9 Hz), 128.3 (d, *J* = 1.5 Hz), 127.8, 126.6, 121.1 (d, *J* = 106.7 Hz), 120.4 (d, *J* = 12.5 Hz), 117.1 (d, *J* = 14.2 Hz), 110.8 (d, *J* = 6.6 Hz), 55.3, 36.4 (d, *J* = 11.8 Hz), 12.7; **^{31}P NMR** (162 MHz, $CDCl_3$) δ 21.3. HRMS (ESI) calcd for $C_{24}H_{25}IO_3P^+$ $[M+H]^+$ 519.0581, found 519.0584.

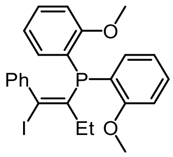

(*E*)-(1-iodo-1-phenylbut-1-en-2-yl)bis(2-methoxyphenyl)phosphane (3n'): White solid (m.p.: 129 - 131 °C); 44% yield; **^{1}H NMR** (400 MHz, $CDCl_3$) δ 7.32 (t, *J* = 7.5 Hz, 2H), 7.27 - 7.25 (m, 2H) (overlap one peak of 7.26), 7.21 (t, *J* = 6.9 Hz, 2H), 7.17 - 7.13 (m, 1H), 7.01 - 6.99 (m, 2H), 6.91 (t, *J* =7.3 Hz, 2H), 6.80 (dd, *J* =8.1, 4.8 Hz, 2H), 3.72 (s, 6H), 2.47 (qd, *J* = 7.4, 3.5 Hz, 2H), 0.59 (t, *J* = 7.4 Hz, 3H); **^{13}C NMR** (101 MHz, $CDCl_3$) δ 161.1 (d, *J* = 16.6 Hz), 145.6 (d, *J* = 9.8 Hz), 144.8 (d, *J* = 28.2 Hz), 133.1, 130.0, 128.3 (d, *J* = 3.9 Hz), 127.8, 127.7, 123.8 (d, *J* = 18.3 Hz), 120.8, 112.5 (d, *J* = 42.8 Hz), 109.8, 55.4, 36.5, 12.2; **^{31}P NMR** (162 MHz, $CDCl_3$) δ -26.7. HRMS (ESI) calcd for $C_{24}H_{25}IO_2P^+$ $[M+H]^+$ 503.0631, found 503.0641.

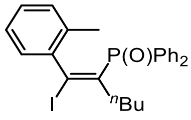

(*E*)-(1-Iodo-1-(*o*-tolyl)hex-1-en-2-yl)diphenylphosphine oxide (4a): Yellow solid (m.p.: 105 - 107 °C); 77% yield; **^{1}H NMR** (400 MHz, $CDCl_3$) δ 7.65 – 7.60 (m, 2H), 7.47 – 7.34 (m, 6H), 7.26 – 7.22 (m, 2H), 6.91 – 6.81 (m, 3H), 6.71 (d, *J* = 7.3 Hz, 1H), 2.68 – 2.51 (m, 2H), 2.07 (s, 3H), 1.59 – 1.39 (m, 2H), 1.26 – 1.20 (m, 2H), 0.79 (t, *J* = 7.3 Hz, 3H); **^{13}C NMR** (101 MHz, $CDCl_3$) δ 143.1 (d, *J* = 5.9 Hz), 141.0 (d, *J* = 79.8 Hz), 134.8, 132.8 (d, *J* = 103.6 Hz), 132.7 (d, *J* = 102.9 Hz), 131.6 (d, *J* = 2.8 Hz), 131.5 (d, *J* = 2.0 Hz), 131.4 (d, *J* = 2.0 Hz), 131.3 (d, *J* = 2.8 Hz), 130.0, 128.7, 128.3 (d, *J* = 12.2 Hz), 128.0 (d, *J* = 12.3 Hz), 127.6 (d, *J* = 1.3 Hz), 125.0, 120.1 (d, *J* = 13.5 Hz), 42.7 (d, *J* = 11.0 Hz), 30.1, 22.8, 20.0, 13.6; **^{31}P NMR** (162 MHz, $CDCl_3$) δ 22.8. HRMS (ESI) calcd for $C_{25}H_{27}IOP^+$ $[M+H]^+$ 501.0839, found 501.0852.

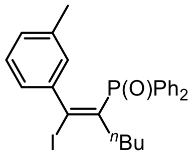

(*E*)-(1-Iodo-1-(*m*-tolyl)hex-1-en-2-yl)diphenylphosphine oxide (4b): Colorless oil; 70% yield; **^{1}H NMR** (400 MHz, $CDCl_3$) δ 7.57 – 7.52 (m, 4H), 7.37 – 7.33 (m, 2H), 7.30 – 7.25 (m, 4H), 6.93 (d, *J* = 7.7 Hz, 1H), 6.82 (t, *J* = 7.6 Hz, 1H), 6.74 (s, 1H), 6.69 (d, *J* = 7.6 Hz, 1H), 2.59 – 2.51 (m, 2H), 2.02 (s, 3H), 1.53 – 1.46 (m, 2H), 1.23 – 1.14 (m, 2H), 0.76 (t, *J* = 7.3 Hz, 3H); **^{13}C NMR** (101

MHz, $CDCl_3$) δ 143.9 (d, *J* = 6.5 Hz), 142.0 (d, *J* = 80.2 Hz), 136.7, 133.1 (d, *J* = 103.5 Hz), 131.3 (d, *J* = 9.6 Hz), 131.2 (d, *J* = 2.8 Hz), 129.2 (d, *J* = 1.0 Hz), 129.1, 128.0 (d, *J* = 12.1 Hz), 127.3, 125.8 (d, *J* = 1.1 Hz), 120.9 (d, *J* = 13.0 Hz), 43.2 (d, *J* = 11.2 Hz), 30.2, 22.7, 21.1, 13.6; **^{31}P NMR** (162 MHz, $CDCl_3$) δ 22.2. HRMS (ESI) calcd for $C_{25}H_{27}IOP^+$ $[M+H]^+$ 501.0839, found 501.0833.

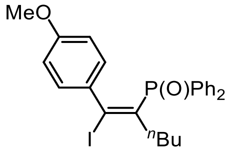

(*E*)-(1-Iodo-1-(4-methoxyphenyl)hex-1-en-2-yl)diphenylphosphine oxide (4c): Colorless oil; 58% yield; **^{1}H NMR** (400 MHz, $CDCl_3$) δ 7.58 – 7.53 (m, 4H), 7.38 – 7.35 (m, 2H), 7.31 – 7.26 (m, 4H), 7.03 (d, *J* = 8.7 Hz, 2H), 6.40 (d, *J* = 8.7 Hz, 2H), 3.66 (s, 3H), 2.54 – 2.46 (m, 2H), 1.49 – 1.41 (m, 2H), 1.20 – 1.11 (m, 2H), 0.73 (t, *J* = 7.3 Hz, 3H); **^{13}C NMR** (101 MHz, $CDCl_3$) δ 159.2, 141.8 (d, *J* = 80.0 Hz), 136.8 (d, *J* = 6.4 Hz), 133.2 (d, *J* = 103.4 Hz), 131.4 – 131.3 (m), 130.4, 128.1 (d, *J* = 12.1 Hz), 121.5 (d, *J* = 13.0 Hz), 112.6, 55.2, 43.2 (d, *J* = 11.4 Hz), 30.2, 22.6, 13.5; **^{31}P NMR** (162 MHz, $CDCl_3$) δ 23.0. HRMS (ESI) calcd for $C_{25}H_{27}IO_2P^+$ $[M+H]^+$ 517.0788, found 517.0795.

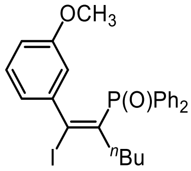

(*E*)-(1-Iodo-1-(3-methoxyphenyl)hex-1-en-2-yl)diphenylphosphine oxide (4d): Colorless oil; 29% yield; **^{1}H NMR** (400 MHz, $CDCl_3$) δ 7.57 – 7.52 (m, 4H), 7.36 (t, *J* = 7.3 Hz, 2H), 7.30 – 7.26 (m, 4H), 6.81 (t, *J* = 7.9 Hz, 1H), 6.68 (d, *J* = 7.6 Hz, 1H), 6.57 (s, 1H), 6.44 (dd, *J* = 8.2, 2.3 Hz, 1H), 3.61 (s, 3H), 2.59 – 2.51 (m, 2H), 1.52 – 1.45 (m, 2H), 1.22 – 1.13 (m, 2H), 0.75 (t, *J* = 7.3 Hz, 3H); **^{13}C NMR** (101 MHz, $CDCl_3$) δ 157.9, 145.1 (d, *J* = 6.2 Hz), 141.9 (d, *J* = 81.5 Hz), 132.7 (d, *J* = 102.4 Hz), 131.4 – 131.3 (m), 128.4, 128.2 (d, *J* = 12.2 Hz), 121.4, 120.4 (d, *J* = 13.2 Hz), 115.1, 113.2, 55.1, 43.2 (d, *J* = 11.0 Hz), 30.2, 22.6, 13.6; **^{31}P NMR** (162 MHz, $CDCl_3$) δ 22.9. HRMS (ESI) calcd for $C_{25}H_{27}IO_2P^+$ $[M+H]^+$ 517.0788, found 517.0810.

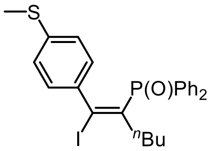

(*E*)-((4-(2-(Diphenylphosphoryl)-1-iodohex-1-en-1-yl)phenyl)-l6-methylene)sulfane (4e): Colorless oil; 50% yield; **^{1}H NMR** (400 MHz, $CDCl_3$) δ 7.57 – 7.52 (m, 4H), 7.38 (t, *J* = 7.3 Hz, 2H), 7.32 – 7.26 (m, 4H), 6.97 (d, *J* = 8.4 Hz, 2H), 6.73 (d, *J* = 8.3 Hz, 2H), 2.56 – 2.48 (m, 2H), 2.35 (s, 3H), 1.49 – 1.41 (m, 2H), 1.21 – 1.12 (m, 2H), 0.74 (t, *J* = 7.3 Hz, 3H); **^{13}C NMR** (101 MHz, $CDCl_3$) δ 142.4 (d, *J* = 80.0 Hz), 140.7 (d, *J* = 6.4 Hz), 139.2, 133.0 (d, *J* = 103.6 Hz), 131.4 – 131.3 (m), 129.0, 128.2 (d, *J* = 12.2 Hz), 124.7, 120.3 (d, *J* = 12.7 Hz), 43.2 (d, *J* = 11.1 Hz), 30.1, 22.6, 15.3, 13.5; **^{31}P NMR** (162 MHz, $CDCl_3$) δ 22.4. HRMS (ESI) calcd for $C_{25}H_{27}IOPS^+$ $[M+H]^+$ 533.0559, found 533.0565.

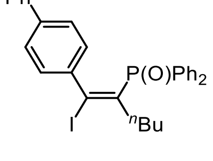

(*E*)-(1-([1,1'-Biphenyl]-4-yl)-1-iodohex-1-en-2-yl)diphenylphosphine oxide (4f): Colorless oil; 83% yield; **^{1}H NMR** (400 MHz, $CDCl_3$) δ 7.60 – 7.55 (m, 4H), 7.41 – 7.26 (m, 11H), 7.14 – 7.08 (m, 4H), 2.63 – 2.55 (m, 2H), 1.57 – 1.49 (m, 2H), 1.26 – 1.17 (m, 2H), 0.78 (t, *J* = 7.3 Hz, 3H); **^{13}C NMR** (101 MHz, $CDCl_3$) δ 143.1 (d, *J* = 6.6 Hz), 142.6 (d, *J* = 80.0 Hz), 140.8, 140.5, 133.0 (d, *J* = 103.4 Hz), 131.4 (d, *J* = 9.6 Hz), 131.3 (d, *J* = 2.8 Hz), 129.1 (d, *J* = 1.0 Hz), 128.8, 128.2 (d, *J* = 12.2 Hz), 127.5, 127.0, 126.0, 120.2 (d, *J* = 12.9 Hz), 43.2 (d, *J* = 11.1 Hz), 30.2, 22.7, 13.6; **^{31}P NMR** (162 MHz, $CDCl_3$) δ 22.4. HRMS (ESI) calcd for $C_{30}H_{29}IOP^+$ $[M+H]^+$ 563.0995, found 563.1000.

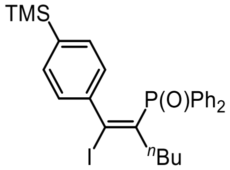

(*E*)-(1-Iodo-1-(4-(trimethylsilyl)phenyl)hex-1-en-2-yl)diphenylphosphine oxide (4g): Yellow solid (m.p.: 96 - 98 °C); 67% yield; **^{1}H NMR** (400 MHz, $CDCl_3$) δ 7.58 – 7.53 (m, 4H), 7.38 – 7.34 (m, 2H), 7.30 – 7.25 (m, 4H), 7.05 (s, 4H), 2.62 – 2.54 (m, 2H), 1.56 – 1.49 (m, 2H), 1.26 – 1.17 (m, 2H), 0.78 (t, *J* = 7.3 Hz, 3H), 0.18 (s, 9H); **^{13}C NMR** (101 MHz, $CDCl_3$) δ 144.2 (d, *J* = 6.5 Hz), 142.1 (d, *J* = 80.2 Hz), 140.7, 133.0 (d, *J* = 103.4 Hz), 132.2, 131.4 (d, *J* = 9.7 Hz), 131.2 (d, *J* = 2.8 Hz), 128.1 (d, *J* = 12.2 Hz), 127.7, 120.8 (d, *J* = 12.9 Hz), 43.2 (d, *J* = 11.1 Hz), 30.2, 22.7, 13.6, -1.3; **^{31}P NMR** (162 MHz, $CDCl_3$) δ 22.3. HRMS (ESI) calcd for $C_{27}H_{33}IOPSi^+$ $[M+H]^+$ 559.1077, found 559.1078.

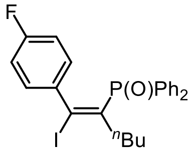

(*E*)-(1-(4-Fluorophenyl)-1-iodohex-1-en-2-yl)diphenylphosphine oxide (4h): Yellow solid (m.p.: 124 - 126 °C); 67% yield; **^{1}H NMR** (400 MHz, $CDCl_3$) δ 7.59 – 7.54 (m, 4H), 7.42 – 7.38 (m, 2H), 7.34 – 7.30 (m, 4H), 7.08 – 7.05 (m, 2H), 6.61 – 6.57 (m, 2H), 2.53 – 2.45 (m, 2H), 1.46 – 1.38 (m, 2H), 1.19 – 1.09 (m, 2H), 0.72 (t, *J* = 7.3 Hz, 3H); **^{13}C NMR** (101 MHz, $CDCl_3$) δ 162.0 (d, *J* = 250.2 Hz), 143.3 (d, *J* = 79.3 Hz), 140.2 (dd, *J* = 6.5, 3.4 Hz), 132.9 (d, *J* = 103.4 Hz), 131.5 (d, *J* = 2.6 Hz), 131.3 (d, *J* = 9.6 Hz), 130.84 – 130.76 (m), 128.2 (d, *J* = 12.3 Hz), 119.3 (d, *J* = 12.3 Hz), 114.2 (d, *J* = 22.0 Hz), 43.2 (d, *J* = 11.2 Hz), 30.0, 22.6, 13.5; **^{31}P NMR** (162 MHz, $CDCl_3$) δ 22.1; **^{19}F NMR** (376 MHz, $CDCl_3$) δ -112.5. HRMS (ESI) calcd for $C_{24}H_{24}FIOP^+$ $[M+H]^+$ 505.0588, found 505.0595.

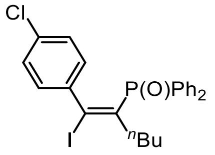

(*E*)-(1-(4-Chlorophenyl)-1-iodohex-1-en-2-yl)diphenylphosphine oxide (4i): Yellow solid (m.p.: 118 - 120 °C); 52% yield; **^{1}H NMR** (400 MHz, $CDCl_3$) δ 7.58 – 7.53 (m, 4H), 7.43 – 7.40 (m, 2H), 7.32 (td, *J* = 7.6, 2.9 Hz, 4H), 6.99 (d, *J* = 8.6 Hz, 2H), 6.87 (d, *J* = 8.6 Hz, 2H), 2.54 – 2.46 (m, 2H), 1.45 – 1.38 (m, 2H), 1.20 – 1.10 (m, 2H), 0.73 (t, *J* = 7.3 Hz, 3H); **^{13}C NMR** (101 MHz, $CDCl_3$) δ 143.4 (d, *J* = 79.2 Hz), 142.5 (d, *J* = 6.5 Hz), 134.1, 132.8 (d, *J* = 103.5 Hz), 131.5 (d, *J* = 2.6 Hz), 131.4 (d, *J* = 9.6 Hz), 130.0 (d, *J* = 1.0 Hz), 128.3 (d, *J* = 12.3 Hz), 127.4, 118.6 (d, *J* = 12.3 Hz),

43.1 (d, *J* = 11.2 Hz), 30.0, 22.6, 13.5; **^{31}P NMR** (162 MHz, $CDCl_3$) δ 22.3. HRMS (ESI) calcd for $C_{24}H_{24}ClIOP^+$ $[M+H]^+$ 521.0292, found 521.0296.

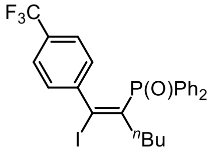

(*E*)-(1-Iodo-1-(4-(trifluoromethyl)phenyl)hex-1-en-2-yl)diphenylphosphine oxide (4j): Colorless oil; 9% yield; **^{1}H NMR** (400 MHz, $CDCl_3$) δ 7.57 – 7.52 (m, 4H), 7.41 (t, *J* = 7.3 Hz, 2H), 7.31 (td, *J* = 7.6, 2.7 Hz, 4H), 7.16 (s, 4H), 2.58 – 2.50 (m, 2H), 1.48 – 1.40 (m, 2H), 1.22 – 1.12 (m, 2H), 0.74 (t, *J* = 7.3 Hz, 3H); **^{13}C NMR** (101 MHz, $CDCl_3$) δ 147.2 (d, *J* = 5.6 Hz), 144.1 (d, *J* = 78.5 Hz), 132.6 (d, *J* = 103.4 Hz), 131.6 (d, *J* = 2.5 Hz), 131.3 (d, *J* = 9.7 Hz), 129.7 (q, *J* = 32.5 Hz), 128.9, 128.3 (d, *J* = 12.2 Hz), 124.2 (q, *J* = 3.6 Hz), 123.6 (q, *J* = 273.3 Hz), 117.4 (d, *J* = 11.9 Hz), 43.1 (d, *J* = 11.0 Hz), 30.0, 22.6, 13.5; **^{31}P NMR** (162 MHz, $CDCl_3$) δ 22.2; **^{19}F NMR** (376 MHz, $CDCl_3$) δ -63.0. HRMS (ESI) calcd for $C_{25}H_{24}F_3IOP^+$ $[M+H]^+$ 555.0556, found 555.0562.

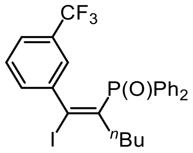

(*E*)-(1-Iodo-1-(3-(trifluoromethyl)phenyl)hex-1-en-2-yl)diphenylphosphine oxide (4k): Yellow solid (m.p.: 118 - 120 °C); 12% yield; **^{1}H NMR** (400 MHz, $CDCl_3$) δ 7.56 (dd, *J* = 11.2, 7.8 Hz, 4H), 7.39 (d, *J* = 6.8 Hz, 3H), 7.33 – 7.30 (m, 4H), 7.22 (s, 1H), 7.18 (d, *J* = 7.8 Hz, 1H), 7.10 (t, *J* = 7.6 Hz, 1H), 2.56 – 2.48 (m, 2H), 1.49 – 1.42 (m, 2H), 1.20 – 1.10 (m, 2H), 0.73 (t, *J* = 7.3 Hz, 3H); **^{13}C NMR** (101 MHz, $CDCl_3$) δ 144.6 (d, *J* = 78.7 Hz), 144.5 (d, *J* = 6.4 Hz), 132.6 (d, *J* = 103.7 Hz), 132.2, 131.7, 131.2 (d, *J* = 9.5 Hz), 129.4 (q, *J* = 32.6 Hz), 128.3 (d, *J* = 12.0 Hz), 127.9, 125.6 (q, *J* = 3.7 Hz), 124.9 (q, *J* = 3.8 Hz), 123.5 (q, *J* = 273.5 Hz), 117.6 (d, *J* = 11.9 Hz), 43.2 (d, *J* = 11.0 Hz), 29.9, 22.6, 13.5; **^{31}P NMR** (162 MHz, $CDCl_3$) δ 21.9. HRMS (ESI) calcd for $C_{25}H_{24}F_3IOP^+$ $[M+H]^+$ 555.0556, found 555.0562.

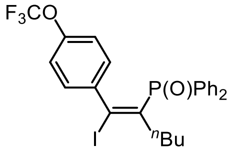

(*E*)-(1-Iodo-1-(4-(trifluoromethoxy)phenyl)hex-1-en-2-yl)diphenylphosphine oxide (4l): Yellow solid (m.p.: 86 - 88 °C); 38% yield; **^{1}H NMR** (400 MHz, $CDCl_3$) δ 7.59 – 7.54 (m, 4H), 7.42 – 7.38 (m, 2H), 7.34 – 7.29 (m, 4H), 7.13 – 7.10 (m, 2H), 6.74 (d, *J* = 8.0 Hz, 2H), 2.55 – 2.47 (m, 2H), 1.49 – 1.41 (m, 2H), 1.20 – 1.11 (m, 2H), 0.73 (t, *J* = 7.3 Hz, 3H); **^{13}C NMR** (101 MHz, $CDCl_3$) δ 148.5 – 148.4 (m), 144.0 (d, *J* = 78.9 Hz), 142.6 (d, *J* = 6.7 Hz), 132.7 (d, *J* = 103.5 Hz), 131.5 (d, *J* = 2.7 Hz), 131.3 (d, *J* = 9.6 Hz), 130.5, 128.3 (d, *J* = 12.1 Hz), 120.3 (q, *J* = 258.7 Hz), 119.5, 118.3 (d, *J* = 12.3 Hz), 43.2 (d, *J* = 11.2 Hz), 30.0, 22.6, 13.5; **^{31}P NMR** (162 MHz, $CDCl_3$) δ 21.9; **^{19}F NMR** (376 MHz, $CDCl_3$) δ -57.6. HRMS (ESI) calcd for $C_{25}H_{24}F_3IO_2P^+$ $[M+H]^+$ 571.0505, found 571.0514.

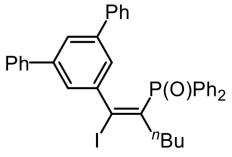

(*E*)-(1-([1,1':3',1''-Terphenyl]-5'-yl)-1-iodohex-1-en-2-yl)diphenylphosphine oxide (4m): Yellow solid (m.p.: 136 - 137 °C); 57% yield; **^{1}H NMR** (400 MHz, $CDCl_3$) δ 7.69 – 7.64 (m, 4H), 7.54 – 7.52 (m, 4H), 7.47 (t, *J* = 7.3 Hz, 4H), 7.40 – 7.37 (m, 5H), 7.33 – 7.26 (m, 6H), 2.68 – 2.60 (m, 2H), 1.65 – 1.57 (m, 2H), 1.29 – 1.19 (m, 2H), 0.81 (t, *J* = 7.3 Hz, 3H); **^{13}C NMR** (101 MHz, $CDCl_3$) δ 144.8 (d, *J* = 6.7 Hz), 143.4 (d, *J* = 80.0 Hz), 140.6, 140.3, 132.7 (d, *J* = 103.5 Hz), 131.3 (d, *J* = 2.5 Hz), 131.2 (d, *J* = 9.5 Hz), 128.8, 128.1 (d, *J* = 12.1 Hz), 127.5, 127.3, 126.9, 125.9, 120.3 (d, *J* = 12.7 Hz), 43.3 (d, *J* = 11.2 Hz), 30.3, 22.7, 13.6; **^{31}P NMR** (162 MHz, $CDCl_3$) δ 22.4. HRMS (ESI) calcd for $C_{36}H_{33}IOP^+$ $[M+H]^+$ 639.1308, found 639.1314.

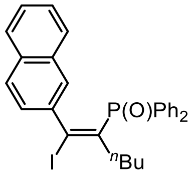

(*E*)-(1-Iodo-1-(naphthalen-2-yl)hex-1-en-2-yl)diphenylphosphine oxide (4n): Yellow solid (m.p.: 122 - 124 °C); 64% yield; **^{1}H NMR** (400 MHz, $CDCl_3$) δ 7.62 – 7.48 (m, 7H), 7.40 – 7.32 (m, 3H), 7.26 – 7.06 (m, 7H), 2.65 – 2.57 (m, 2H), 1.61 – 1.53 (m, 2H), 1.28 – 1.19 (m, 2H), 0.79 (t, *J* = 7.3 Hz, 3H); **^{13}C NMR** (101 MHz, $CDCl_3$) δ 142.7 (d, *J* = 80.2 Hz), 141.1 (d, *J* = 6.4 Hz), 133.0 (d, *J* = 103.5 Hz), 132.6, 131.7, 131.2 - 131.1 (m), 128.6, 128.0 – 127.9 (m), 127.2 (d, *J* = 5.9 Hz), 126.7, 126.18, 126.15, 120.9 (d, *J* = 12.7 Hz), 43.2 (d, *J* = 11.1 Hz), 30.4, 22.7, 13.6; **^{31}P NMR** (162 MHz, $CDCl_3$) δ 22.5. HRMS (ESI) calcd for $C_{28}H_{27}IOP^+$ $[M+H]^+$ 537.0839, found 537.0844.

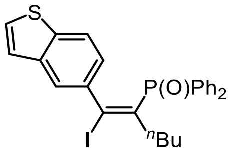

(*E*)-(1-(Benzo[b]thiophen-5-yl)-1-iodohex-1-en-2-yl)diphenylphosphine oxide (4o): Colorless oil; 67% yield; **^{1}H NMR** (400 MHz, $CDCl_3$) δ 7.53 – 7.48 (m, 5H), 7.36 – 7.32 (m, 2H), 7.26 – 7.17 (m, 6H), 7.07 (d, *J* = 5.4 Hz, 1H), 7.01 (dd, *J* = 8.4, 1.4 Hz, 1H), 2.63 – 2.56 (m, 2H), 1.59 – 1.51 (m, 2H), 1.25 – 1.20 (m, 2H), 0.79 (t, *J* = 7.3 Hz, 3H); **^{13}C NMR** (101 MHz, $CDCl_3$) δ 142.7 (d, *J* = 80.3 Hz), 140.2 (d, *J* = 6.3 Hz), 139.7, 138.2, 132.9 (d, *J* = 103.6 Hz), 131.2 (d, *J* = 9.6 Hz), 131.1, 127.9 (d, *J* = 12.1 Hz), 126.8, 124.8, 124.1, 123.8, 121.4, 120.6 (d, *J* = 12.9 Hz), 43.2 (d, *J* = 11.1 Hz), 30.4, 22.7, 13.6; **^{31}P NMR** (162 MHz, $CDCl_3$) δ 22.4. HRMS (ESI) calcd for $C_{26}H_{25}IOPS^+$ $[M+H]^+$ 543.0403, found 543.0406.

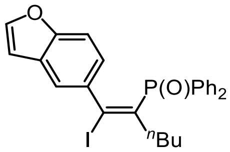

(*E*)-(1-(Benzofuran-5-yl)-1-iodohex-1-en-2-yl)diphenylphosphine oxide (4p): Colorless oil; 67% yield; **^{1}H NMR** (400 MHz, $CDCl_3$) δ 7.54 – 7.50 (m, 5H), 7.36 (s, 1H), 7.30 – 7.19 (m, 6H) (overlap one peak of 7.26), 7.01 – 6.96 (m, 2H), 6.54 (d, *J* = 2.1 Hz, 1H), 2.61 – 2.53 (m, 2H), 1.56 – 1.48 (m, 2H), 1.25 – 1.16 (m, 2H), 0.77 (t, *J* = 7.3 Hz, 3H); **^{13}C NMR** (101 MHz, $CDCl_3$) δ 154.2, 145.4, 142.5 (d, *J* = 80.3 Hz), 139.1 (d, *J* = 6.5 Hz), 133.0 (d, *J* = 103.5 Hz), 131.3 – 131.1 (m), 127.9 (d, *J* = 12.2 Hz), 126.1, 125.4, 122.0, 121.0 (d, *J* = 12.9 Hz), 110.3, 106.8, 43.3 (d, *J* = 11.3 Hz), 30.3, 22.7, 13.6; **^{31}P NMR** (162 MHz, $CDCl_3$) δ 22.2. HRMS (ESI) calcd for $C_{26}H_{25}IO_2P^+$ $[M+H]^+$ 527.0631, found 527.0637.

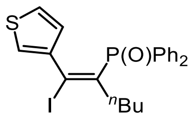

(*E*)-(1-Iodo-1-(thiophen-3-yl)hex-1-en-2-yl)diphenylphosphine oxide (4q): White solid (m.p.: 113 - 115 °C); 52% yield; **^{1}H NMR** (400 MHz, $CDCl_3$) δ 7.62 – 7.57 (m, 4H), 7.52 – 7.51 (m, 1H), 7.39 – 7.29 (m, 6H), 6.77 – 6.75 (m, 1H), 6.65 – 6.64 (m, 1H), 2.50 – 2.41 (m, 2H), 1.47 – 1.40 (m, 2H), 1.17 – 1.07 (m, 2H), 0.71 (t, *J* = 7.3 Hz, 3H); **^{13}C NMR** (101 MHz, $CDCl_3$) δ 143.8 (d, *J* = 7.2 Hz), 143.0 (d, *J* = 80.5 Hz), 132.9 (d, *J* = 104.0 Hz), 131.3 (d, *J* = 2.6 Hz), 131.1 (d, *J* = 9.5 Hz), 128.8, 128.2 (d, *J* = 12.1 Hz), 126.6 (d, *J* = 1.8 Hz), 124.5, 114.8 (d, *J* = 12.0 Hz), 42.9 (d, *J* = 11.4 Hz), 30.0, 22.5, 13.5; **^{31}P NMR** (162 MHz, $CDCl_3$) δ 23.0. HRMS (ESI) calcd for $C_{22}H_{23}IOPS^+$ $[M+H]^+$ 493.0246, found 493.0242.

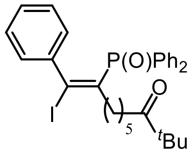

(*E*)-9-(Diphenylphosphoryl)-10-iodo-2,2-dimethyl-10-phenyldec-9-en-3-one (4r): Colorless oil; 51% yield; **^{1}H NMR** (400 MHz, $CDCl_3$) δ 7.56 – 7.51 (m, 4H), 7.38 – 7.35 (m, 2H), 7.31 – 7.26 (m, 4H), 7.06 – 7.04 (m, 2H), 6.94 – 6.87 (m, 3H), 2.57 – 2.49 (m, 2H), 2.38 (t, *J* = 7.3 Hz, 2H), 1.51 – 1.36 (m, 4H), 1.18 – 1.05 (m, 11H); **^{13}C NMR** (101 MHz, $CDCl_3$) δ 215.9, 144.0 (d, *J* = 6.4 Hz), 142.0 (d, *J* = 79.7 Hz), 133.0 (d, *J* = 103.6 Hz), 131.4 – 131.3 (m), 128.6, 128.23, 128.21 (d, *J* = 12.1 Hz), 127.3, 120.9 (d, *J* = 12.7 Hz), 44.1, 43.3 (d, *J* = 11.2 Hz), 36.2, 29.0, 27.8, 26.4, 23.3; **^{31}P NMR** (162 MHz, $CDCl_3$) δ 22.3. HRMS (ESI) calcd for $C_{30}H_{35}IO_2P^+$ $[M+H]^+$ 585.1414, found 585.1413.

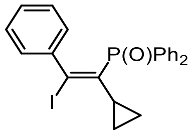

(*E*)-(1-Cyclopropyl-2-iodo-2-phenylvinyl)diphenylphosphine oxide (4s): Yellow solid (m.p.: 135 - 137 °C); 52% yield; **^{1}H NMR** (400 MHz, $CDCl_3$) δ 7.63 (dd, *J* = 10.8, 7.9 Hz, 4H), 7.27 – 7.17 (m, 8H), 6.92 – 6.85 (m, 3H), 1.49 – 1.40 (m, 1H), 0.87 – 0.83 (m, 2H), 0.73 – 0.68 (m, 2H); **^{13}C NMR** (101 MHz, $CDCl_3$) δ 143.5 (d, *J* = 5.6 Hz), 142.6 (d, *J* = 86.6 Hz), 134.1 (d, *J* = 104.3 Hz), 130.8 (d, *J* = 9.1 Hz), 130.6, 129.7, 128.9, 128.3 (d, *J* = 15.7 Hz), 127.9 (d, *J* = 11.9 Hz), 127.4, 25.9 (d, *J* = 12.2 Hz), 11.5 (d, *J* = 4.2 Hz); **^{31}P NMR** (162 MHz, $CDCl_3$) δ 20.9. HRMS (ESI) calcd for $C_{23}H_{21}IOP^+$ $[M+H]^+$ 471.0369, found 471.0369.

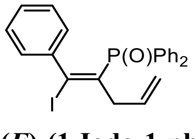

(*E*)-(1-Iodo-1-phenylpenta-1,4-dien-2-yl)diphenylphosphine oxide (4t): Yellow solid (m.p.: 106 - 108 °C); 75% yield; **^{1}H NMR** (400 MHz, $CDCl_3$) δ 7.55 (dd, *J* = 11.6, 7.7 Hz, 4H), 7.37 (t, *J* = 7.3 Hz, 2H), 7.31 – 7.26 (m, 4H), 7.09 – 7.07 (m, 2H), 6.96 – 6.89 (m, 3H), 5.76 – 5.66 (m, 1H), 5.09 – 4.97 (m, 2H), 3.36 (dd, *J* = 15.0, 6.0 Hz, 2H); **^{13}C NMR** (101 MHz, $CDCl_3$) δ 144.0 (d, *J* = 6.2 Hz), 139.9 (d, *J* = 80.9 Hz), 132.8 (d, *J* = 104.1 Hz), 132.3, 131.5 (d, *J* = 9.6 Hz), 131.4 (d, *J* = 1.9 Hz), 128.6, 128.3, 128.1 (d, *J* = 12.2 Hz), 127.3, 122.8 (d, *J* = 11.8 Hz), 117.9, 46.8 (d, *J* = 11.6 Hz); **^{31}P NMR** (162 MHz, $CDCl_3$) δ 22.4. HRMS (ESI) calcd for $C_{23}H_{21}IOP^+$ $[M+H]^+$ 471.0369, found

471.0367.

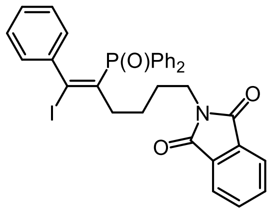

(*E*)-2-(5-(Diphenylphosphoryl)-6-iodo-6-phenylhex-5-en-1-yl)isoindoline-1,3-dione (4u): White solid (m.p.: 140 - 142 °C); 68% yield; **^{1}H NMR** (400 MHz, $CDCl_3$) δ 7.87 – 7.83 (m, 2H), 7.74 – 7.70 (m, 2H), 7.56 – 7.51 (m, 4H), 7.34 – 7.24 (m, 6H), 7.09 – 7.07 (m, 2H), 6.97 – 6.90 (m, 3H), 3.56 – 3.53 (m, 2H), 2.62 – 2.55 (m, 2H), 1.54 – 1.47 (m, 4H); **^{13}C NMR** (101 MHz, $CDCl_3$) δ 168.2, 144.0 (d, *J* = 6.3 Hz), 141.4 (d, *J* = 80.0 Hz), 133.9, 132.8 (d, *J* = 103.7 Hz), 132.2, 131.4 – 131.3 (m), 128.5 – 128.2 (m), 127.3, 123.2, 121.5 (d, *J* = 12.3 Hz), 43.0 (d, *J* = 11.4 Hz), 37.6, 28.4, 25.4; **^{31}P NMR** (162 MHz, $CDCl_3$) δ 22.4. HRMS (ESI) calcd for $C_{32}H_{28}INO_3P^+$ $[M+H]^+$ 632.0846, found 632.0838.

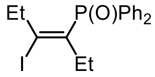

(*E*)-(4-Iodohex-3-en-3-yl)diphenylphosphine oxide (4v): White solid (m.p.: 95 - 96 °C); 15% yield; **^{1}H NMR** (400 MHz, $CDCl_3$) δ 7.70 – 7.65 (m, 4H), 7.57 – 7.53 (m, 2H), 7.49 – 7.45 (m, 4H), 3.07 – 3.01 (m, 2H), 2.33 – 2.23 (m, 2H), 0.92 (t, *J* = 7.2 Hz, 3H), 0.70 (t, *J* = 7.4 Hz, 3H); **^{13}C NMR** (101 MHz, $CDCl_3$) δ 137.7 (d, *J* = 79.9 Hz), 134.0 (d, *J* = 13.3 Hz), 133.1 (d, *J* = 102.8 Hz), 132.0 (d, *J* = 2.6 Hz), 131.7 (d, *J* = 10.0 Hz), 128.6 (d, *J* = 12.3 Hz), 39.0 (d, *J* = 6.9 Hz), 36.2 (d, *J* = 12.9 Hz), 14.8, 12.1; **^{31}P NMR** (162 MHz, $CDCl_3$) δ 26.8. HRMS (ESI) calcd for $C_{18}H_{21}IOP^+$ $[M+H]^+$ 411.0369, found 411.0378.

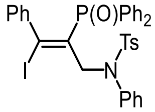

(*E*)-*N*-(2-(Diphenylphosphoryl)-3-iodo-3-phenylallyl)-4-methyl-*N*-phenylbenzenesulfonamide (6): Colorless oil; 27% yield; **^{1}H NMR** (400 MHz, $CDCl_3$) δ 7.51 – 7.46 (m, 4H), 7.35 – 7.16 (m, 15H), 6.83 (t, *J* = 7.4 Hz, 1H), 6.74 (t, *J* = 7.8 Hz, 2H), 6.62 (d, *J* = 7.3 Hz, 2H), 4.83 (d, *J* = 12.4 Hz, 2H), 2.39 (s, 3H); **^{13}C NMR** (101 MHz, $CDCl_3$) δ 143.8 (d, *J* = 6.6 Hz), 143.5, 137.8, 136.7 (d, *J* = 83.5 Hz), 133.9, 133.0 (d, *J* = 105.5 Hz), 131.22 (d, *J* = 9.5 Hz), 131.17 (overlap one peak of 131.22), 130.3, 129.2, 128.8, 128.7, 128.3 – 128.1 (m), 127.3 (d, *J* = 10.3 Hz), 127.1, 59.6 (d, *J* = 11.0 Hz), 21.6; **^{31}P NMR** (162 MHz, $CDCl_3$) δ 20.0. HRMS (ESI) calcd for $C_{34}H_{30}INO_3PS^+$ $[M+H]^+$ 690.0723, found 690.0725.

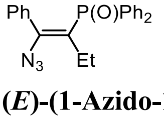

(*E*)-(1-Azido-1-phenylbut-1-en-2-yl)diphenylphosphine oxide (8): Colorless oil; 90% yield; **^{1}H NMR** (400 MHz, $CDCl_3$) δ 8.18 – 8.13 (m, 2H), 7.75 – 7.73 (m, 2H), 7.60 – 7.50 (m, 6H), 7.44 (t, *J* = 7.6 Hz, 2H), 7.28 – 7.24 (m, 1H) (overlap one peak of 7.26), 7.18 (t, *J* = 7.1 Hz, 2H), 2.45 – 2.34 (m, 1H), 2.05 – 1.94 (m, 1H), 0.72 (t, *J* = 7.5 Hz, 3H); **^{13}C NMR** (101 MHz, $CDCl_3$) δ 167.6, 133.6, 132.1 – 131.5 (m), 130.5 (d, *J* = 105.4 Hz), 129.8, 129.1, 128.7 (d, *J* = 11.5 Hz), 128.0 (d, *J* = 11.4 Hz), 124.2, 23.2 (d, *J* = 17.4 Hz), 10.6 (d, *J* = 4.0 Hz); **^{31}P NMR** (162 MHz, $CDCl_3$) δ 34.2. HRMS (ESI) calcd for $C_{22}H_{21}NOP^+$ $[M+H-N_2]^+$ 346.1355, found 346.1350.

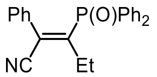

(*E*)-3-(Diphenylphosphoryl)-2-phenylpent-2-enenitrile (9): White solid (m.p.: 111 - 113 °C); 99% yield; **^{1}H NMR** (400 MHz, $CDCl_3$) δ 7.59 – 7.54 (m, 4H), 7.39 – 7.26 (m, 8H), 7.07 – 6.98 (m, 3H), 2.74 – 2.64 (m, 2H), 1.12 (t, *J* = 7.5 Hz, 3H); **^{13}C NMR** (101 MHz, $CDCl_3$) δ 154.7 (d, *J* = 85.6 Hz), 132.8 (d, *J* = 5.3 Hz), 131.9 (d, *J* = 2.8 Hz), 131.5 (d, *J* = 105.4 Hz), 131.2 (d, *J* = 9.6 Hz), 129.6, 129.5, 128.5 (d, *J* = 12.3 Hz), 128.0, 127.2 (d, *J* = 15.5 Hz), 117.0 (d, *J* = 22.0 Hz), 29.6 (d, *J* = 9.4 Hz), 14.3 (d, *J* = 1.0 Hz); **^{31}P NMR** (162 MHz, $CDCl_3$) δ 26.2. HRMS (ESI) calcd for $C_{23}H_{21}NOP^+$ $[M+H]^+$ 358.1355, found 358.1352.

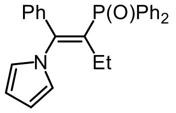

(*E*)-Diphenyl(1-phenyl-1-(1H-pyrrol-1-yl)but-1-en-2-yl)phosphine oxide (10): White solid (m.p.: 204 - 206 °C); 90% yield; **^{1}H NMR** (400 MHz, $CDCl_3$) δ 7.73 – 7.68 (m, 4H), 7.34 – 7.21 (m, 8H), 7.08 – 7.04 (m, 1H), 6.98 (t, *J* = 7.7 Hz, 2H), 6.67 (t, *J* = 2.2 Hz, 2H), 6.27 (t, *J* = 2.2 Hz, 2H), 2.53 – 2.44 (m, 2H), 0.88 (t, *J* = 7.4 Hz, 3H); **^{13}C NMR** (101 MHz, $CDCl_3$) δ 151.9 (d, *J* = 18.3 Hz), 137.5 (d, *J* = 4.8 Hz), 134.0 (d, *J* = 104.2 Hz), 131.0 (d, *J* = 9.0 Hz), 130.9, 130.8 (d, *J* = 2.7 Hz), 129.8, 128.1 (d, *J* = 12.0 Hz), 128.0 (d, *J* = 97.7 Hz), 127.7, 122.4, 109.8, 25.9 (d, *J* = 9.5 Hz), 14.3 (d, *J* = 3.1 Hz); **^{31}P NMR** (162 MHz, $CDCl_3$) δ 26.2. HRMS (ESI) calcd for $C_{26}H_{25}NOP^+$ $[M+H]^+$ 398.1668, found 398.1666.

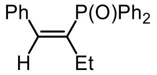

(*Z*)-Diphenyl(1-phenylbut-1-en-2-yl)phosphine oxide (11-1): White solid (m.p.: 105 - 107 °C); 61% yield; **^{1}H NMR** (400 MHz, $CDCl_3$) δ 7.67 – 7.62 (m, 4H), 7.45 – 7.23 (m, 9H), 6.97 – 6.95 (m, 3H), 2.31 – 2.23 (m, 2H), 1.12 – 1.06 (m, 3H); **^{13}C NMR** (101 MHz, $CDCl_3$) δ 144.3 (d, *J* = 7.3 Hz), 135.9 (d, *J* = 6.7 Hz), 135.7 (d, *J* = 91.2 Hz), 133.2 (d, *J* = 102.3 Hz), 131.4 (d, *J* = 9.6 Hz), 131.1 (d, *J* = 2.8 Hz), 129.4 (d, *J* = 1.3 Hz), 128.1 (d, *J* = 12.0 Hz), 127.7, 127.5, 29.8 (d, *J* = 12.6 Hz), 14.3 (d, *J* = 4.8 Hz); **^{31}P NMR** (162 MHz, $CDCl_3$) δ 27.2. HRMS (ESI) calcd for $C_{22}H_{22}OP^+$ $[M+H]^+$ 333.1403, found 333.1409.

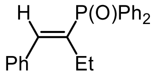

(*E*)-Diphenyl(1-phenylbut-1-en-2-yl)phosphine oxide (11-2): Colorless oil; 30% yield; **^{1}H NMR** (400 MHz, $CDCl_3$) δ 7.79 – 7.74 (m, 4H), 7.54 (td, *J* = 7.4, 1.1 Hz, 2H), 7.50 – 7.46 (m, 4H), 7.37 – 7.28 (m, 5H), 7.06 (d, *J* = 22.7 Hz, 1H), 2.67 – 2.57 (m, 2H), 0.97 (t, *J* = 7.4 Hz, 3H); **^{13}C NMR** (101 MHz, $CDCl_3$) δ 142.7 (d, *J* = 12.1 Hz), 137.0 (d, *J* = 94.8 Hz), 135.7 (d, *J* = 19.6 Hz), 132.2 (d, *J* = 9.6 Hz), 132.0 (d, *J* = 101.8 Hz), 131.9 (d, *J* = 2.7 Hz), 129.1 (d, *J* = 0.9 Hz), 128.59, 128.57, 128.5, 128.4, 21.9 (d, *J* = 10.3 Hz), 14.0 (d, *J* = 1.8 Hz); **^{31}P NMR** (162 MHz, $CDCl_3$) δ 33.8. HRMS (ESI) calcd for $C_{22}H_{22}OP^+$ $[M+H]^+$ 333.1403, found 333.1406.

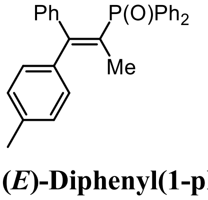

(*E*)-Diphenyl(1-phenyl-1-(*p*-tolyl)prop-1-en-2-yl)phosphine oxide (12): White solid (m.p.: 256 - 258 °C); 93% yield; **^{1}H NMR** (400 MHz, $CDCl_3$) δ 7.71 – 7.66 (m, 4H), 7.32 – 7.24 (m, 6H), 7.13

– 7.10 (m, 4H), 7.06 – 7.04 (m, 2H), 6.93 – 6.87 (m, 3H), 2.32 (s, 3H), 1.90 (d, *J* = 13.4 Hz, 3H); **^{13}C NMR** (101 MHz, $CDCl_3$) δ 157.9 (d, *J* = 8.8 Hz), 141.1 (d, *J* = 7.4 Hz), 139.6 (d, *J* = 15.4 Hz), 137.7, 134.1 (d, *J* = 102.4 Hz), 131.2 (d, *J* = 9.3 Hz), 130.7 (d, *J* = 2.7 Hz), 130.4, 129.1, 128.9, 128.1 (d, *J* = 12.0 Hz), 127.7, 127.4, 126.8 (d, *J* = 96.7 Hz), 21.6 (d, *J* = 12.6 Hz), 21.2; **^{31}P NMR** (162 MHz, $CDCl_3$) δ 27.2. HRMS (ESI) calcd for $C_{28}H_{26}OP^+$ $[M+H]^+$ 409.1716, found 409.1720.

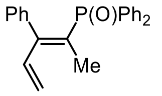

(*Z*)-Diphenyl(3-phenylpenta-2,4-dien-2-yl)phosphine oxide (13): Colorless oil; 95% yield; **^{1}H NMR** (400 MHz, $CDCl_3$) δ 7.57 – 7.52 (m, 4H), 7.33 (td, *J* = 7.2, 1.1 Hz, 2H), 7.28 – 7.23 (m, 4H), 7.06 (ddd, *J* = 17.0, 10.6, 1.8 Hz, 1H), 6.99 – 6.89 (m, 5H), 5.40 (dd, *J* = 10.7, 2.5 Hz, 1H), 4.81 (d, *J* = 17.0 Hz, 1H), 2.10 (d, *J* = 13.2 Hz, 3H); **^{13}C NMR** (101 MHz, $CDCl_3$) δ 152.6 (d, *J* = 8.7 Hz), 137.1 (d, *J* = 7.4 Hz), 136.0 (d, *J* = 15.3 Hz), 133.9 (d, *J* = 103.0 Hz), 131.3 (d, *J* = 9.5 Hz), 130.8 (d, *J* = 2.8 Hz), 130.6, 128.1 (d, *J* = 11.9 Hz), 127.7 (d, *J* = 99.6 Hz), 127.3, 127.2, 123.2, 17.9 (d, *J* = 12.3 Hz); **^{31}P NMR** (162 MHz, $CDCl_3$) δ 29.0. HRMS (ESI) calcd for $C_{23}H_{22}OP^+$ $[M+H]^+$ 345.1403, found 345.1404.

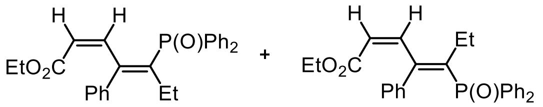

Z,E / *Z,Z* = 5 : 4

Ethyl (2*Z*,4*E*)-5-(Diphenylphosphoryl)-4-phenylhepta-2,4-dienoate (14): Colorless oil; 88% yield; **^{1}H NMR** (400 MHz, $CDCl_3$, two isomers) δ 8.40 (dd, *J* = 15.4, 1.4 Hz, 1H, major), 7.99 (dd, *J* = 15.4, 1.6 Hz, 0.8H, minor), 7.78 (dd, *J* = 11.7, 7.6 Hz, 4H, major), 7.54 – 7.25 (m, 9H + 8H, major + minor), 7.13 (d, *J* = 7.8 Hz, 2H, major), 6.99 (t, *J* = 7.4 Hz, 0.8H, minor), 6.92 (t, *J* = 7.4 Hz, 1.6H, minor), 6.84 (t, *J* = 7.6 Hz, 1.6H, minor), 5.38 (d, *J* = 15.4 Hz, 0.8H, minor), 5.21 (d, *J* = 15.4 Hz, 1H, major), 4.14 (q, *J* = 7.1 Hz, 1.6H, minor), 4.00 (q, *J* = 7.1 Hz, 1H, major), 2.70 – 2.61 (m, 1.7H, minor), 2.15 – 2.05 (m, 2H, major), 1.21 (td, *J* = 7.2, 1.2 Hz, 2.4H, minor), 1.13 – 1.07 (m, 3H + 2.4H, major + minor), 0.56 (t, *J* = 7.4 Hz, 3H, major); **^{13}C NMR** (101 MHz, $CDCl_3$, two isomers) δ 166.7 (d, *J* = 1.8 Hz, minor), 166.3 (major), 152.2 (d, *J* = 6.5 Hz, major), 149.7 (d, *J* = 9.7 Hz, minor), 143.2 (d, *J* = 10.0 Hz, major), 142.7 (d, *J* = 93.1 Hz, minor), 142.2 (d, *J* = 15.7 Hz, minor), 142.0 (d, *J* = 86.2 Hz, major), 137.6 (d, *J* = 13.8 Hz, major), 136.1 (d, *J* = 7.2 Hz, minor), 133.3 (d, *J* = 102.4 Hz, major), 133.2 (d, *J* = 103.4 Hz, minor), 132.0 (d, *J* = 2.5 Hz, major), 131.8 (d, *J* = 10.0 Hz, major), 131.4 (d, *J* = 9.6 Hz, minor), 131.2 (d, *J* = 2.4 Hz, minor), 130.3 (minor), 128.7 (d, *J* = 11.8 Hz, major), 128.6 (major), 128.4 (major), 128.2 (d, *J* = 11.9 Hz, minor), 127.8 (major), 127.6 (minor), 127.4 (minor), 126.5 (minor), 125.4 (major), 60.7 (minor), 60.3 (major), 26.3 (d, *J* = 11.2 Hz, major), 24.4 (d, *J* = 11.0 Hz, minor), 15.7 (minor), 14.2 (minor), 14.1 (major), 14.0 (d, *J* = 1.1 Hz, major); **^{31}P NMR** (162 MHz, $CDCl_3$, two isomers) δ 29.6 (major), 29.0 (minor). HRMS (ESI) calcd for $C_{27}H_{28}O_3P^+$ $[M+H]^+$ 431.1771, found 431.1768.

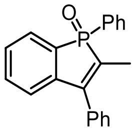

2-Methyl-1,3-diphenylphosphindole 1-oxide (15a): White solid; 95% yield; Spectroscopic data in agreement with that reported previously.[18]

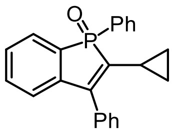

2-Cyclopropyl-1,3-diphenylphosphindole 1-oxide (15b): White solid (m.p.: 82 - 84 °C); 83% yield; **^{1}H NMR** (400 MHz, $CDCl_3$) δ 7.79 – 7.73 (m, 2H), 7.53 – 7.49 (m, 4H), 7.46 – 7.41 (m, 5H), 7.35 (t, *J* = 7.6 Hz, 1H), 7.22 (td, *J* = 7.3, 3.9 Hz, 1H), 7.03 (dd, *J* = 7.6, 2.6 Hz, 1H), 1.70 – 1.58 (m, 1H), 1.28 – 1.22 (m, 1H), 0.79 – 0.71 (m, 1H), 0.57 – 0.52 (m, 2H); **^{13}C NMR** (101 MHz, $CDCl_3$) δ 150.0 (d, *J* = 22.2 Hz), 143.6 (d, *J* = 26.9 Hz), 137.3 (d, *J* = 97.0 Hz), 134.1 (d, *J* = 15.3 Hz), 132.8 (d, *J* = 2.0 Hz), 132.1 (d, *J* = 2.9 Hz), 131.8 (d, *J* = 106.9 Hz), 130.8 (d, *J* = 10.7 Hz), 130.5 (d, *J* = 98.1 Hz), 129.0 - 128.9 (m), 128.7, 128.6 (d, *J* = 10.0 Hz), 128.5, 128.3 (d, *J* = 10.8 Hz), 122.6 (d, *J* = 10.9 Hz), 11.0 (d, *J* = 9.3 Hz), 7.4 (d, *J* = 3.0 Hz), 7.0 (d, *J* = 2.2 Hz); **^{31}P NMR** (162 MHz, $CDCl_3$) δ 37.6. HRMS (ESI) calcd for $C_{23}H_{20}OP^+$ $[M+H]^+$ 343.1246, found 343.1246.

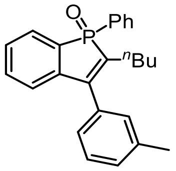

2-Butyl-1-phenyl-3-(*m*-tolyl)phosphindole 1-oxide (15c): Colorless oil; 77% yield; **^{1}H NMR** (400 MHz, $CDCl_3$) δ 7.76 – 7.71 (m, 2H), 7.60 – 7.55 (m, 1H), 7.52 – 7.48 (m, 1H), 7.44 – 7.39 (m, 2H), 7.36 (t, *J* = 7.6 Hz, 2H), 7.27 – 7.21 (m, 2H), 7.09 – 7.08 (m, 2H), 7.00 (dd, *J* = 7.6, 2.8 Hz, 1H), 2.47 – 2.36 (m, 4H), 2.24 – 2.12 (m, 1H), 1.38 – 1.29 (m, 2H), 1.13 – 1.06 (m, 2H), 0.63 (t, *J* = 7.3 Hz, 3H); **^{13}C NMR** (101 MHz, $CDCl_3$) δ 149.6 (d, *J* = 22.2 Hz), 143.3 (d, *J* = 28.1 Hz), 137.4, 135.5 (d, *J* = 94.1 Hz), 132.8 (d, *J* = 15.9 Hz), 131.8 (d, *J* = 1.9 Hz), 131.0 (d, *J* = 2.8 Hz), 130.8 (d, *J* = 105.4 Hz), 129.9 (d, *J* = 10.7 Hz), 129.1 (d, *J* = 97.8 Hz), 128.2, 127.9, 127.8 (d, *J* = 12.2 Hz), 127.7 (d, *J* = 9.6 Hz), 127.6, 127.4 (d, *J* = 10.5 Hz), 124.5, 122.2 (d, *J* = 11.0 Hz), 29.8 (d, *J* = 1.7 Hz), 25.4 (d, *J* = 10.2 Hz), 21.6, 20.5, 12.5; **^{31}P NMR** (162 MHz, $CDCl_3$) δ 40.0. HRMS (ESI) calcd for $C_{25}H_{26}OP^+$ $[M+H]^+$ 373.1716, found 373.1721.

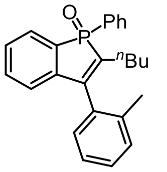

2-Butyl-1-phenyl-3-(*o*-tolyl)phosphindole 1-oxide (15d): Colorless oil; 80% yield; There are two diastereomers in this compound. **^{1}H NMR** (400 MHz, $CDCl_3$) δ 7.80 – 7.75 (m, 4H), 7.64 – 7.59 (m, 2H), 7.55 – 7.51 (m, 2H), 7.46 – 7.43 (m, 4H), 7.38 – 7.25 (m, 10H), 7.17 (d, *J* = 6.8 Hz, 1H), 7.09 (d, *J* = 7.4 Hz, 1H), 6.77 – 6.75 (m, 2H), 2.43 – 2.03 (m, 10H), 1.38 – 1.26 (m, 4H), 1.16 – 1.00 (m, 4H), 0.66 – 0.59 (m, 6H); **^{13}C NMR** (101 MHz, $CDCl_3$) δ 150.7 (d, *J* = 22.5 Hz), 150.5 (d, *J* = 22.2 Hz), 144.3 (d, *J* = 28.1 Hz), 143.9 (d, *J* = 28.2 Hz), 137.5 (d, *J* = 93.4 Hz), 137.3 (d, *J* = 93.5 Hz), 136.4, 135.5, 133.8 (d, *J* = 15.3 Hz), 133.6 (d, *J* = 15.4 Hz), 133.0 (d, *J* = 2.0 Hz), 132.9 (d, *J* = 2.0 Hz), 132.1 (d, *J* = 2.9 Hz), 132.0 (d, *J* = 2.9 Hz), 131.7 (d, *J* = 105.1 Hz), 131.0 (d, *J* = 10.5 Hz), 130.9 (d, *J* = 10.7 Hz) (overlap one peak of 131.0), 130.6, 130.4 (d, *J* = 97.4 Hz), 130.3, 130.2 (d, *J* = 97.7 Hz), 128.9 – 128.5 (m), 126.3, 126.0, 123.0 (d, *J* = 10.8 Hz), 122.9 (d, *J* = 10.9 Hz), 30.3 (d, *J* = 1.8 Hz), 26.6 (d, *J* = 10.4 Hz), 22.7, 22.6, 19.8, 19.7, 13.47, 13.46; **^{31}P NMR** (162

MHz, $CDCl_3$) δ 40.0, 39.2. HRMS (ESI) calcd for $C_{25}H_{26}OP^+$ $[M+H]^+$ 373.1716, found 373.1712.

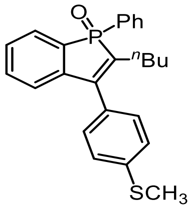

2-Butyl-3-(4-(methylthio)phenyl)-1-phenylphosphindole 1-oxide (15e): Colorless oil; 70% yield; **^{1}H NMR** (400 MHz, $CDCl_3$) δ 7.75 (dd, *J* = 12.4, 8.0 Hz, 2H), 7.63 – 7.58 (m, 1H), 7.54 – 7.51 (m, 1H), 7.46 – 7.36 (m, 5H), 7.31 – 7.24 (m, 3H), 7.05 – 7.03 (m, 1H), 2.55 (s, 3H), 2.49 – 2.39 (m, 1H), 2.27 – 2.17 (m, 1H), 1.40 – 1.30 (m, 2H), 1.18 – 1.05 (m, 2H), 0.66 (t, *J* = 7.2 Hz, 3H); **^{13}C NMR** (101 MHz, $CDCl_3$) δ 149.8 (d, *J* = 22.4 Hz), 144.2 (d, *J* = 27.9 Hz), 139.3, 136.9 (d, *J* = 94.0 Hz), 132.8 (d, *J* = 1.8 Hz), 132.1 (d, *J* = 2.8 Hz), 131.9 (d, *J* = 105.2 Hz), 131.0 (d, *J* = 10.6 Hz), 130.3 (d, *J* = 16.2 Hz), 130.1 (d, *J* = 97.9 Hz), 129.0, 128.86 (d, *J* = 9.5 Hz), 128.82 (d, *J* = 12.3 Hz), 128.5 (d, *J* = 10.7 Hz), 126.3, 123.1 (d, *J* = 11.0 Hz), 30.9, 26.5 (d, *J* = 10.1 Hz), 22.7, 15.5, 13.5; **^{31}P NMR** (162 MHz, $CDCl_3$) δ 39.8. HRMS (ESI) calcd for $C_{25}H_{26}OPS^+$ $[M+H]^+$ 405.1436, found 405.1434.

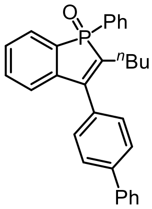

3-([1,1'-Biphenyl]-4-yl)-2-butyl-1-phenylphosphindole 1-oxide (15f): Colorless oil; 71% yield; **^{1}H NMR** (400 MHz, $CDCl_3$) δ 7.69 – 7.64 (m, 2H), 7.60 (d, *J* = 8.3 Hz, 2H), 7.54 – 7.46 (m, 3H), 7.39 – 7.20 (m, 9H), 7.13 (td, *J* = 7.2, 3.8 Hz, 1H), 6.97 (dd, *J* = 7.6, 2.8 Hz, 1H), 2.44 – 2.32 (m, 1H), 2.19 – 2.08 (m, 1H), 1.36 – 1.20 (m, 2H), 1.07 – 0.94 (m, 2H), 0.54 (t, *J* = 7.3 Hz, 3H); **^{13}C NMR** (101 MHz, $CDCl_3$) δ 148.9 (d, *J* = 22.2 Hz), 143.2 (d, *J* = 27.8 Hz), 140.2, 139.3, 136.0 (d, *J* = 93.7 Hz), 131.79 (d, *J* = 1.6 Hz), 131.78 (d, *J* = 16.0 Hz), 131.1 (d, *J* = 2.6 Hz), 130.9 (d, *J* = 105.2 Hz), 129.9 (d, *J* = 10.5 Hz), 129.1 (d, *J* = 97.8 Hz), 128.0, 127.9, 127.77 (d, *J* = 12.5 Hz), 127.76 (d, *J* = 9.7 Hz) (overlap one peak of 127.77), 127.5 (d, *J* = 10.6 Hz), 126.6, 126.3, 126.0, 122.2 (d, *J* = 11.0 Hz), 29.8 (d, *J* = 1.2 Hz), 25.5 (d, *J* = 10.1 Hz), 21.6, 12.5; **^{31}P NMR** (162 MHz, $CDCl_3$) δ 39.7. HRMS (ESI) calcd for $C_{30}H_{28}OP^+$ $[M+H]^+$ 435.1872, found 435.1873.

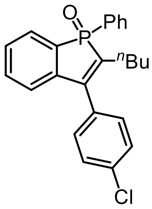

2-Butyl-3-(4-chlorophenyl)-1-phenylphosphindole 1-oxide (15g): White solid (m.p.: 83 - 84 °C); 65% yield; **^{1}H NMR** (400 MHz, $CDCl_3$) δ 7.77 – 7.72 (m, 2H), 7.64 – 7.60 (m, 1H), 7.56 – 7.39 (m, 6H), 7.33 – 7.26 (m, 3H), 6.99 (dd, *J* = 7.6, 2.6 Hz, 1H), 2.48 – 2.37 (m, 1H), 2.25 – 2.13 (m, 1H), 1.43 – 1.28 (m, 2H), 1.21 – 1.04 (m, 2H), 0.66 (t, *J* = 7.4 Hz, 3H); **^{13}C NMR** (101 MHz, $CDCl_3$) δ 149.0 (d, *J* = 22.7 Hz), 144.0 (d, *J* = 27.8 Hz), 137.7 (d, *J* = 93.5 Hz), 134.5, 132.9 (d, *J* = 1.9 Hz),

132.4 (d, J = 16.3 Hz), 132.2 (d, J = 2.8 Hz), 131.8 (d, J = 105.4 Hz), 131.0 (d, J = 10.7 Hz), 130.0, 129.9 (d, J = 98.2 Hz), 129.1, 129.0 (d, J = 11.2 Hz) (overlap one peak of 128.9), 128.9 (d, J = 12.3 Hz), 128.7 (d, J = 10.6 Hz), 123.0 (d, J = 11.0 Hz), 30.8 (d, J = 1.6 Hz), 26.5 (d, J = 10.0 Hz), 22.7, 13.5; **^{31}P NMR** (162 MHz, $CDCl_3$) δ 39.6. HRMS (ESI) calcd for $C_{24}H_{23}ClOP^+$ $[M+H]^+$ 393.1170, found 393.1167.

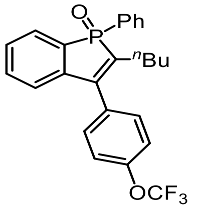

2-Butyl-1-phenyl-3-(4-(trifluoromethoxy)phenyl)phosphindole 1-oxide (15h): Colorless oil; 85% yield; **^{1}H NMR** (400 MHz, $CDCl_3$) δ 7.75 – 7.70 (m, 2H), 7.62 – 7.58 (m, 1H), 7.54 – 7.49 (m, 1H), 7.45 – 7.26 (m, 8H), 6.97 (dd, J = 7.6, 2.9 Hz, 1H), 2.47 – 2.35 (m, 1H), 2.23 – 2.12 (m, 1H), 1.38 – 1.29 (m, 2H), 1.15 – 1.02 (m, 2H), 0.63 (t, J = 7.3 Hz, 3H); **^{13}C NMR** (101 MHz, $CDCl_3$) δ 149.2 (q, J = 1.8 Hz), 148.8 (d, J = 22.7 Hz), 143.9 (d, J = 27.7 Hz), 137.9 (d, J = 93.4 Hz), 132.9 (d, J = 1.6 Hz), 132.6 (d, J = 16.1 Hz), 132.2 (d, J = 2.8 Hz), 131.8 (d, J = 105.3 Hz), 130.9 (d, J = 10.8 Hz), 130.2, 129.9 (d, J = 98.3 Hz), 129.0 (d, J = 9.6 Hz), 128.9 (d, J = 12.3 Hz), 128.7 (d, J = 10.7 Hz), 123.0 (d, J = 11.0 Hz), 121.3, 120.5 (q, J = 258.8 Hz), 30.8 (d, J = 1.5 Hz), 26.5 (d, J = 9.8 Hz), 22.6, 13.4; **^{31}P NMR** (162 MHz, $CDCl_3$) δ 39.6; **^{19}F NMR** (376 MHz, $CDCl_3$) δ -57.8. HRMS (ESI) calcd for $C_{25}H_{23}F_3O_2P^+$ $[M+H]^+$ 443.1382, found 443.1376.

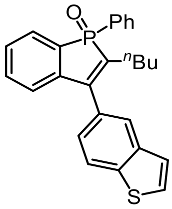

3-(Benzo[*b*]thiophen-5-yl)-2-butyl-1-phenylphosphindole 1-oxide (15i): Colorless oil; 75% yield; **^{1}H NMR** (400 MHz, $CDCl_3$) δ 8.00 (d, J = 8.2 Hz, 1H), 7.82 – 7.77 (m, 3H), 7.65 – 7.61 (m, 1H), 7.55 – 7.51 (m, 2H), 7.48 – 7.43 (m, 2H), 7.40 – 7.36 (m, 2H), 7.31 – 7.26 (m, 2H), 7.03 (dd, J = 7.5, 2.8 Hz, 1H), 2.53 – 2.42 (m, 1H), 2.29 – 2.18 (m, 1H), 1.45 – 1.30 (m, 2H), 1.18 – 1.04 (m, 2H), 0.64 (t, J = 7.3 Hz, 3H); **^{13}C NMR** (101 MHz, $CDCl_3$) δ 150.4 (d, J = 22.5 Hz), 144.5 (d, J = 27.9 Hz), 139.9, 139.8, 137.2 (d, J = 93.8 Hz), 132.8 (d, J = 1.8 Hz), 132.1 (d, J = 2.8 Hz), 131.9 (d, J = 105.2 Hz), 131.0 (d, J = 10.6 Hz), 130.2 (d, J = 97.8 Hz), 130.0 (d, J = 16.1 Hz), 128.85 (d, J = 12.3 Hz), 128.83 (d, J = 8.6 Hz), 128.5 (d, J = 10.5 Hz), 127.5, 124.6, 123.9, 123.5, 123.3 (d, J = 11.0 Hz), 122.9, 30.9 (d, J = 1.6 Hz), 26.6 (d, J = 10.1 Hz), 22.7, 13.5; **^{31}P NMR** (162 MHz, $CDCl_3$) δ 39.8. HRMS (ESI) calcd for $C_{26}H_{24}OPS^+$ $[M+H]^+$ 415.1280, found 415.1286.

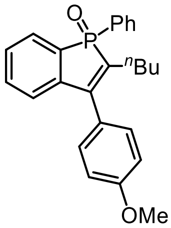

2-Butyl-3-(4-methoxyphenyl)-1-phenylphosphindole 1-oxide (15j): Colorless oil; 20% yield; **^{1}H**

NMR (400 MHz, $CDCl_3$) δ 7.76 – 7.71 (m, 2H), 7.60 – 7.56 (m, 1H), 7.52 – 7.48 (m, 1H), 7.44 – 7.36 (m, 3H), 7.29 – 7.23 (m, 3H), 7.06 – 7.01 (m, 3H), 3.86 (d, *J* = 1.4 Hz, 3H), 2.49 – 2.38 (m, 1H), 2.26 – 2.14 (m, 1H), 1.37 – 1.30 (m, 2H), 1.18 – 1.04 (m, 2H), 0.65 (t, *J* = 7.2 Hz, 3H); **^{13}C NMR** (101 MHz, $CDCl_3$) δ 159.7, 150.2 (d, *J* = 22.4 Hz), 144.5 (d, *J* = 28.0 Hz), 136.4 (d, *J* = 94.3 Hz), 132.8, 132.0 (d, *J* = 2.7 Hz), 131.9 (d, *J* = 105.2 Hz), 131.0 (d, *J* = 10.7 Hz), 130.2 (d, *J* = 97.9 Hz), 129.9, 128.8 (d, *J* = 11.9 Hz), 128.4 (d, *J* = 10.6 Hz), 126.0 (d, *J* = 16.3 Hz), 123.2 (d, *J* = 11.1 Hz), 114.2, 55.3, 30.9 (d, *J* = 1.4 Hz), 26.5 (d, *J* = 10.2 Hz), 22.7, 13.5; **^{31}P NMR** (162 MHz, $CDCl_3$) δ 39.9. HRMS (ESI) calcd for $C_{25}H_{26}O_2P^+$ $[M+H]^+$ 389.1665, found 389.1660.

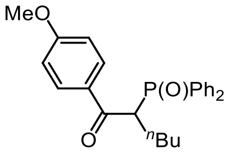

2-(Diphenylphosphoryl)-1-(4-methoxyphenyl)hexan-1-one (16): White solid; 31% yield; **^{1}H NMR** (400 MHz, $CDCl_3$) δ 7.94 – 7.89 (m, 2H), 7.80 (d, *J* = 8.9 Hz, 2H), 7.72 – 7.67 (m, 2H), 7.47 – 7.35 (m, 4H), 7.32 – 7.28 (m, 2H), 6.77 (d, *J* = 8.9 Hz, 2H), 4.43 (ddd, *J* = 16.6, 11.0, 3.0 Hz, 1H), 3.78 (s, 3H), 2.27 – 2.16 (m, 1H), 1.96 – 1.85 (m, 1H), 1.27 – 1.15 (m, 4H), 0.72 (t, *J* = 7.1 Hz, 3H); **^{13}C NMR** (101 MHz, $CDCl_3$) δ 196.3 (d, *J* = 2.4 Hz), 163.5, 132.0 – 131.8 (m), 131.49 (d, *J* = 99.5 Hz), 131.46, 131.4 (overlap one peak of 130.9), 131.1 (d, *J* = 1.2 Hz), 131.0 (overlap one peak of 131.49), 130.9 (d, *J* = 99.9 Hz), 128.4 (d, *J* = 12.1 Hz), 128.3 (d, *J* = 11.9 Hz), 113.6, 55.4, 52.0 (d, *J* = 57.8 Hz), 31.2 (d, *J* = 12.4 Hz), 28.3 (d, *J* = 2.3 Hz), 22.4, 13.7; **^{31}P NMR** (162 MHz, $CDCl_3$) δ 29.7. HRMS (ESI) calcd for $C_{25}H_{28}O_3P^+$ $[M+H]^+$ 407.1771, found 407.1769.

13. NMR spectra

^{1}H NMR (400 MHz, $CDCl_3$) of 3a

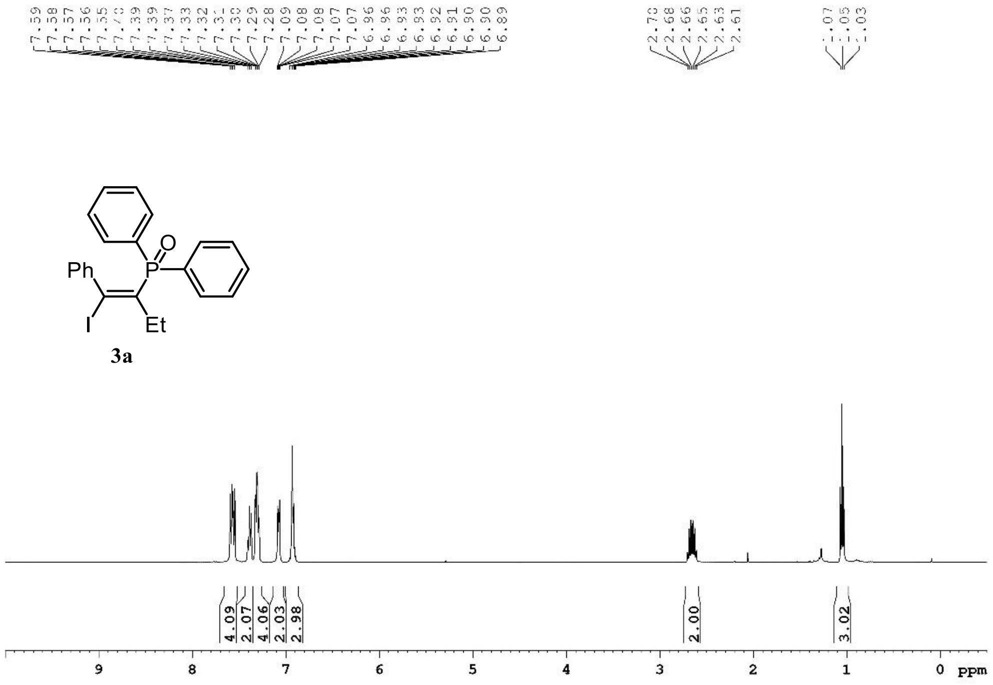

^{13}C NMR (101 MHz, $CDCl_3$) of 3a

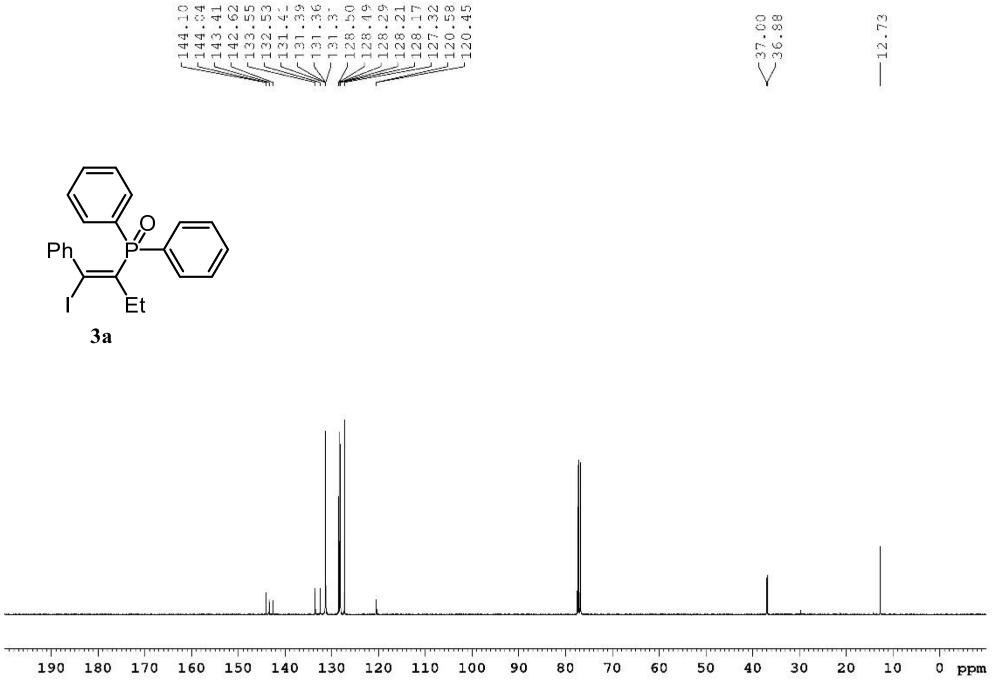

^{31}P NMR (162 MHz, $CDCl_3$) of 3a

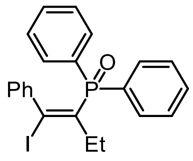
3a

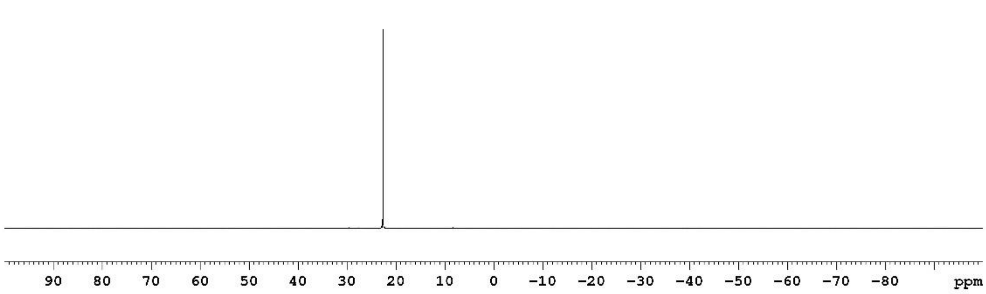

^{1}H NMR (400 MHz, $CDCl_3$) of 3b

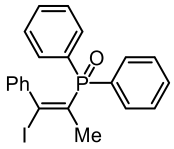
3b

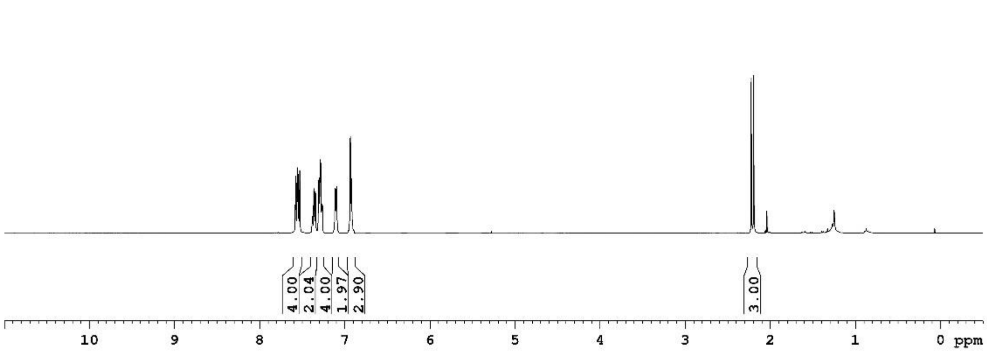

^{13}C NMR (101 MHz, CDCl$_3$) of 3b

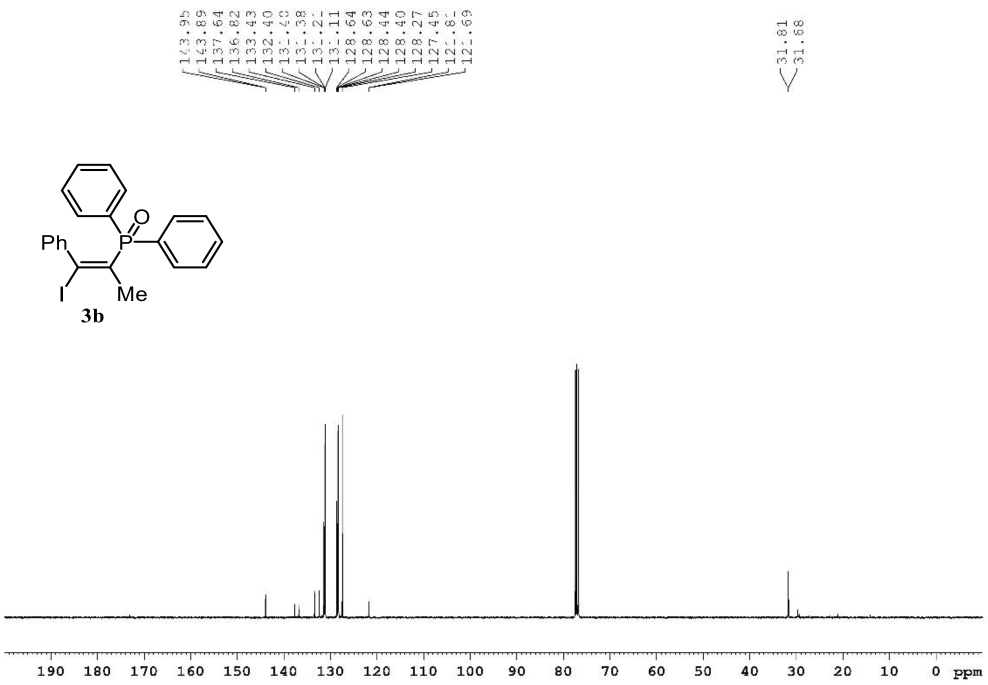

^{31}P NMR (162 MHz, CDCl$_3$) of 3b

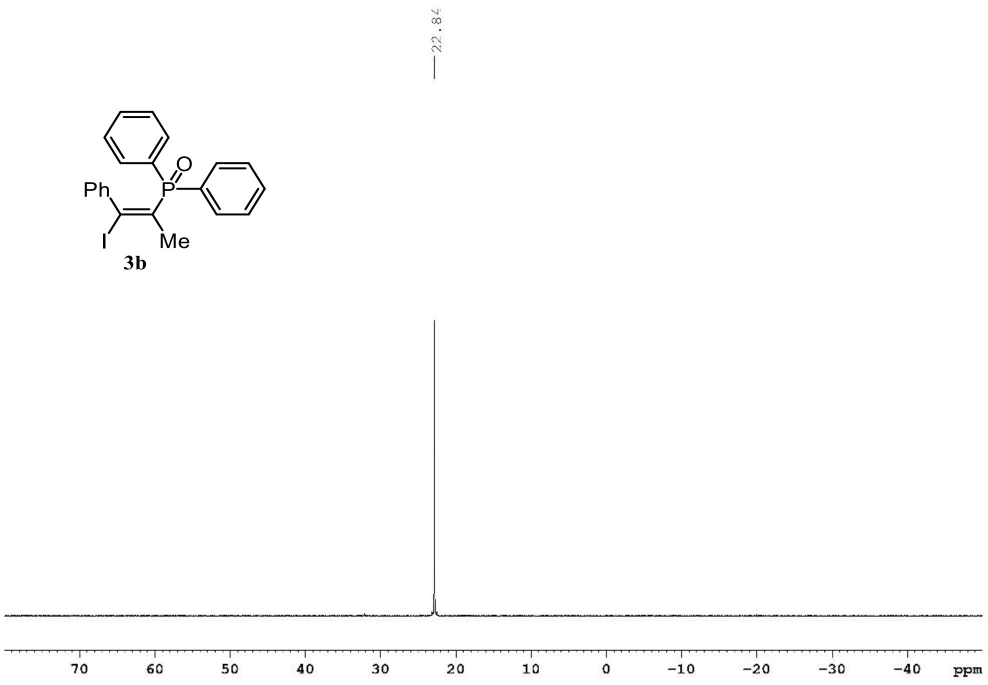

^{1}H NMR (400 MHz, $CDCl_3$) of 3c

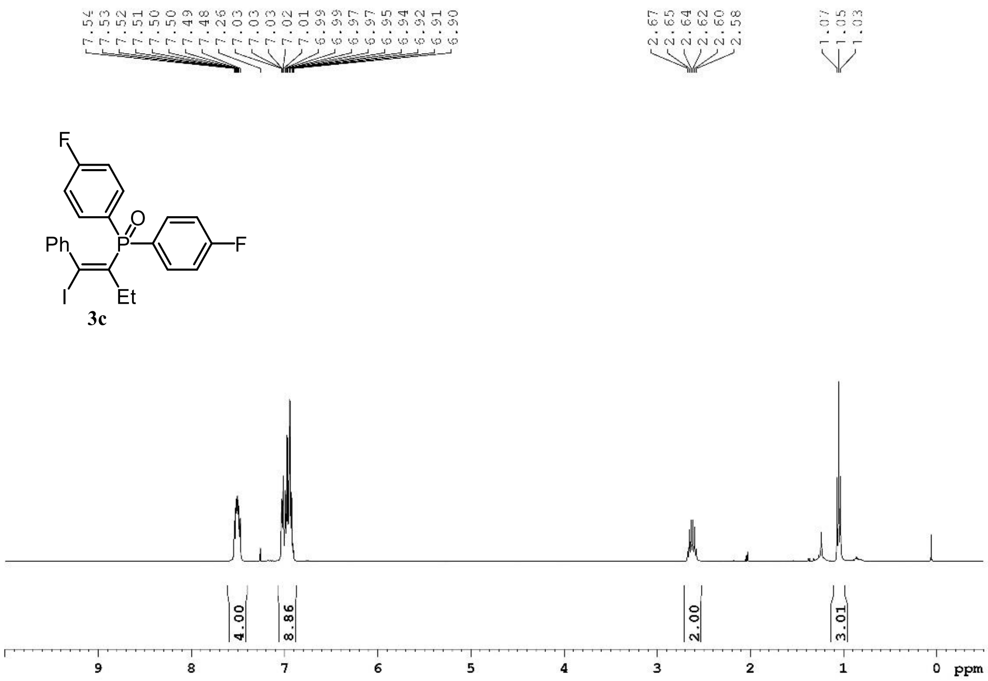

^{13}C NMR (101 MHz, $CDCl_3$) of 3c

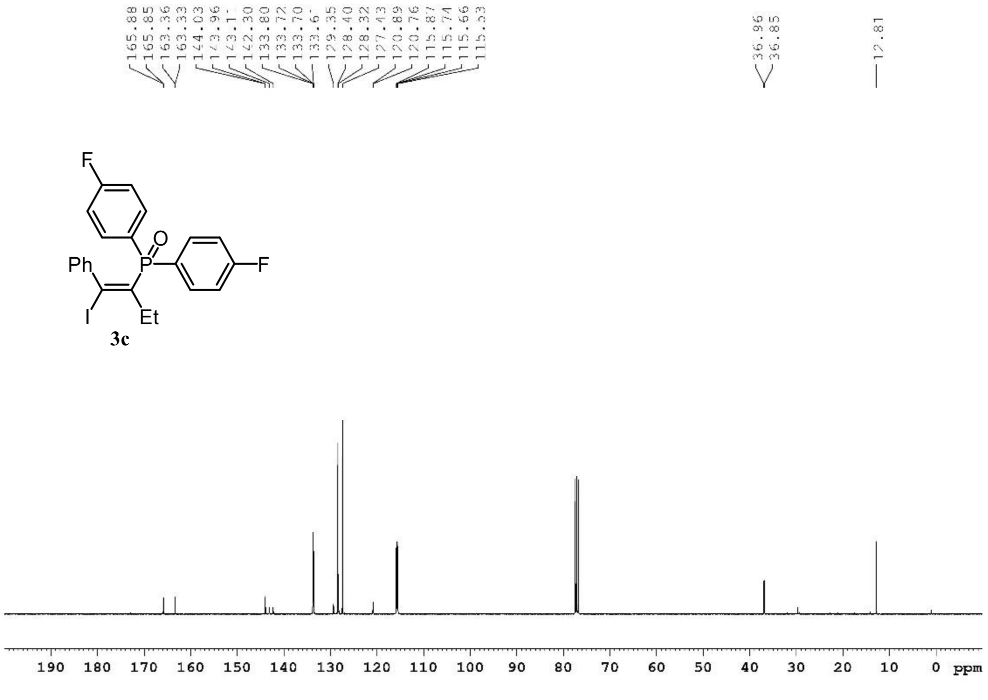

^{31}P NMR (162 MHz, $CDCl_3$) of 3c

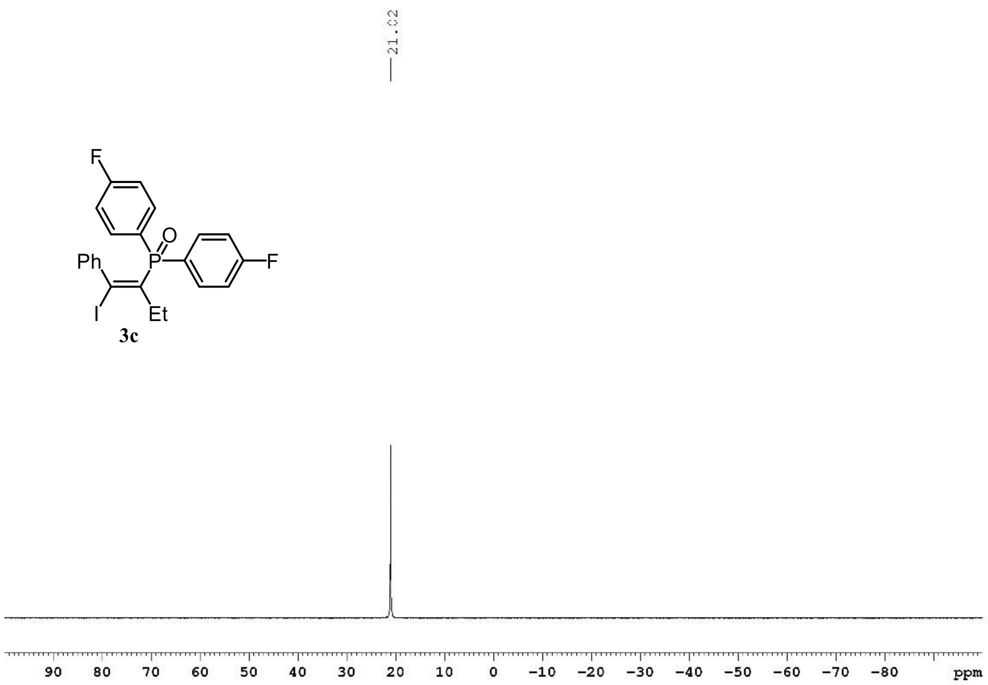

^{1}H NMR (400 MHz, $CDCl_3$) of 3d

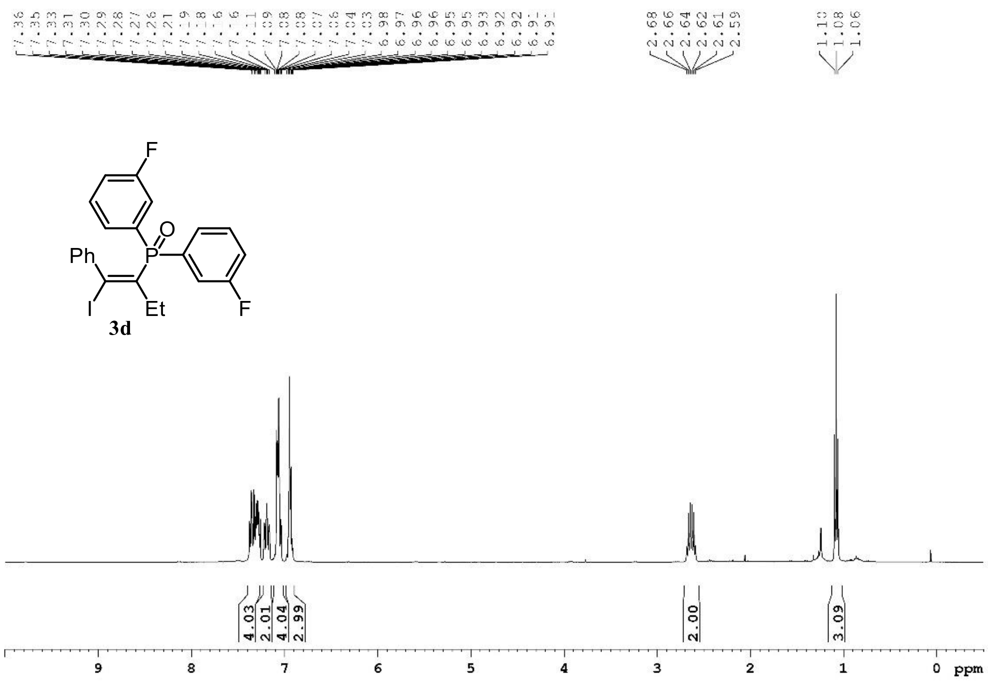

^{13}C NMR (101 MHz, $CDCl_3$) of 3d

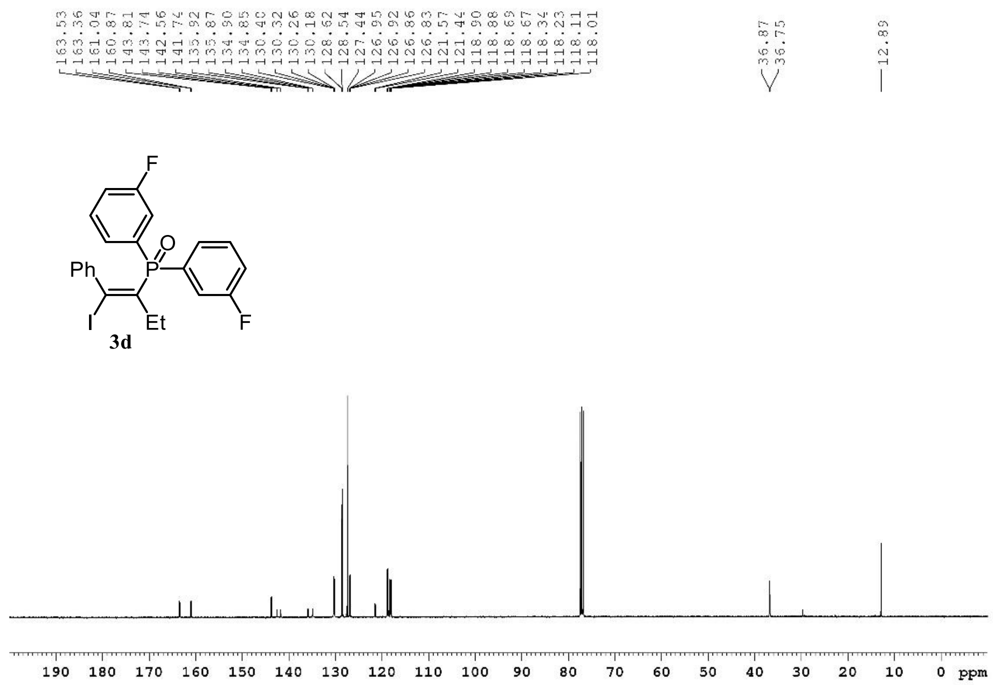

^{31}P NMR (162 MHz, $CDCl_3$) of 3d

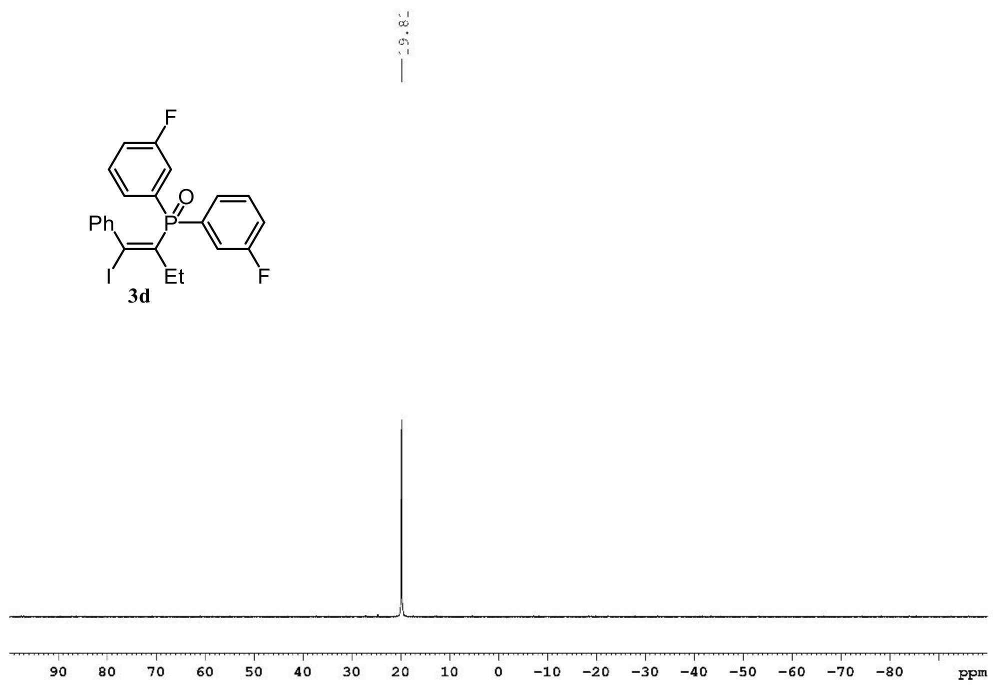

^{1}H NMR (400 MHz, CDCl$_3$) of 3e

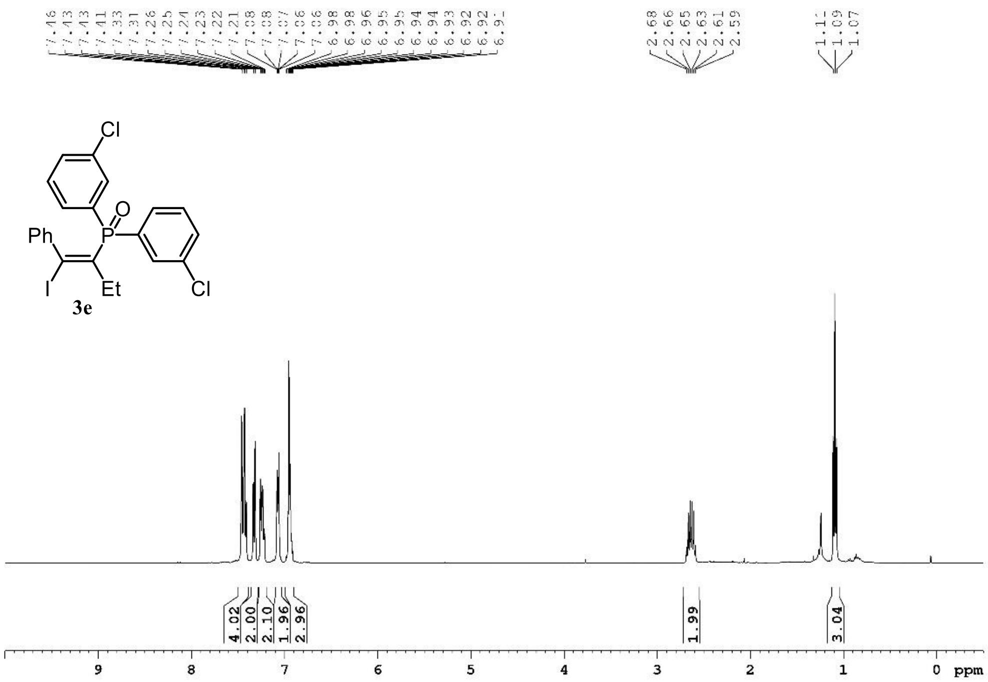

^{13}C NMR (101 MHz, CDCl$_3$) of 3e

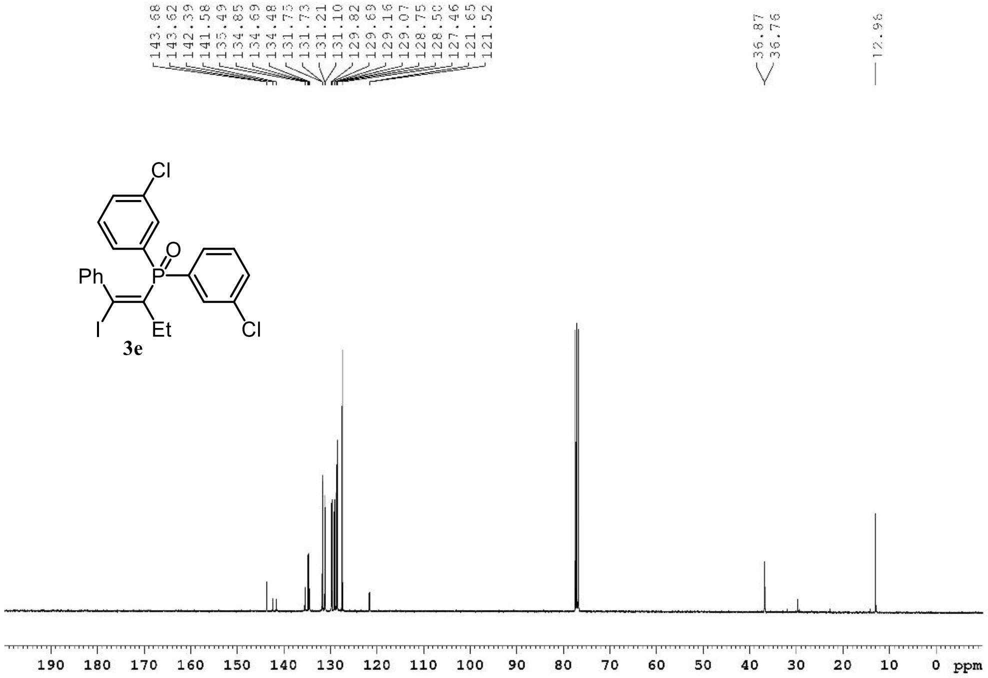

^{31}P NMR (162 MHz, $CDCl_3$) of 3e

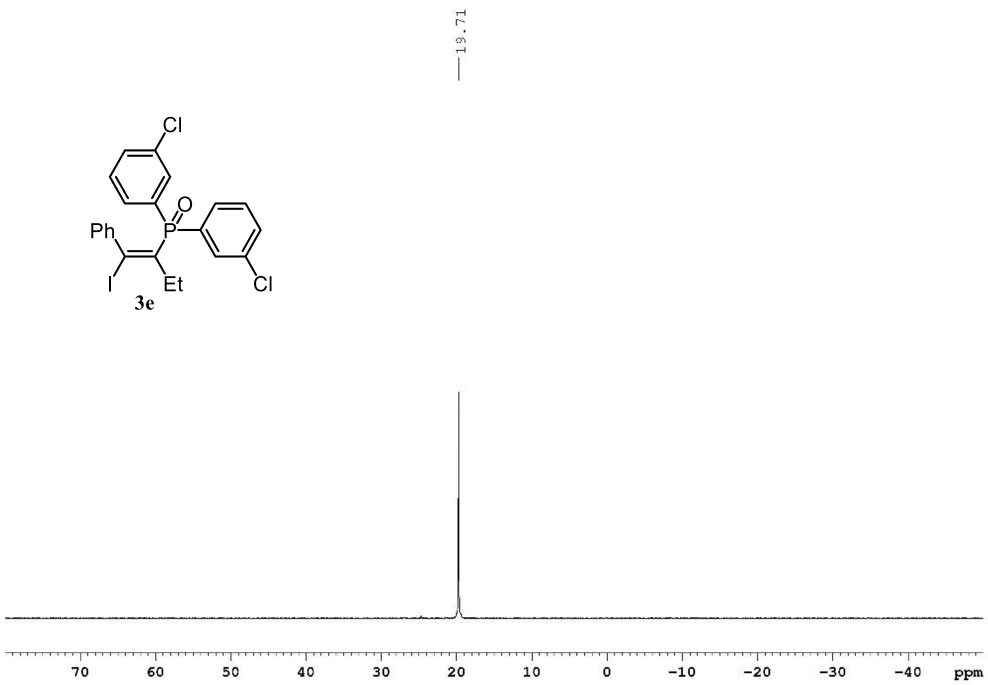

^{1}H NMR (400 MHz, $CDCl_3$) of 3f

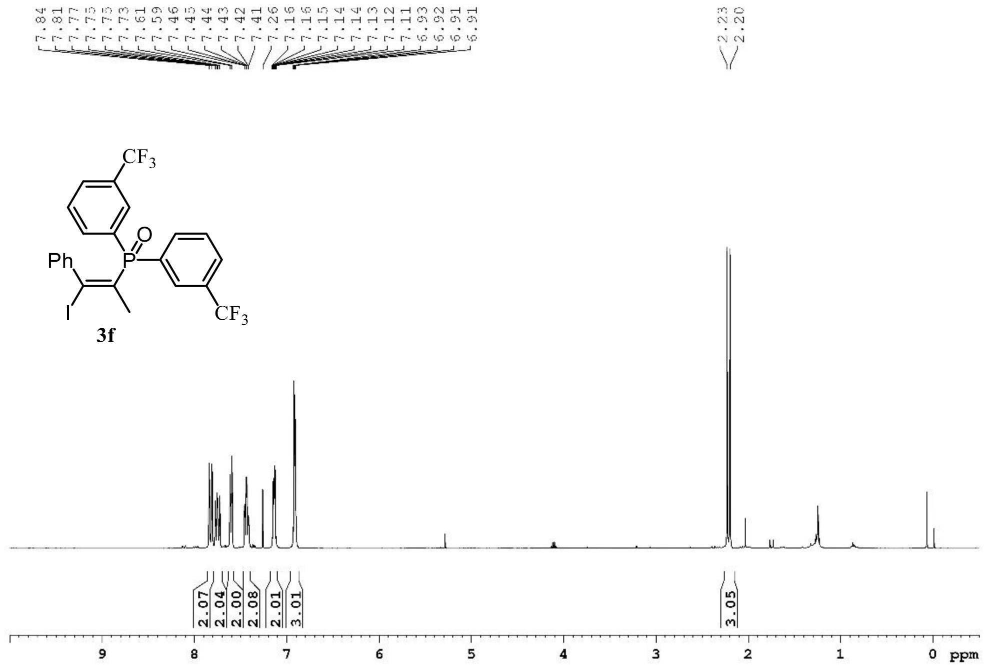

^{13}C NMR (101 MHz, $CDCl_3$) of 3f

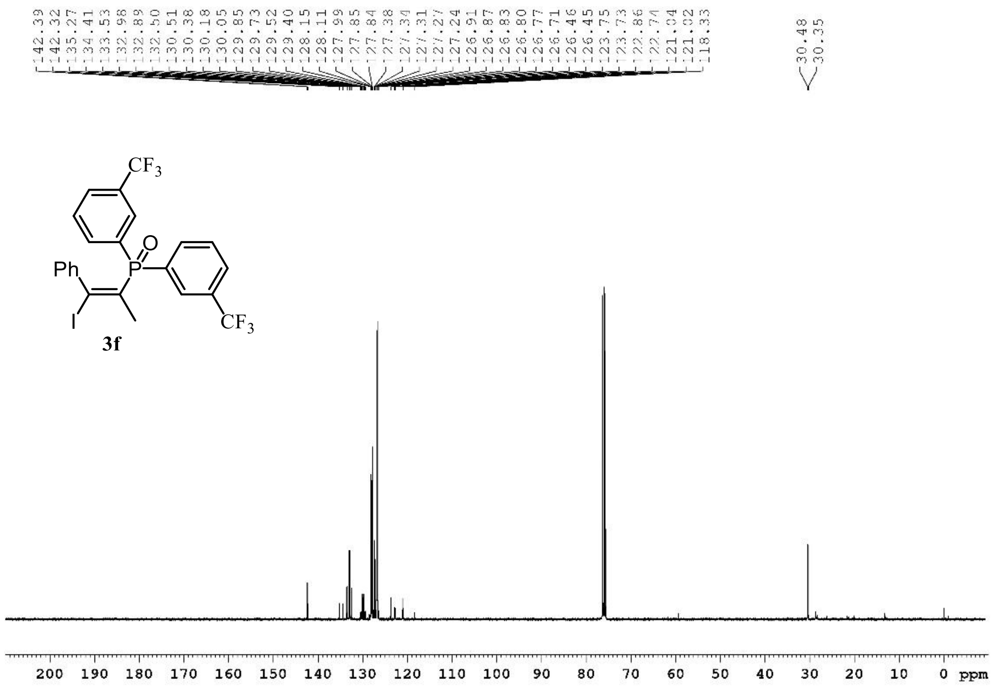

^{31}P NMR (162 MHz, $CDCl_3$) of 3f

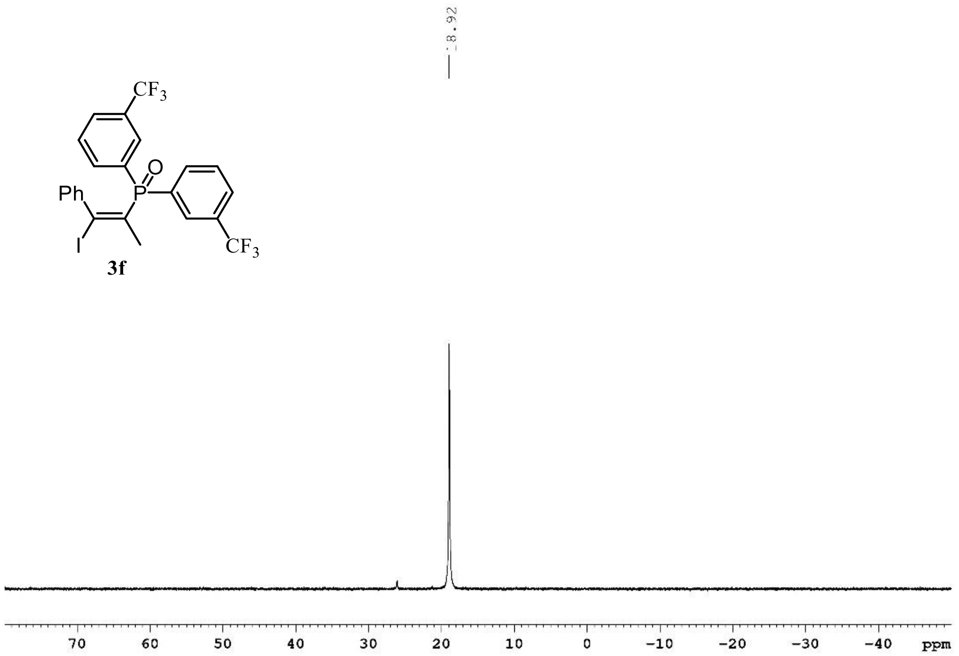

^{1}H NMR (400 MHz, $CDCl_3$) of 3g

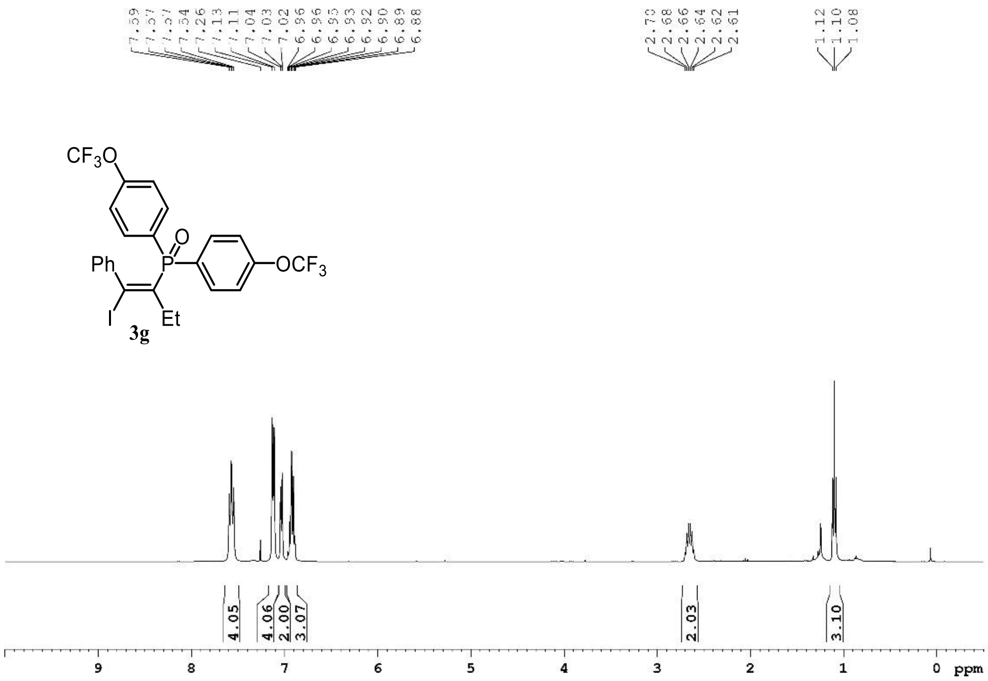

^{13}C NMR (101 MHz, $CDCl_3$) of 3g

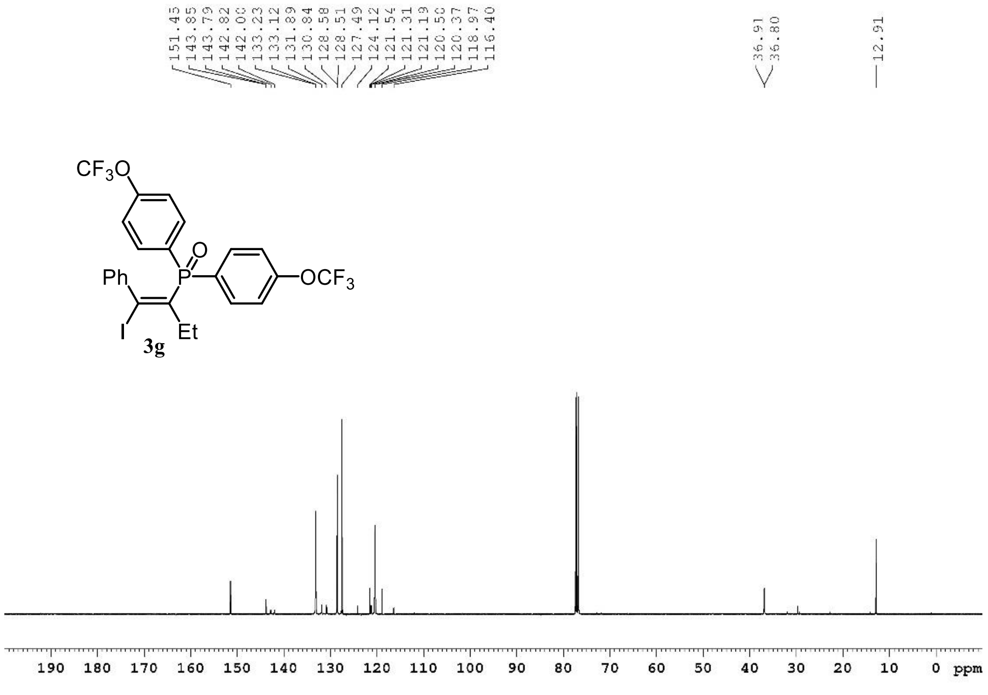

^{31}P NMR (162 MHz, CDCl$_3$) of 3g

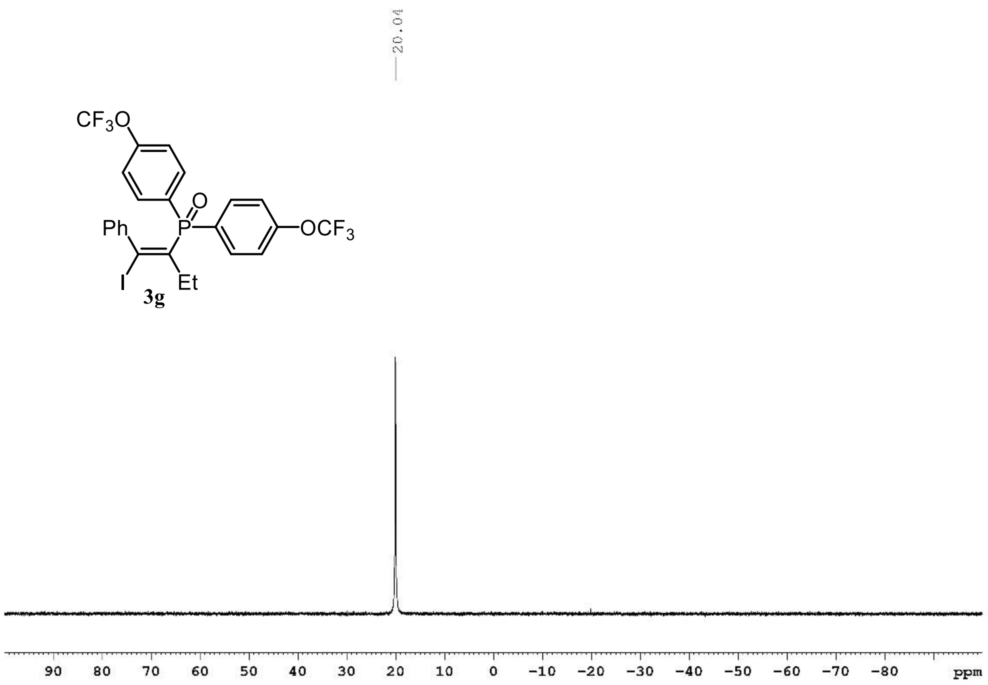

^{1}H NMR (400 MHz, CDCl$_3$) of 3h

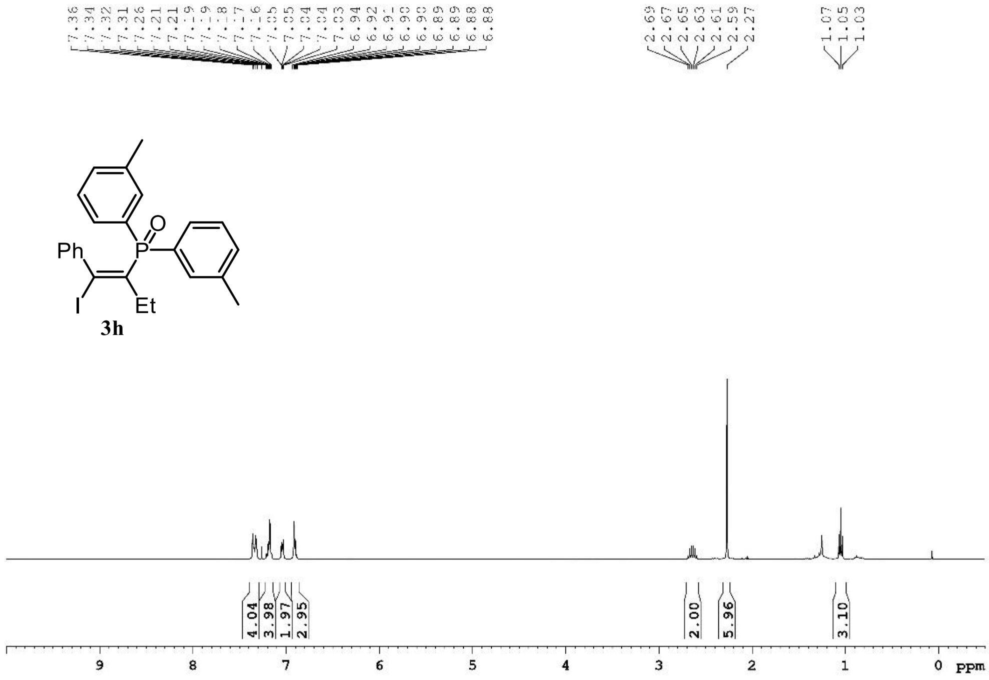

^{13}C NMR (101 MHz, $CDCl_3$) of 3h

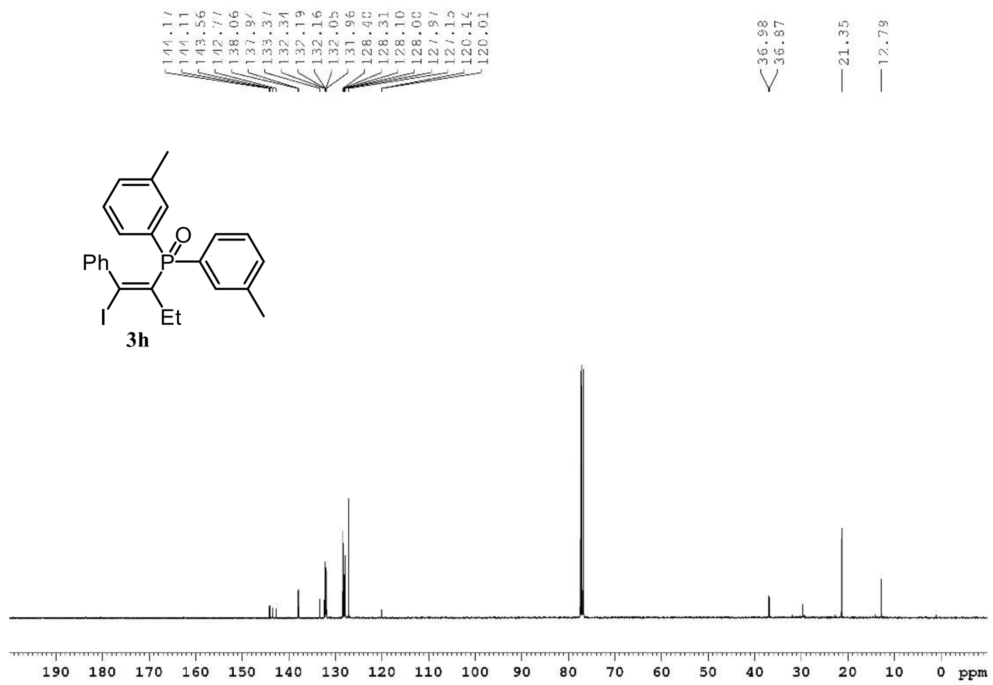

^{31}P NMR (162 MHz, $CDCl_3$) of 3h

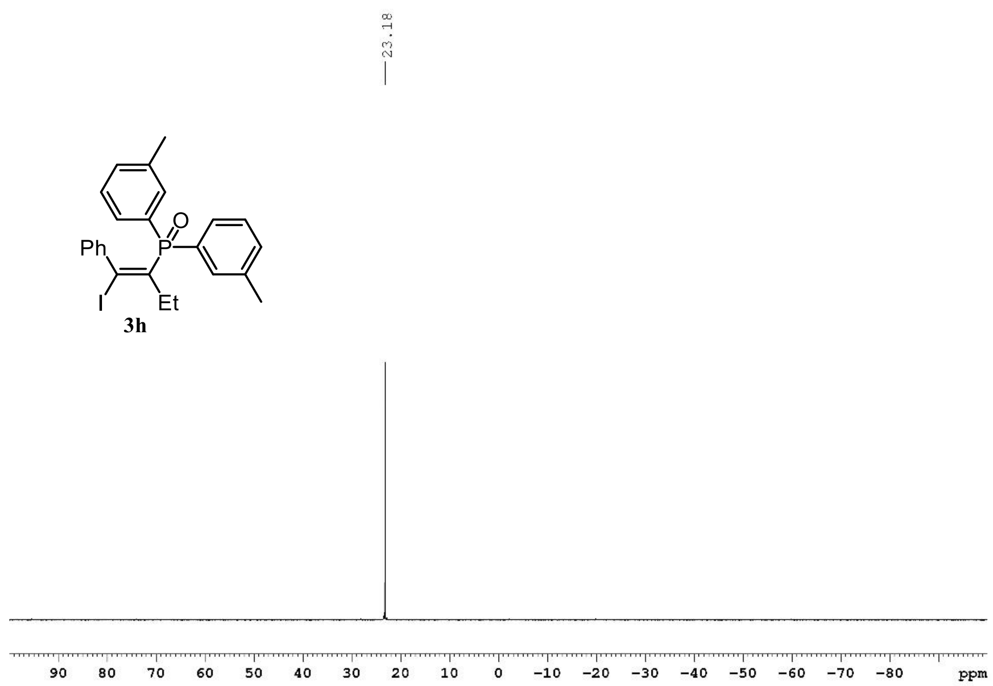

^{1}H NMR (400 MHz, $CDCl_3$) of 3i

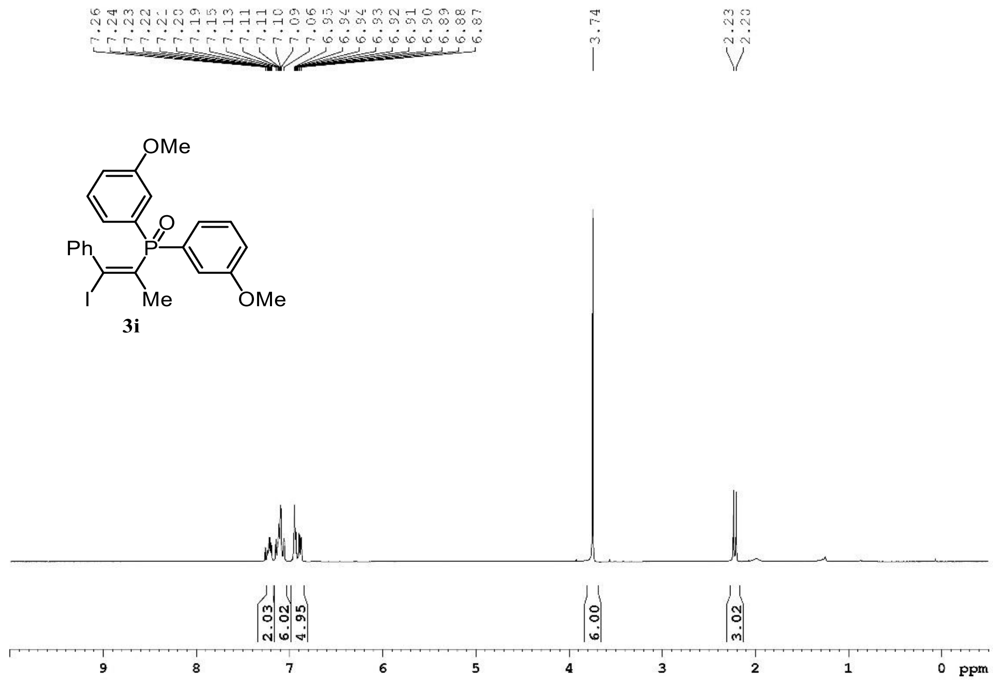

^{13}C NMR (101 MHz, $CDCl_3$) of 3i

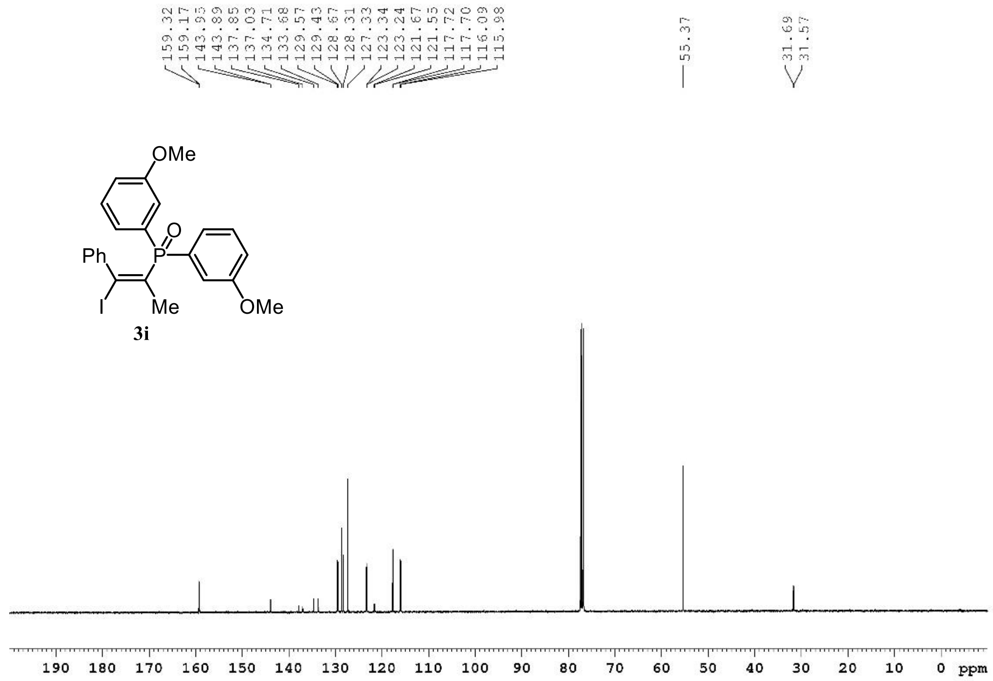

^{31}P NMR (162 MHz, CDCl$_3$) of 3i

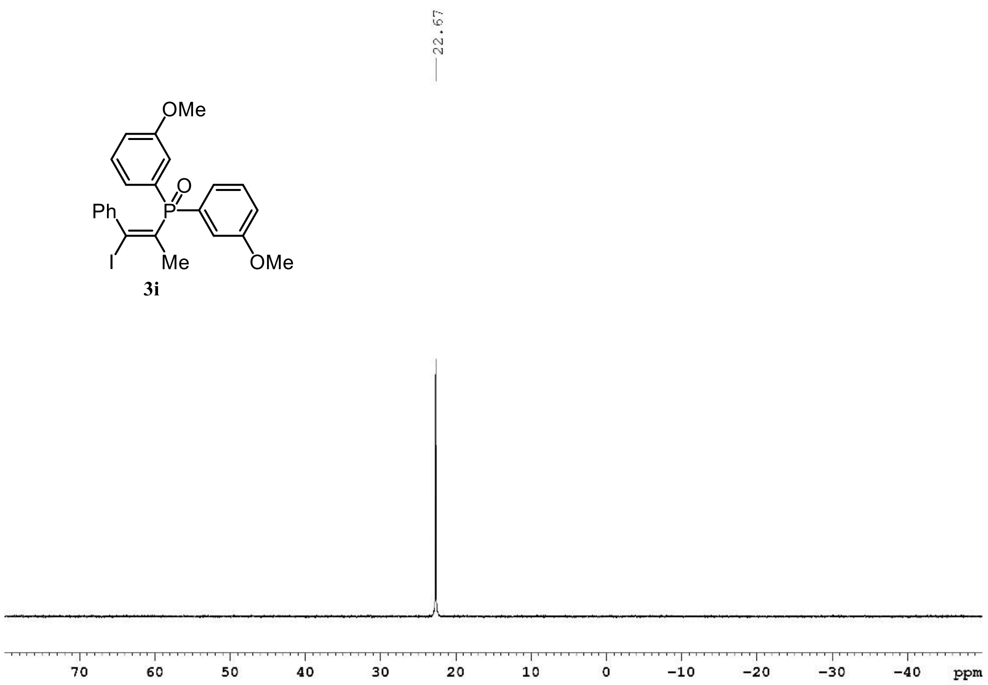

^{1}H NMR (400 MHz, CDCl$_3$) of 3j

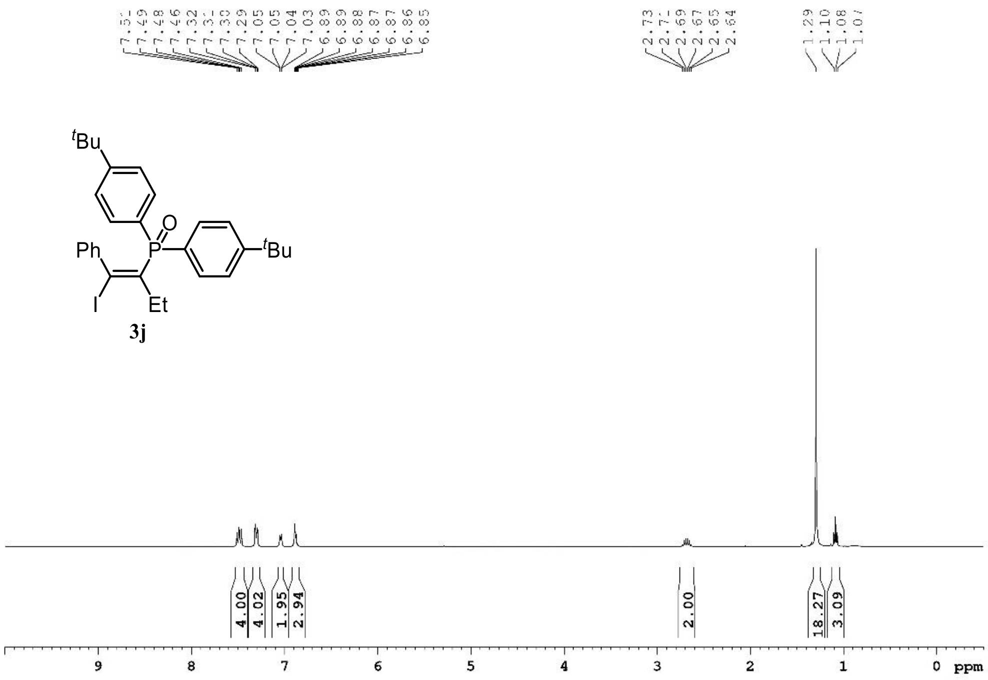

^{13}C NMR (101 MHz, $CDCl_3$) of 3j

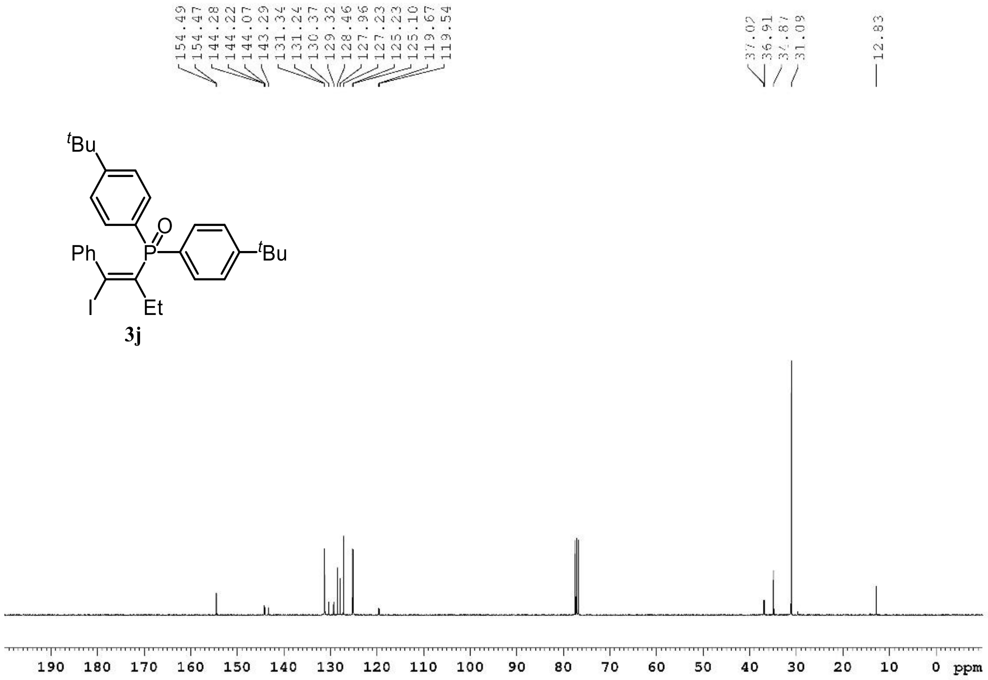

^{31}P NMR (162 MHz, $CDCl_3$) of 3j

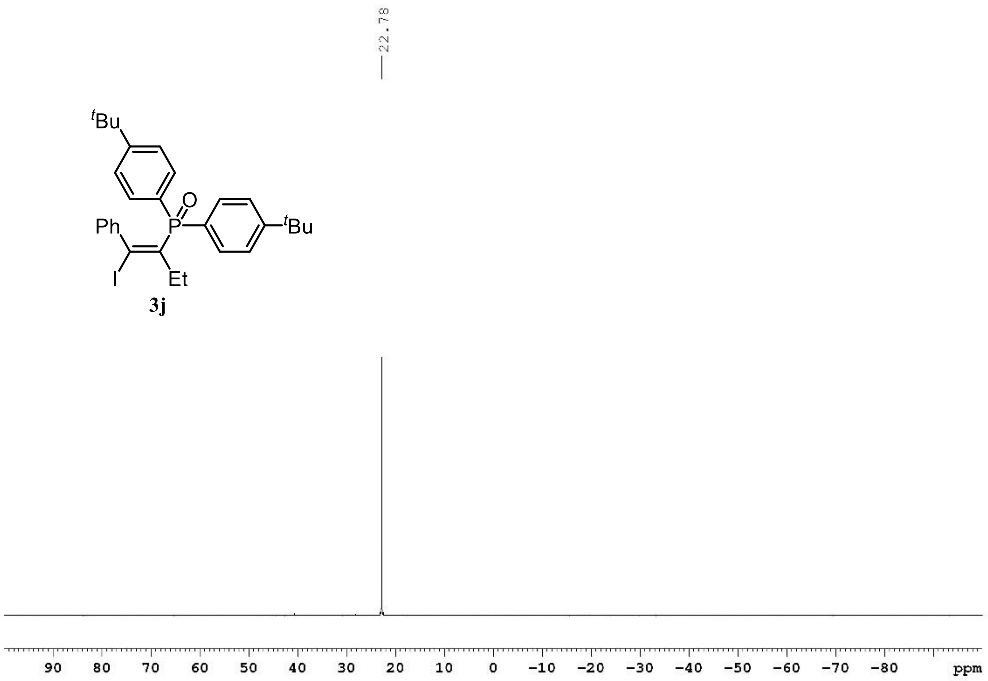

^{1}H NMR (400 MHz, $CDCl_3$) of 3k

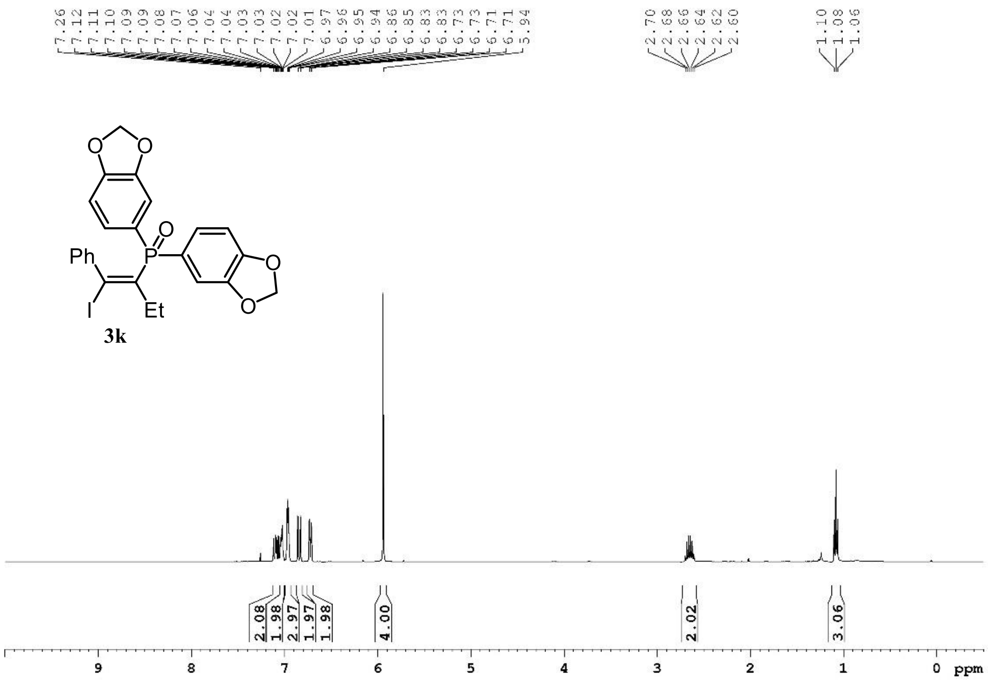

^{13}C NMR (101 MHz, $CDCl_3$) of 3k

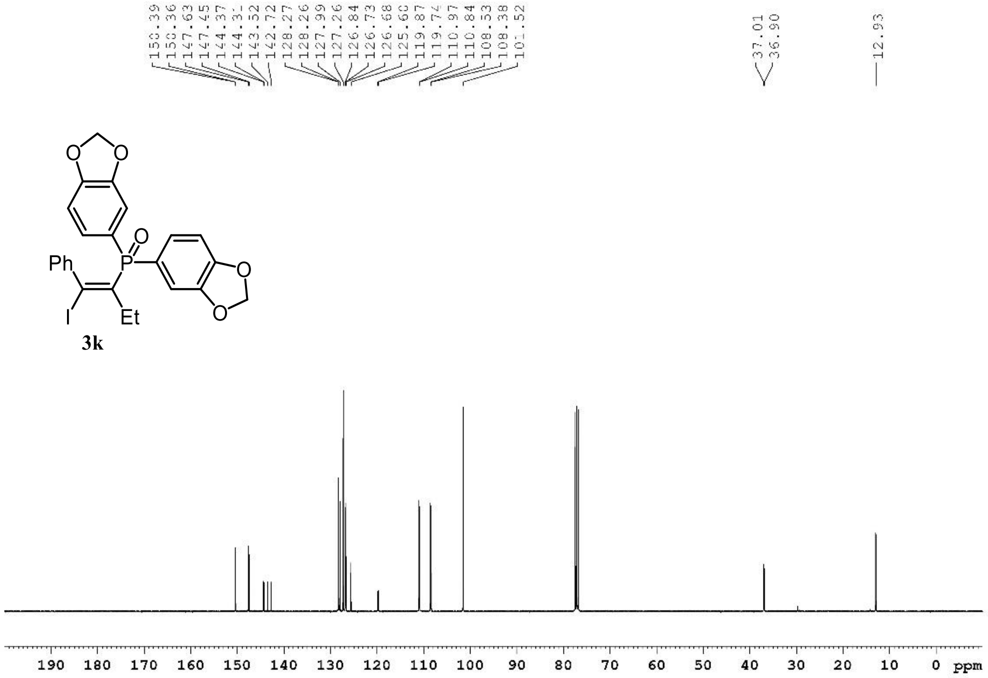

^{31}P NMR (162 MHz, CDCl3) of 3k

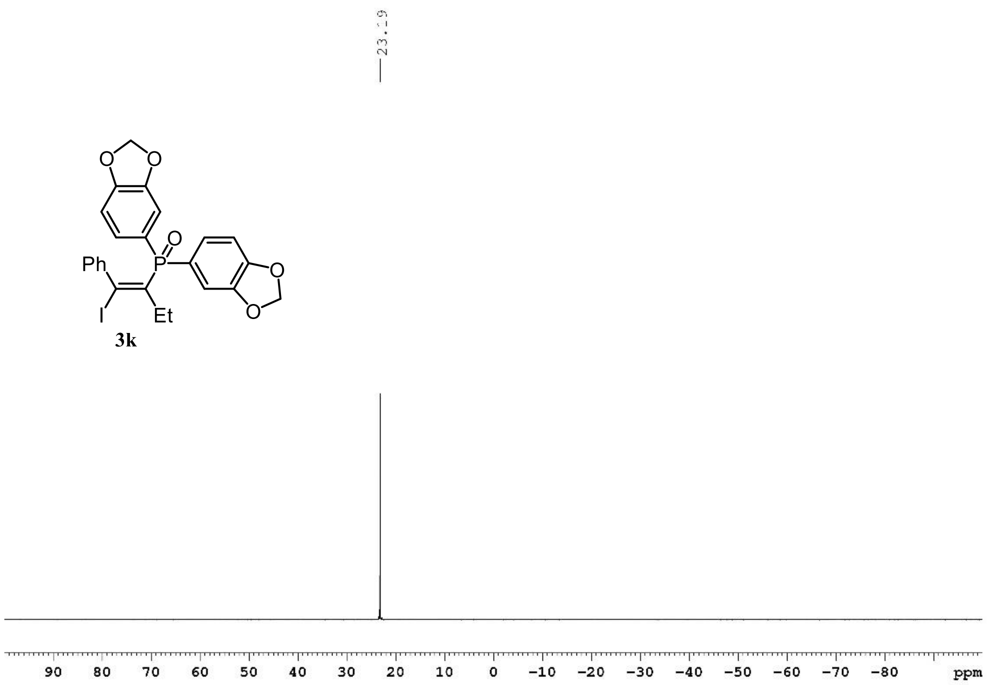

^{1}H NMR (400 MHz, CDCl3) of 3l

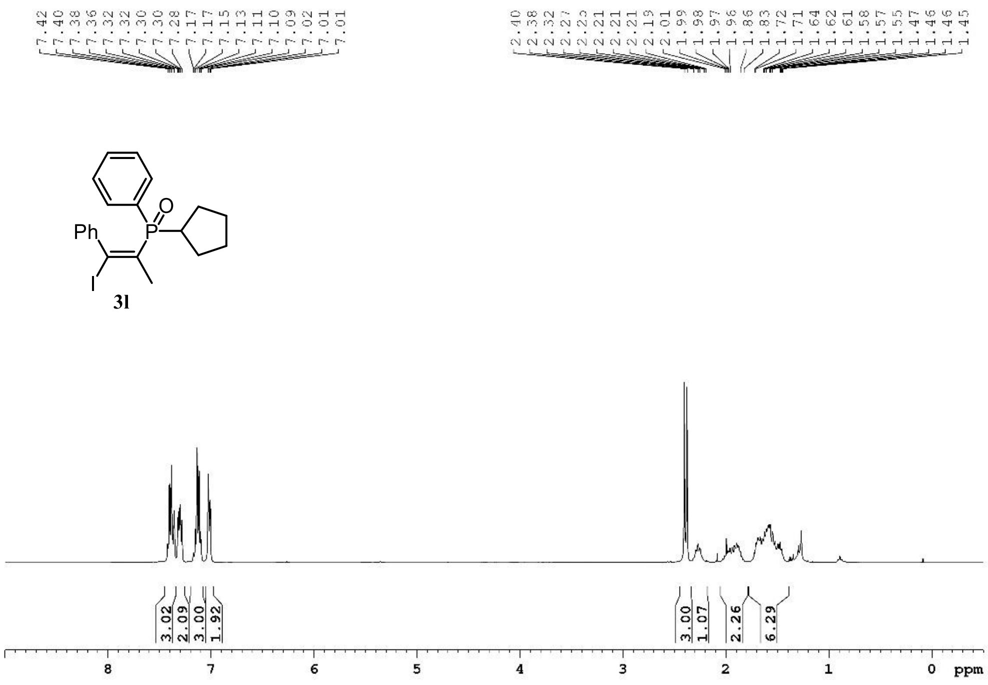

^{13}C NMR (101 MHz, $CDCl_3$) of 3l

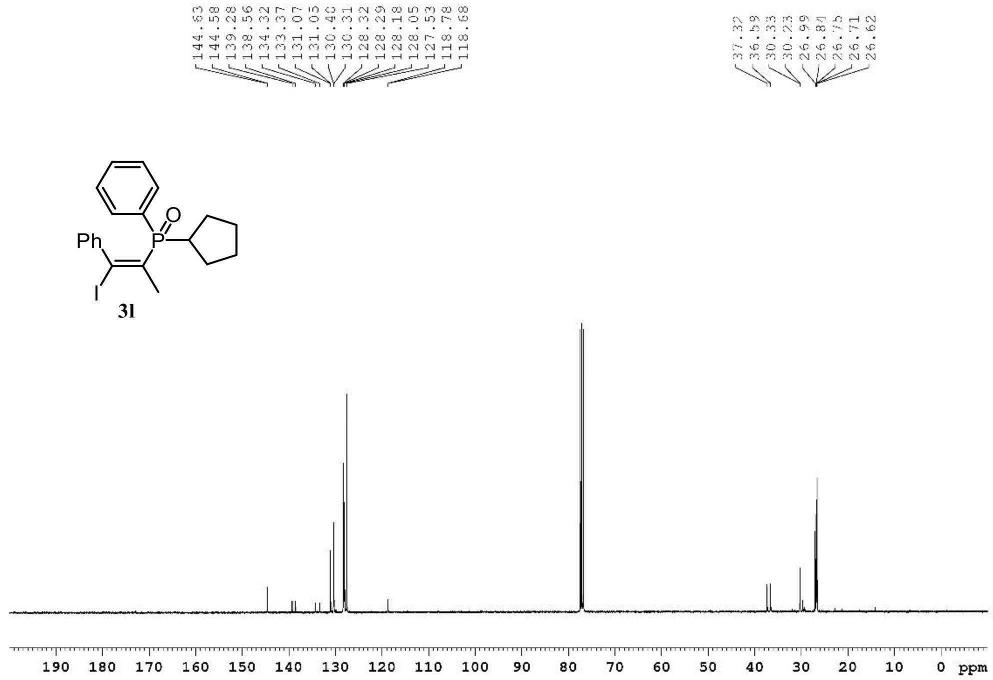

^{31}P NMR (162 MHz, $CDCl_3$) of 3l

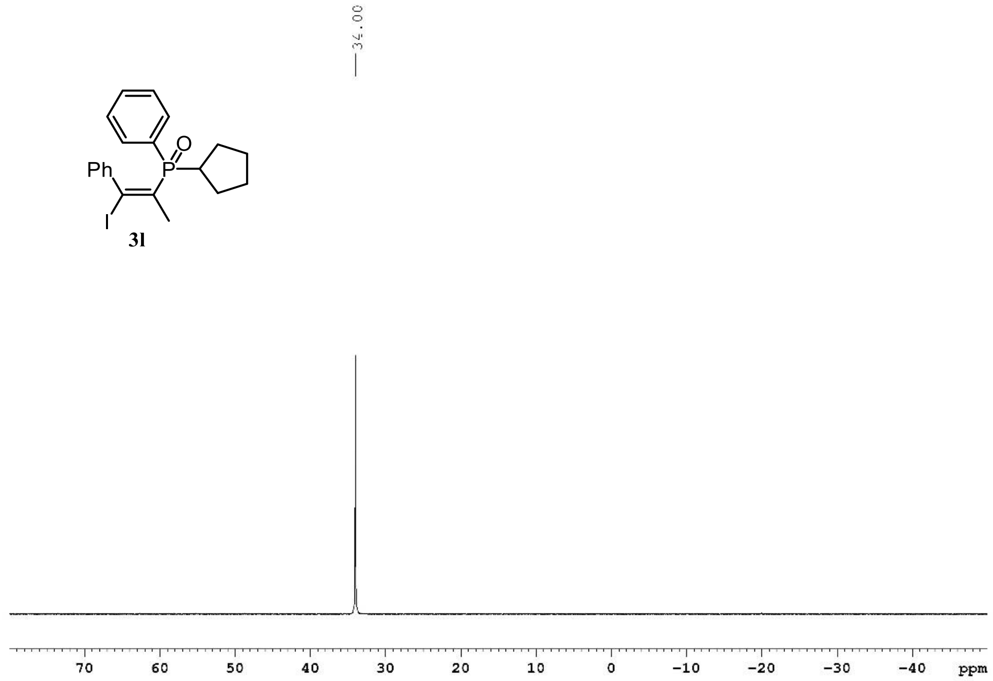

^{1}H NMR (400 MHz, $CDCl_3$) of 3m

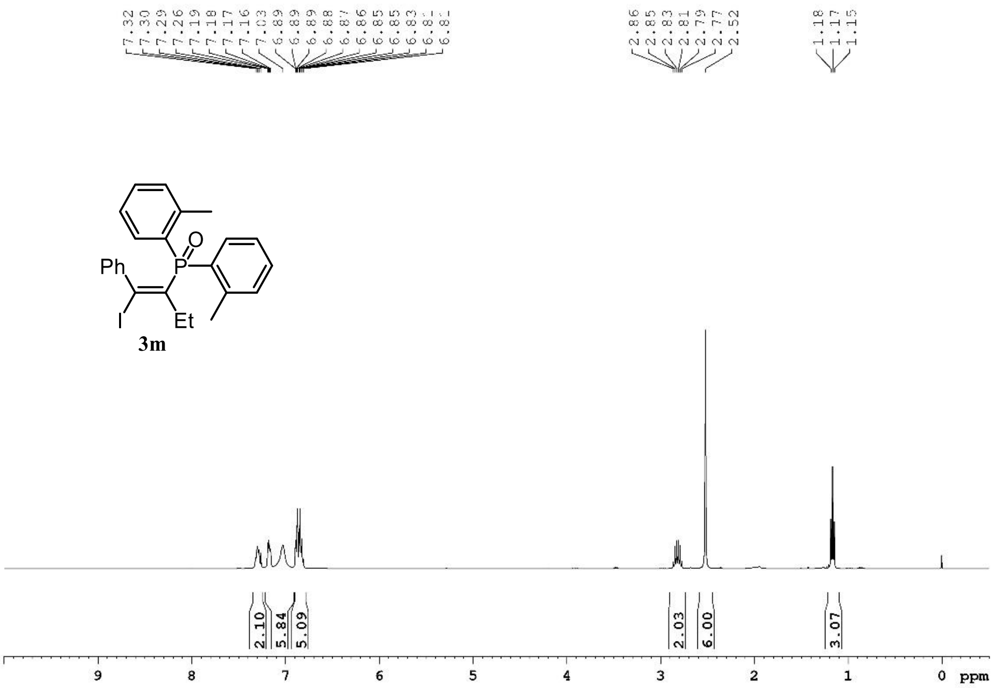

^{13}C NMR (101 MHz, $CDCl_3$) of 3m

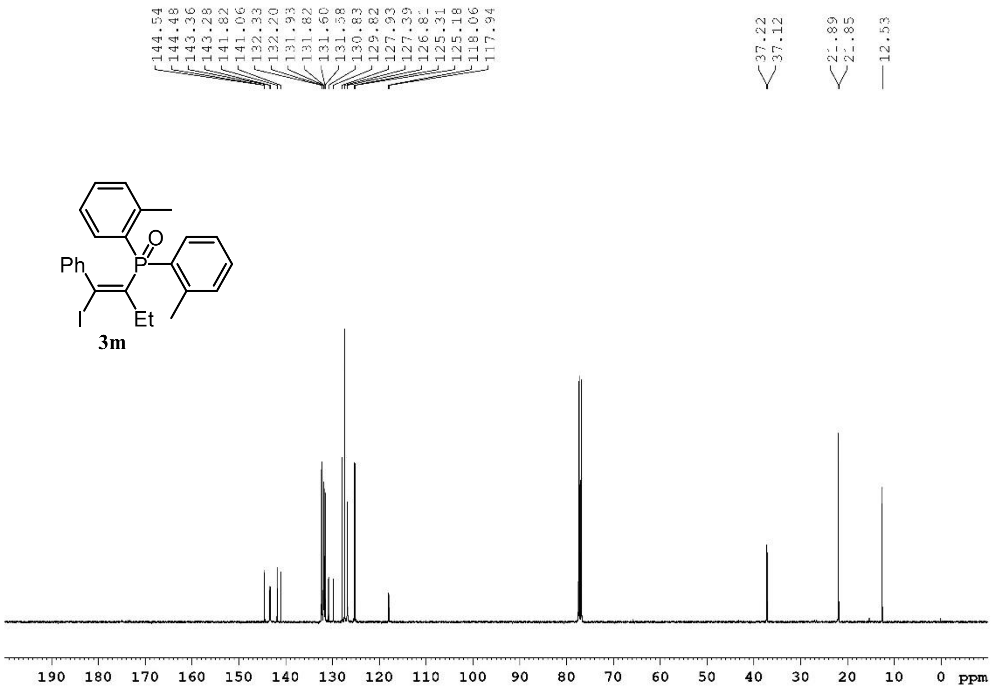

^{31}P NMR (162 MHz, $CDCl_3$) of 3m

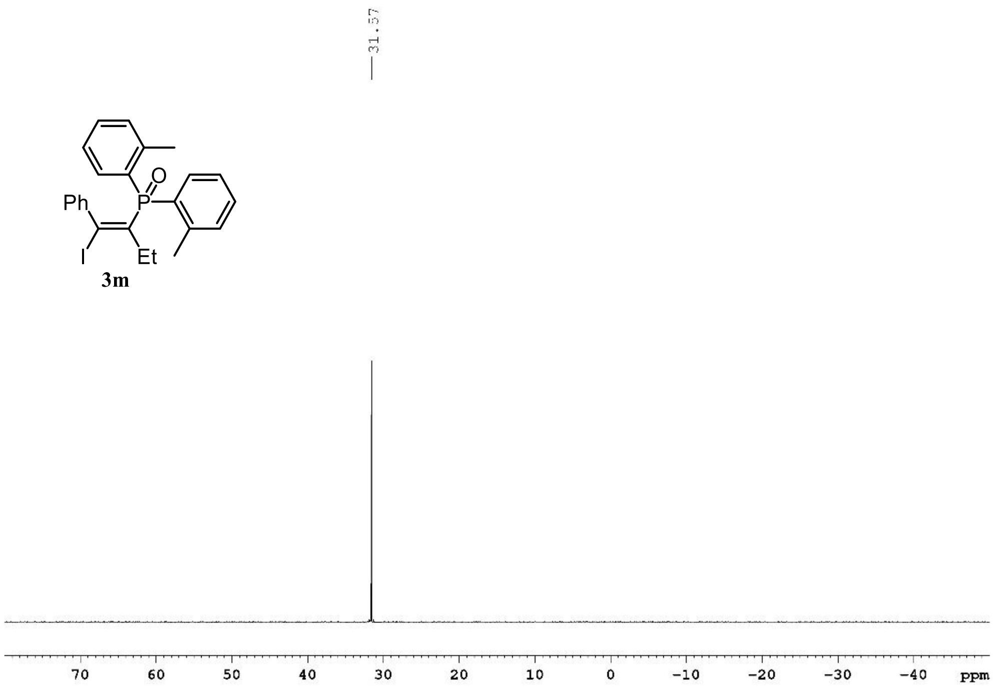

^{1}H NMR (400 MHz, $CDCl_3$) of 3m'

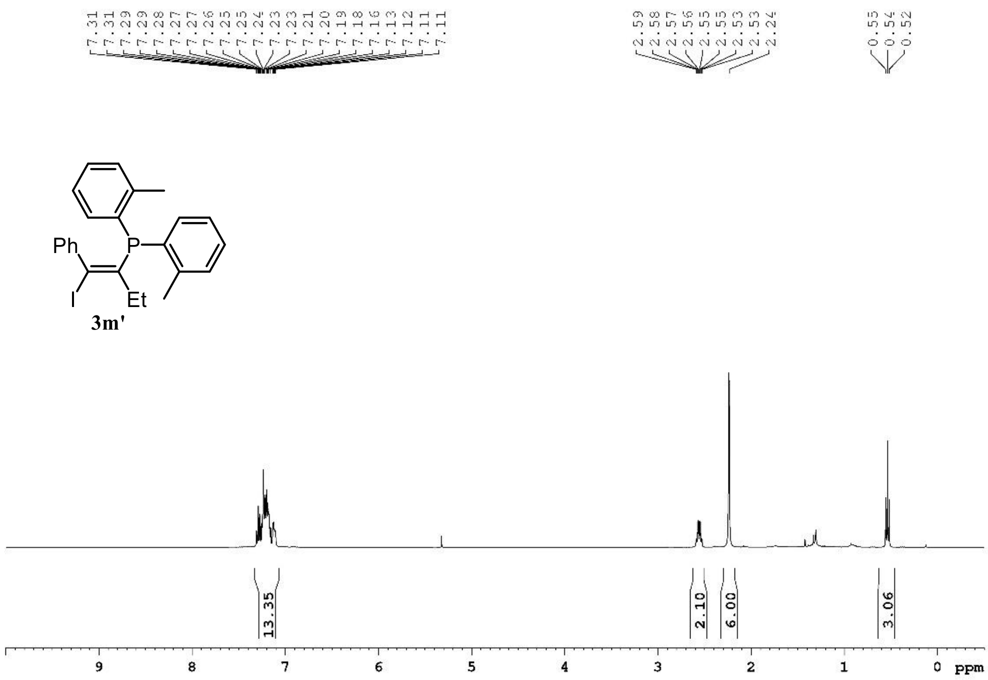

^{13}C NMR (101 MHz, $CDCl_3$) of 3m’

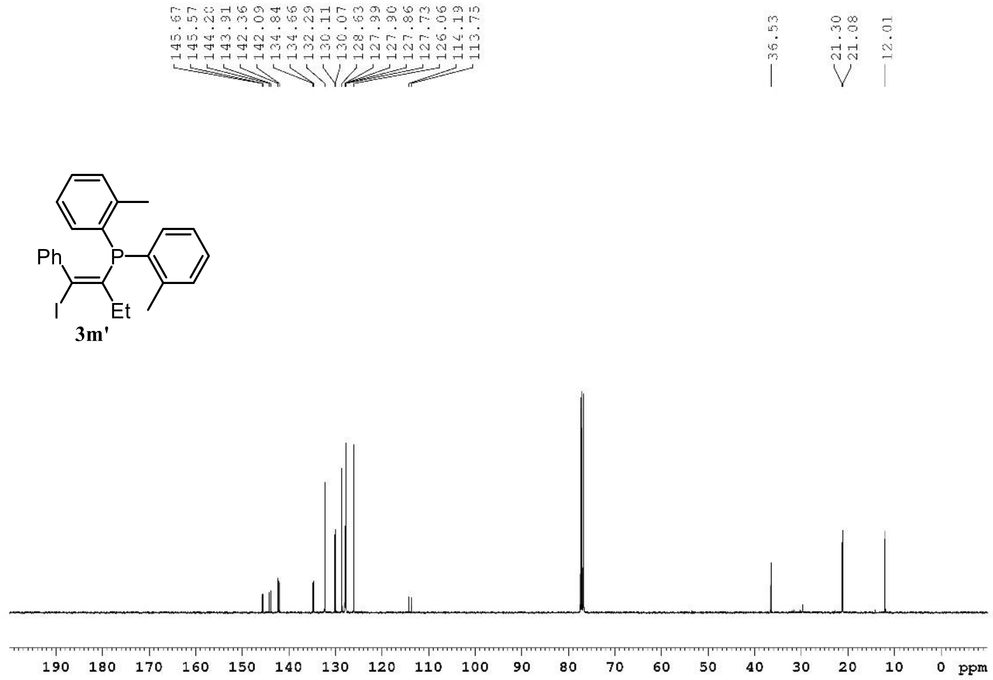

^{31}P NMR (162 MHz, $CDCl_3$) of 3m’

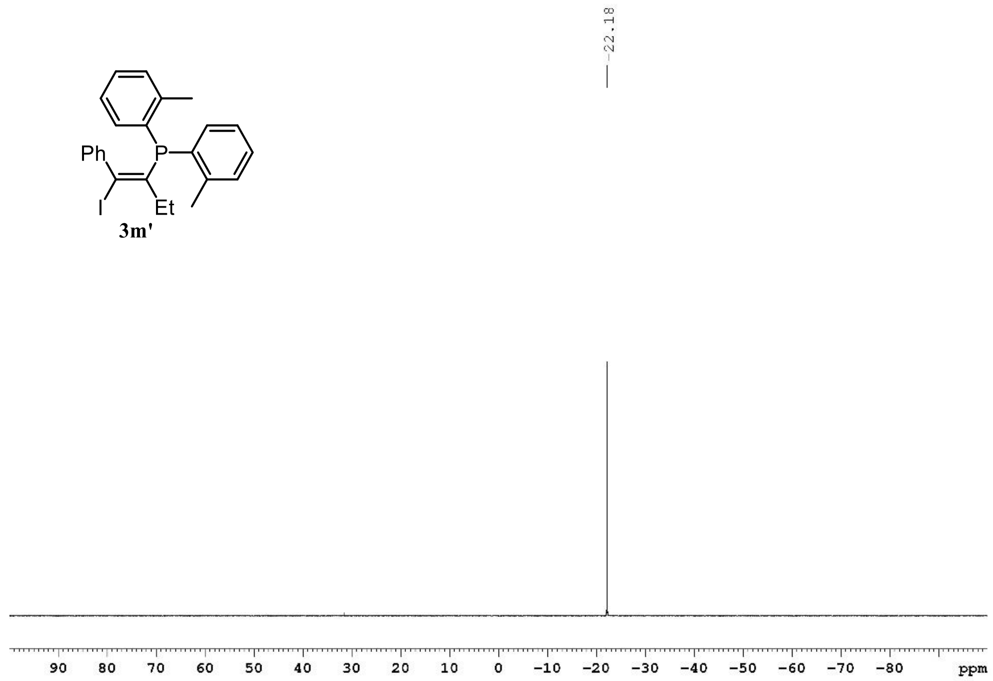

^{1}H NMR (400 MHz, CDCl$_3$) of 3n

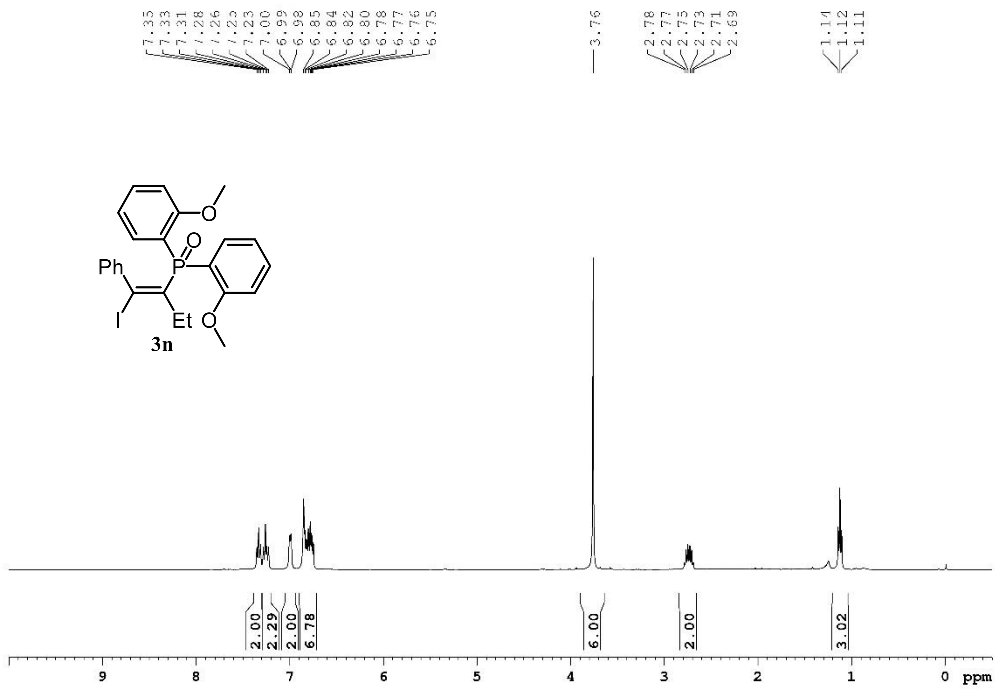

^{13}C NMR (101 MHz, CDCl$_3$) of 3n

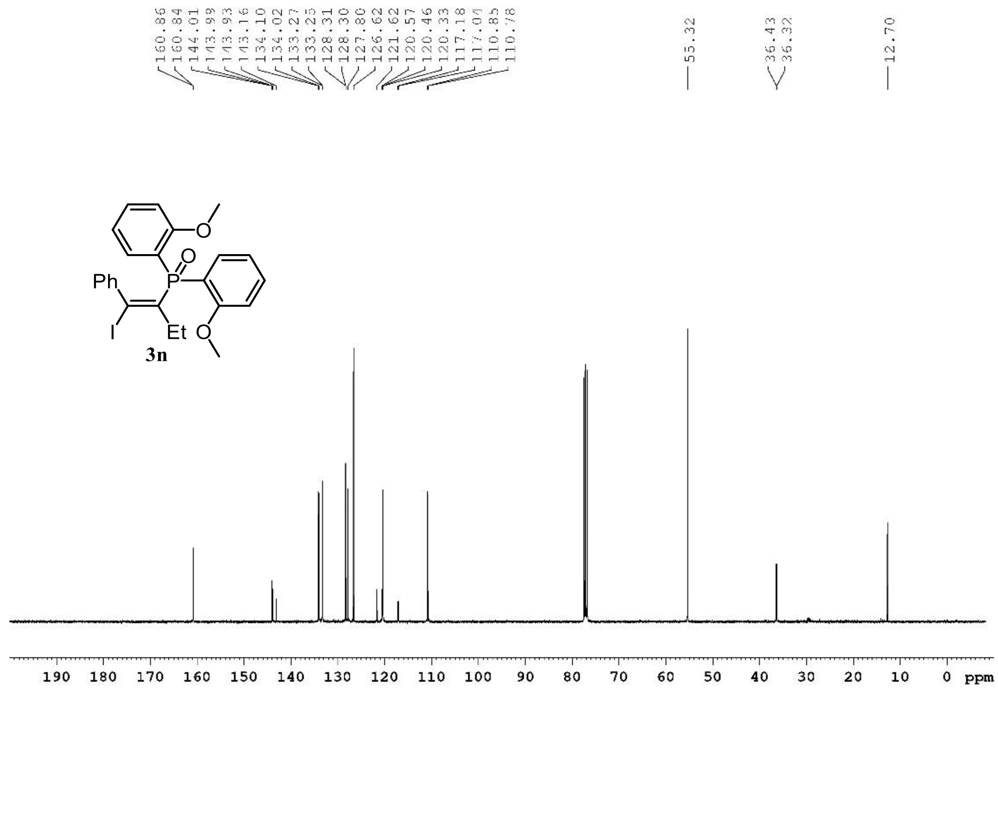

^{31}P NMR (162 MHz, $CDCl_3$) of 3n

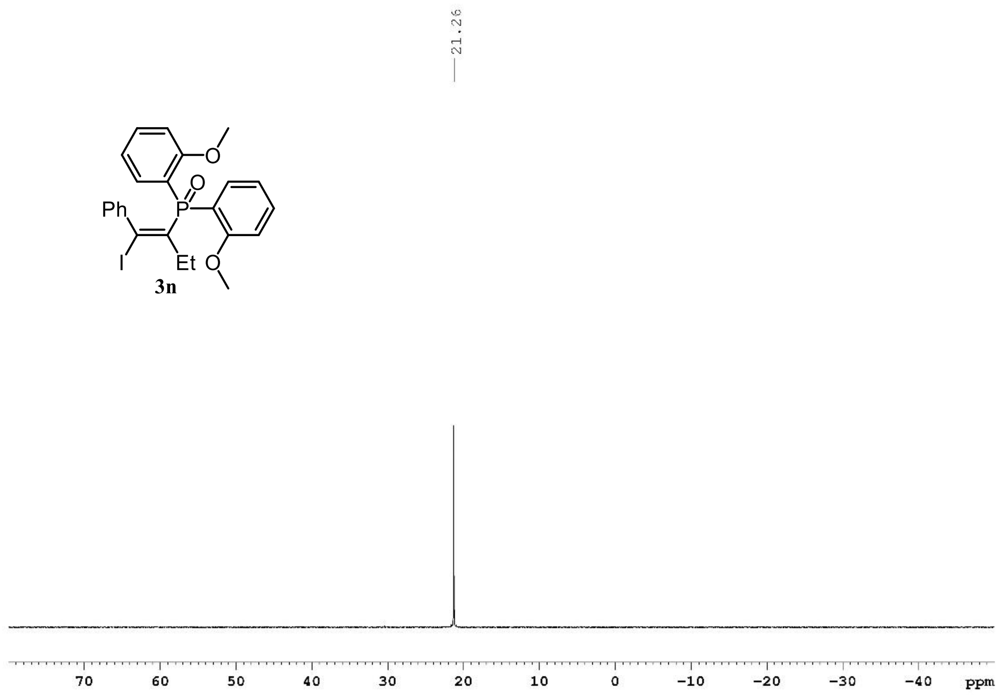

^{1}H NMR (400 MHz, $CDCl_3$) of 3n’

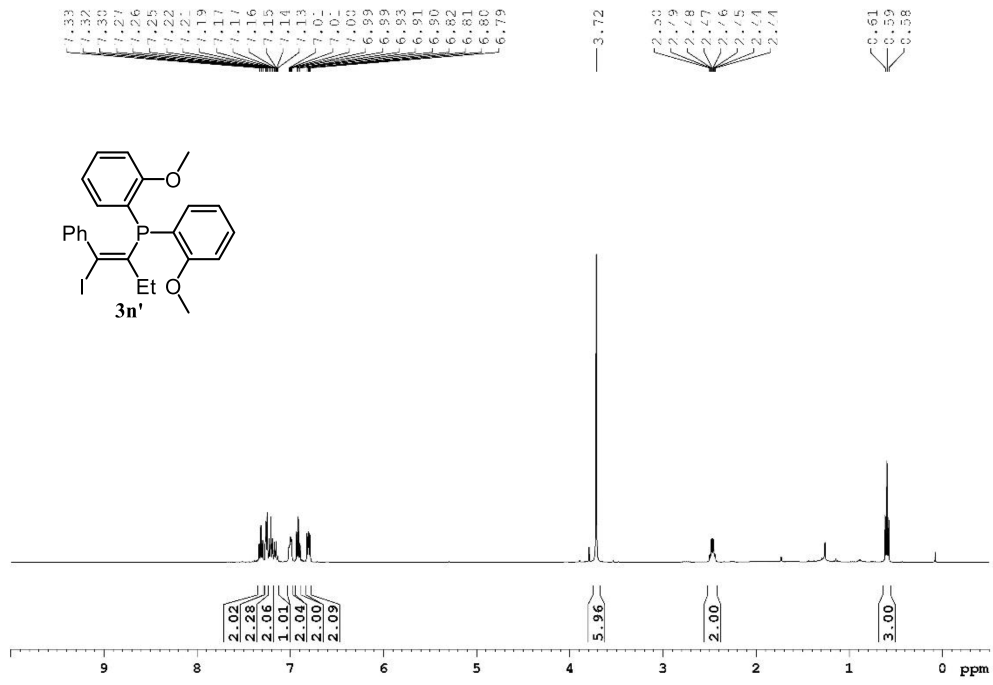

^{13}C NMR (101 MHz, CDCl3) of 3n'

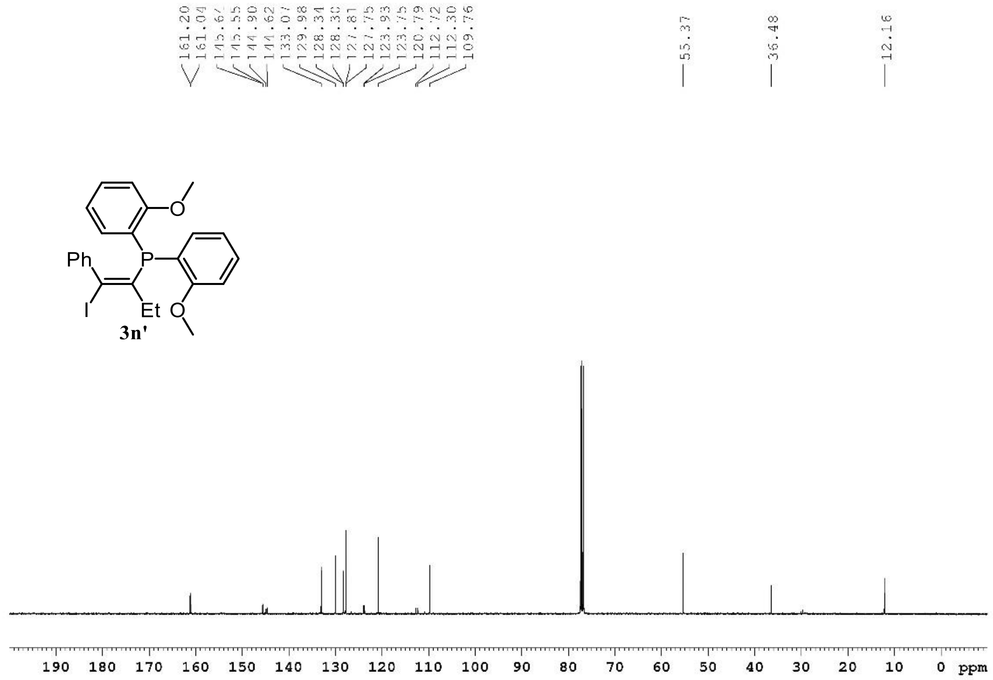

^{31}P NMR (162 MHz, CDCl3) of 3n'

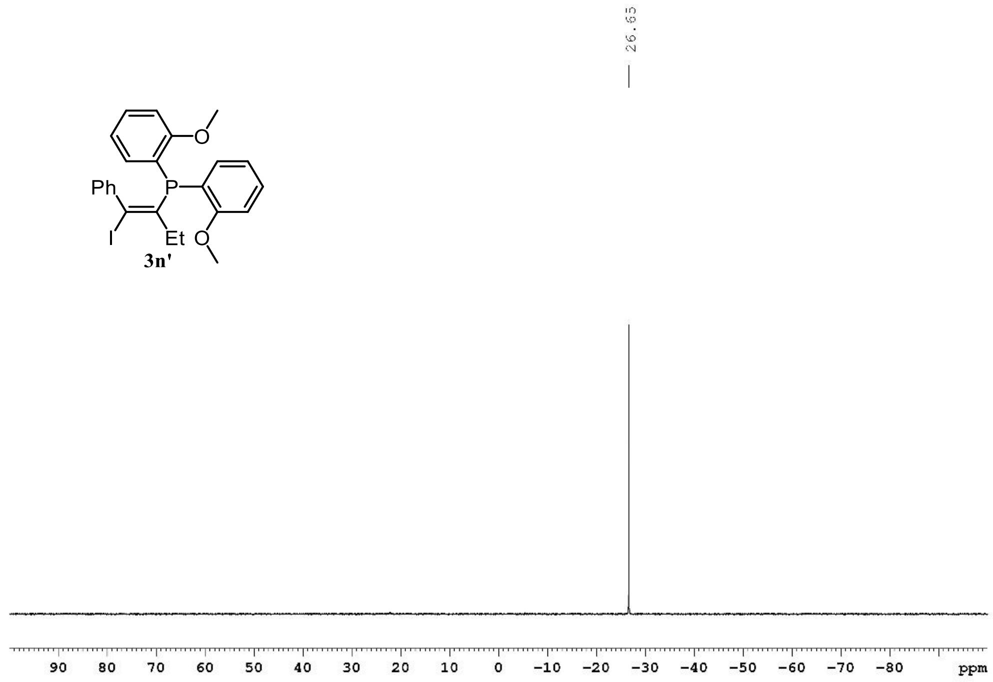

^{1}H NMR (400 MHz, CDCl$_3$) of 4a

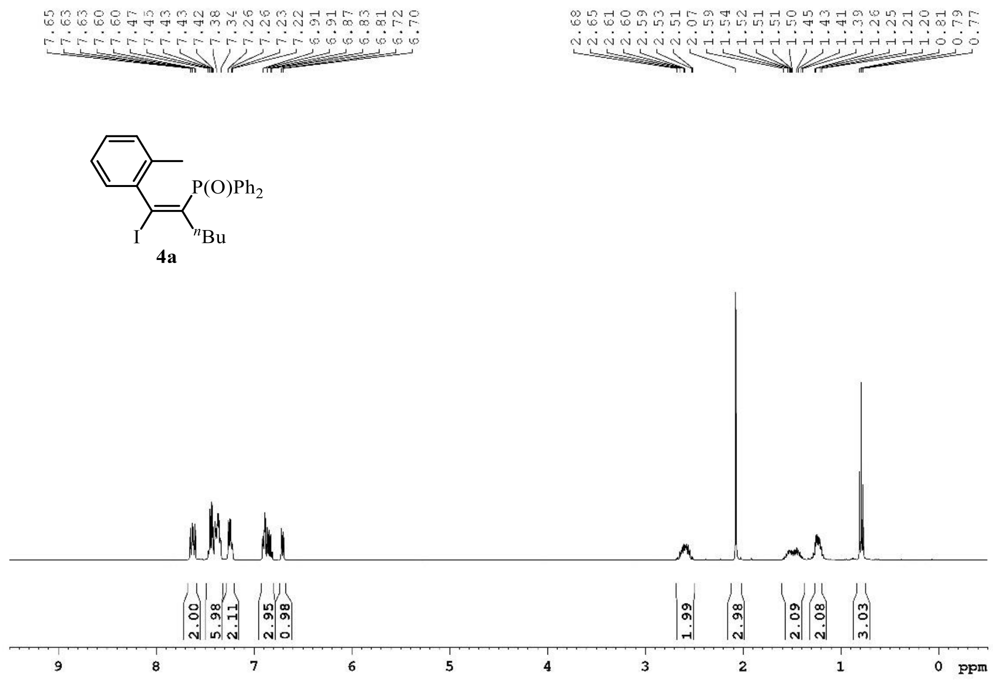

^{13}C NMR (101 MHz, CDCl$_3$) of 4a

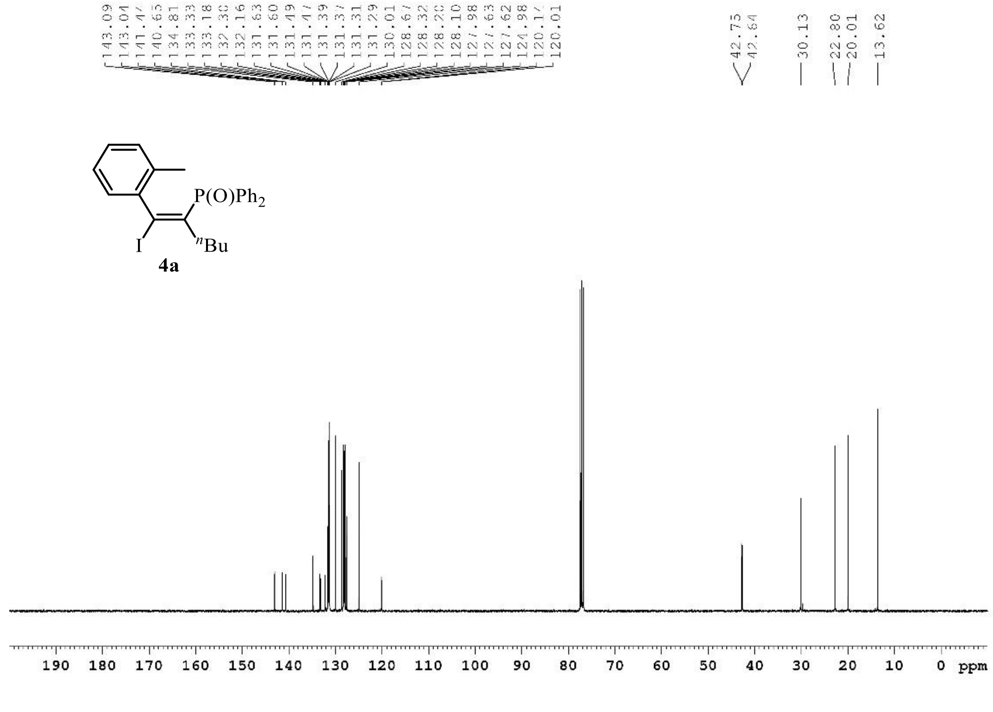

^{31}P NMR (162 MHz, CDCl$_3$) of 4a

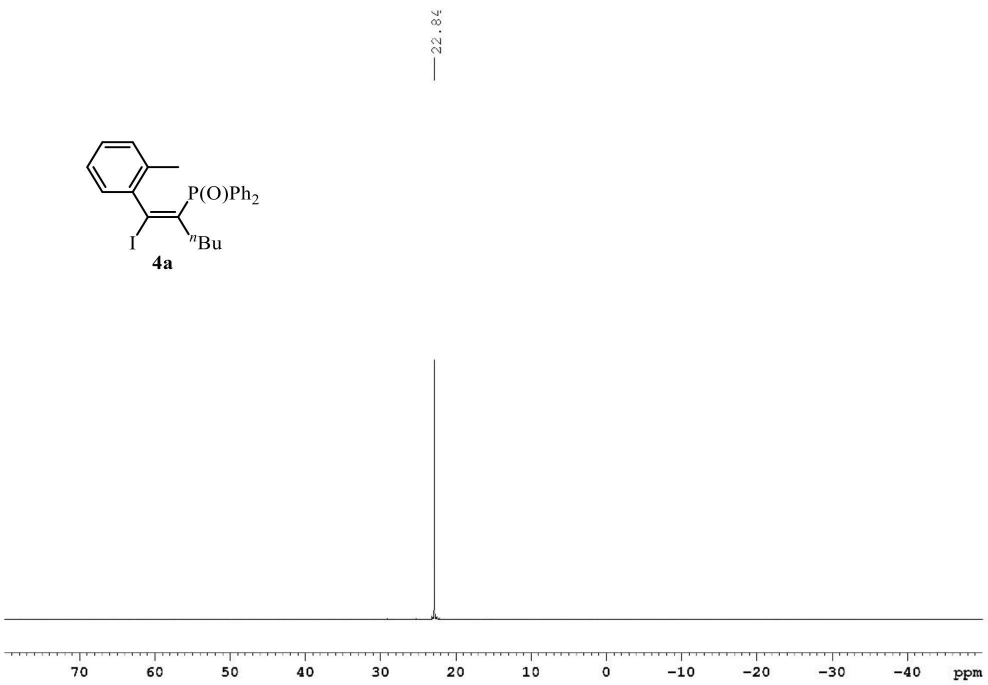

^{1}H NMR (400 MHz, CDCl$_3$) of 4b

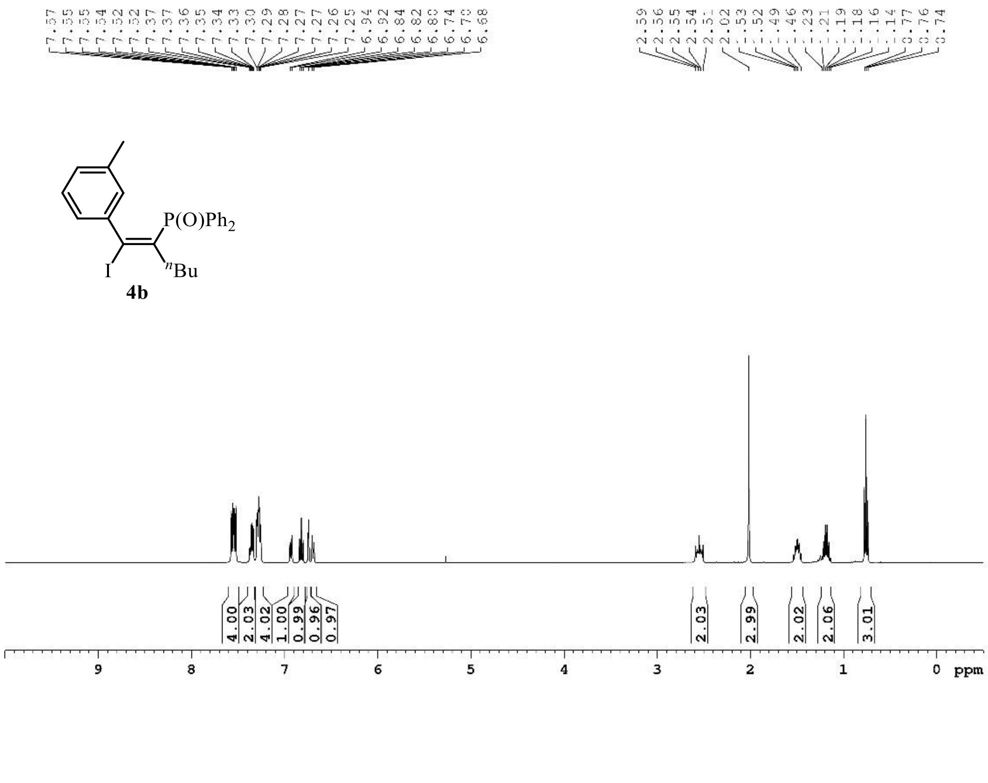

^{13}C NMR (101 MHz, CDCl$_3$) of 4b

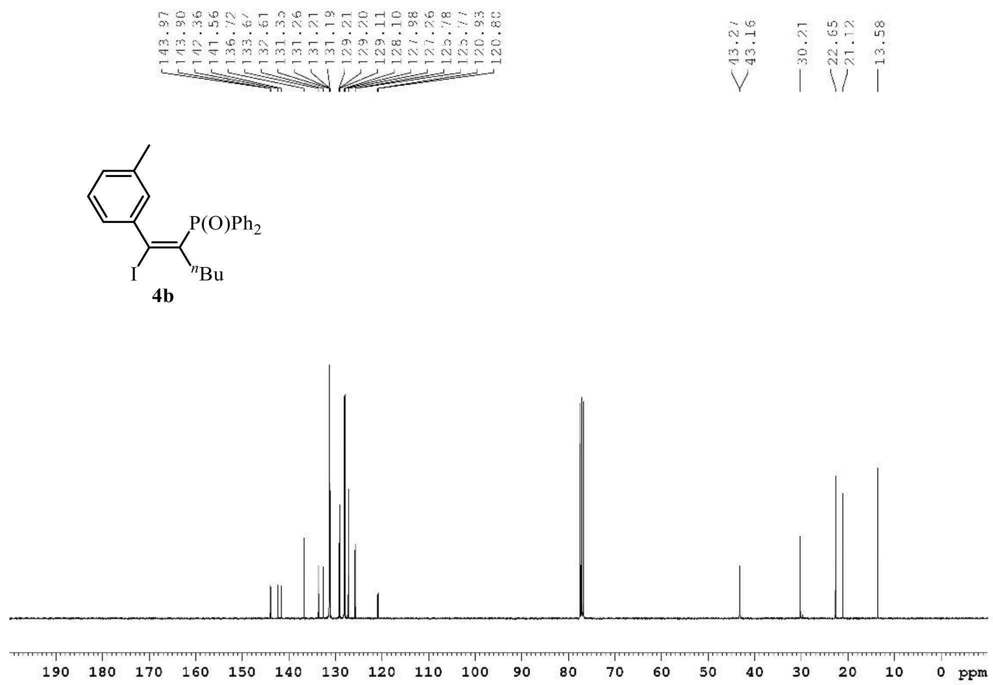

^{31}P NMR (162 MHz, CDCl$_3$) of 4b

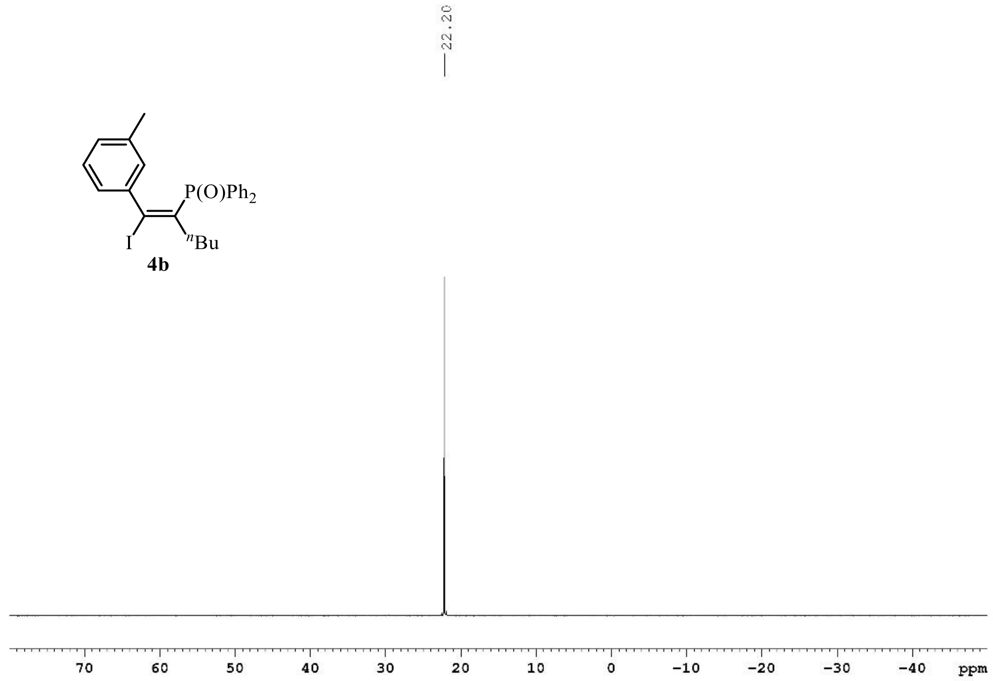

^{1}H NMR (400 MHz, CDCl$_3$) of 4c

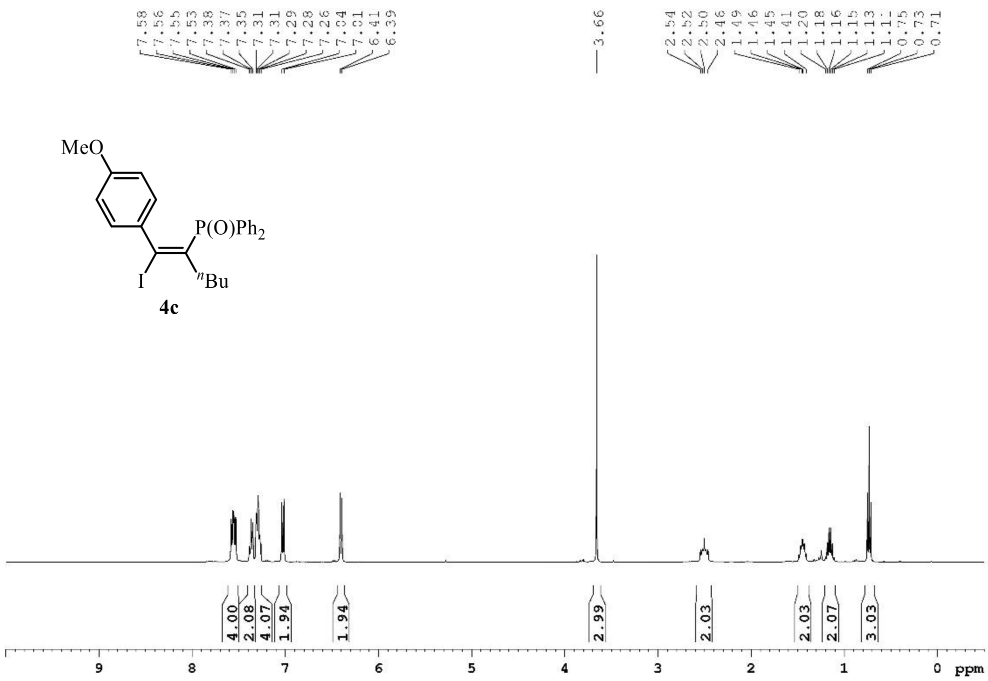

^{13}C NMR (101 MHz, CDCl$_3$) of 4c

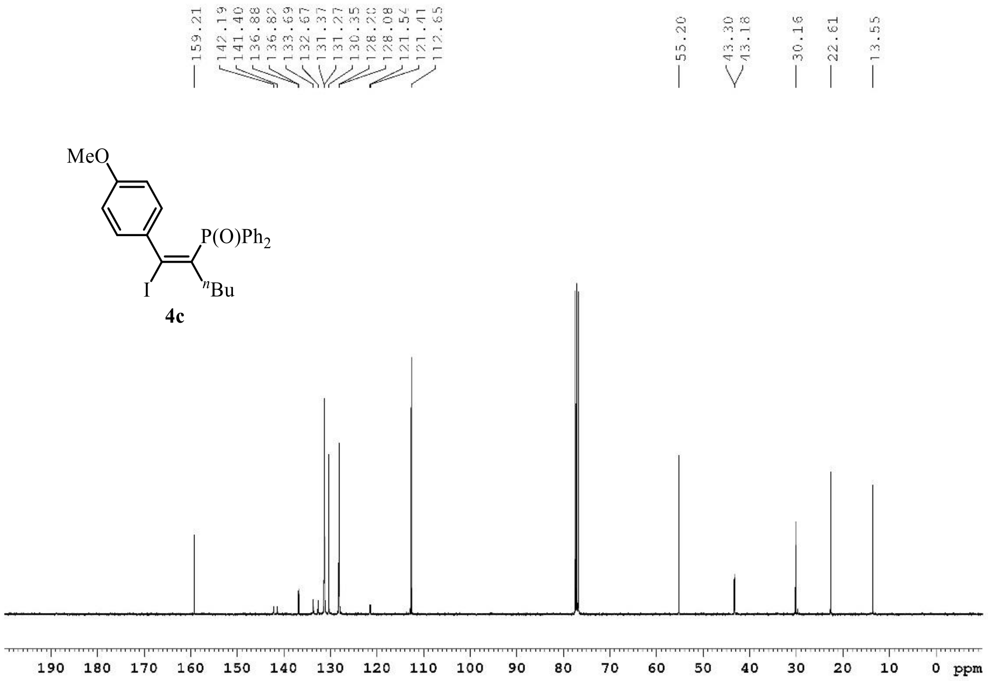

^{31}P NMR (162 MHz, CDCl$_3$) of 4c

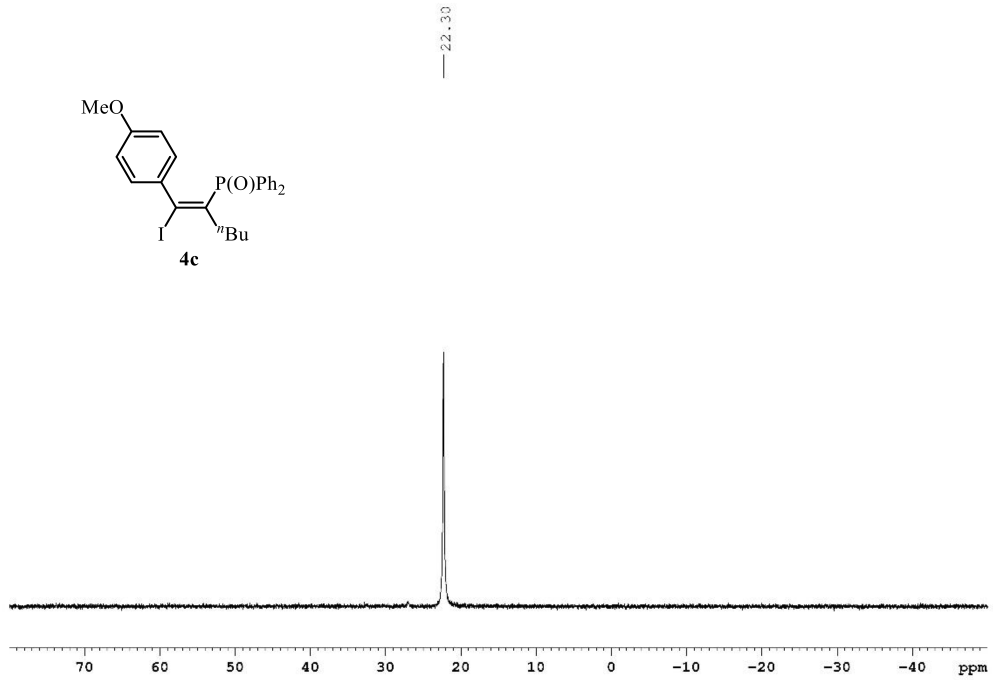

^{1}H NMR (400 MHz, CDCl$_3$) of 4d

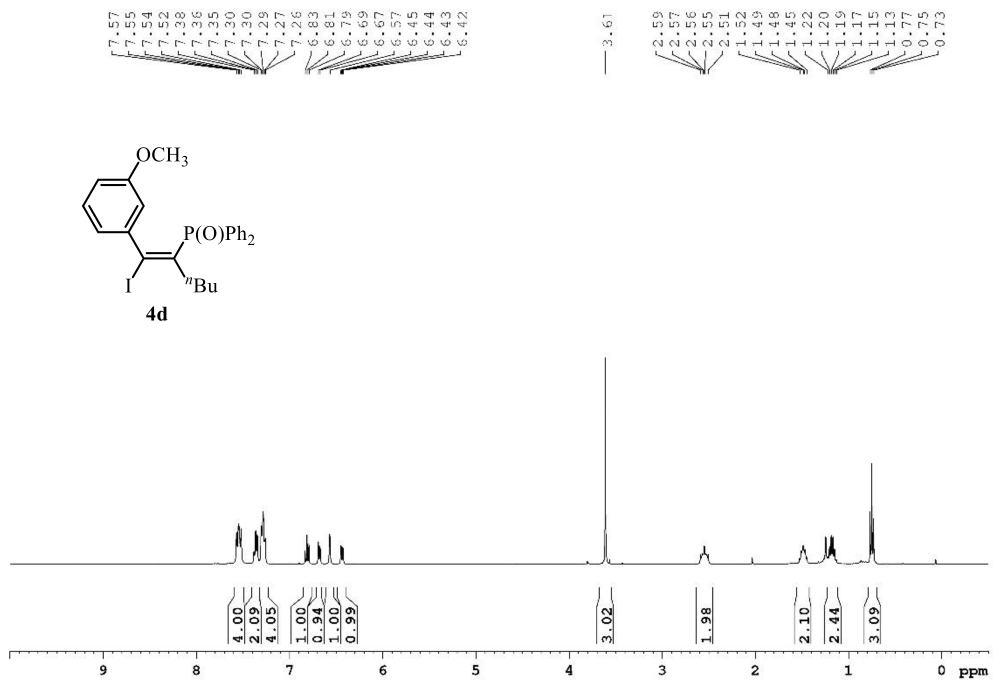

^{13}C NMR (101 MHz, $CDCl_3$) of 4d

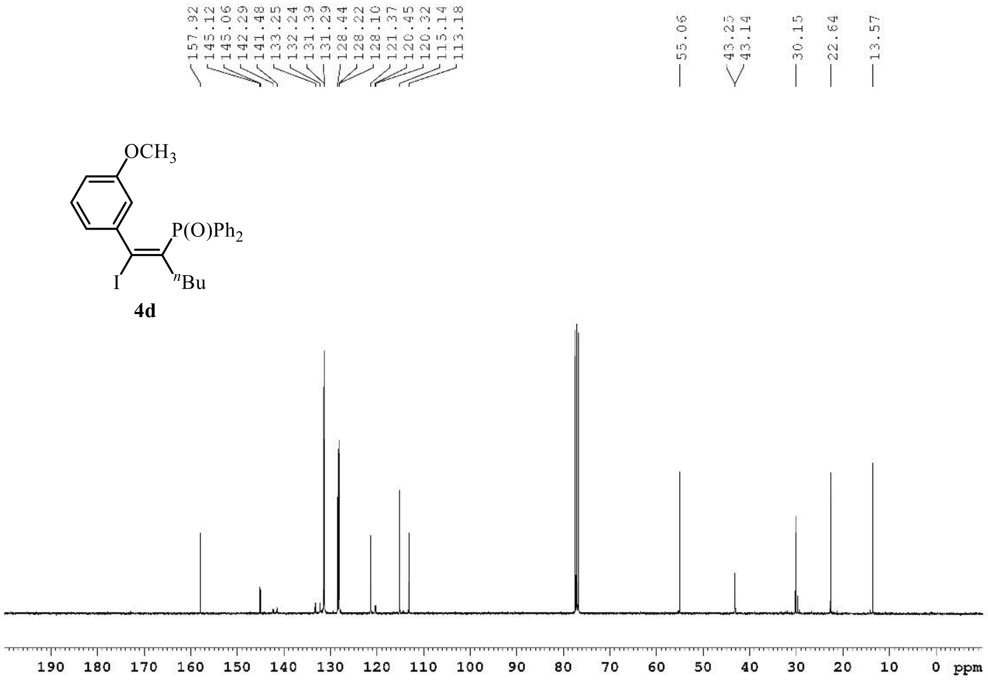

^{31}P NMR (162 MHz, $CDCl_3$) of 4d

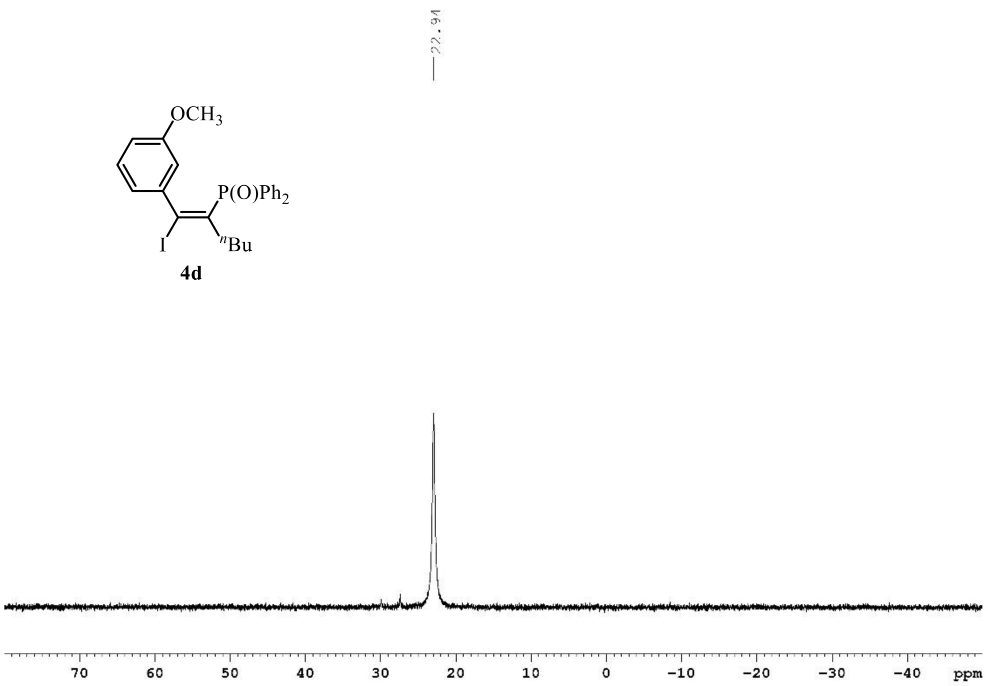

^{1}H NMR (400 MHz, $CDCl_3$) of 4e

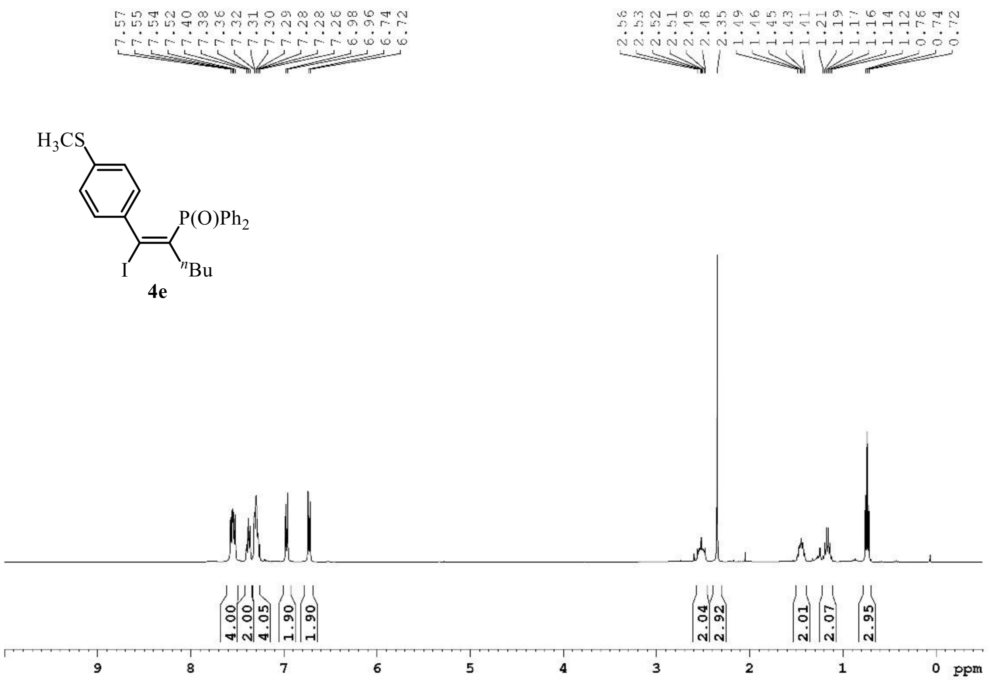

^{13}C NMR (101 MHz, $CDCl_3$) of 4e

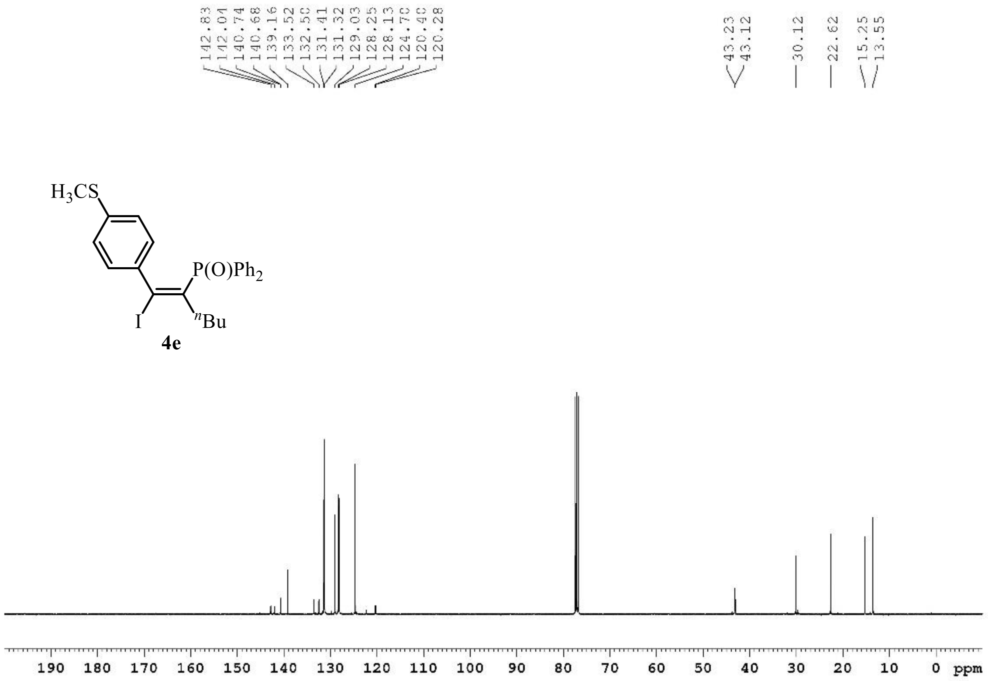

^{31}P NMR (162 MHz, CDCl$_3$) of 4e

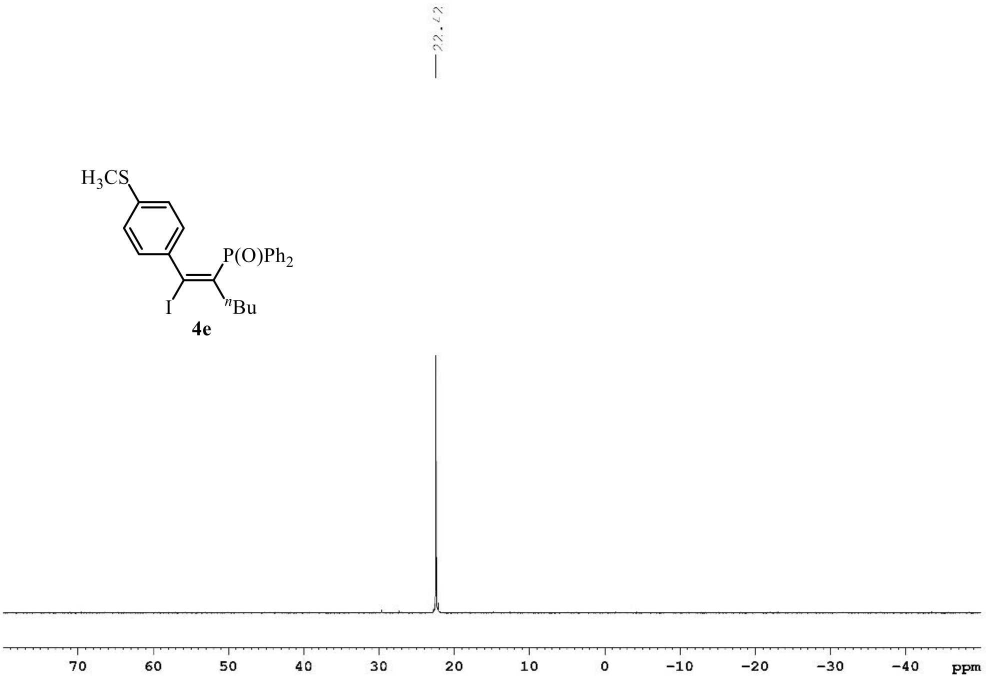

^{1}H NMR (400 MHz, CDCl$_3$) of 4f

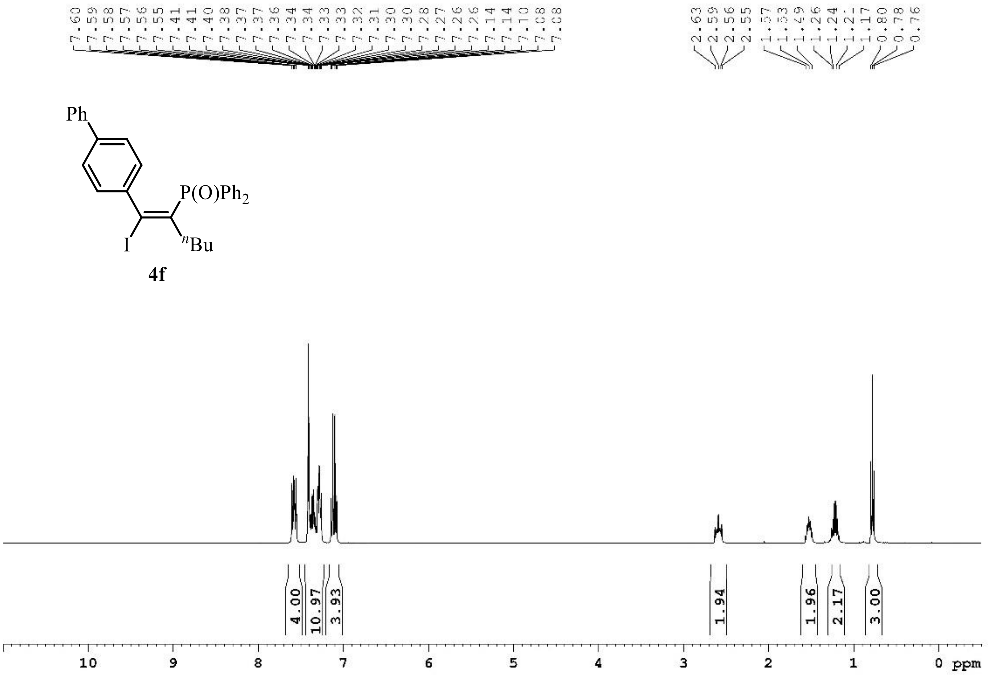

^{13}C NMR (101 MHz, $CDCl_3$) of 4f

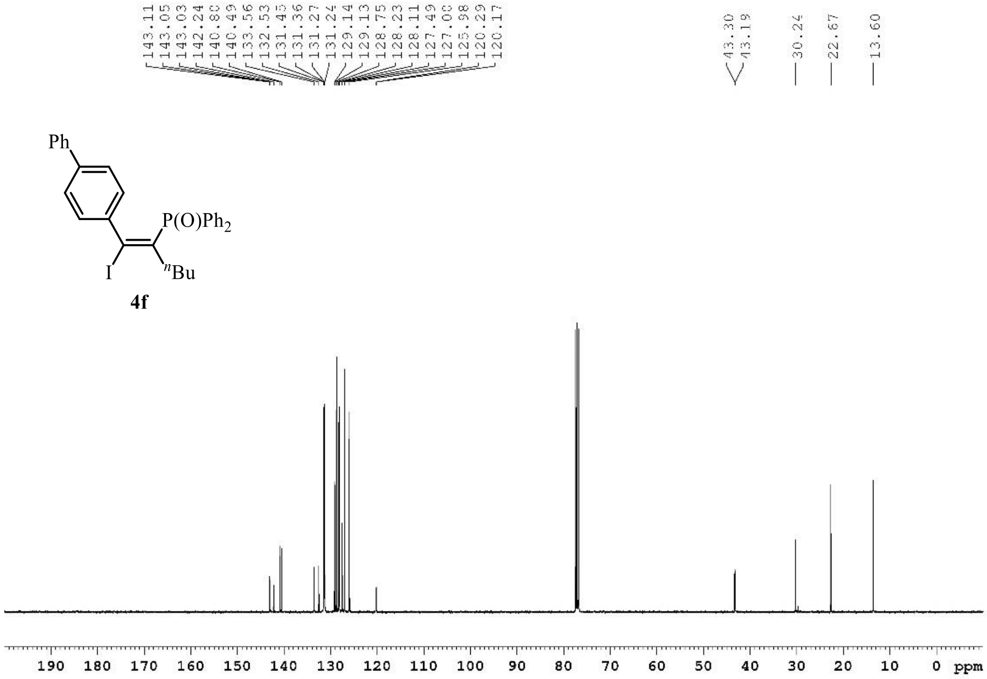

^{31}P NMR (162 MHz, $CDCl_3$) of 4f

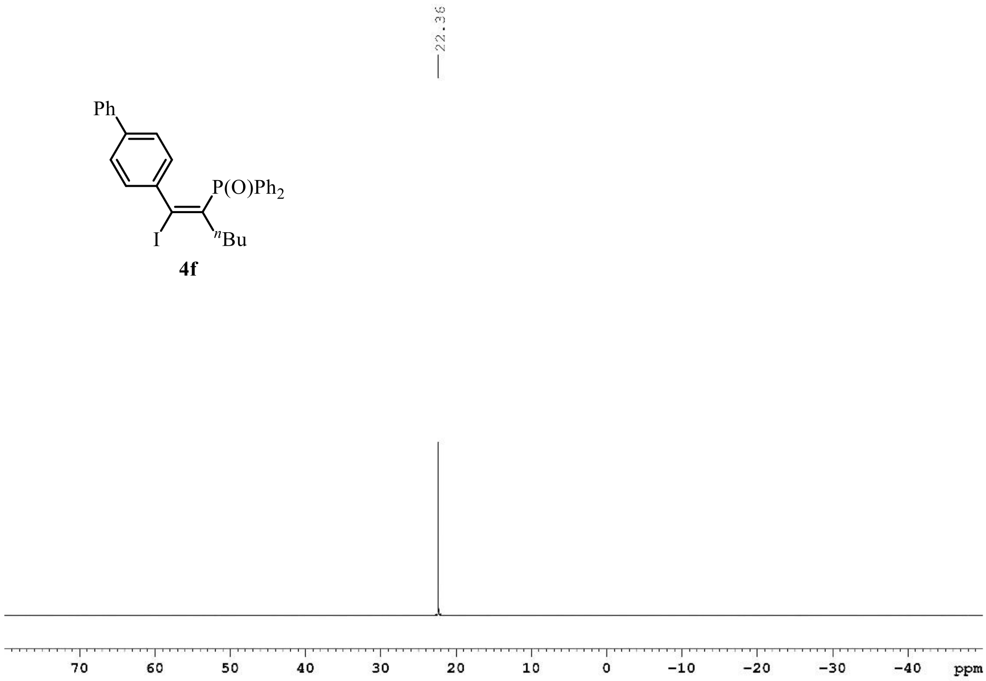

^{1}H NMR (400 MHz, CDCl$_3$) of 4g

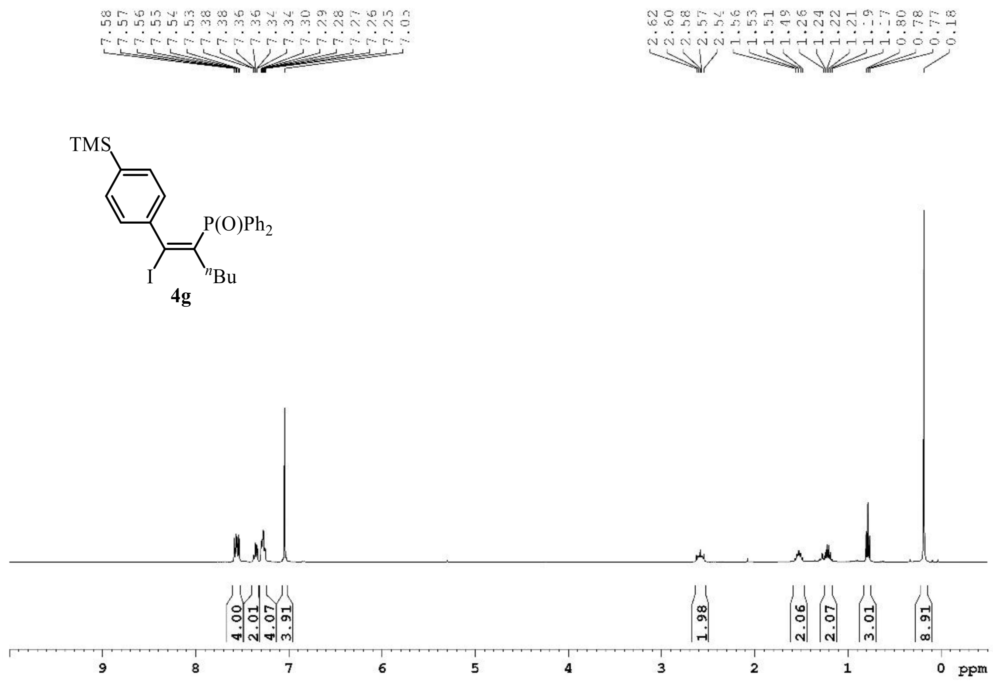

^{13}C NMR (101 MHz, CDCl$_3$) of 4g

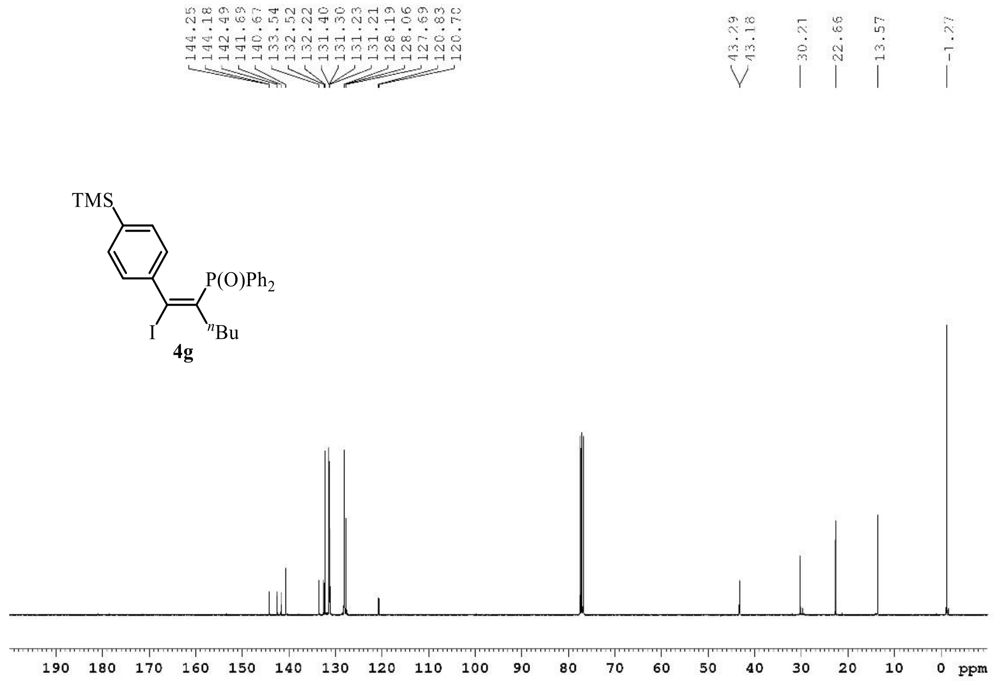

^{31}P NMR (162 MHz, CDCl$_3$) of 4g

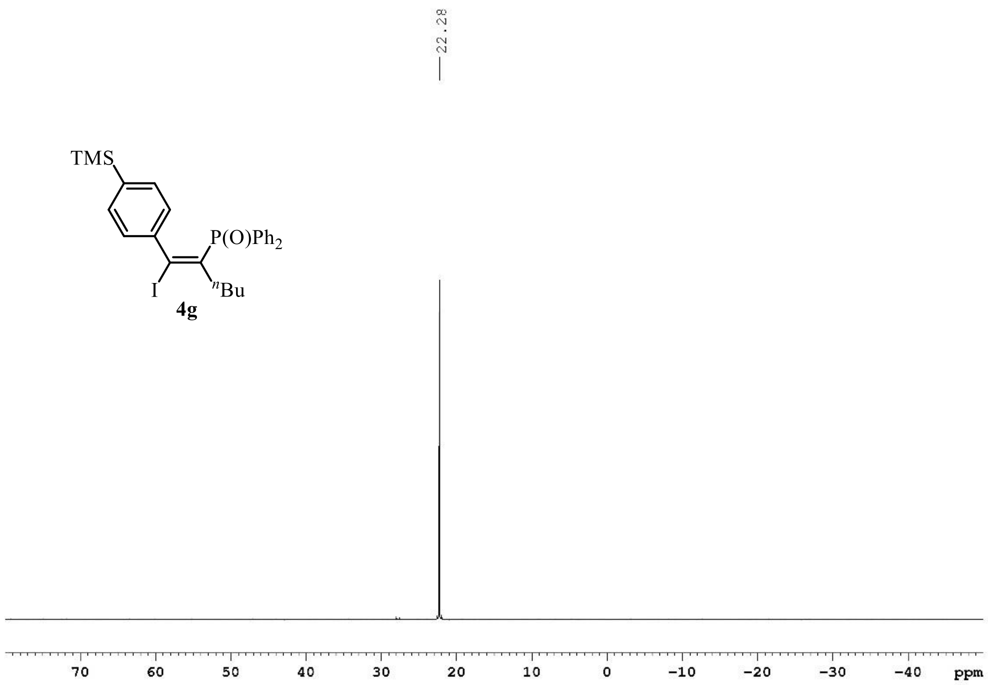

^{1}H NMR (400 MHz, CDCl$_3$) of 4h

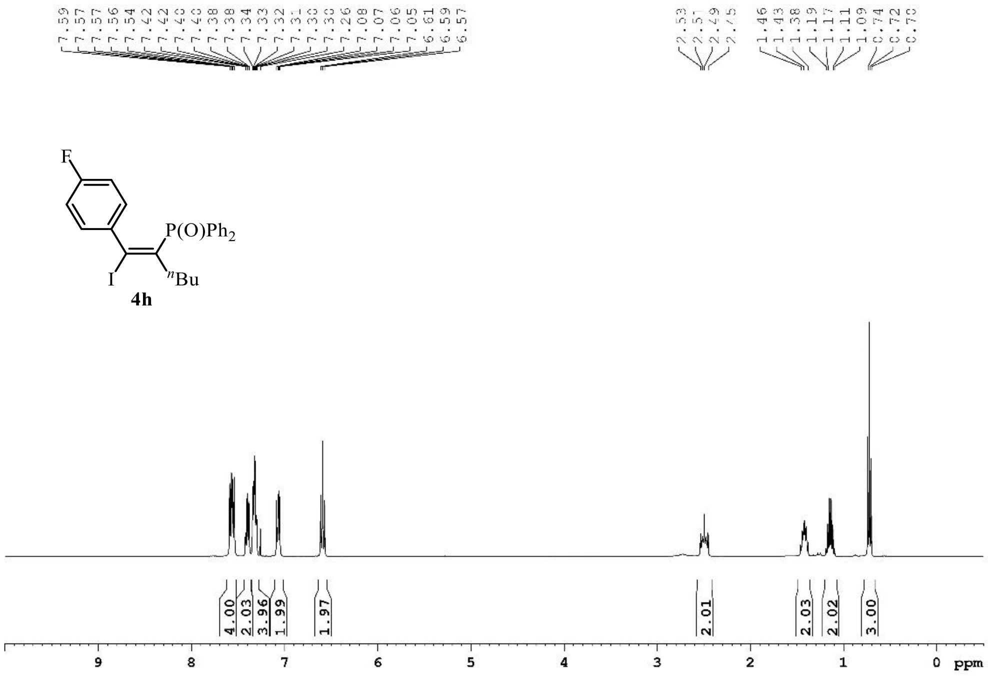

^{13}C NMR (101 MHz, $CDCl_3$) of 4h

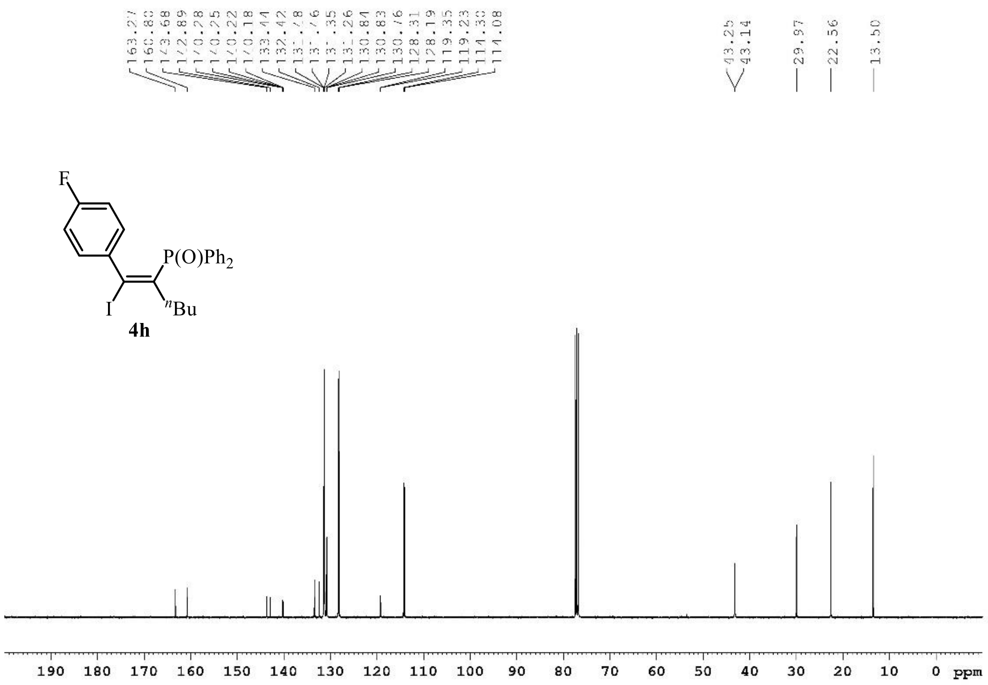

^{31}P NMR (162 MHz, $CDCl_3$) of 4h

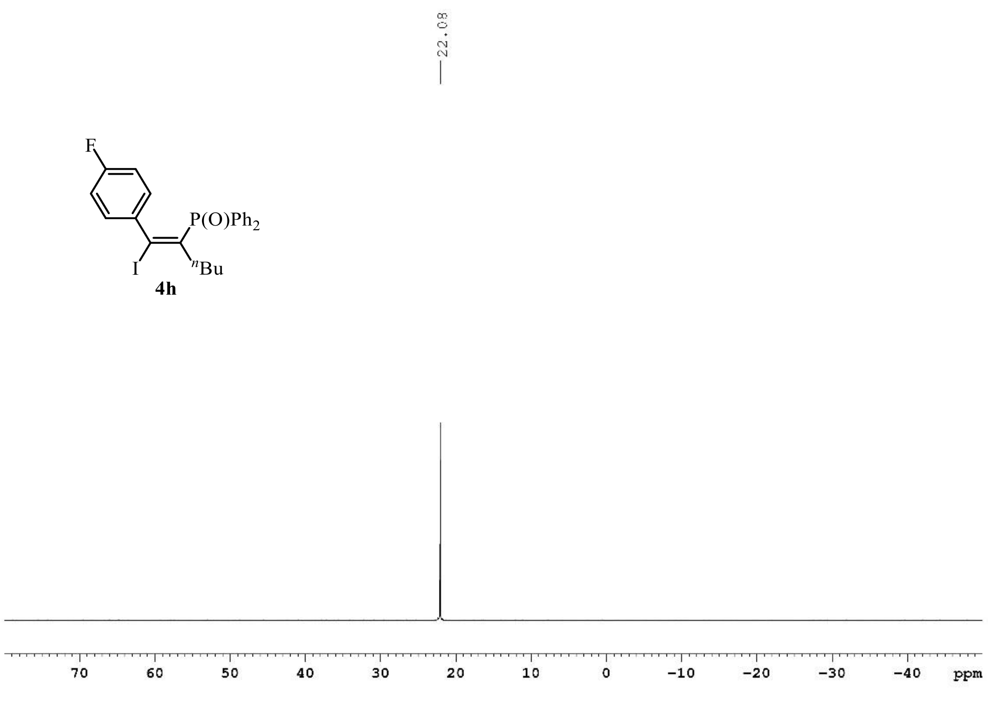

^{1}H NMR (400 MHz, CDCl$_3$) of 4i

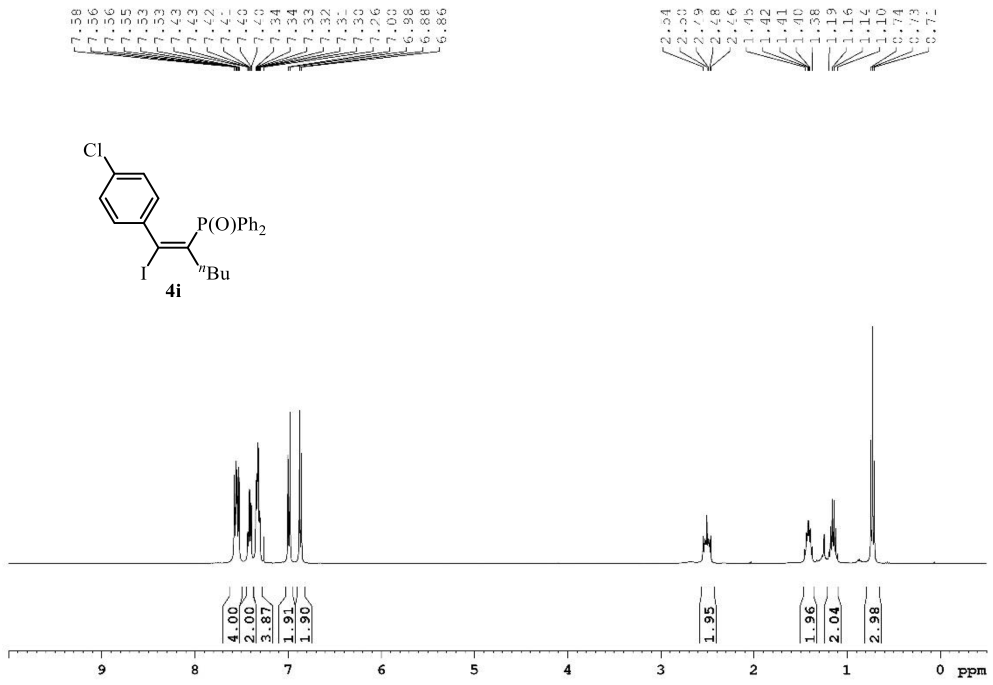

^{13}C NMR (101 MHz, CDCl$_3$) of 4i

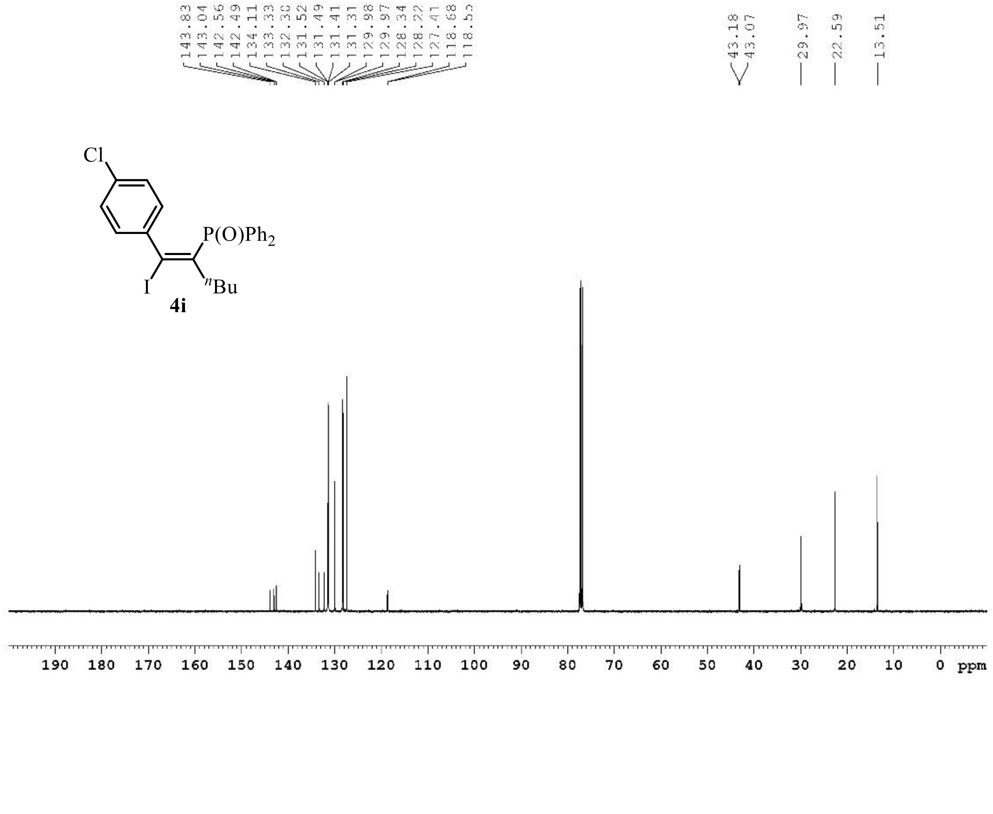

^{31}P NMR (162 MHz, $CDCl_3$) of 4i

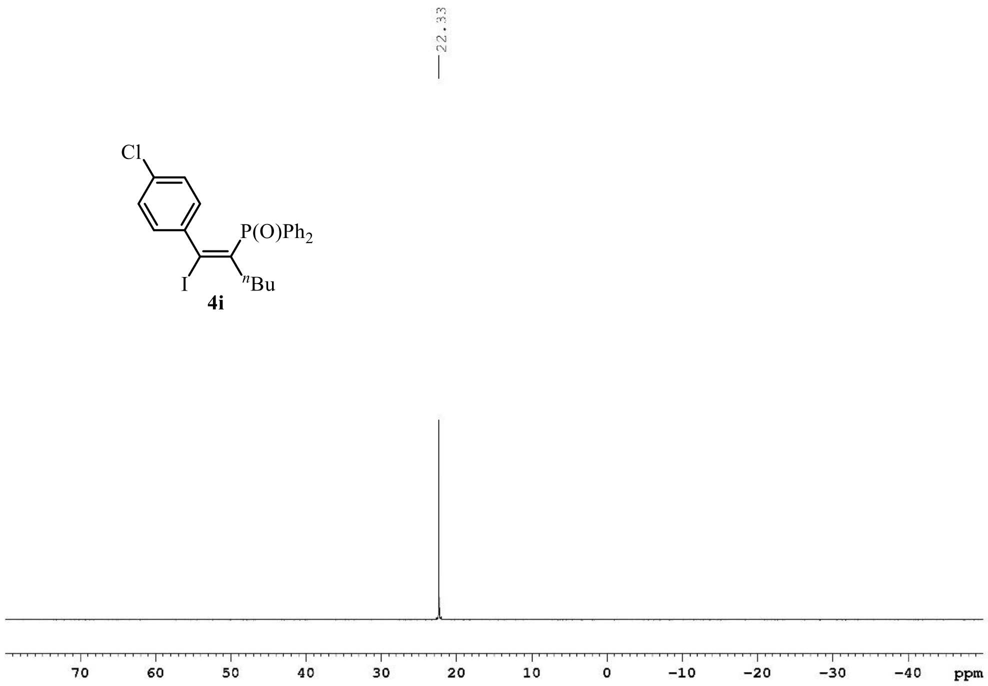

^{1}H NMR (400 MHz, $CDCl_3$) of 4j

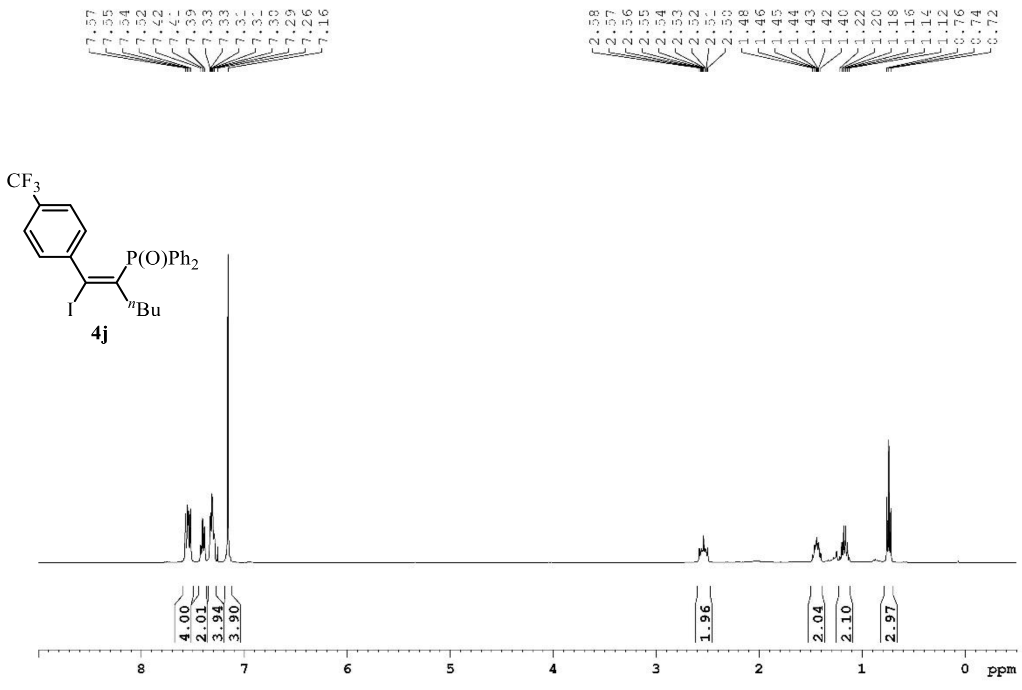

^{13}C NMR (101 MHz, CDCl3) of 4j

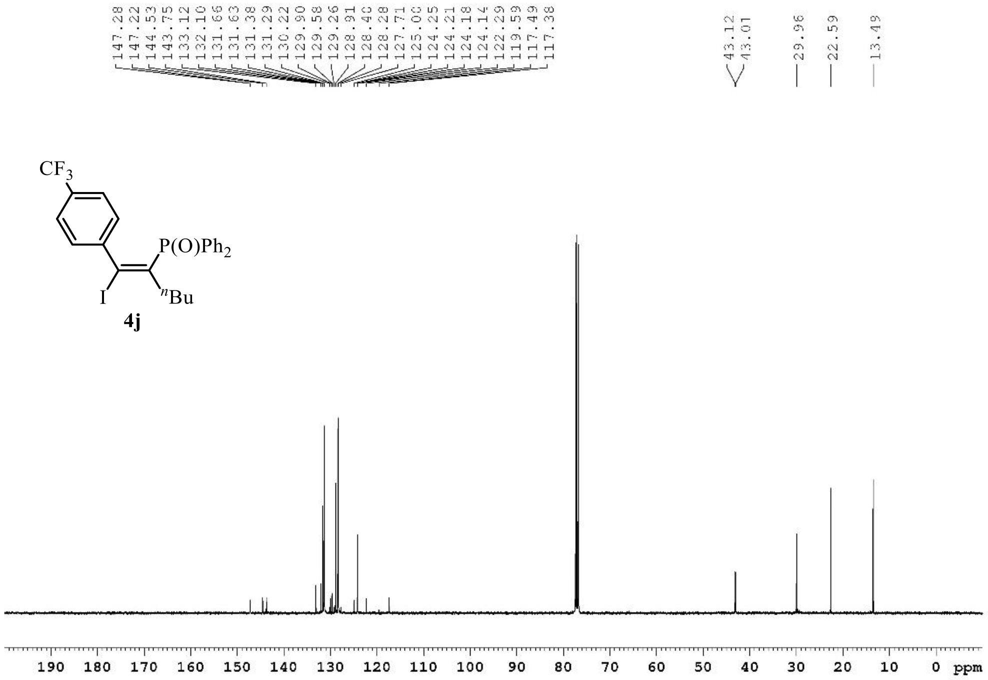

^{31}P NMR (162 MHz, CDCl3) of 4j

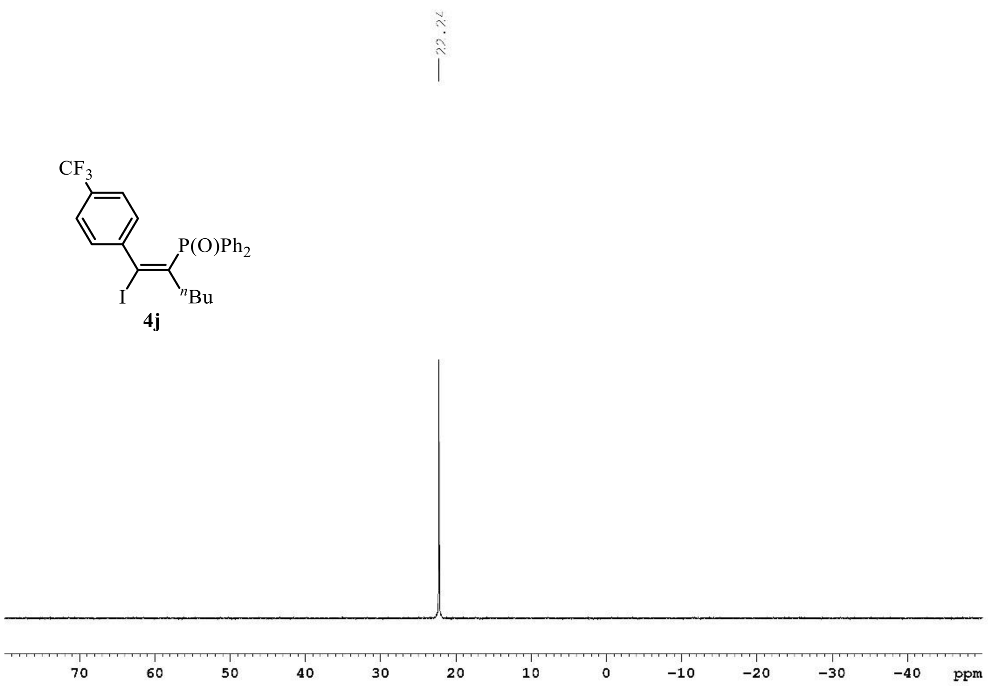

^{1}H NMR (400 MHz, CDCl$_3$) of 4k

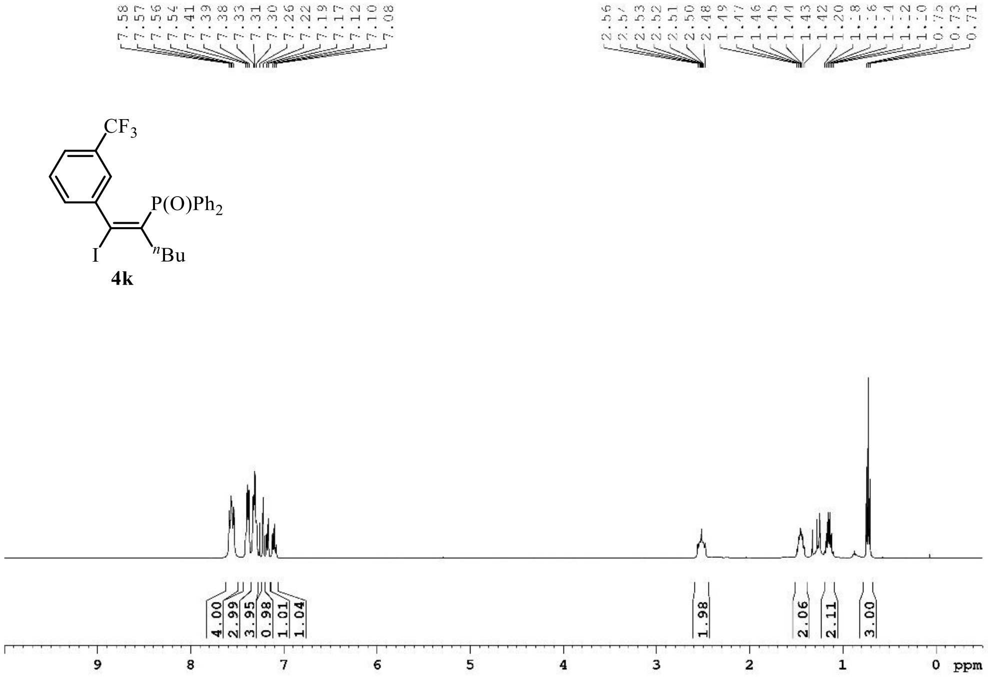

^{13}C NMR (101 MHz, CDCl$_3$) of 4k

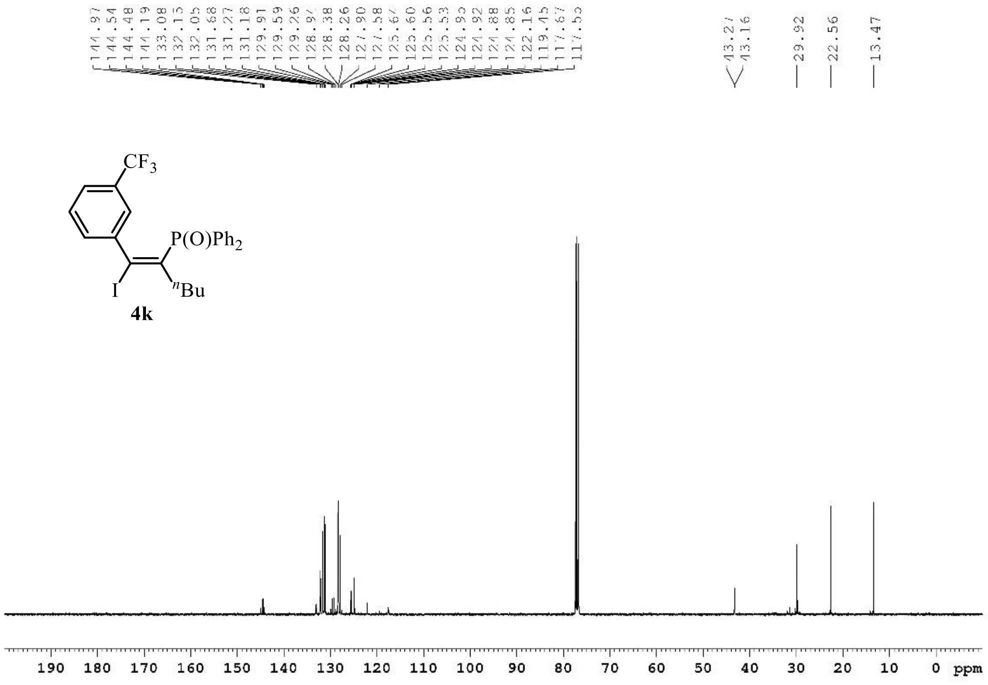

^{31}P NMR (162 MHz, $CDCl_3$) of 4k

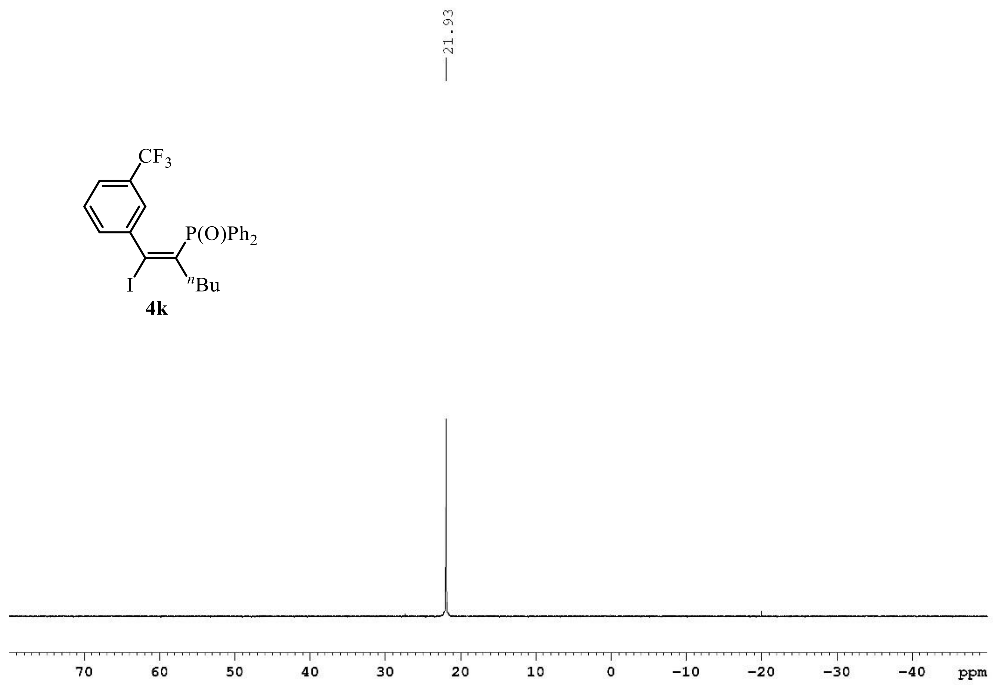

^{1}H NMR (400 MHz, $CDCl_3$) of 4l

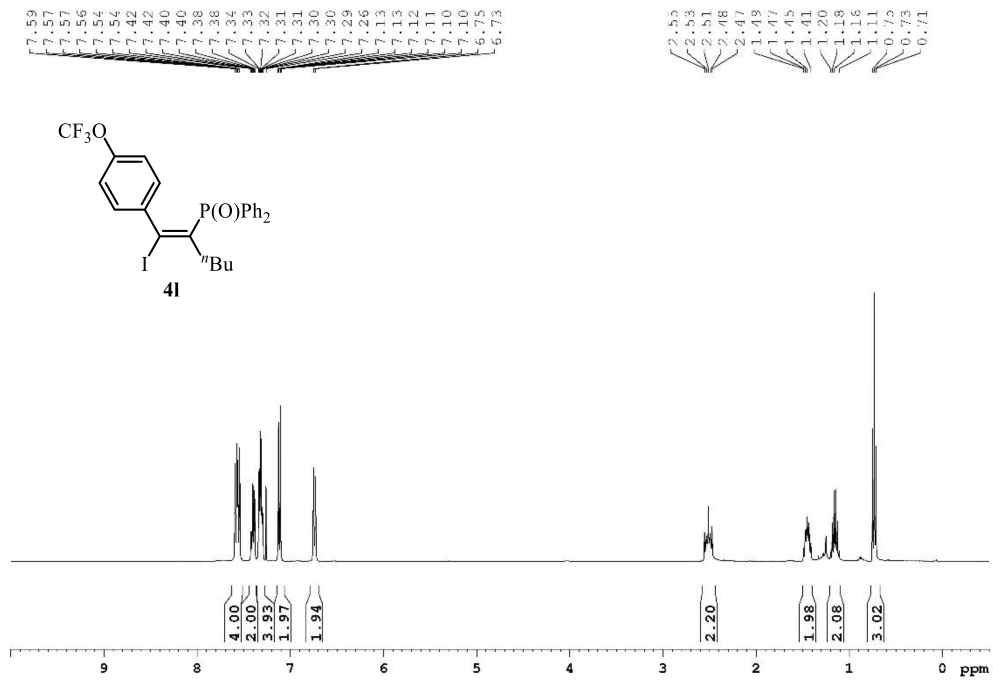

^{13}C NMR (101 MHz, $CDCl_3$) of 4l

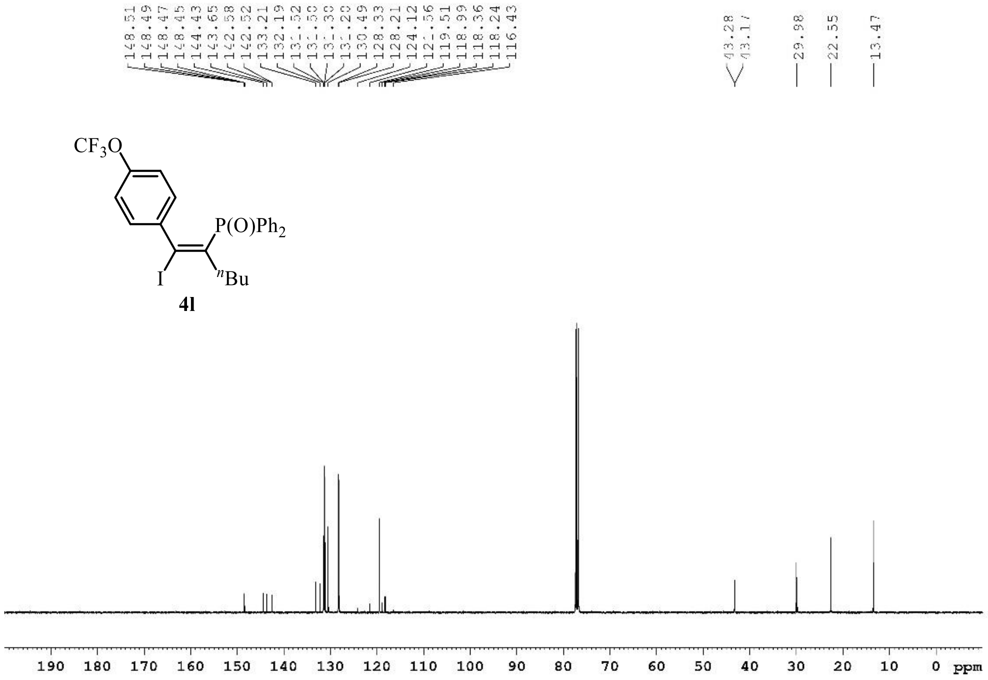

^{31}P NMR (162 MHz, $CDCl_3$) of 4l

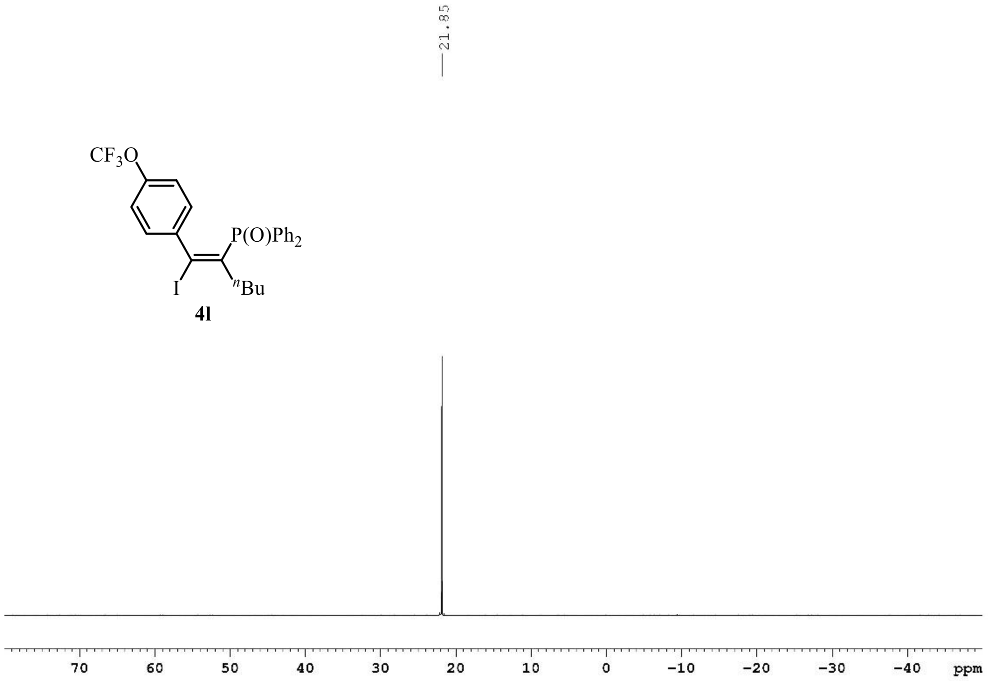

^{1}H NMR (400 MHz, CDCl$_3$) of 4m

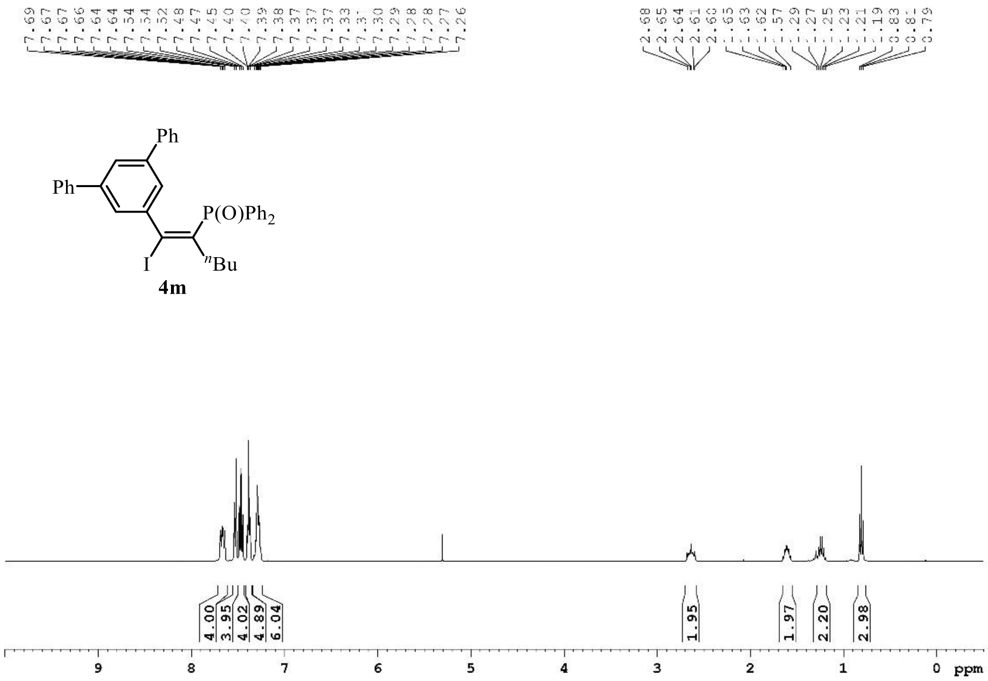

^{13}C NMR (101 MHz, CDCl$_3$) of 4m

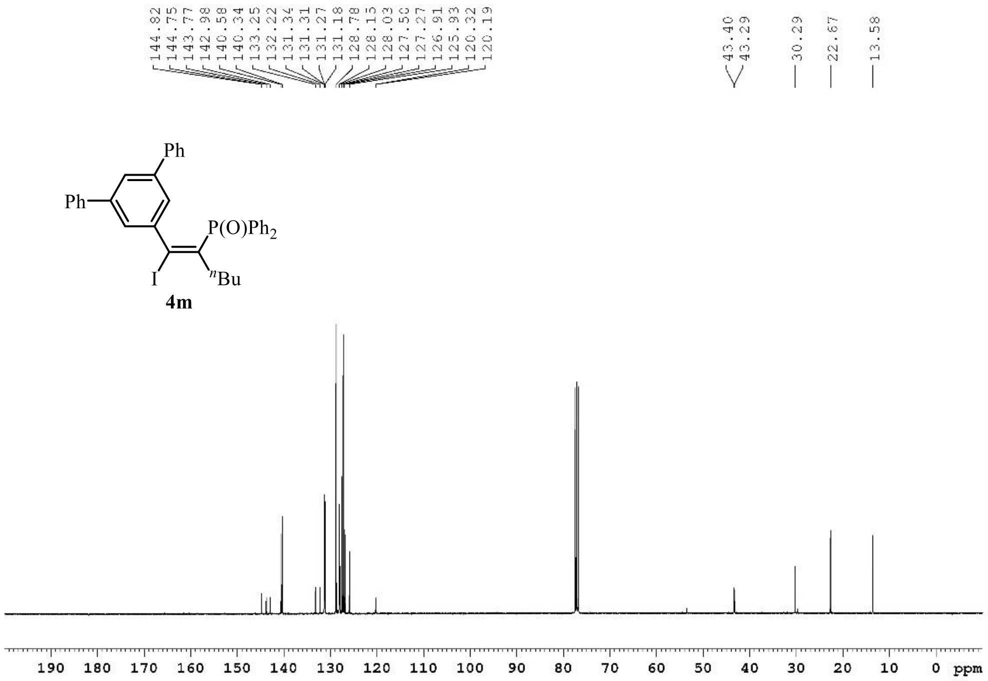

^{31}P NMR (162 MHz, $CDCl_3$) of 4m

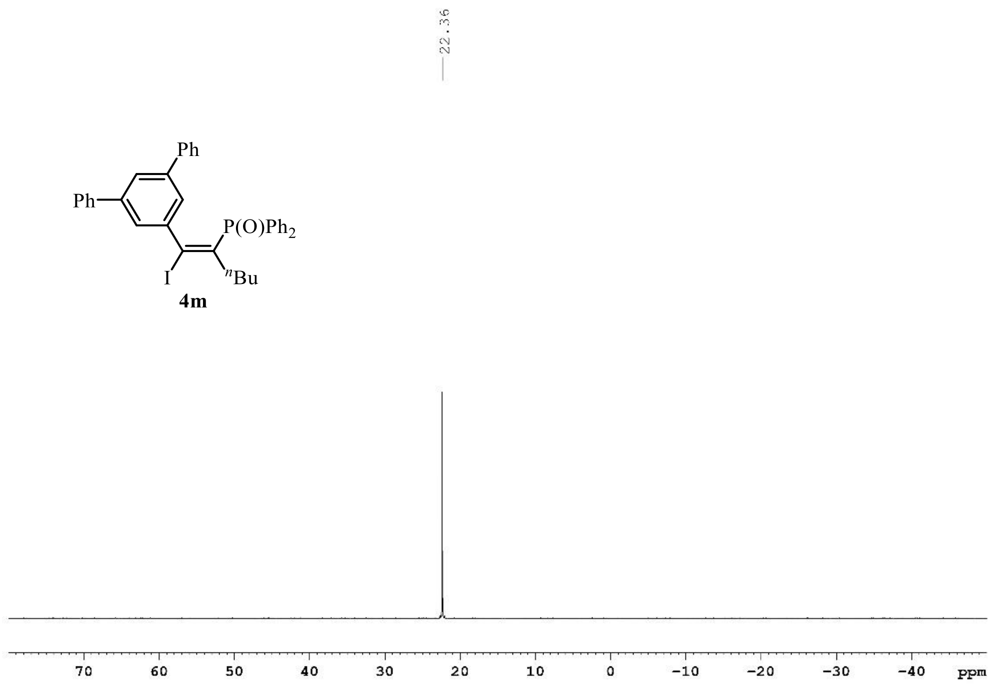

^{1}H NMR (400 MHz, $CDCl_3$) of 4n

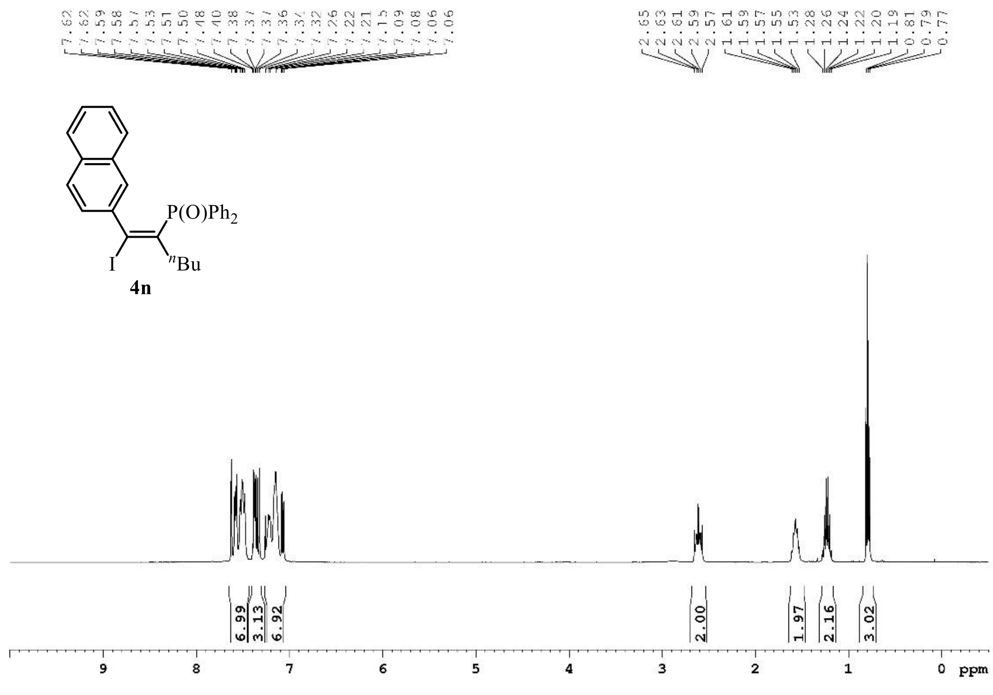

^{13}C NMR (101 MHz, $CDCl_3$) of 4n

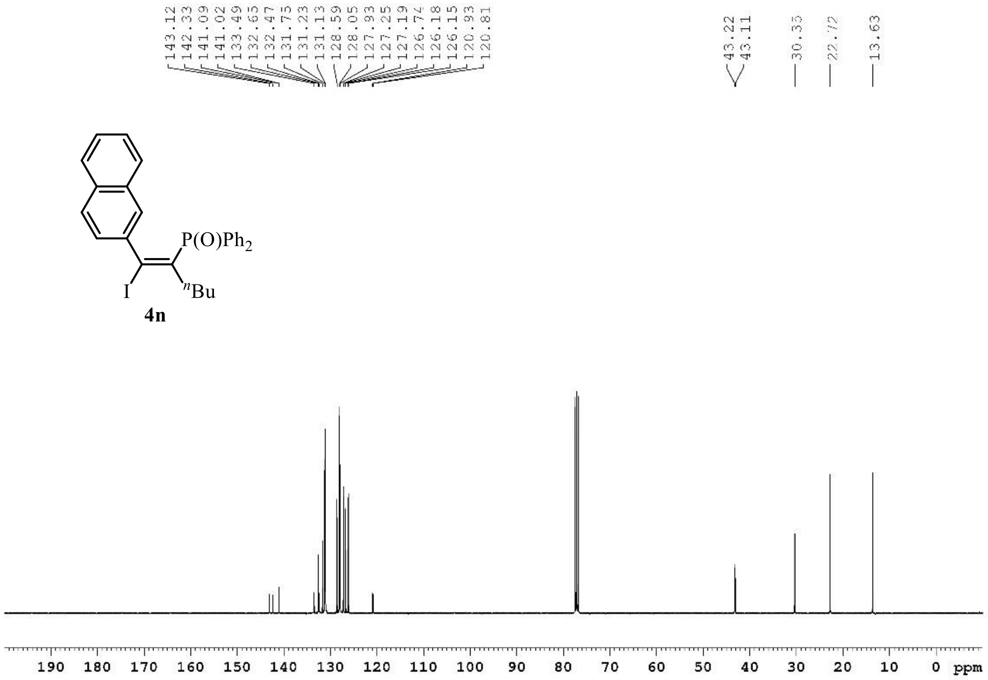

^{31}P NMR (162 MHz, $CDCl_3$) of 4n

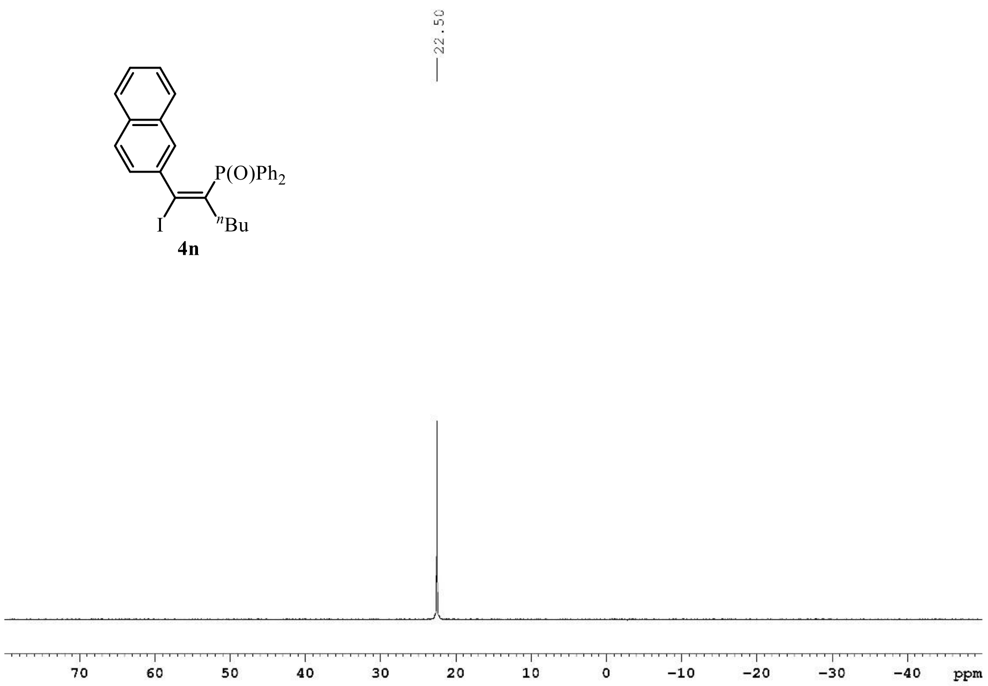

^{1}H NMR (400 MHz, CDCl$_3$) of 4o

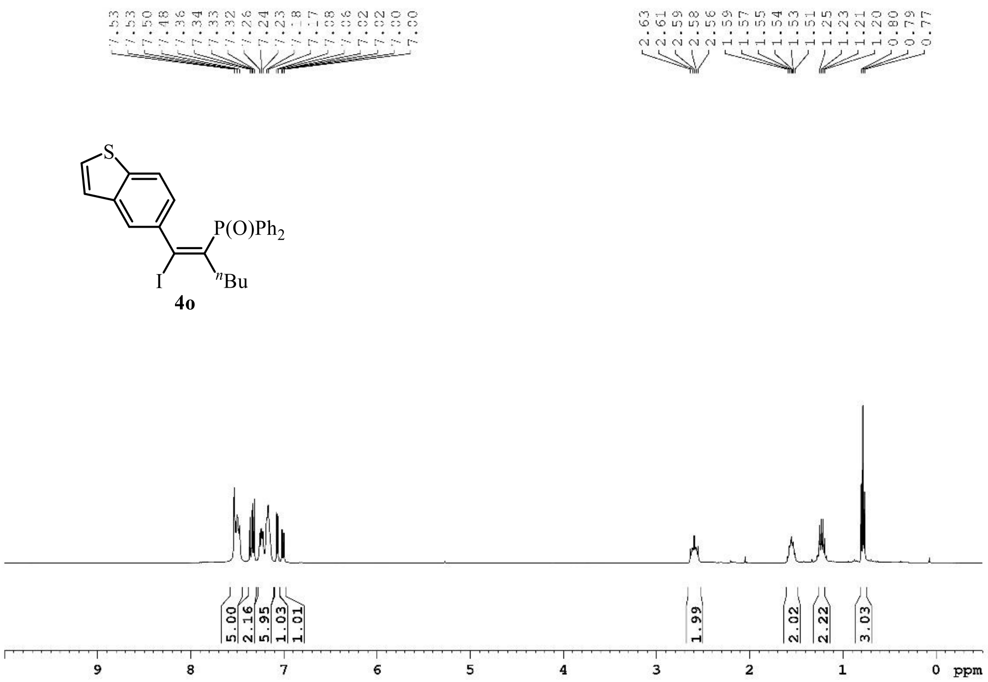

^{13}C NMR (101 MHz, CDCl$_3$) of 4o

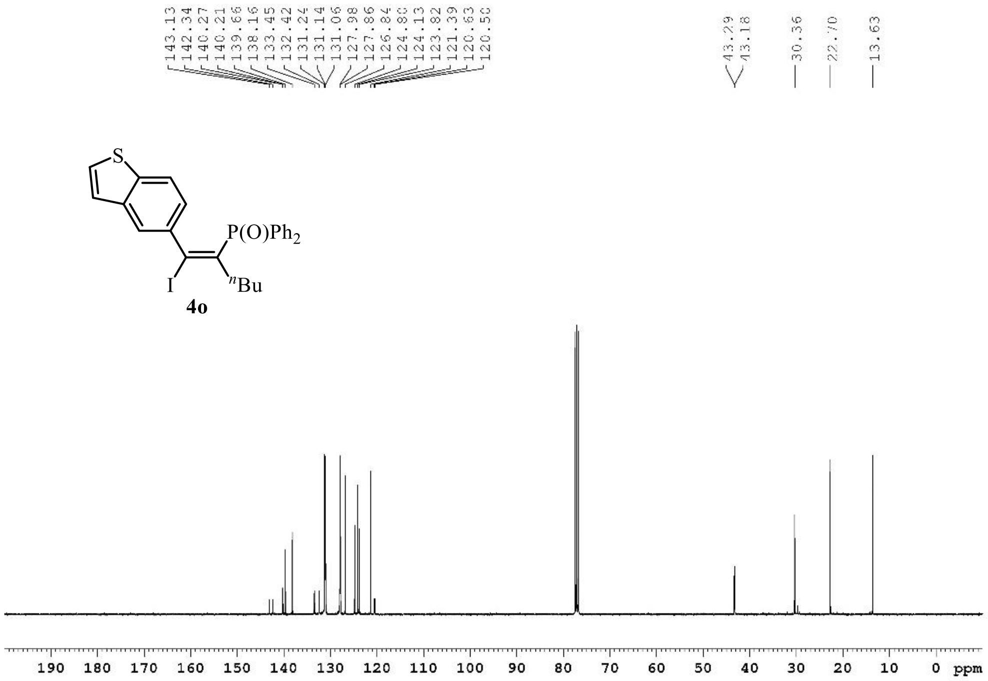

^{31}P NMR (162 MHz, CDCl$_3$) of 4o

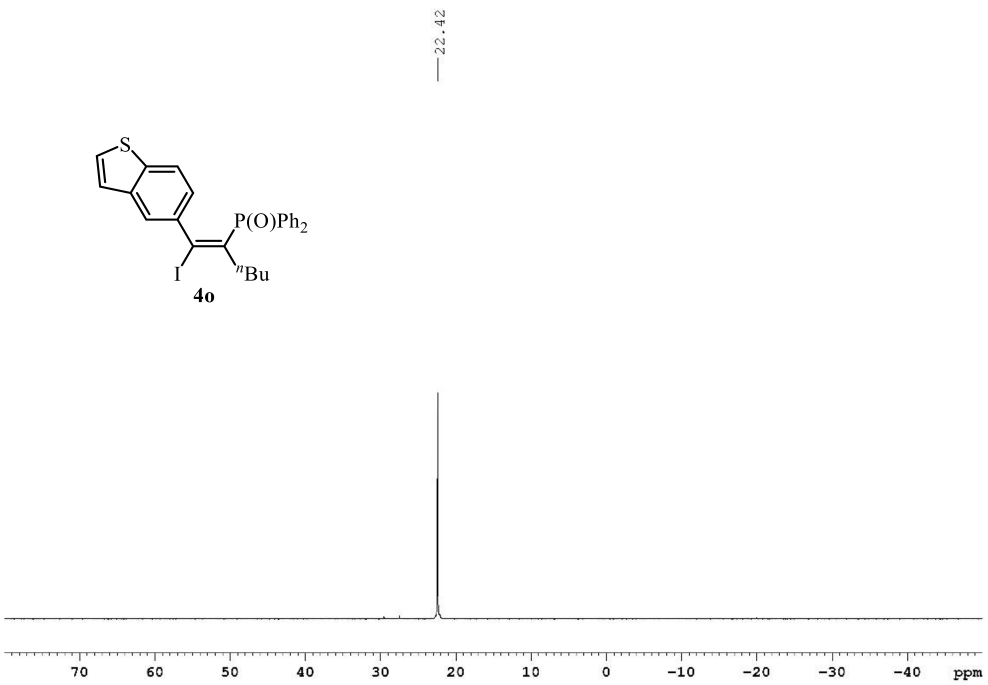

^{1}H NMR (400 MHz, CDCl$_3$) of 4p

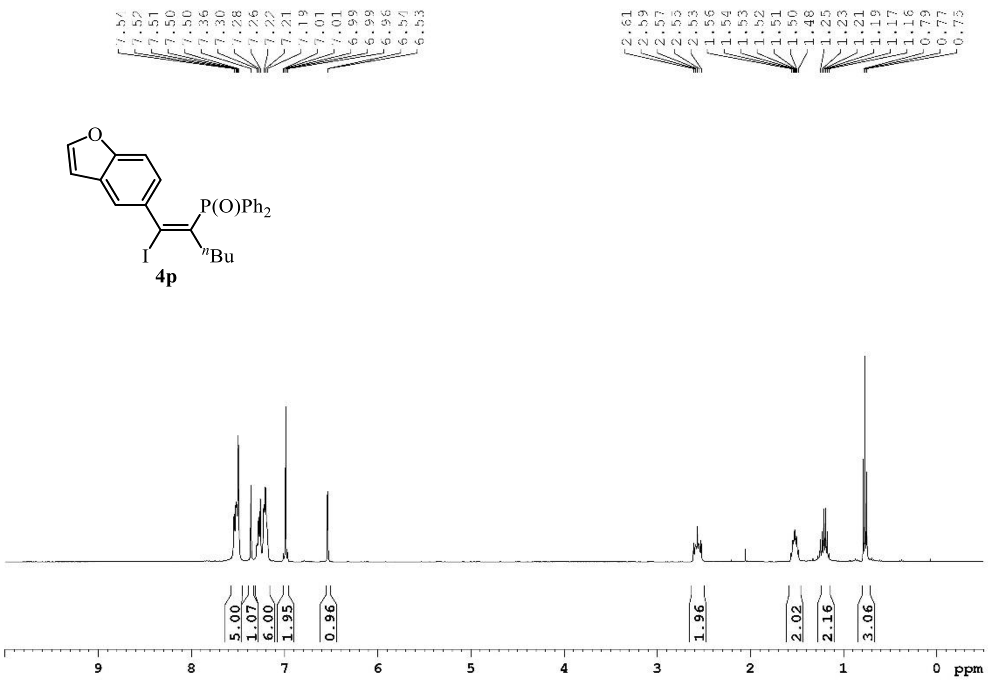

^{13}C NMR (101 MHz, $CDCl_3$) of 4p

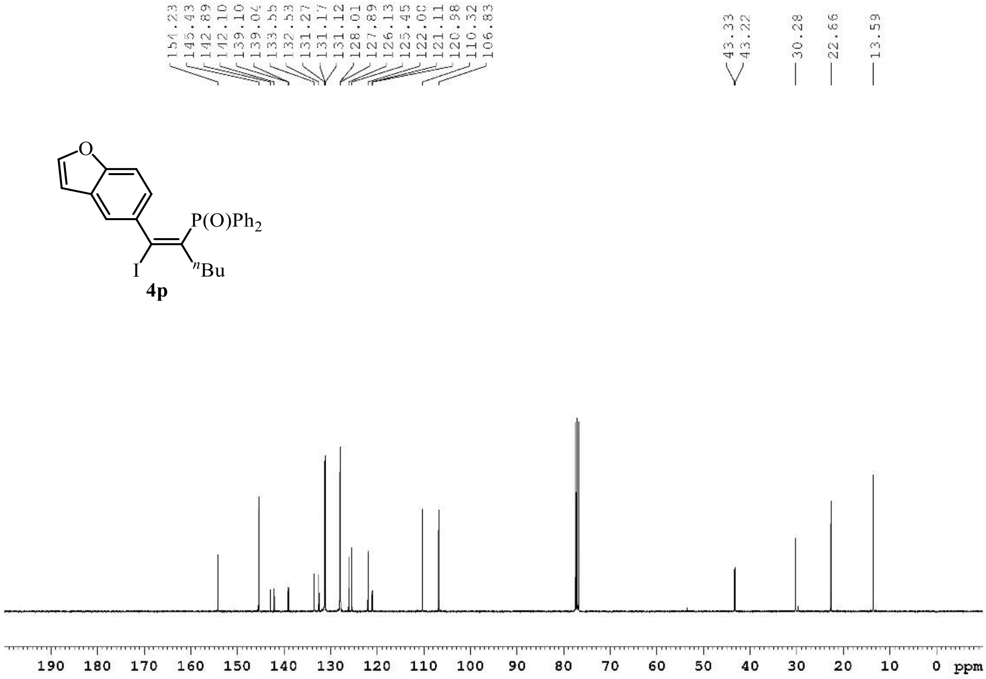

^{31}P NMR (162 MHz, $CDCl_3$) of 4p

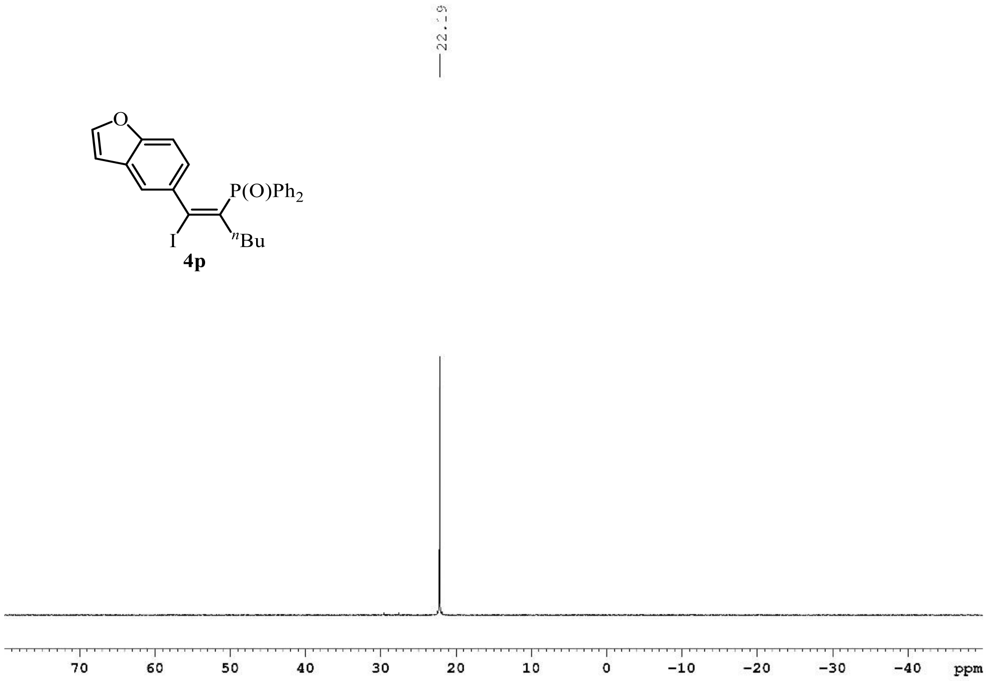

^{1}H NMR (400 MHz, CDCl$_3$) of 4q

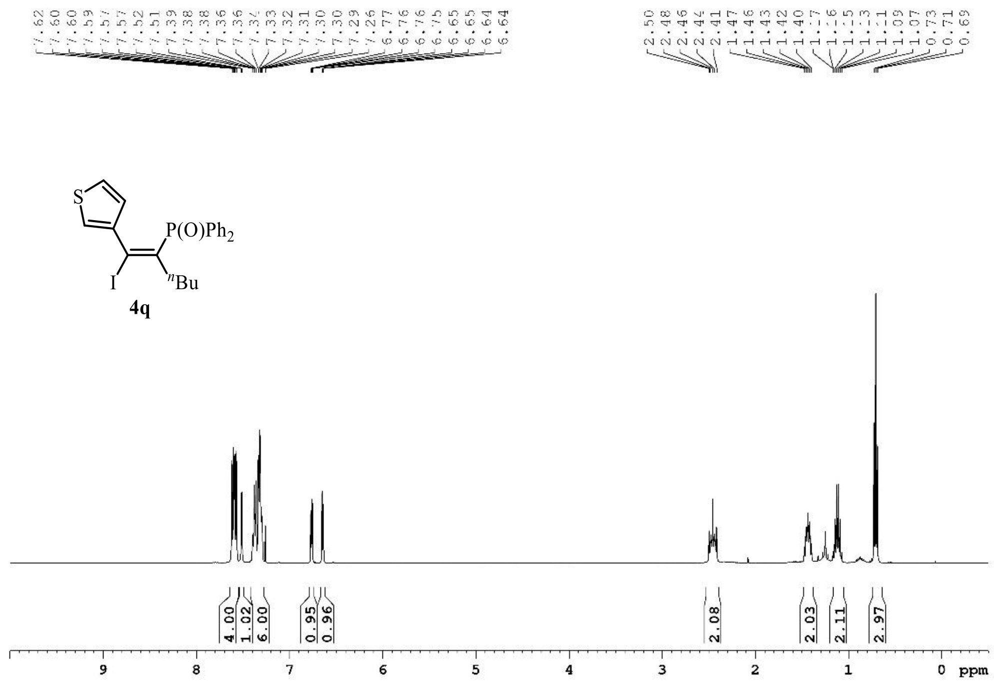

^{13}C NMR (101 MHz, CDCl$_3$) of 4q

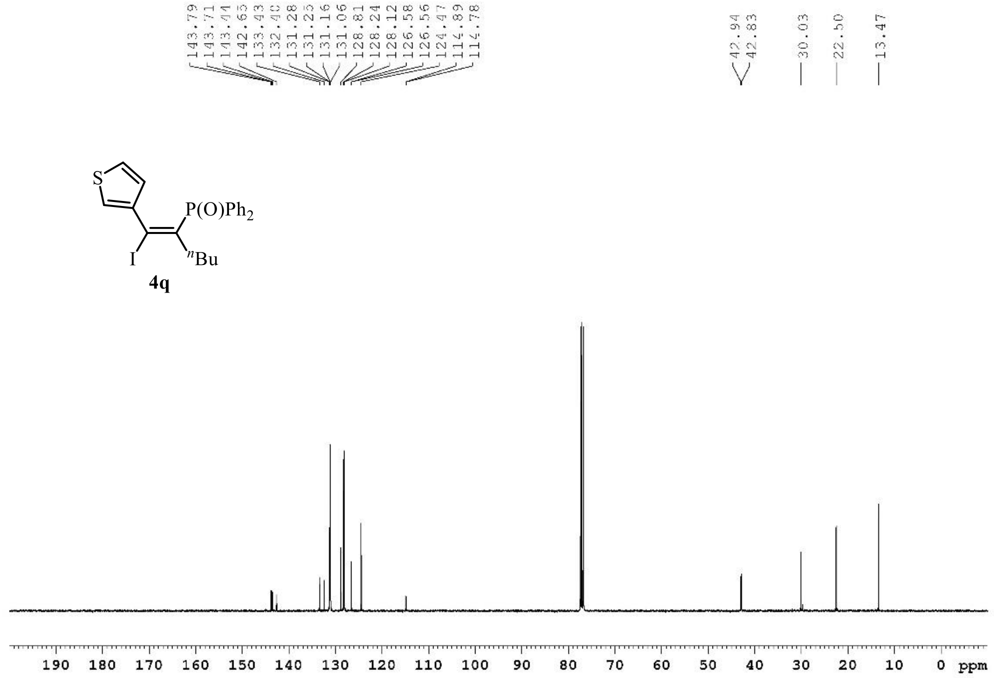

^{31}P NMR (162 MHz, CDCl$_3$) of 4q

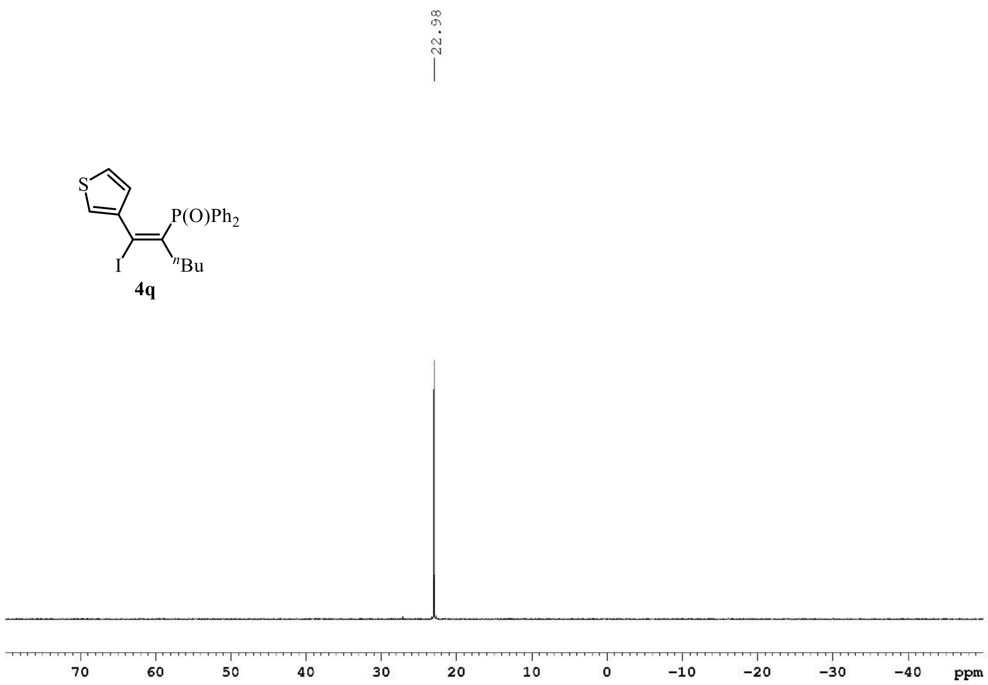

^{1}H NMR (400 MHz, CDCl$_3$) of 4r

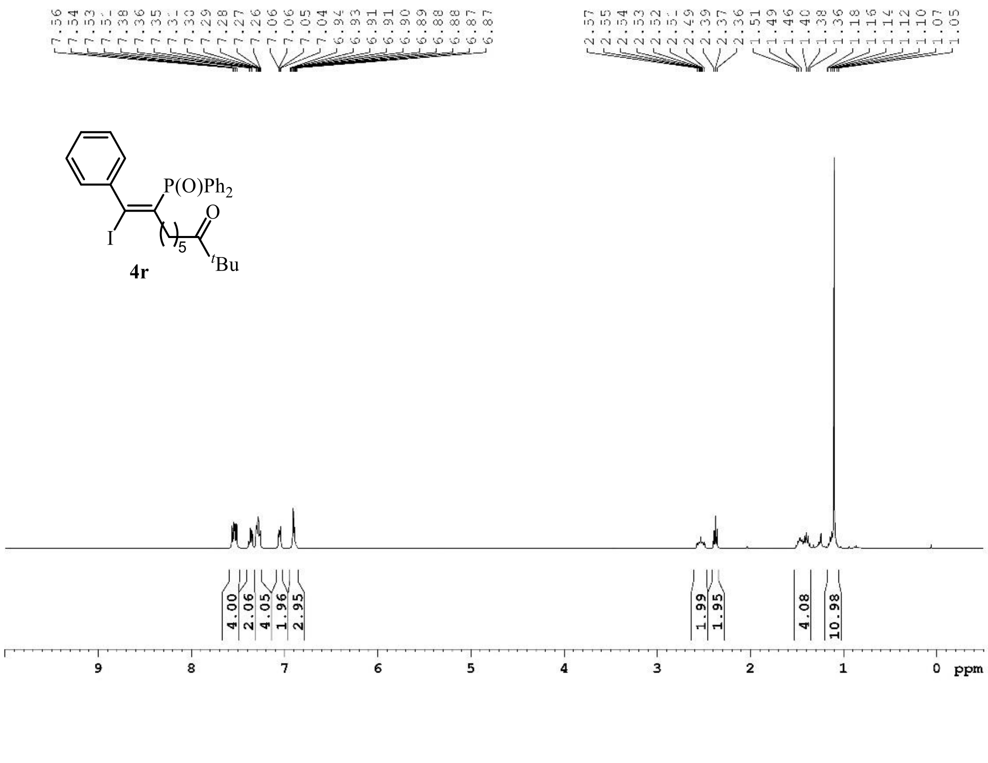

^{13}C NMR (101 MHz, CDCl$_3$) of 4r

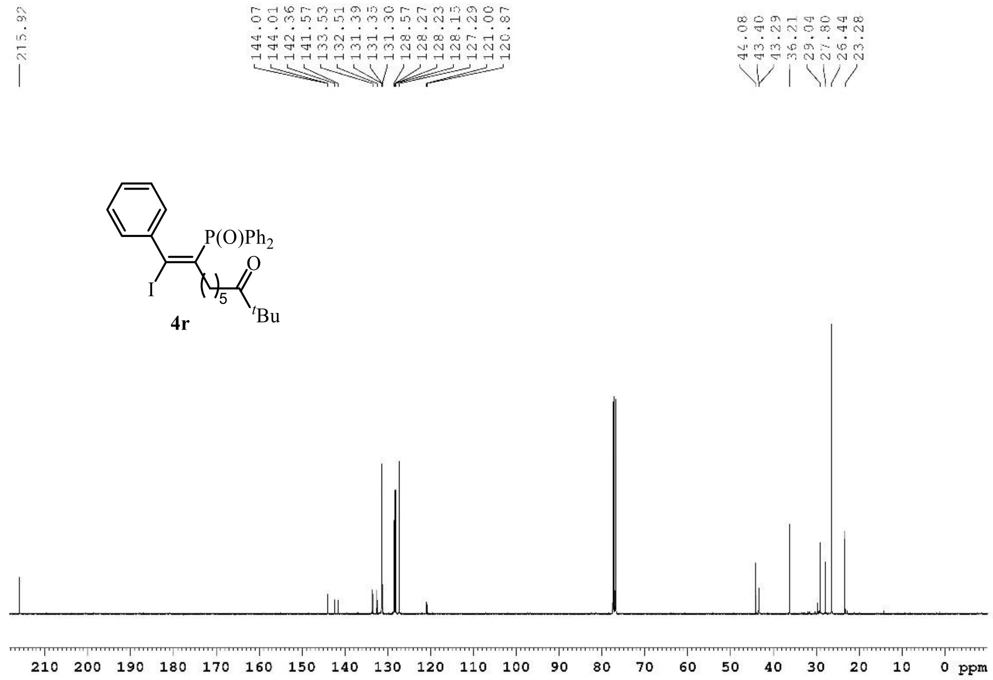

^{31}P NMR (162 MHz, CDCl$_3$) of 4r

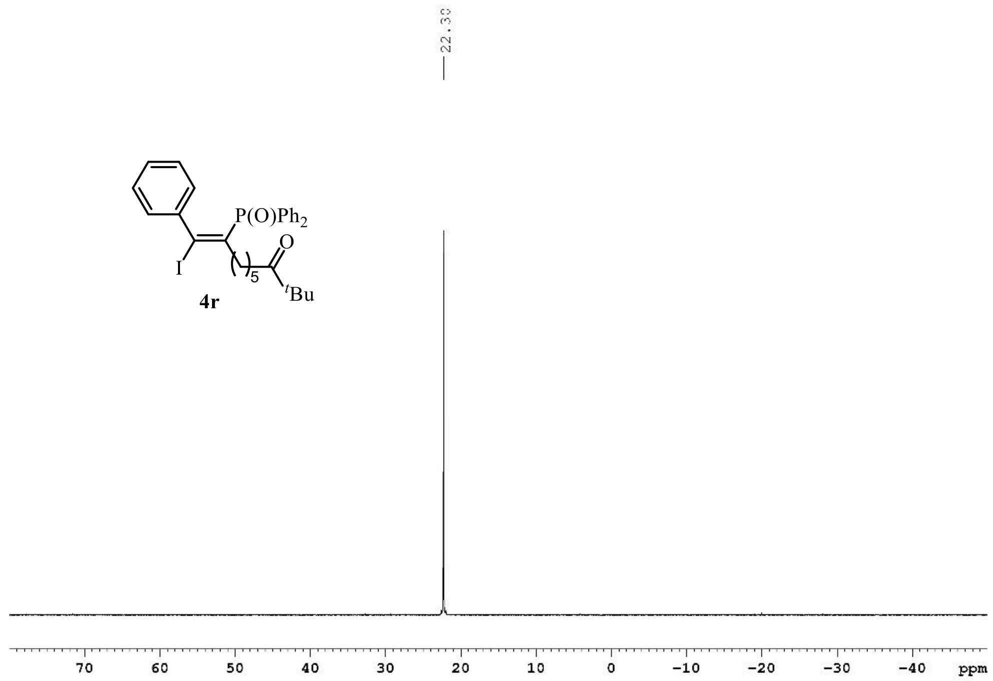

^{1}H NMR (400 MHz, $CDCl_3$) of 4s

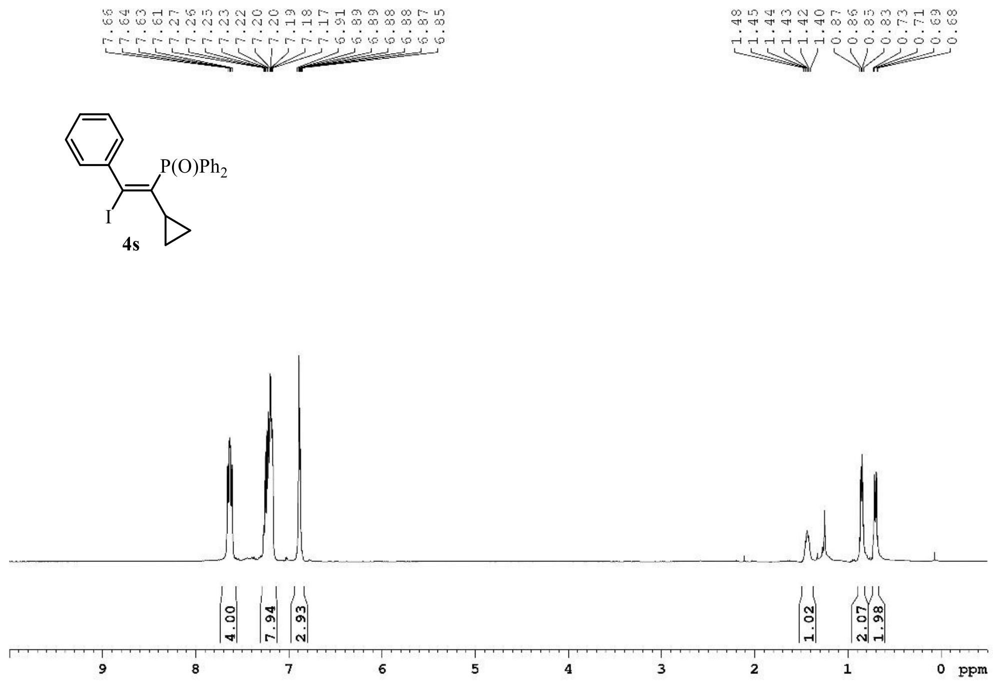

^{13}C NMR (101 MHz, $CDCl_3$) of 4s

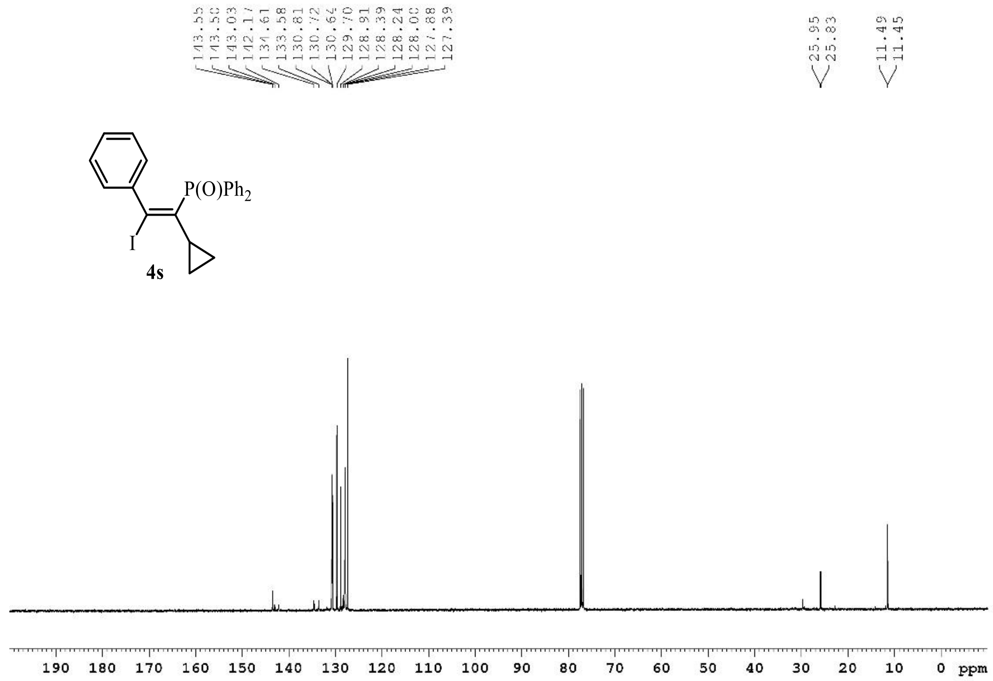

^{31}P NMR (162 MHz, CDCl$_3$) of 4s

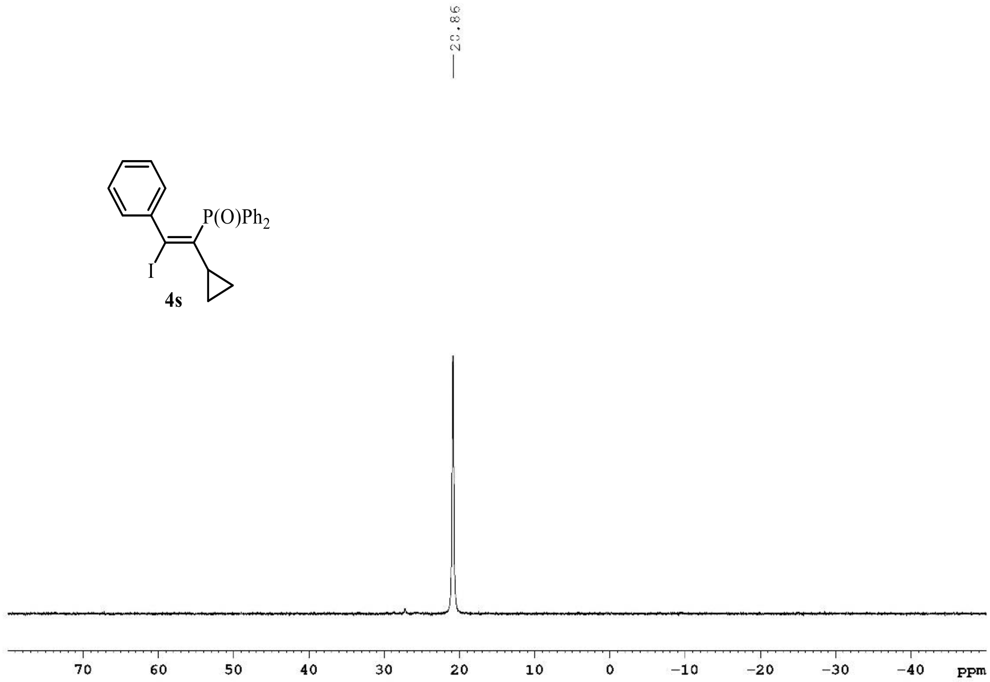

^{1}H NMR (400 MHz, CDCl$_3$) of 4t

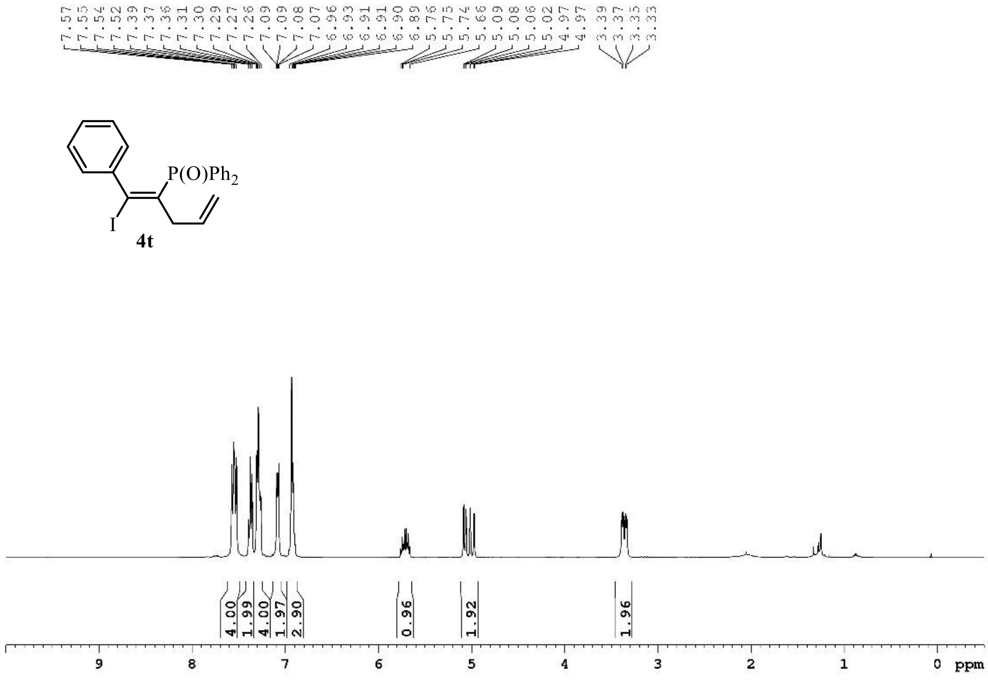

^{13}C NMR (101 MHz, $CDCl_3$) of 4t

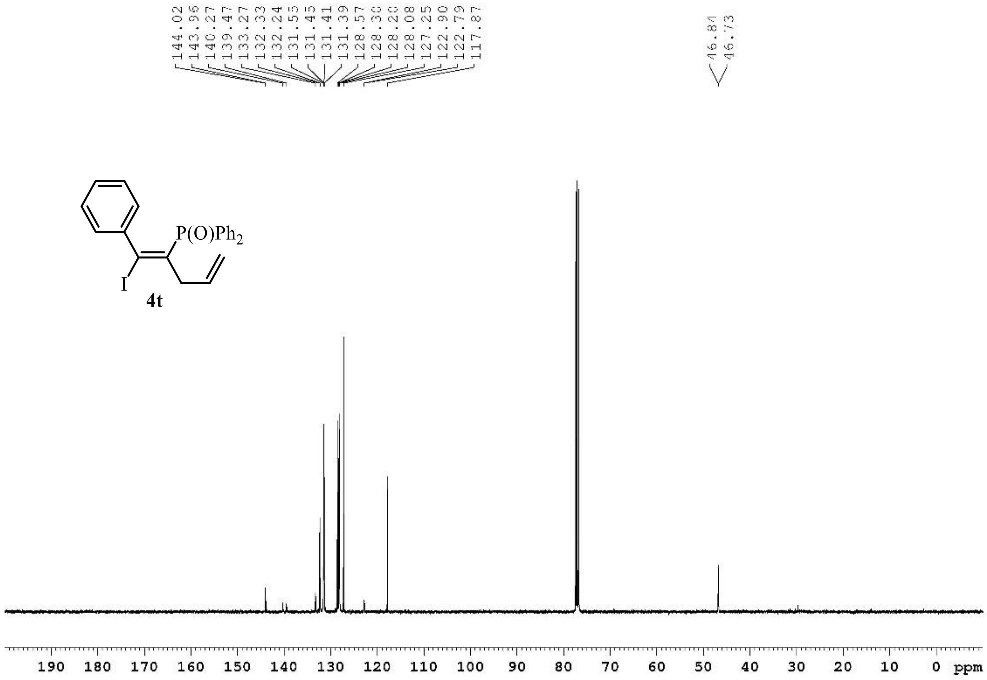

^{31}P NMR (162 MHz, $CDCl_3$) of 4t

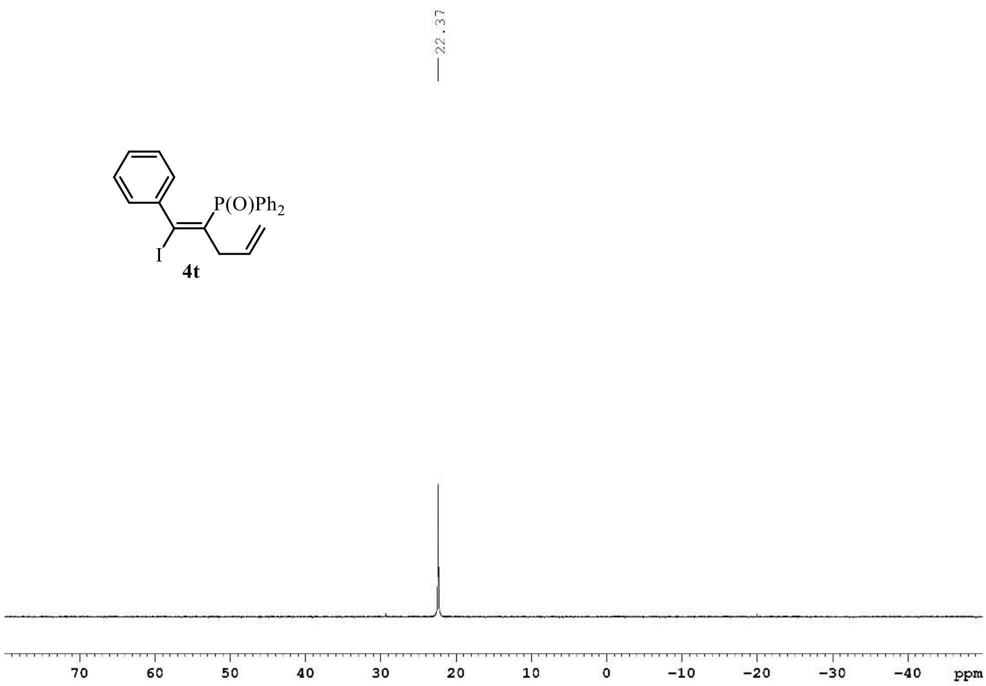

^{1}H NMR (400 MHz, $CDCl_3$) of 4u

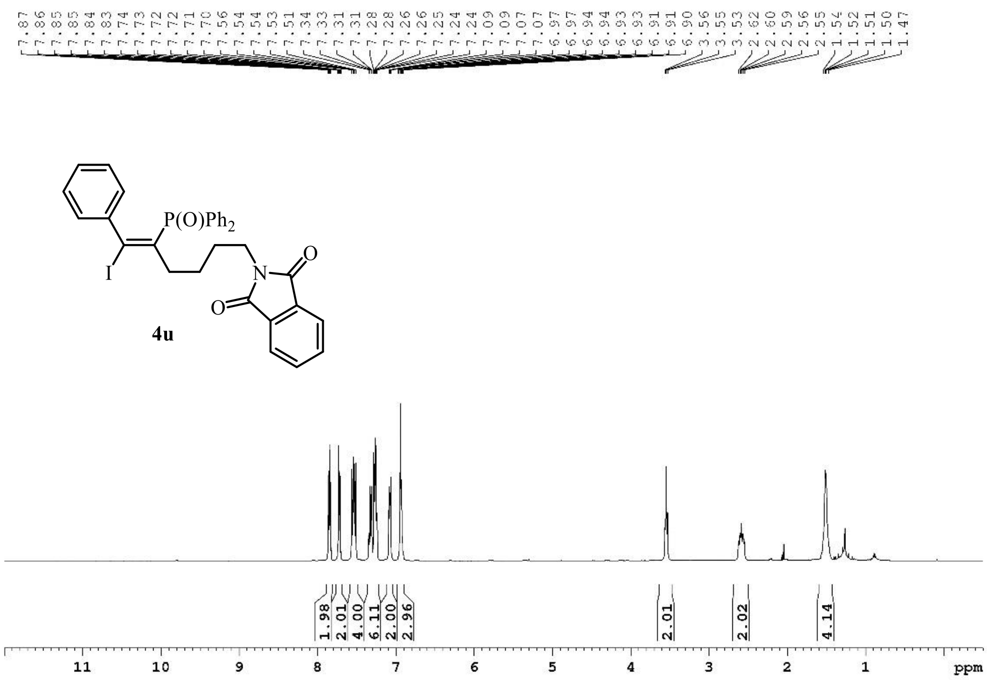

^{13}C NMR (101 MHz, $CDCl_3$) of 4u

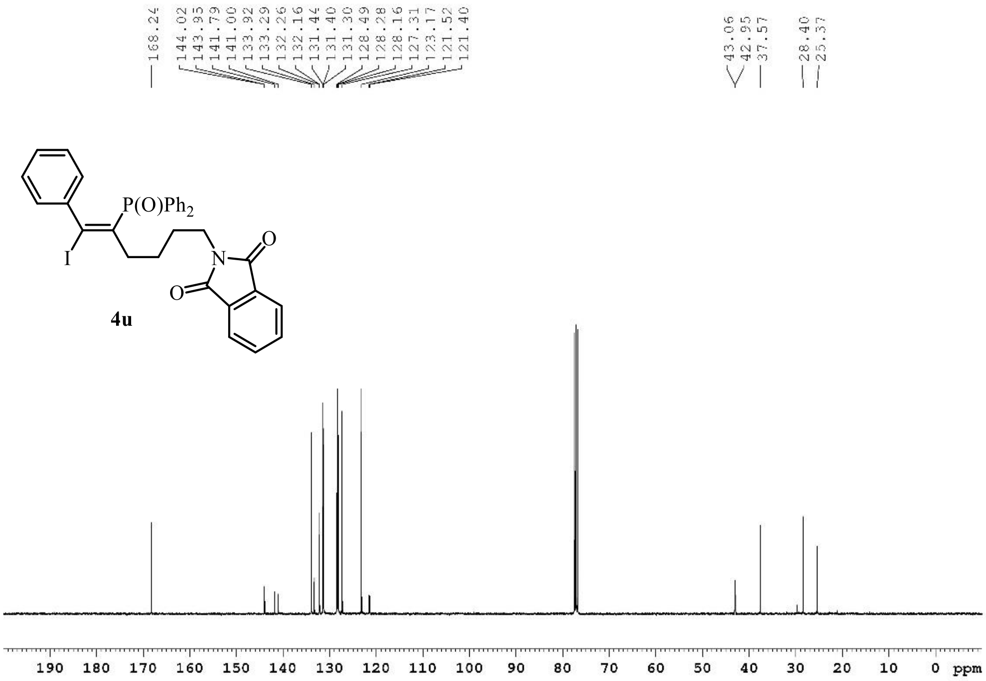

^{31}P NMR (162 MHz, CDCl$_3$) of 4u

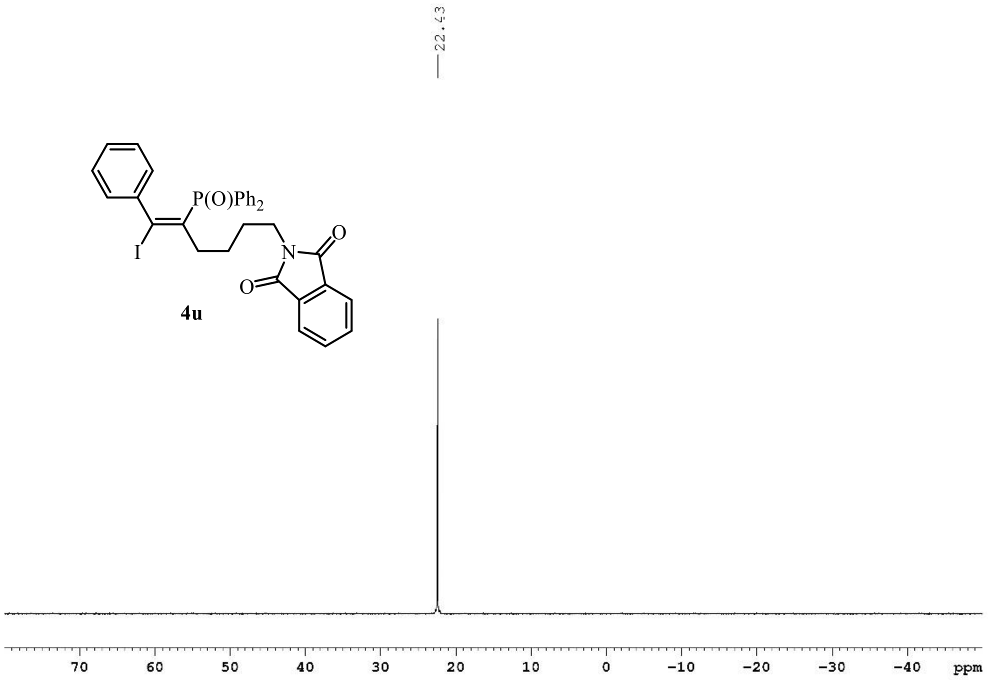

^{1}H NMR (400 MHz, CDCl$_3$) of 4v

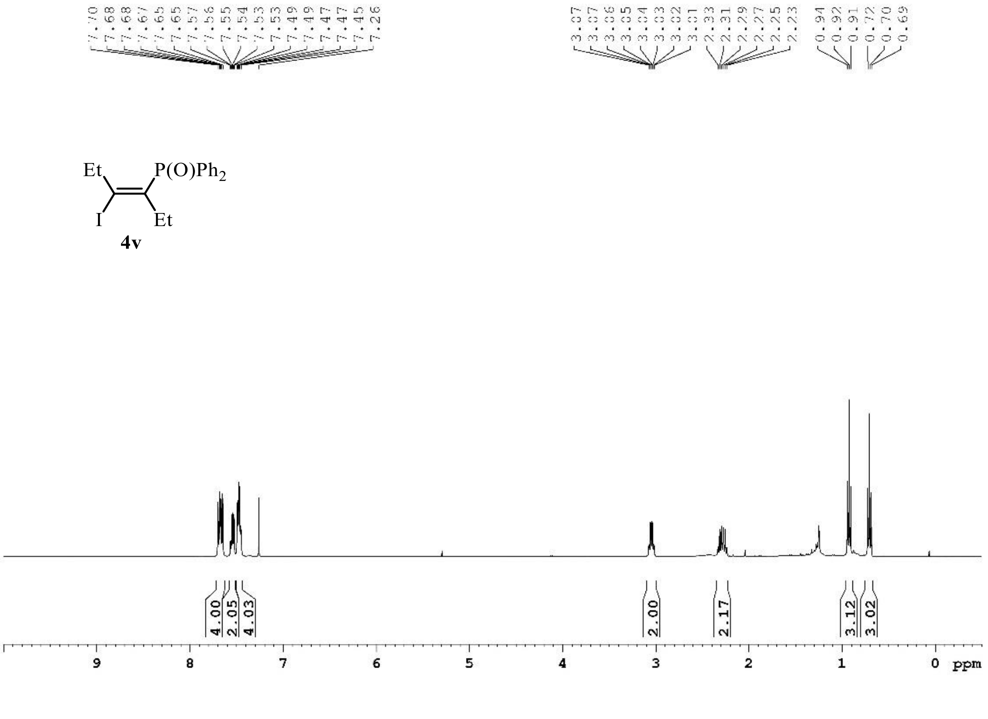

^{13}C NMR (101 MHz, $CDCl_3$) of 4v

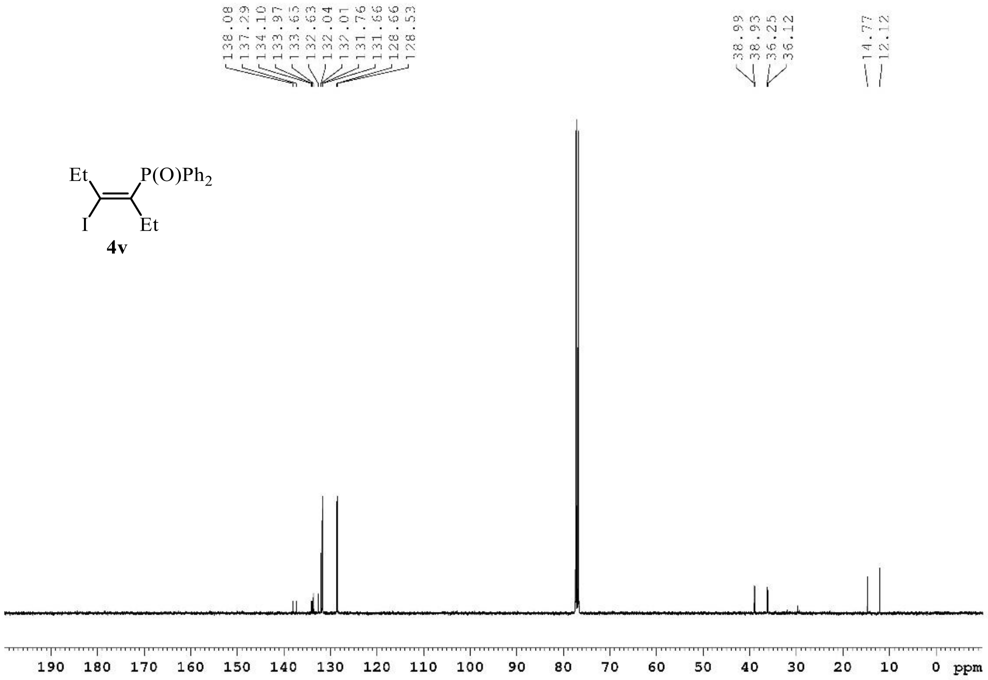

^{31}P NMR (162 MHz, $CDCl_3$) of 4v

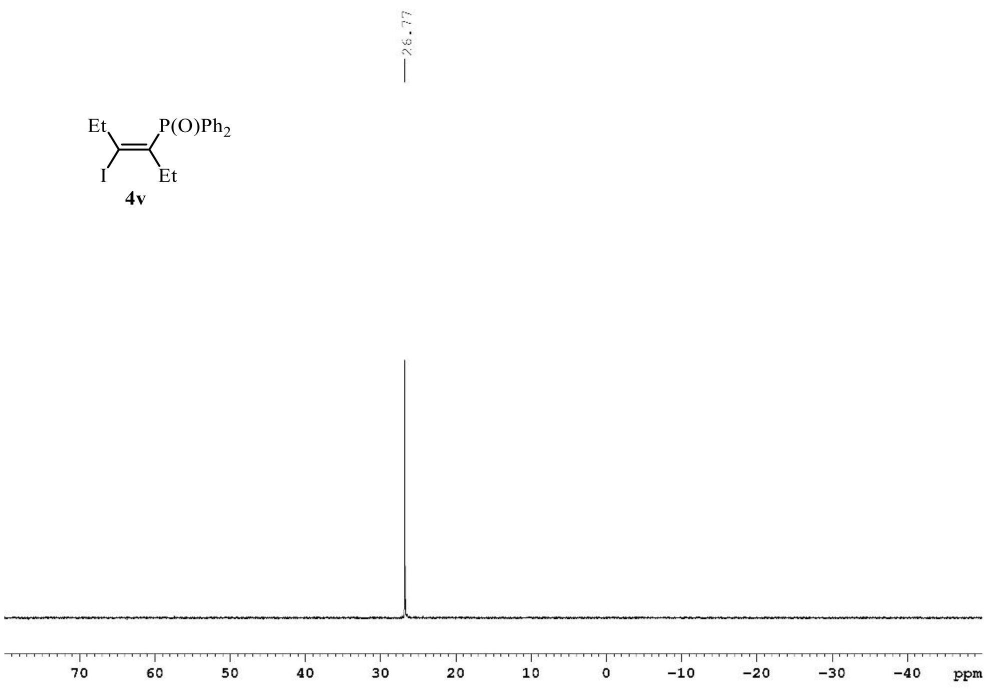

^{1}H NMR (400 MHz, $CDCl_3$) of 6

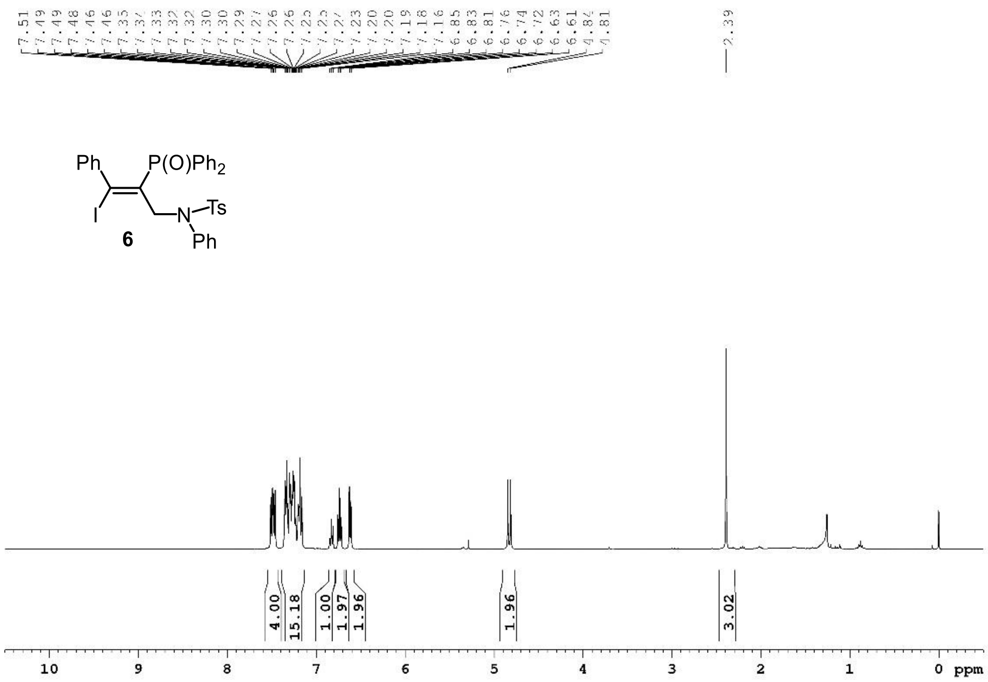

^{13}C NMR (101 MHz, $CDCl_3$) of 6

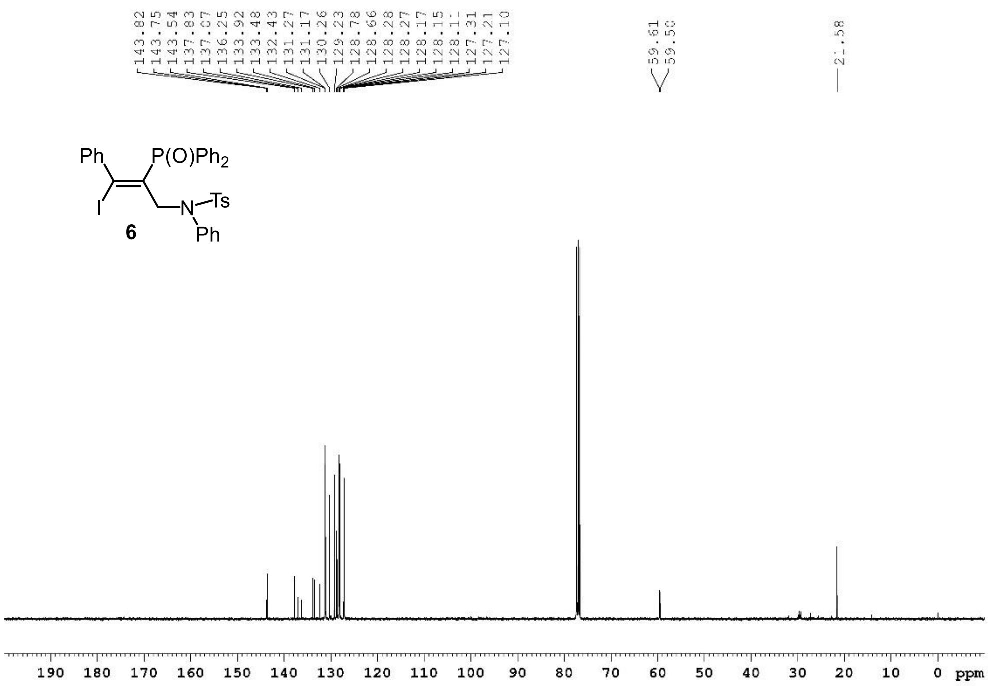

^{31}P NMR (162 MHz, $CDCl_3$) of 6

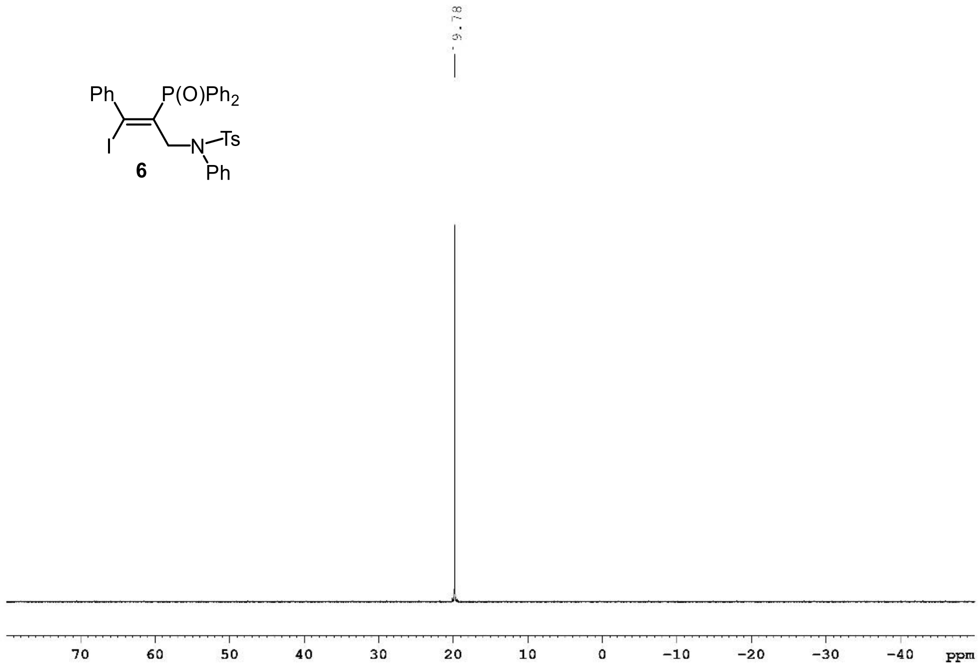

^{1}H NMR (400 MHz, $CDCl_3$) of 8

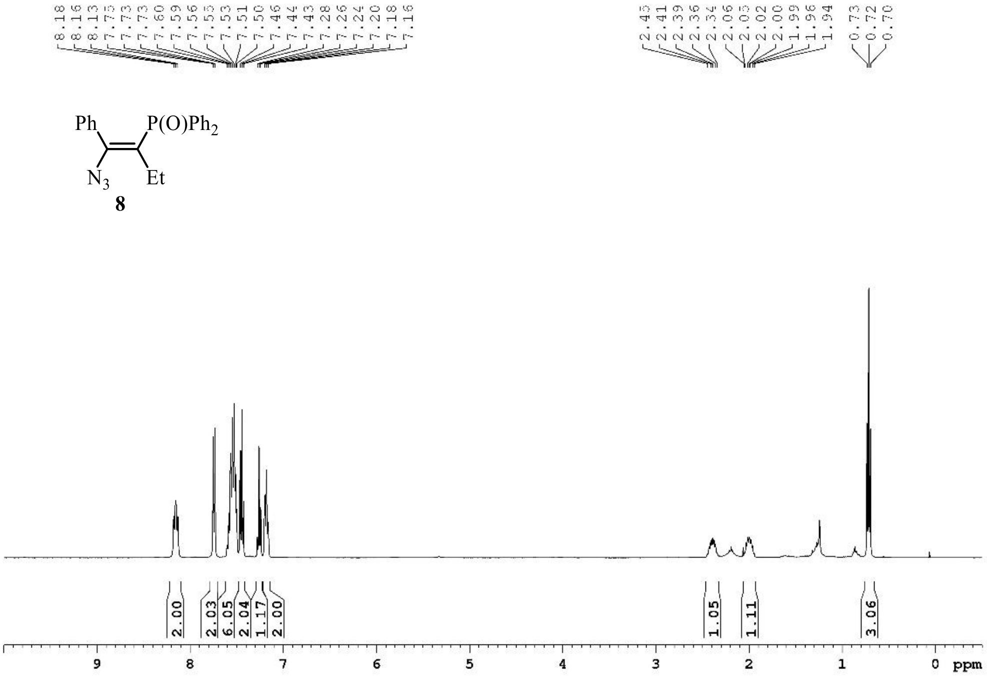

^{13}C NMR (101 MHz, $CDCl_3$) of 8

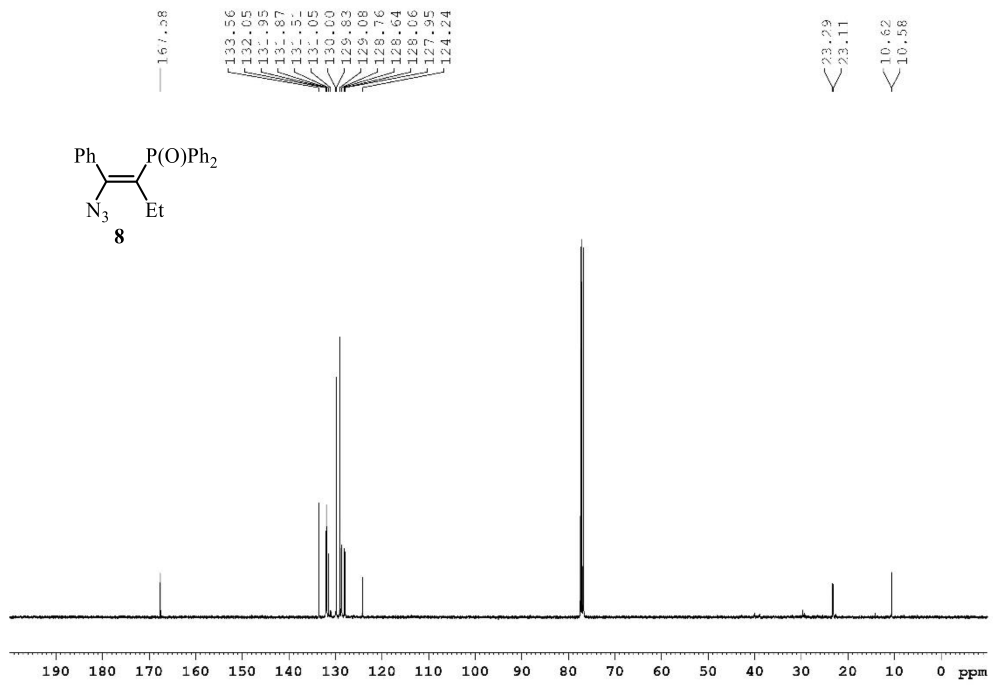

^{31}P NMR (162 MHz, $CDCl_3$) of 8

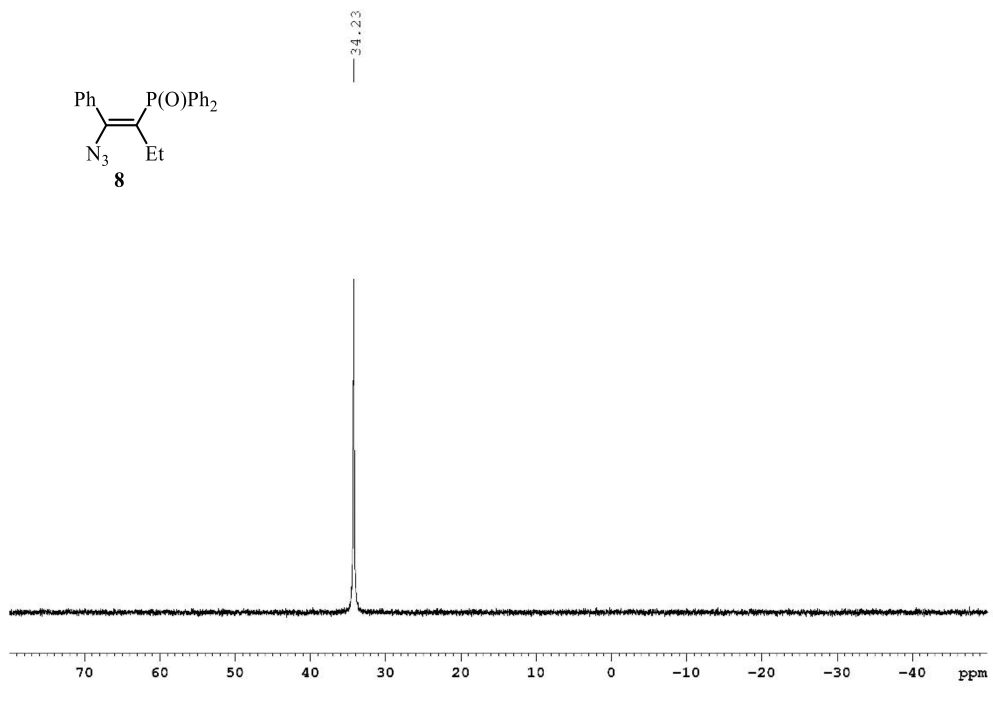

^{1}H NMR (400 MHz, $CDCl_3$) of 9

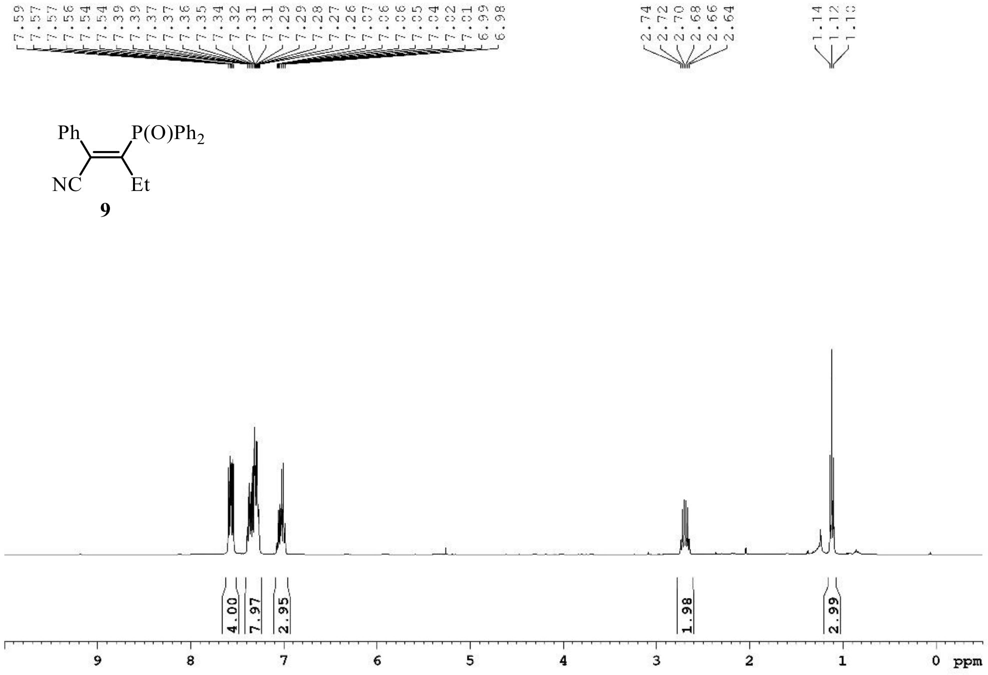

^{13}C NMR (101 MHz, $CDCl_3$) of 9

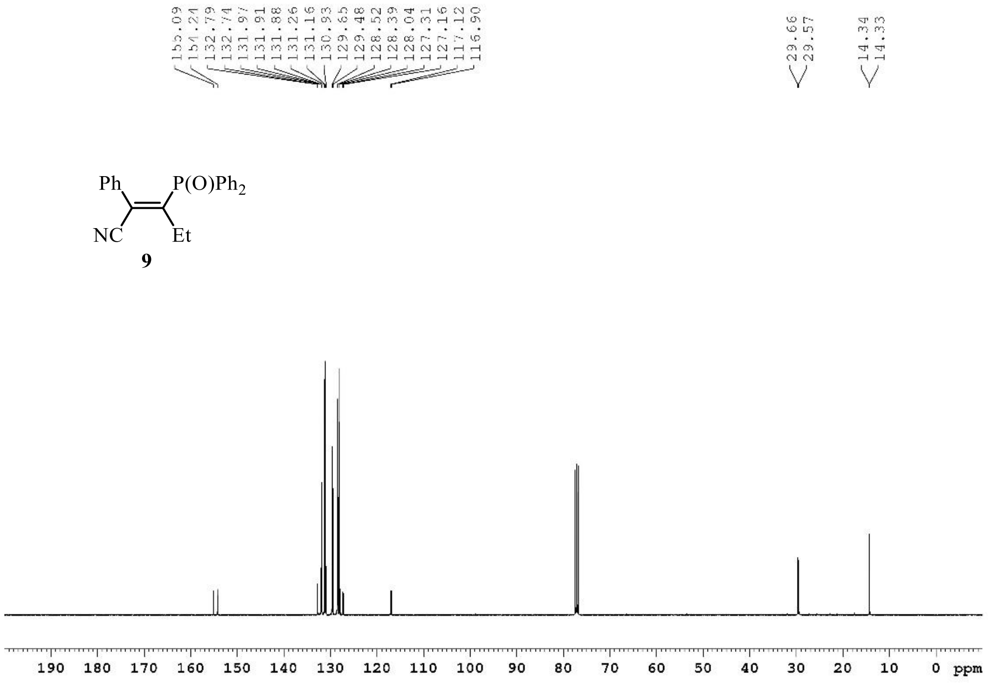

^{31}P NMR (162 MHz, CDCl$_3$) of 9

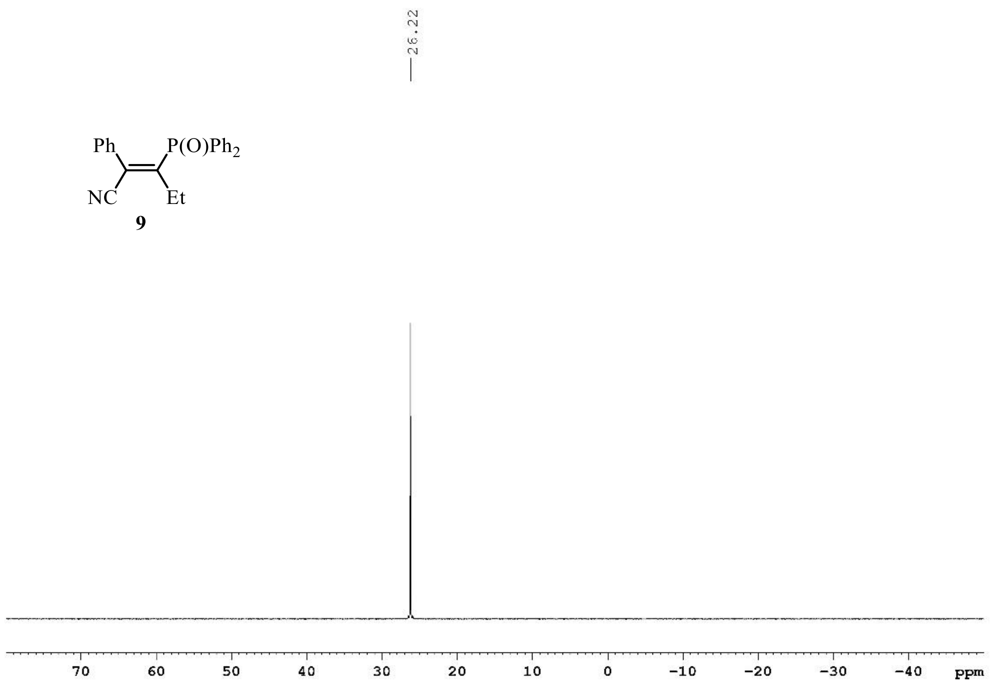

^{1}H NMR (400 MHz, CDCl$_3$) of 10

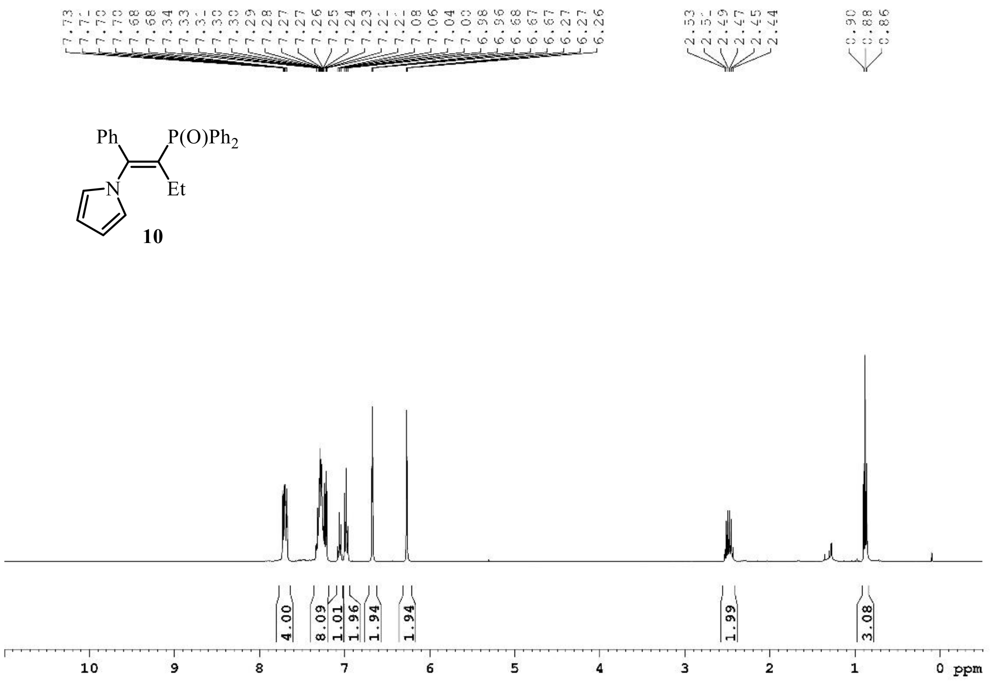

^{13}C NMR (101 MHz, CDCl$_3$) of 10

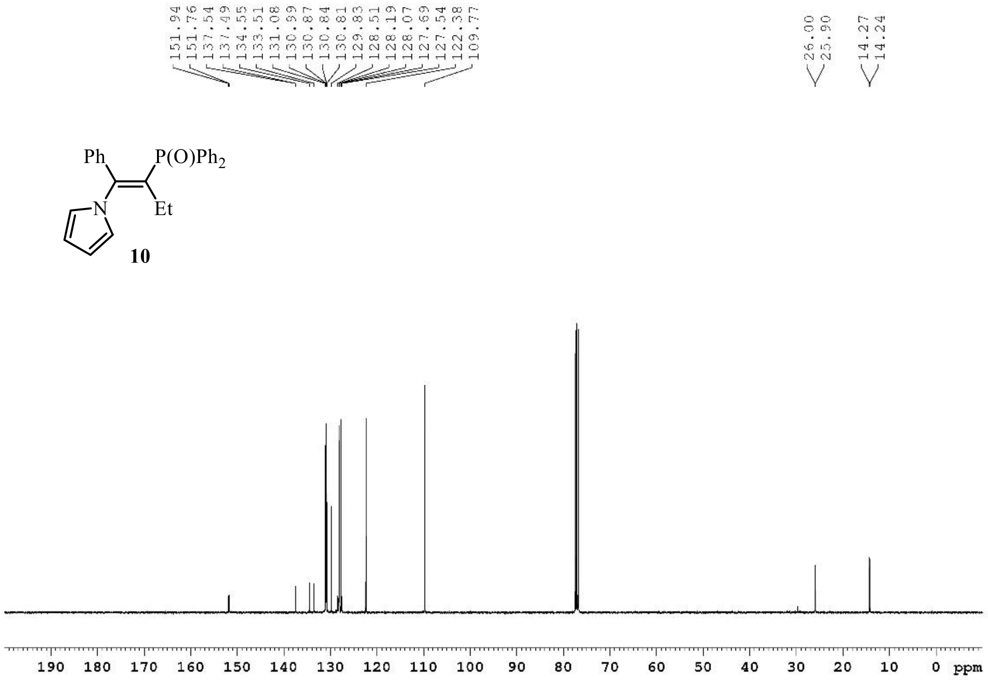

^{31}P NMR (162 MHz, CDCl$_3$) of 10

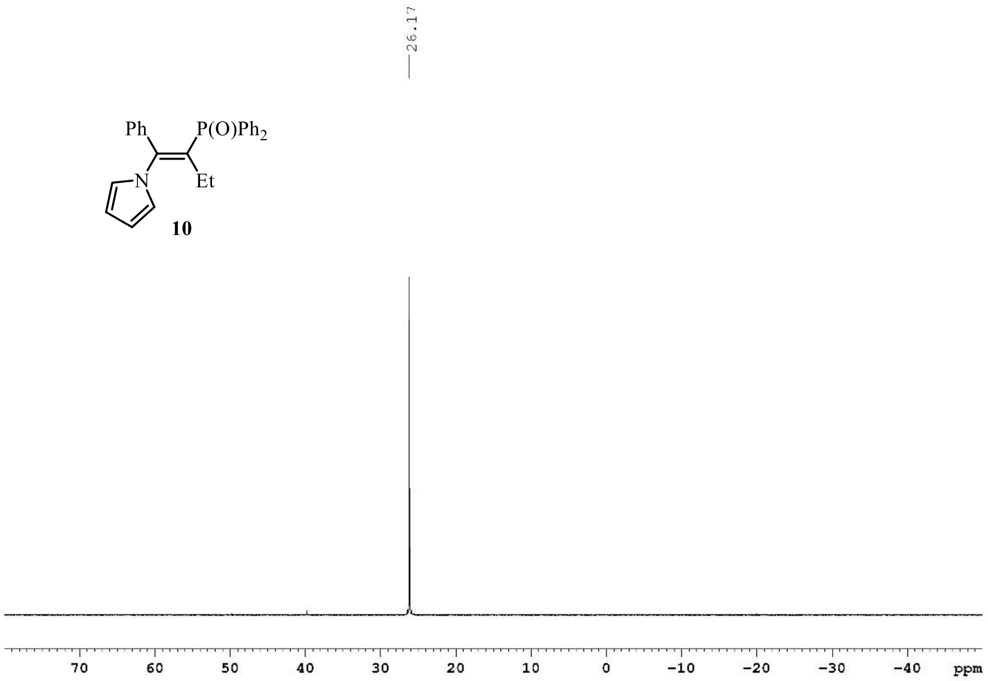

^{1}H NMR (400 MHz, $CDCl_3$) of 11-1

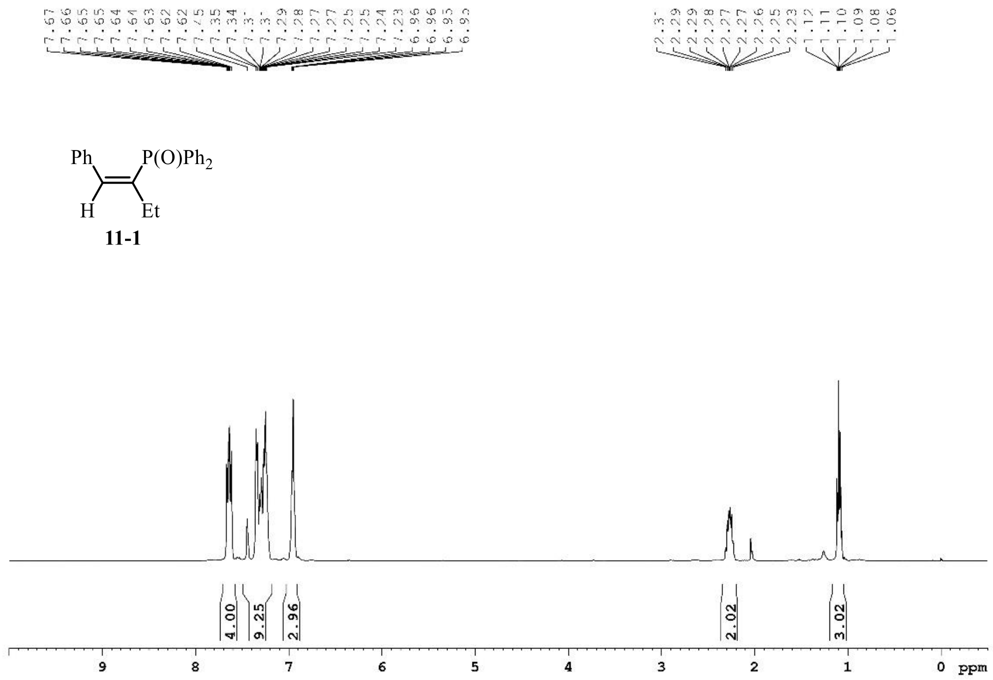

^{13}C NMR (101 MHz, $CDCl_3$) of 11-1

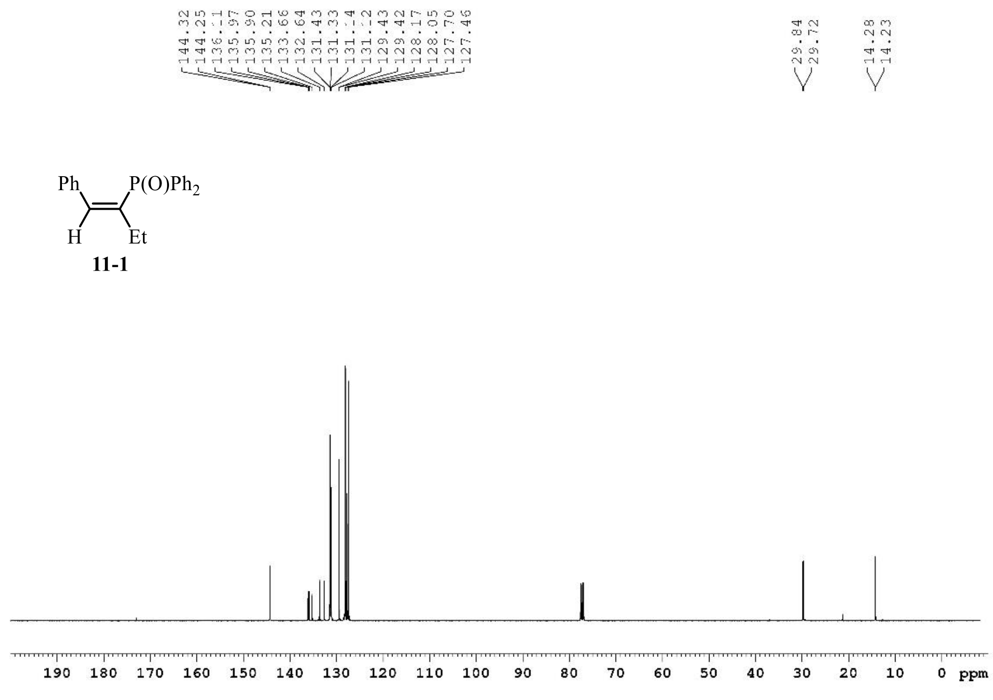

^{31}P NMR (162 MHz, $CDCl_3$) of 11-1

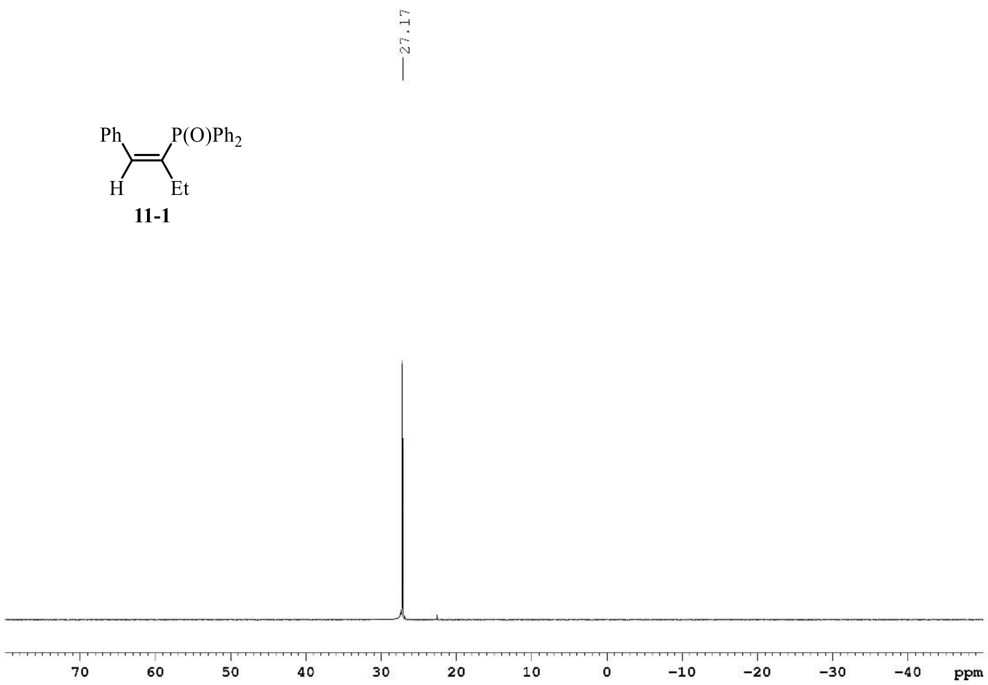

^{1}H NMR (400 MHz, $CDCl_3$) of 11-2

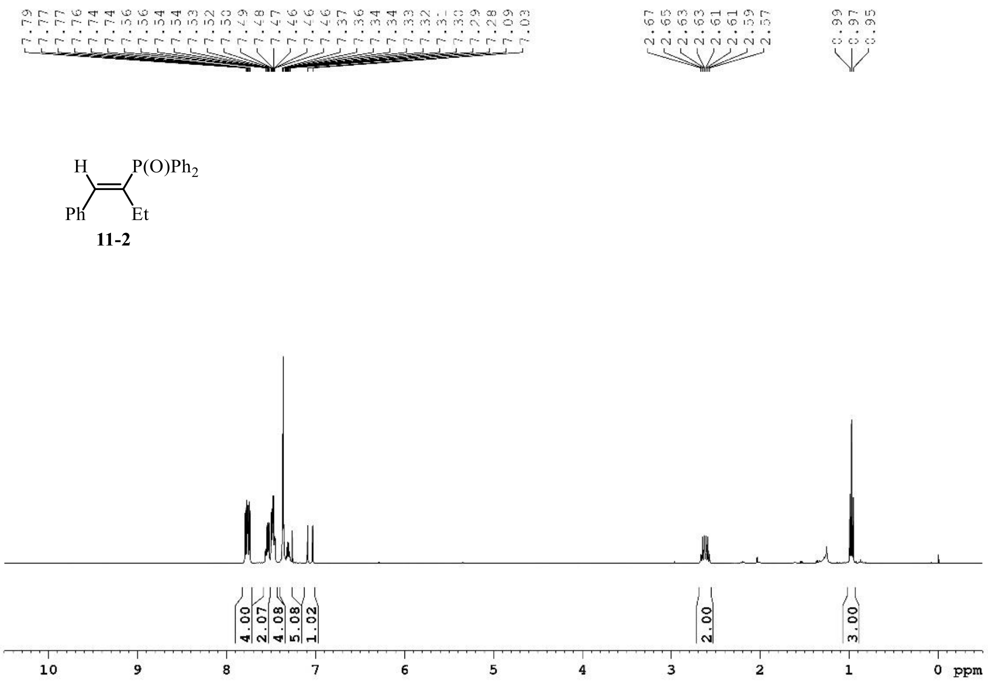

^{13}C NMR (101 MHz, $CDCl_3$) of 11-2

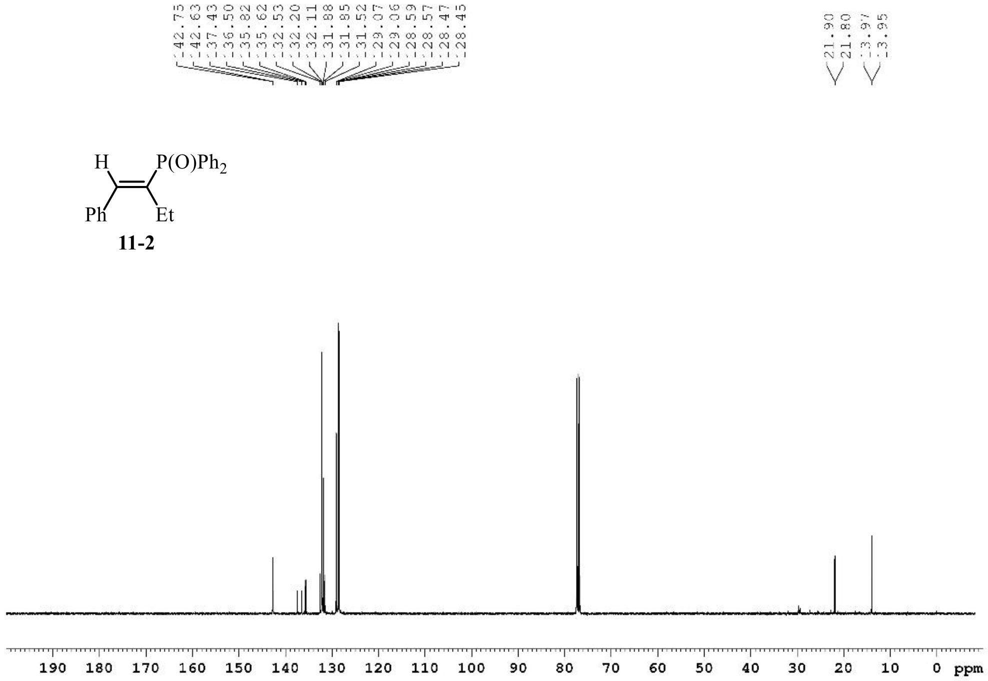

^{31}P NMR (162 MHz, $CDCl_3$) of 11-2

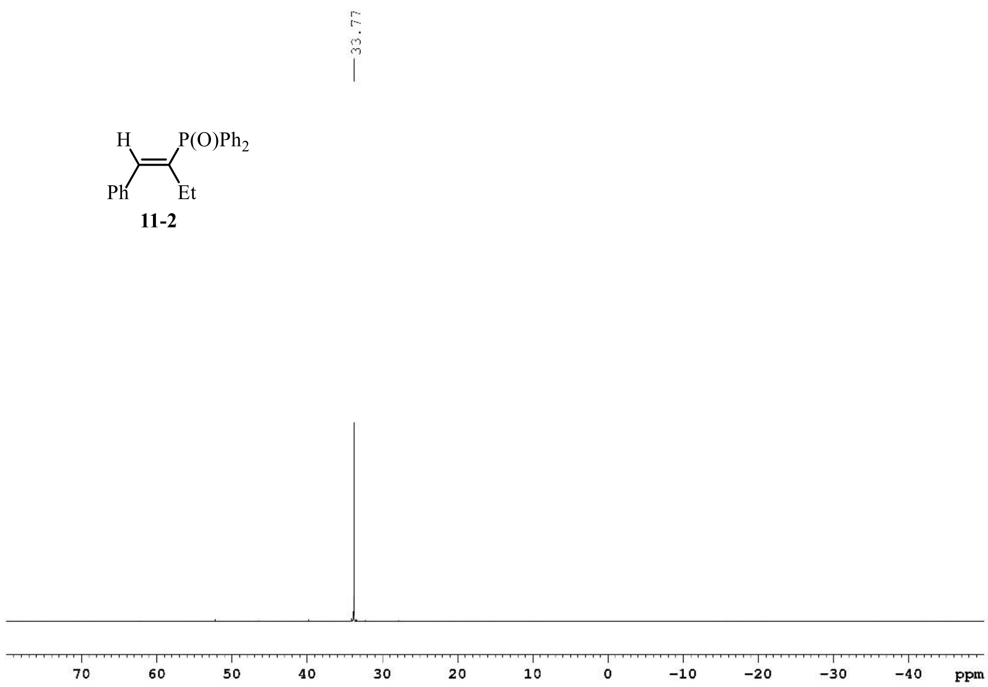

^{1}H NMR (400 MHz, $CDCl_3$) of 12

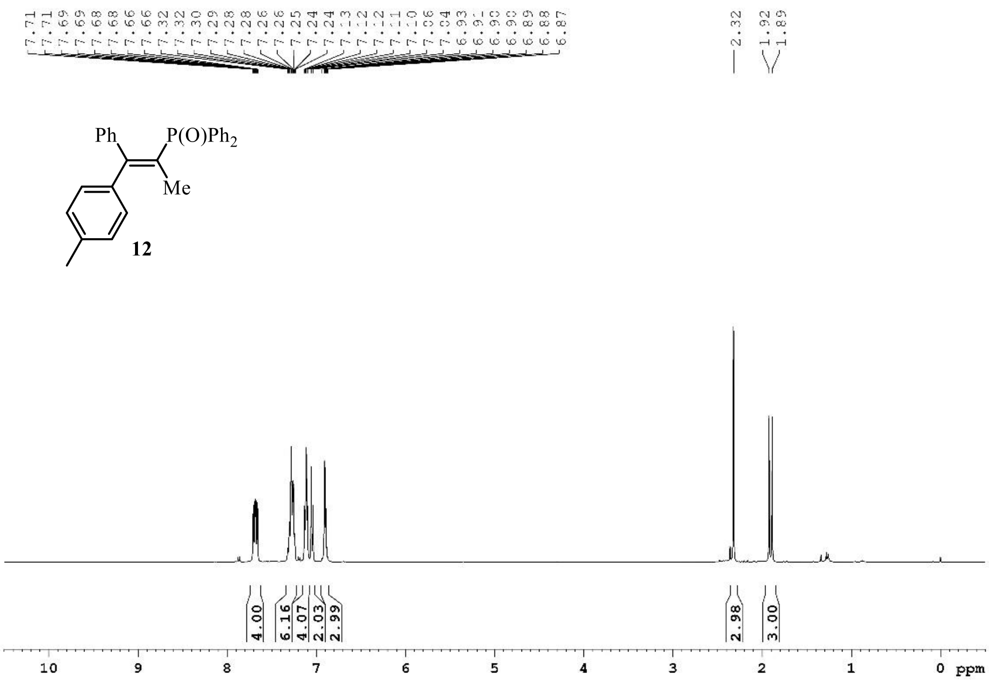

^{13}C NMR (101 MHz, $CDCl_3$) of 12

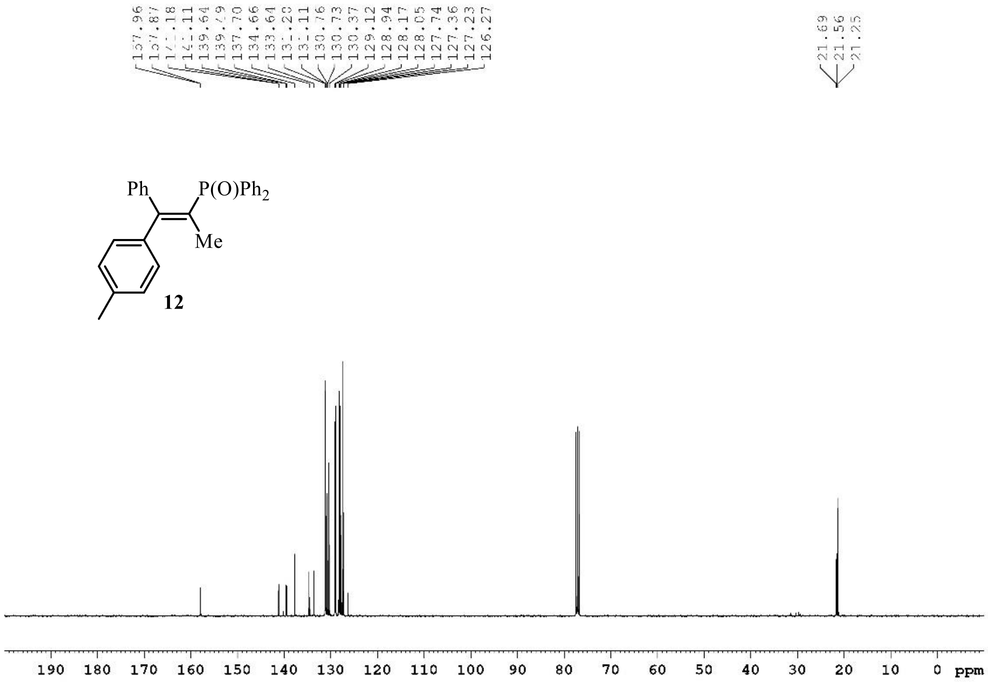

^{31}P NMR (162 MHz, CDCl$_3$) of 12

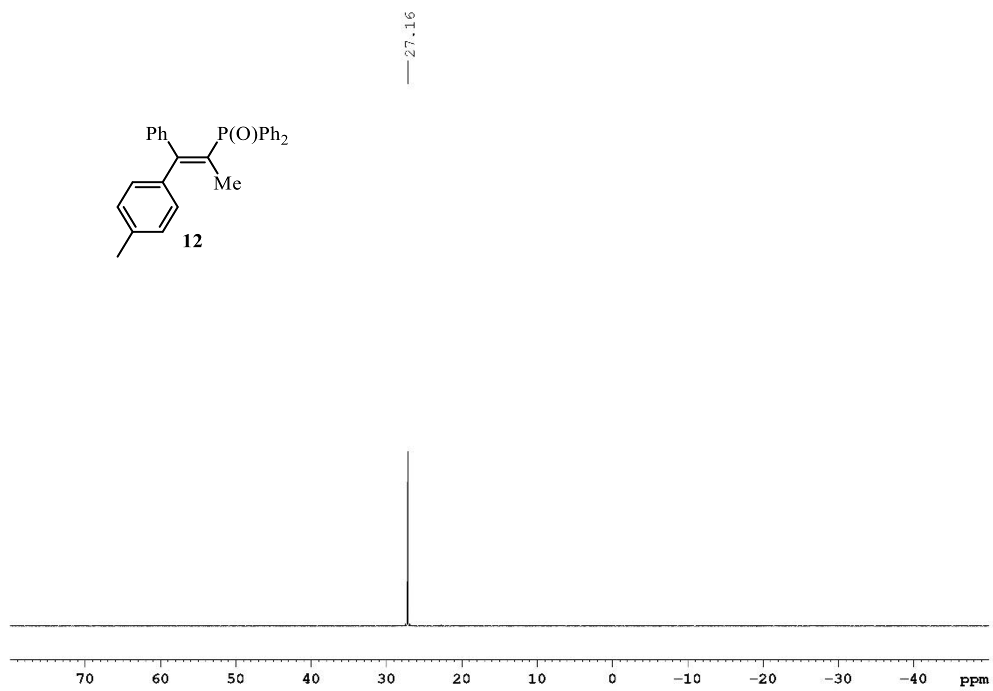

^{1}H NMR (400 MHz, CDCl$_3$) of 13

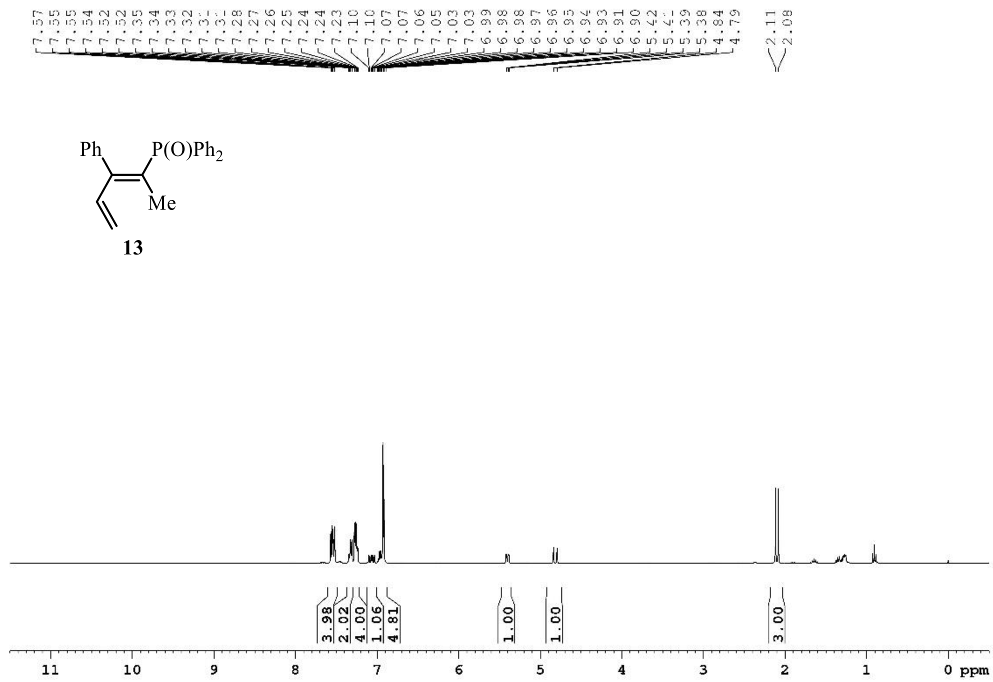

^{13}C NMR (101 MHz, $CDCl_3$) of 13

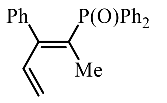

13

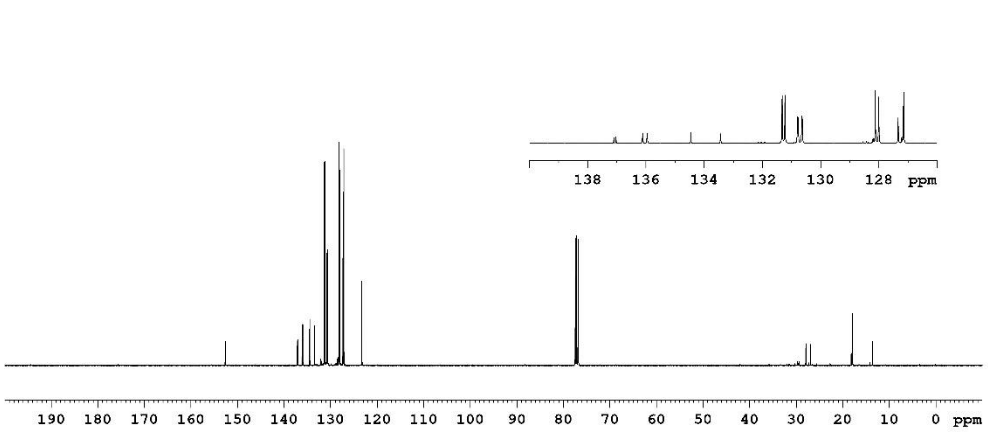

^{31}P NMR (162 MHz, $CDCl_3$) of 13

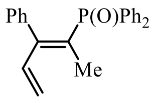

13

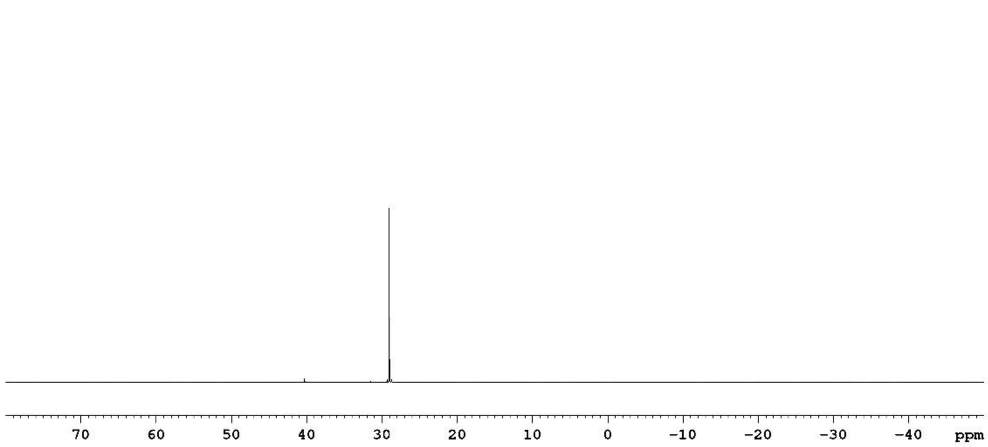

^{1}H NMR (400 MHz, $CDCl_3$) of 14

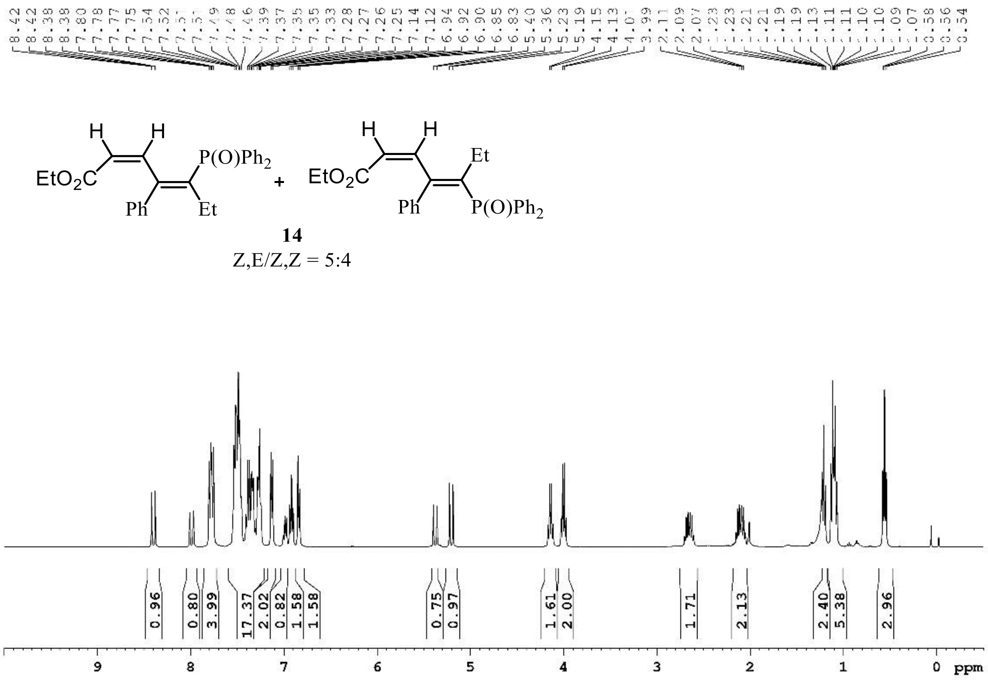

^{13}C NMR (101 MHz, $CDCl_3$) of 14

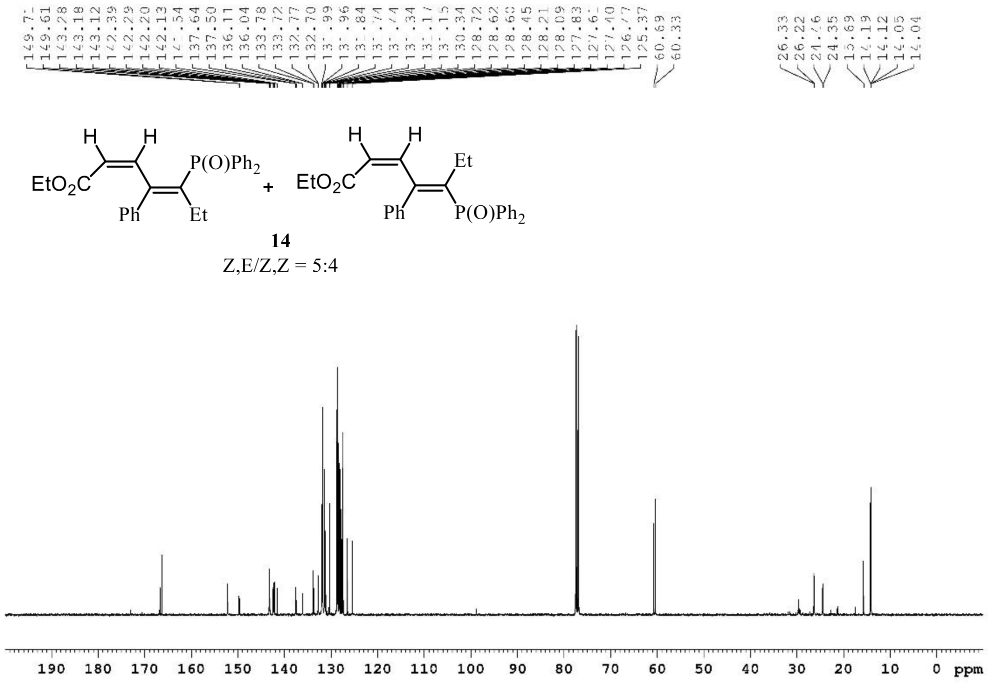

^{31}P NMR (162 MHz, $CDCl_3$) of 14

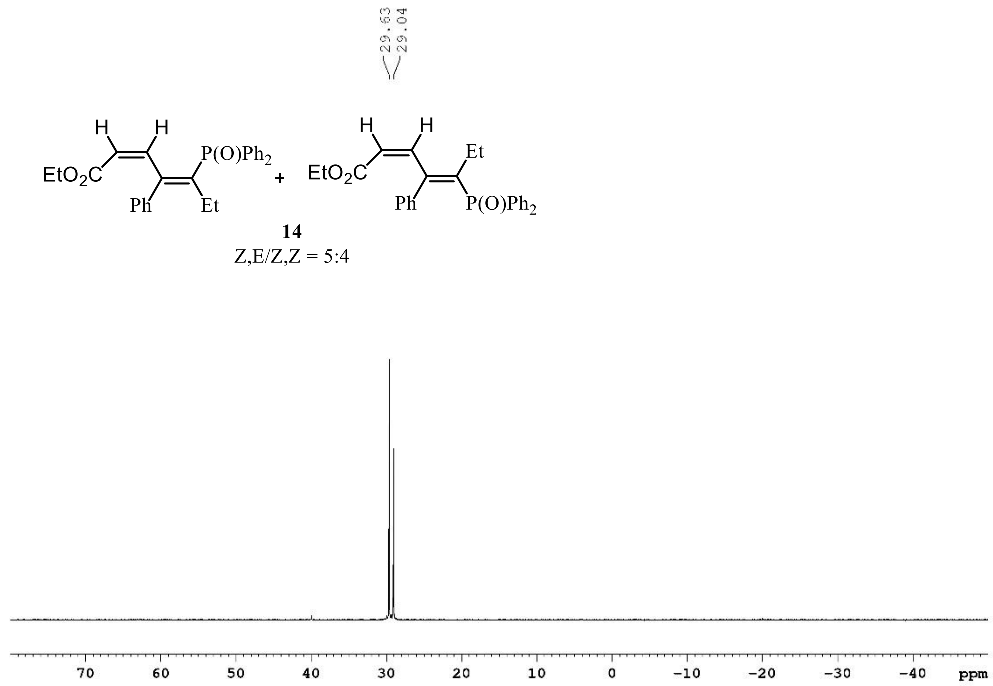

HSQC NMR Spectra of (*Z, Z*)-14

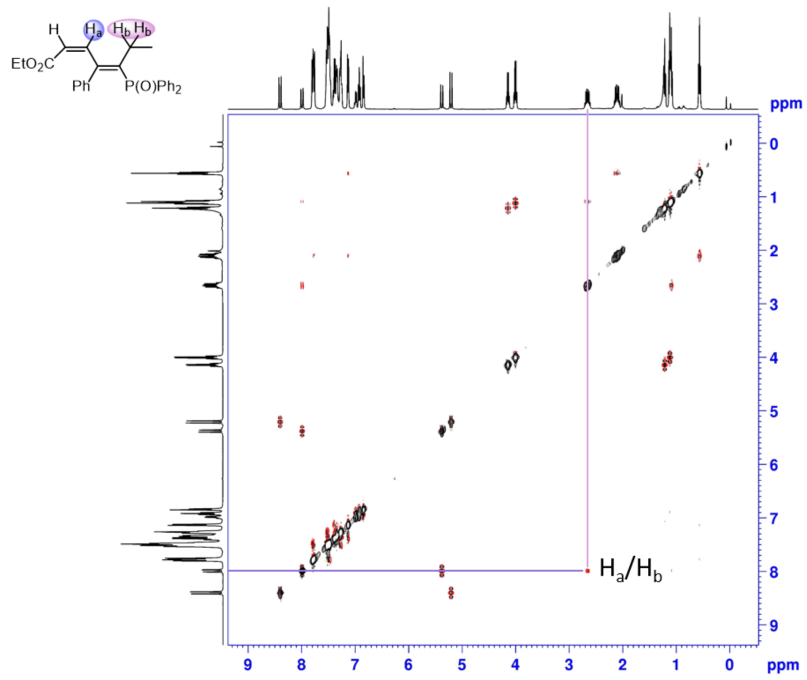

^{1}H NMR (400 MHz, CDCl$_3$) of 15b

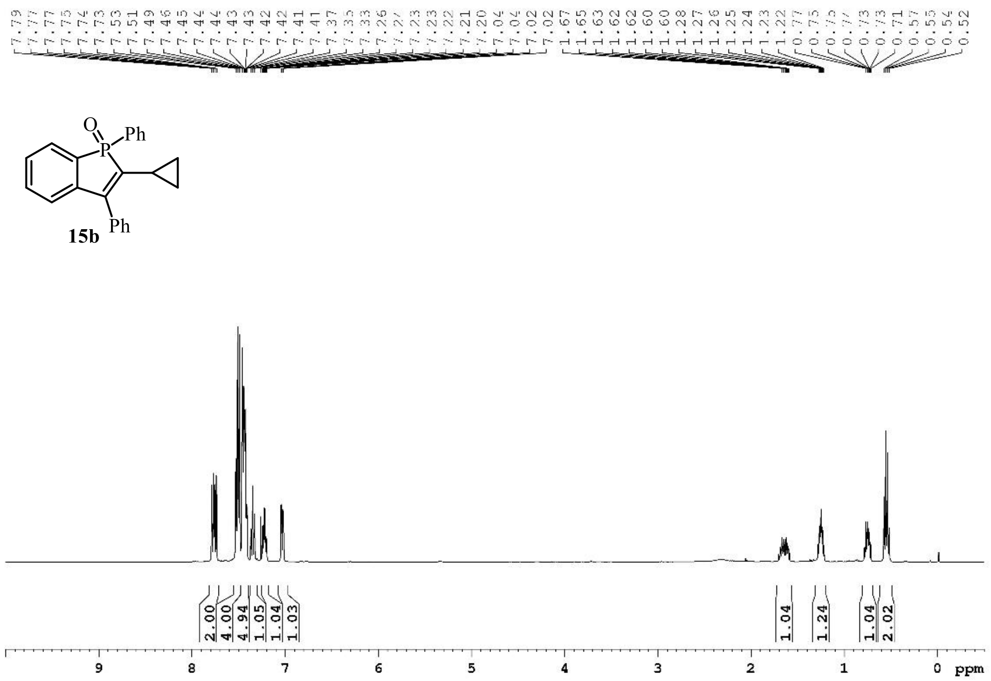

^{13}C NMR (101 MHz, CDCl$_3$) of 15b

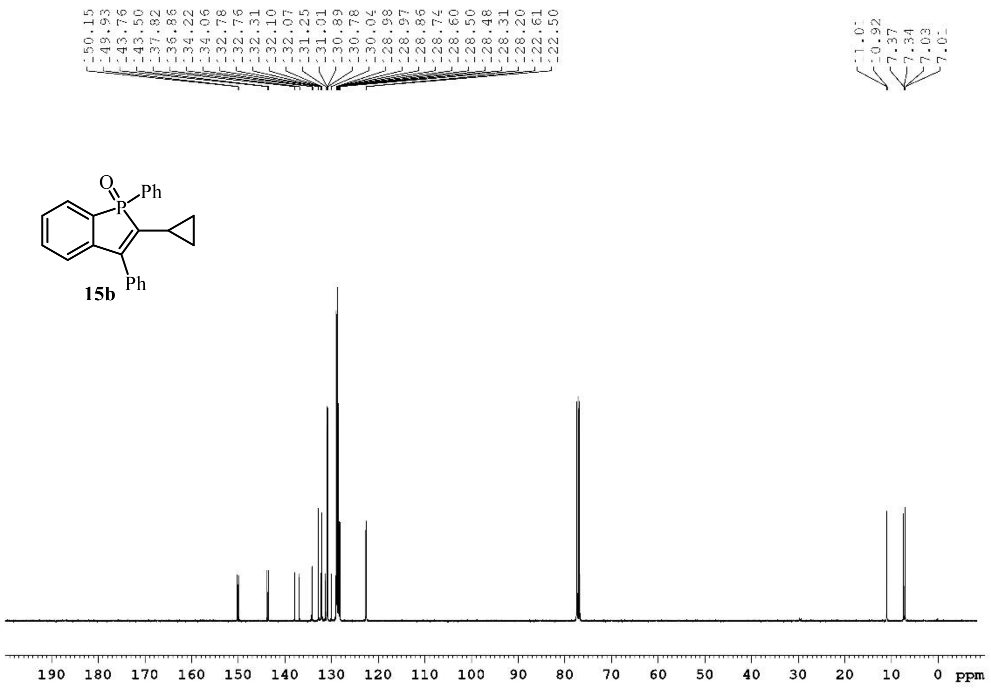

^{31}P NMR (162 MHz, CDCl$_3$) of 15b

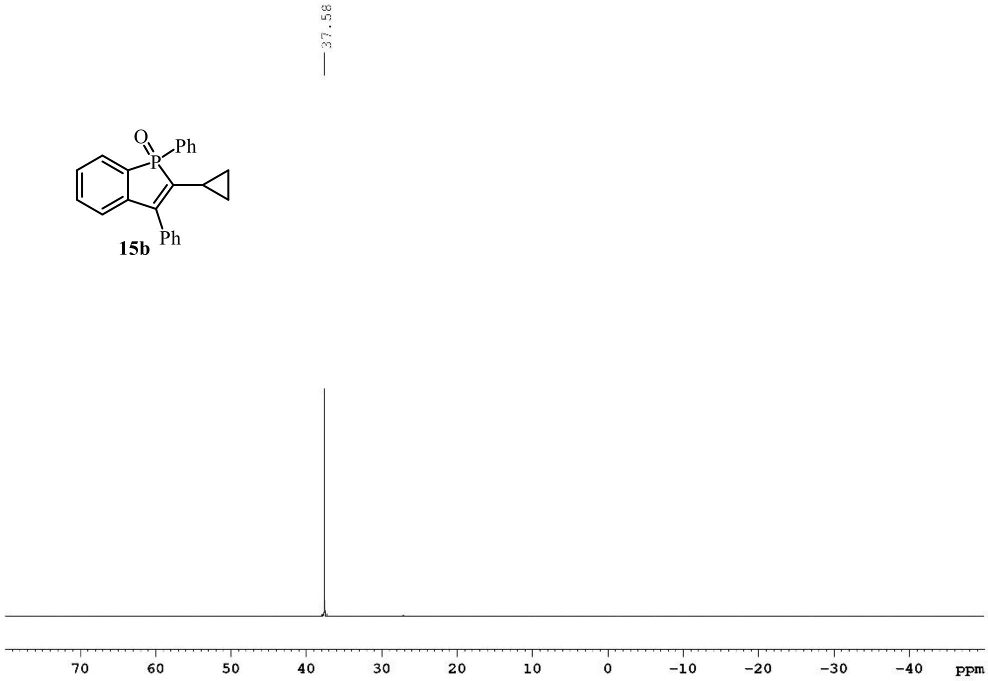

^{1}H NMR (400 MHz, CDCl$_3$) of 15c

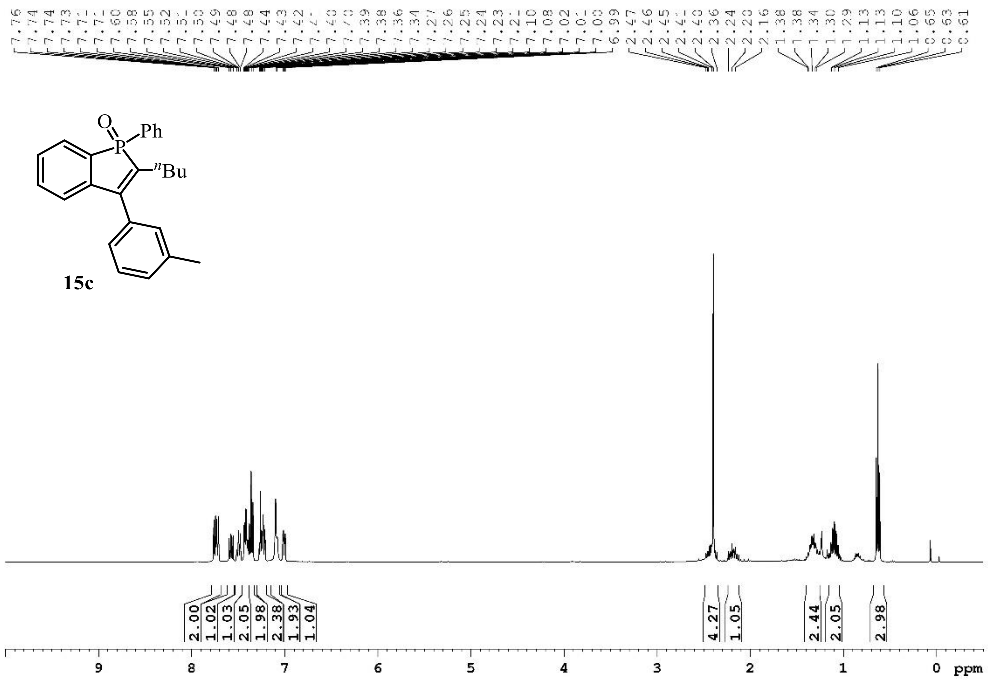

^{13}C NMR (101 MHz, $CDCl_3$) of 15c

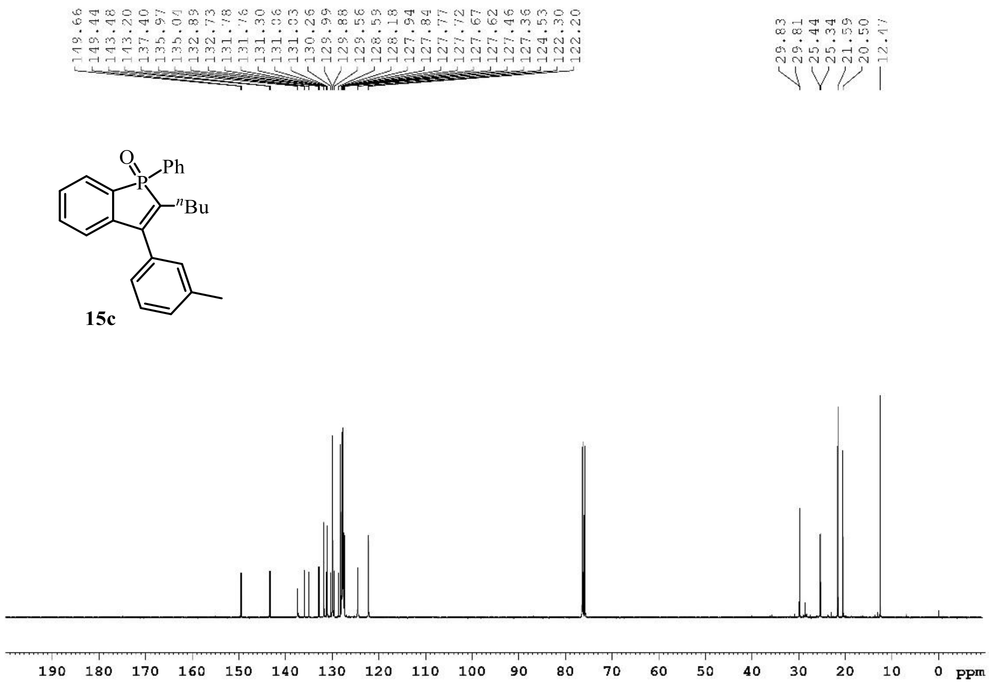

^{31}P NMR (162 MHz, $CDCl_3$) of 15c

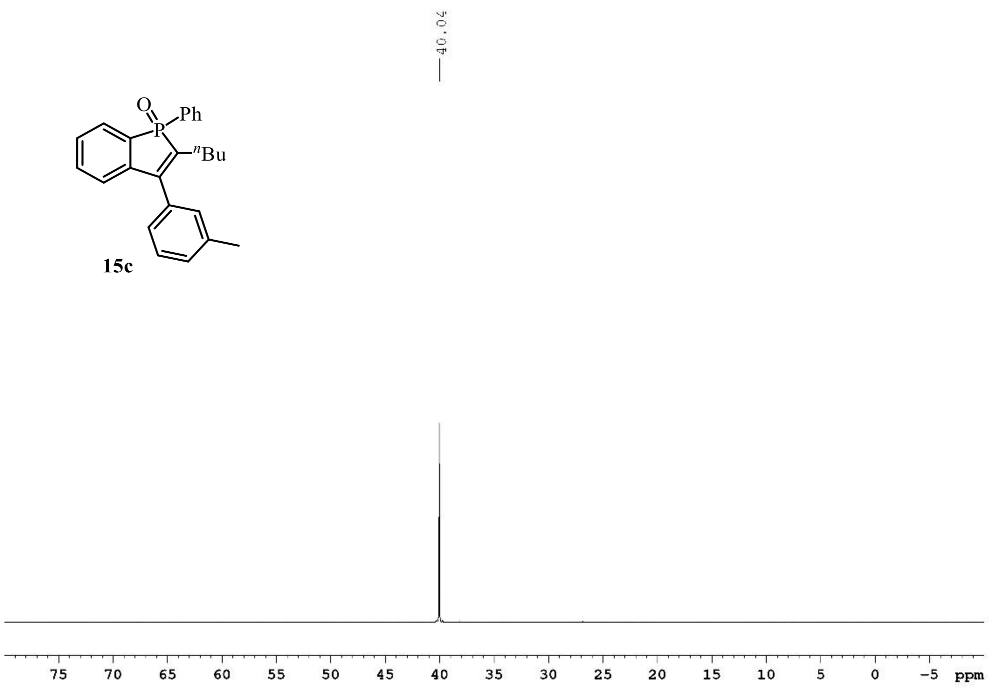

^{1}H NMR (400 MHz, $CDCl_3$) of 15d

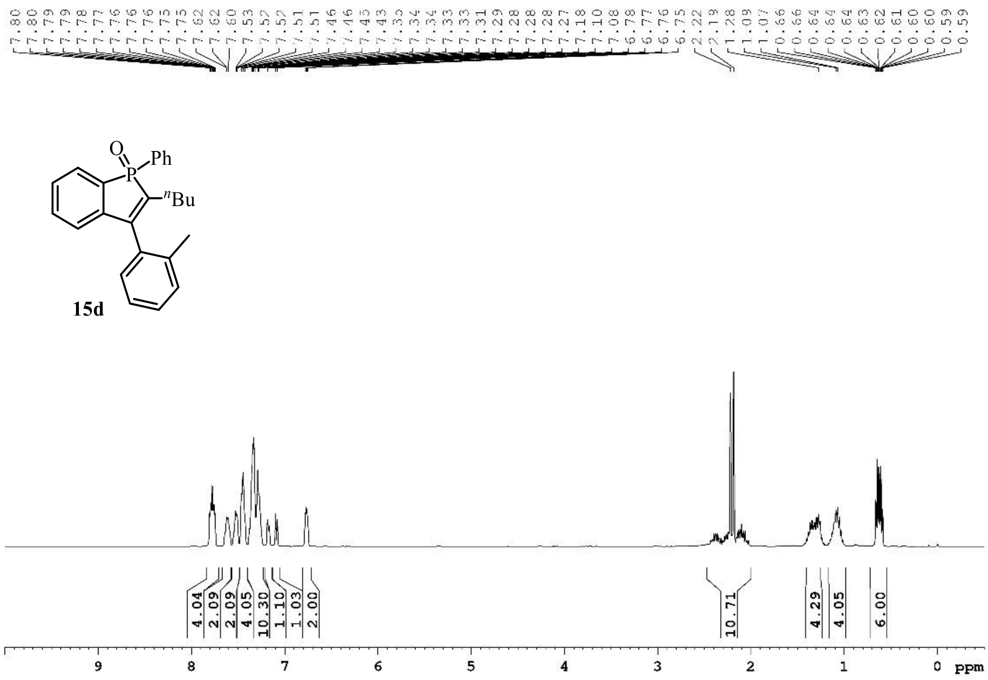

^{13}C NMR (101 MHz, $CDCl_3$) of 15d

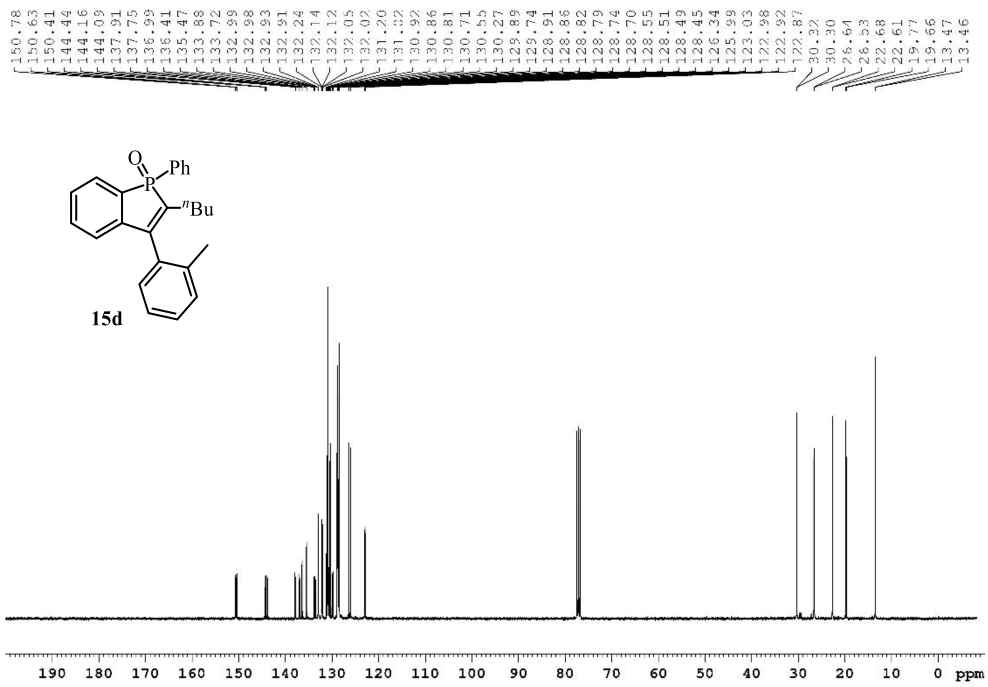

^{31}P NMR (162 MHz, $CDCl_3$) of 15d

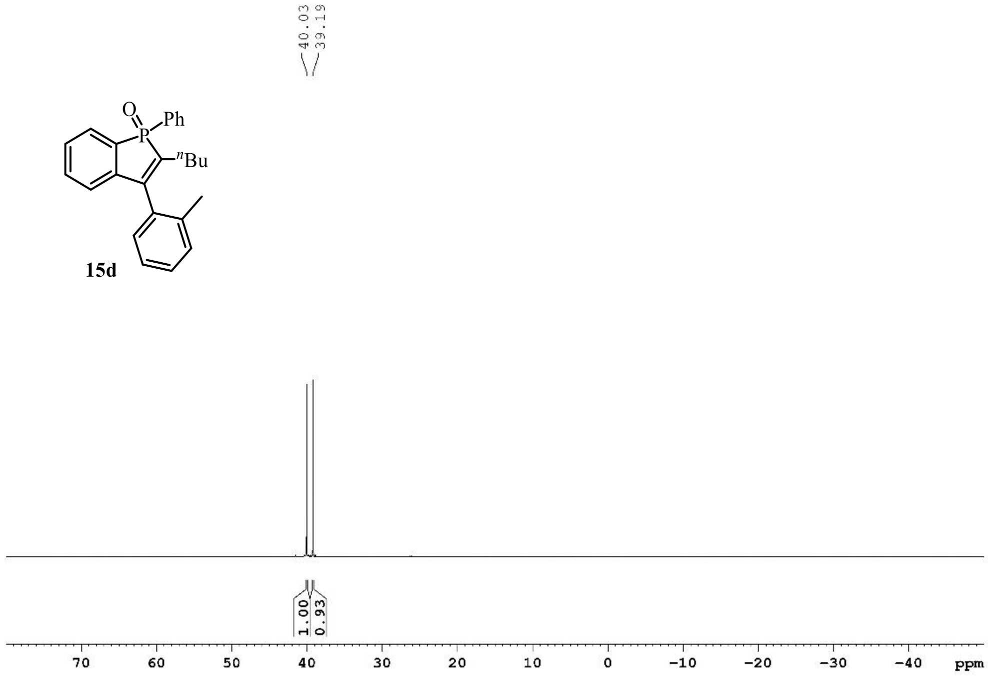

^{1}H NMR (400 MHz, $CDCl_3$) of 15e

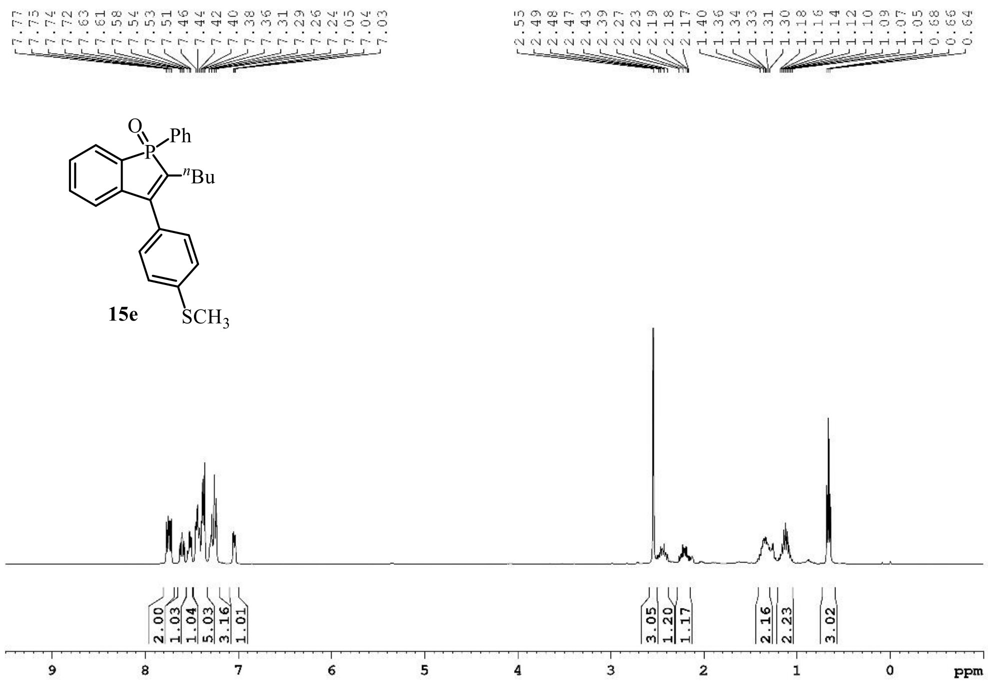

^{13}C NMR (101 MHz, $CDCl_3$) of 15e

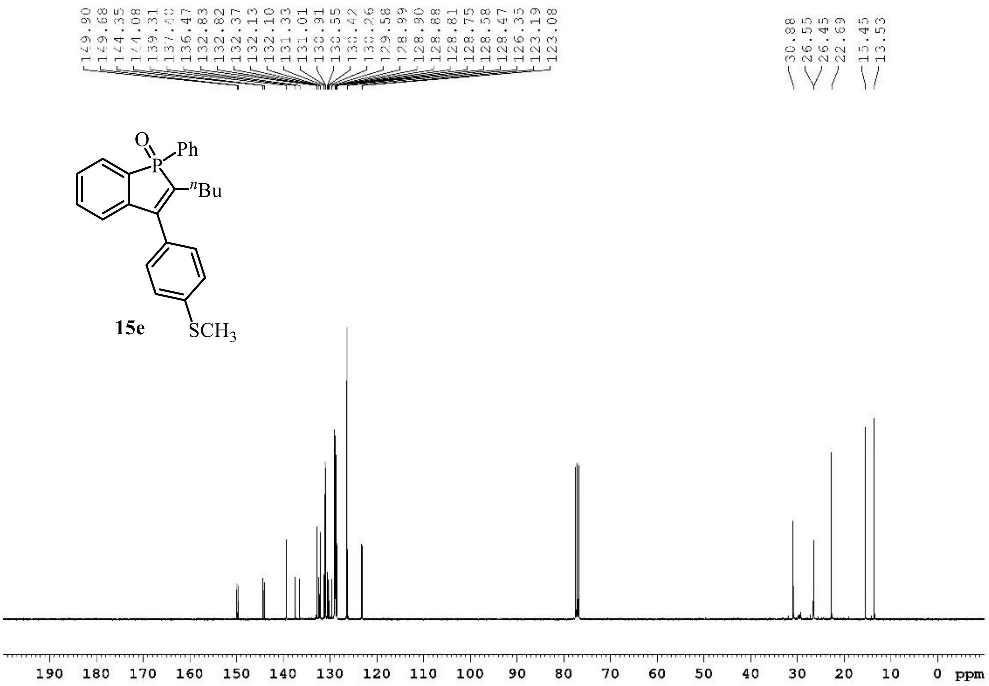

^{31}P NMR (162 MHz, $CDCl_3$) of 15e

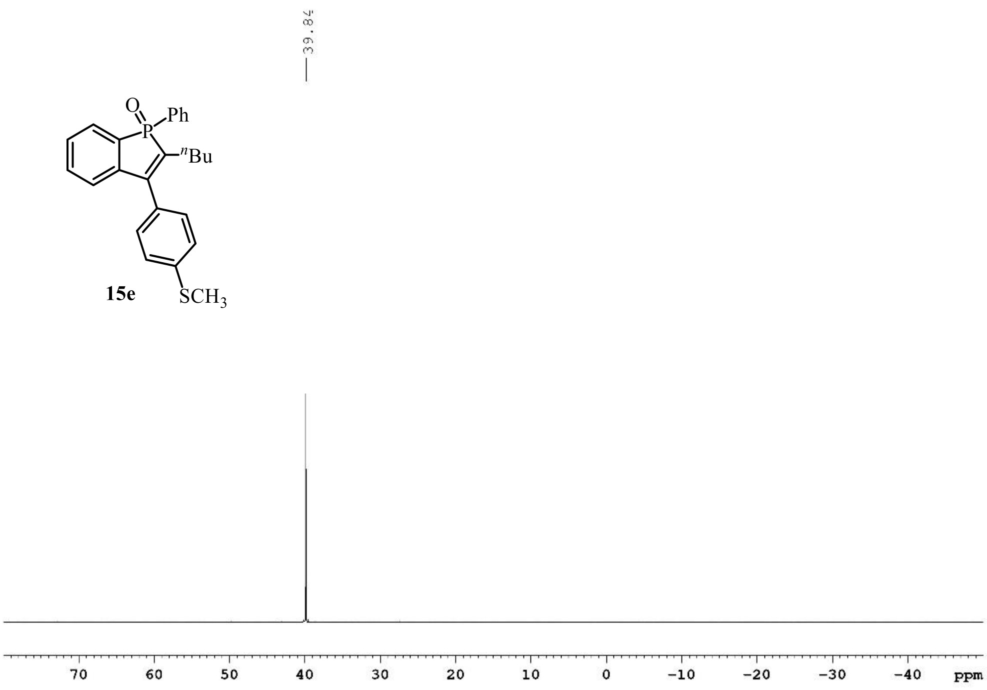

^{1}H NMR (400 MHz, $CDCl_3$) of 15f

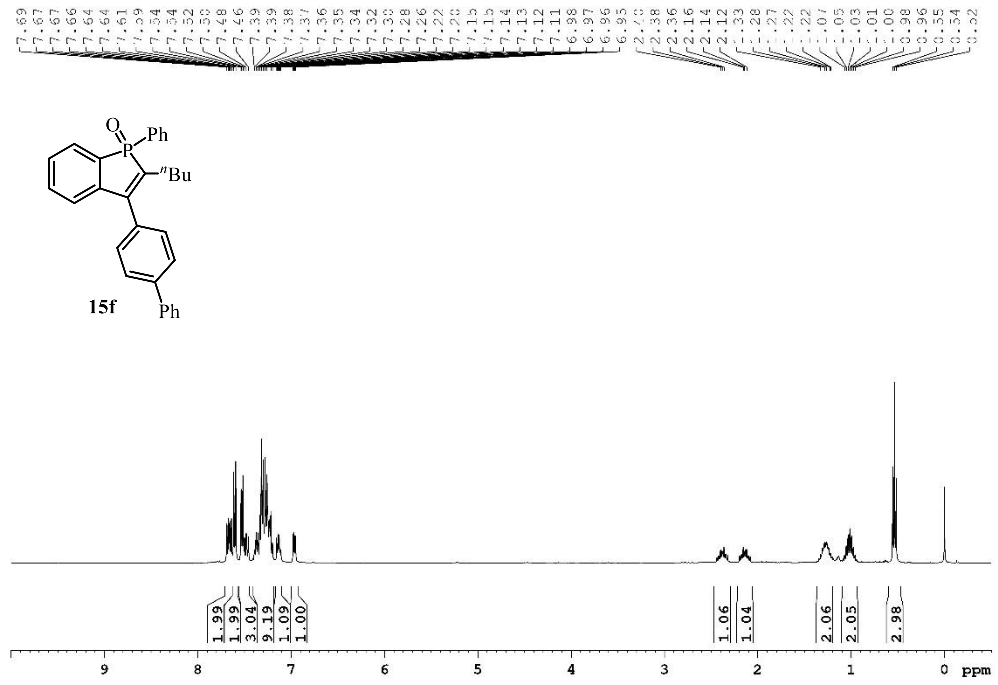

^{13}C NMR (101 MHz, $CDCl_3$) of 15f

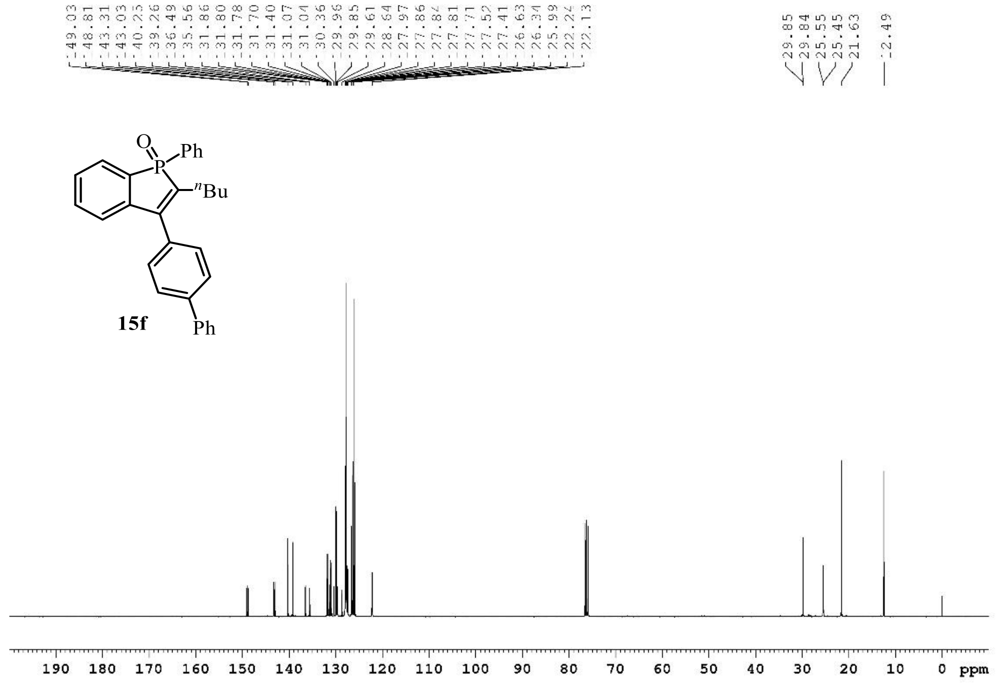

^{31}P NMR (162 MHz, $CDCl_3$) of 15f

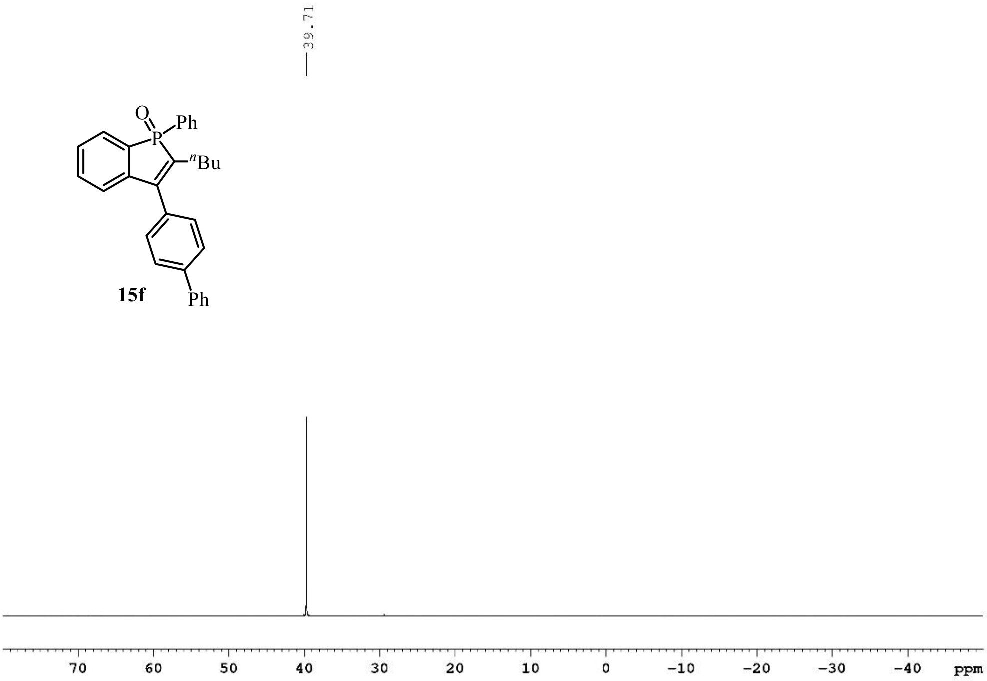

^{1}H NMR (400 MHz, $CDCl_3$) of 15g

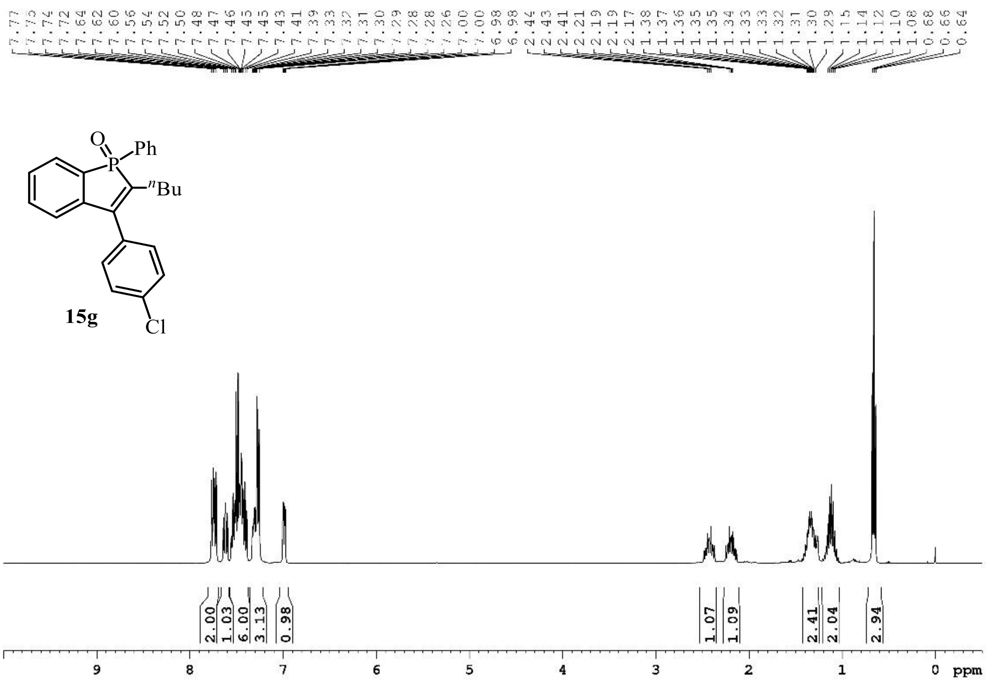

^{13}C NMR (101 MHz, CDCl$_3$) of 15g

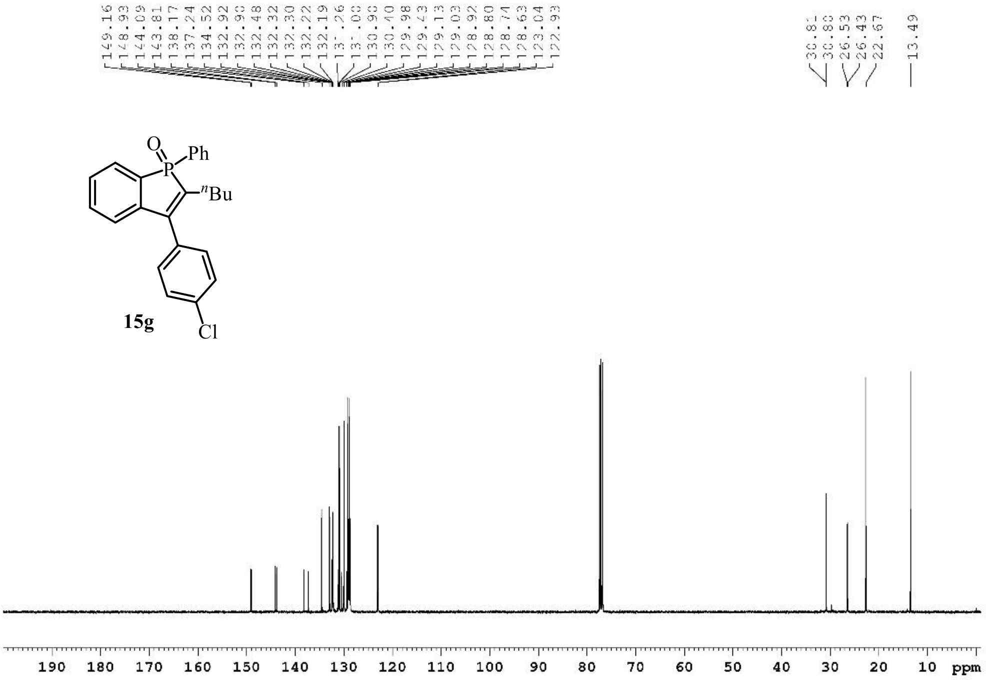

^{31}P NMR (162 MHz, CDCl$_3$) of 15g

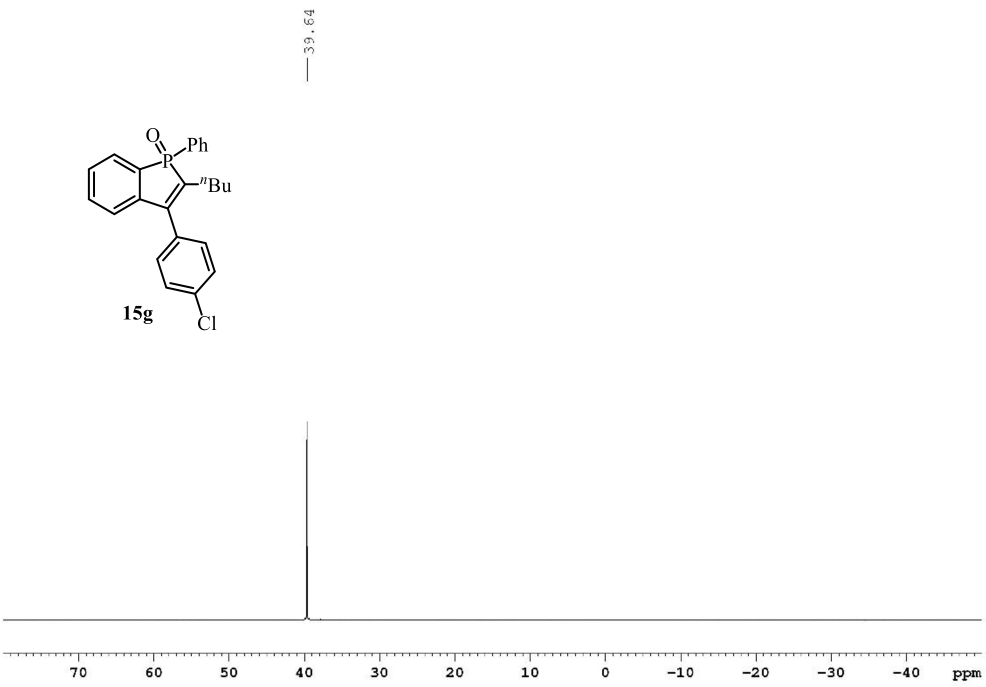

^{1}H NMR (400 MHz, $CDCl_3$) of 15h

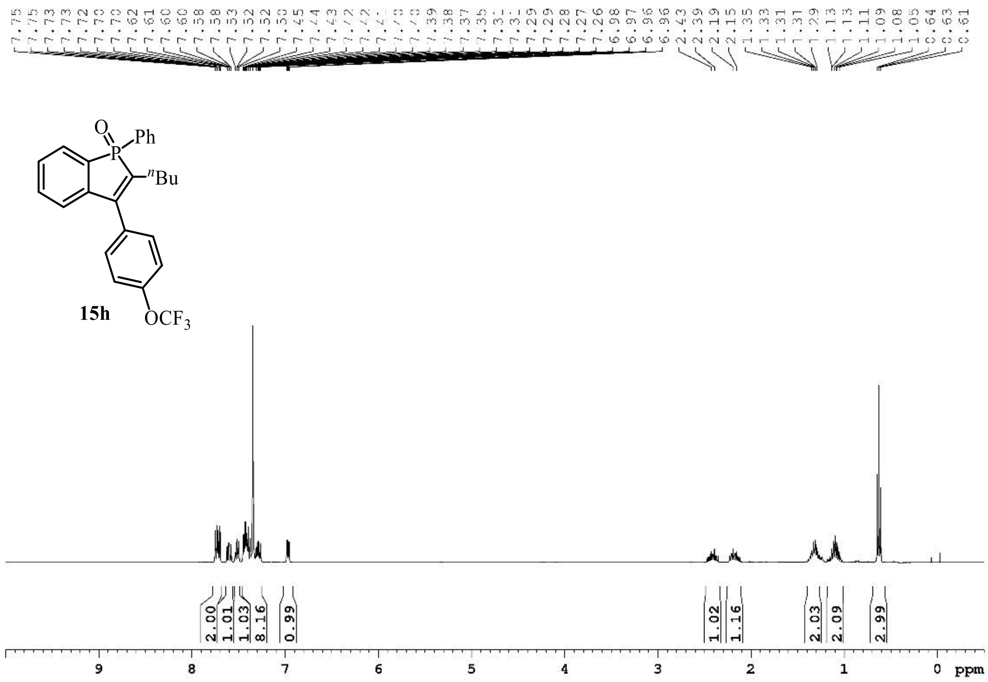

^{13}C NMR (101 MHz, $CDCl_3$) of 15h

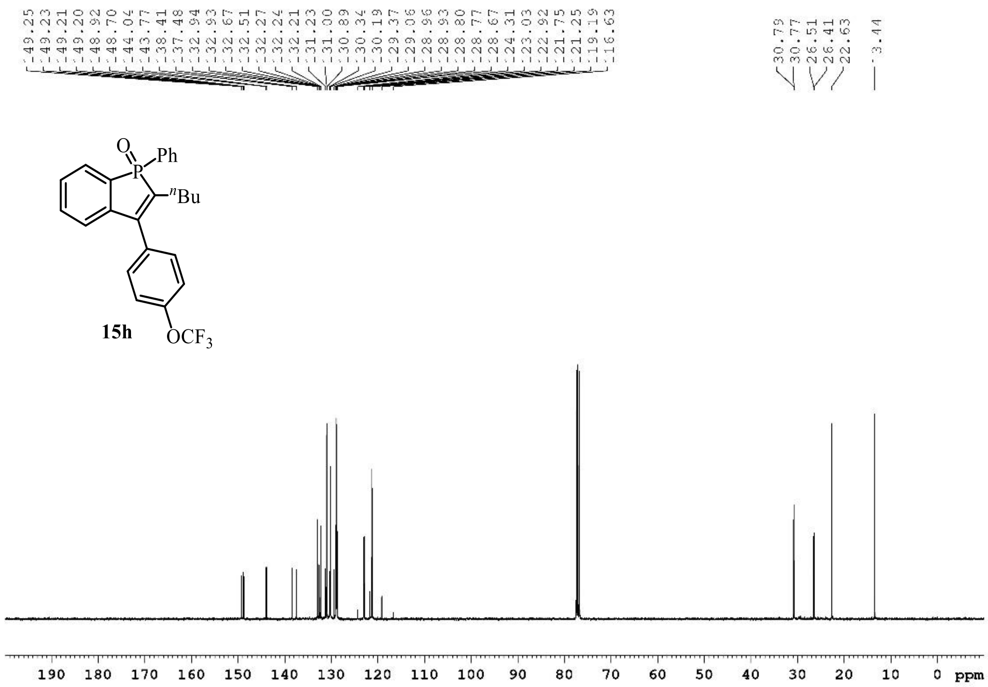

^{31}P NMR (162 MHz, $CDCl_3$) of 15h

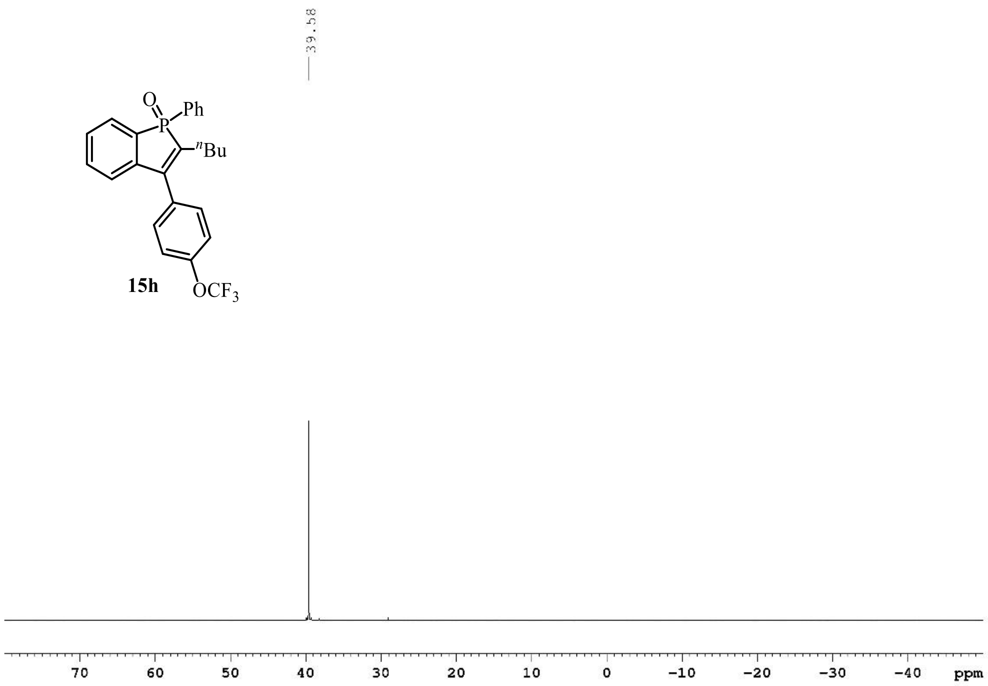

^{1}H NMR (400 MHz, $CDCl_3$) of 15i

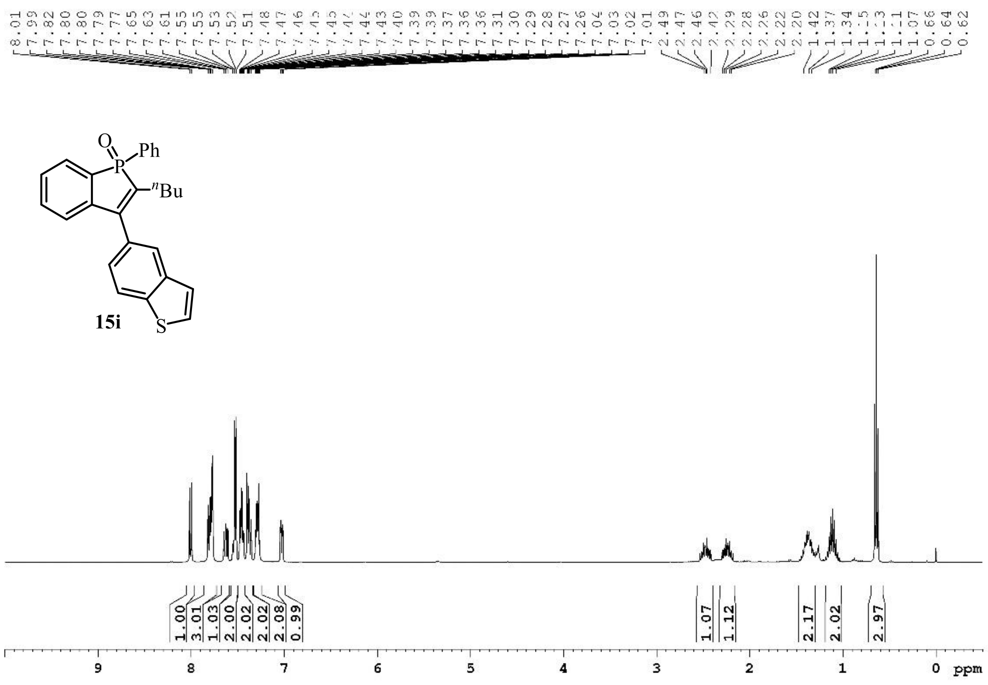

^{13}C NMR (101 MHz, CDCl$_3$) of 15i

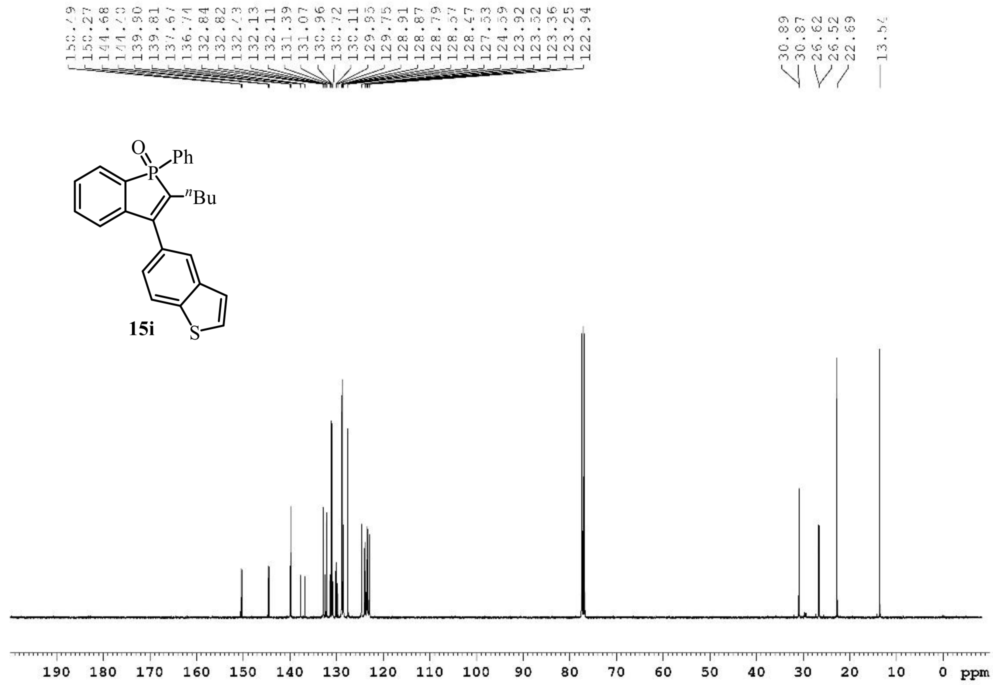

^{31}P NMR (162 MHz, CDCl$_3$) of 15i

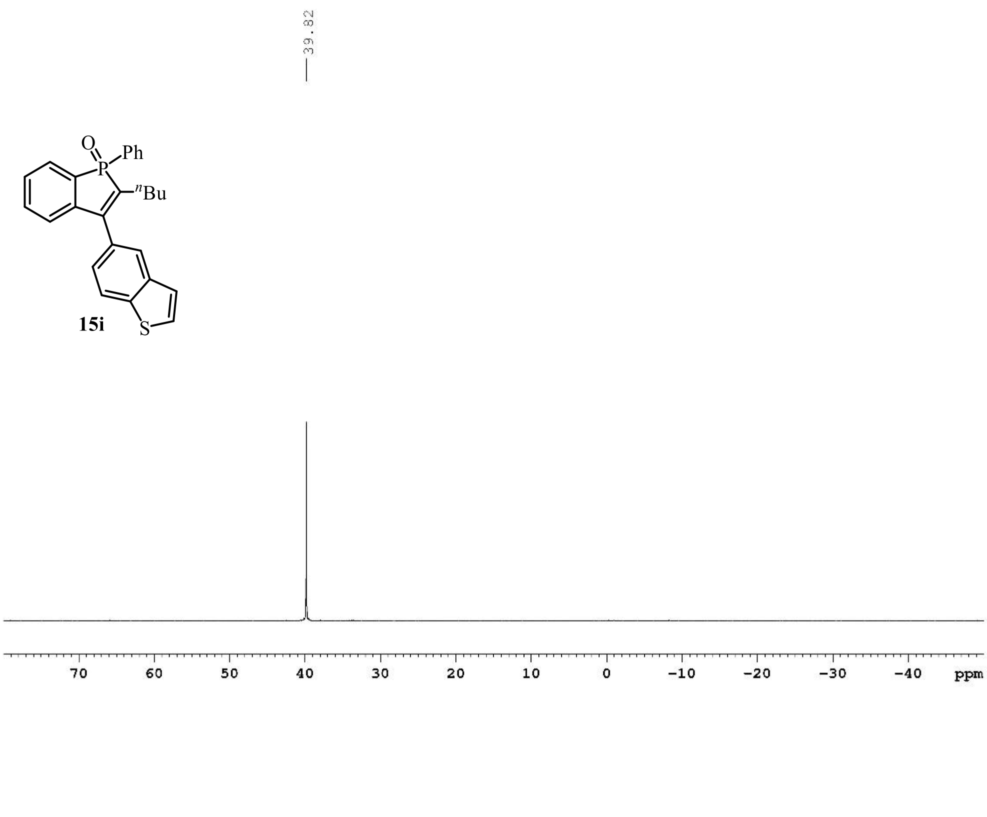

^{1}H NMR (400 MHz, CDCl$_3$) of 15j

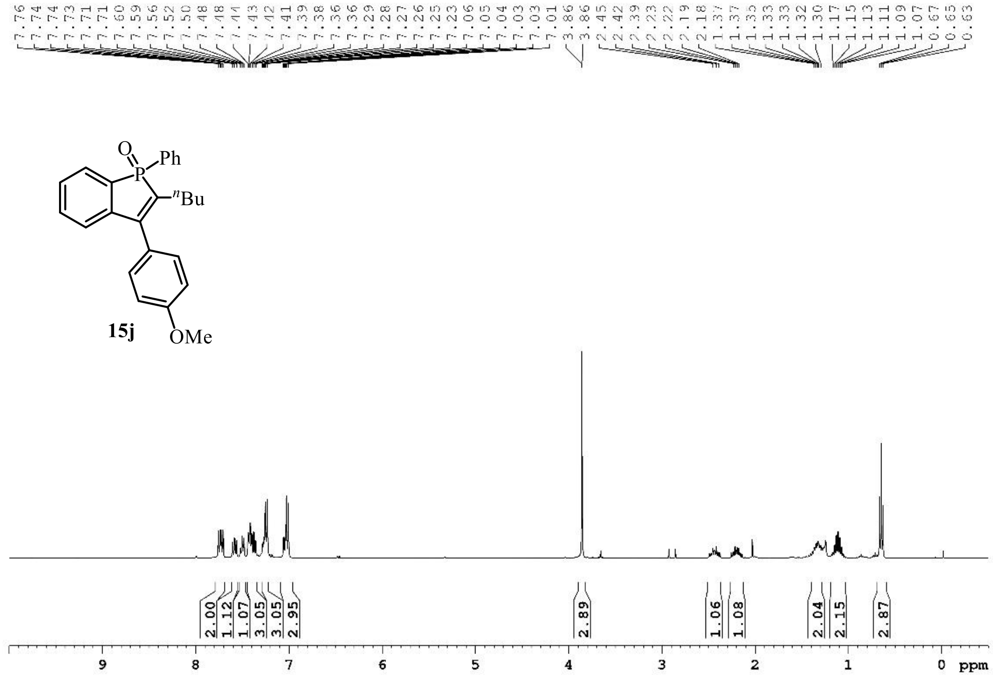

^{13}C NMR (101 MHz, CDCl$_3$) of 15j

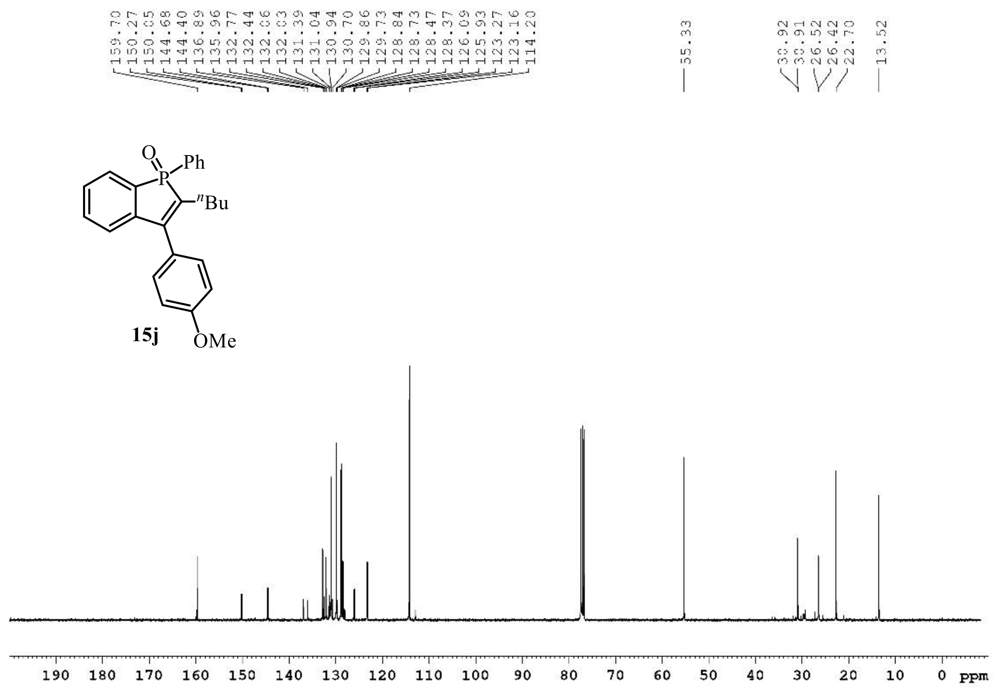

^{31}P NMR (162 MHz, CDCl$_3$) of 15j

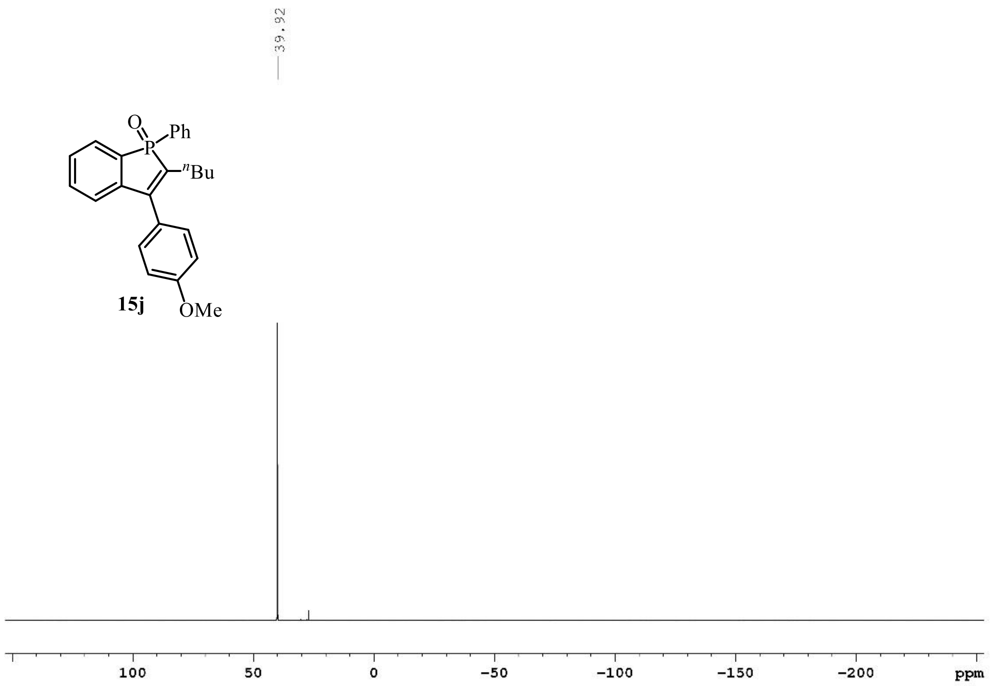

^{1}H NMR (400 MHz, CDCl$_3$) of 16

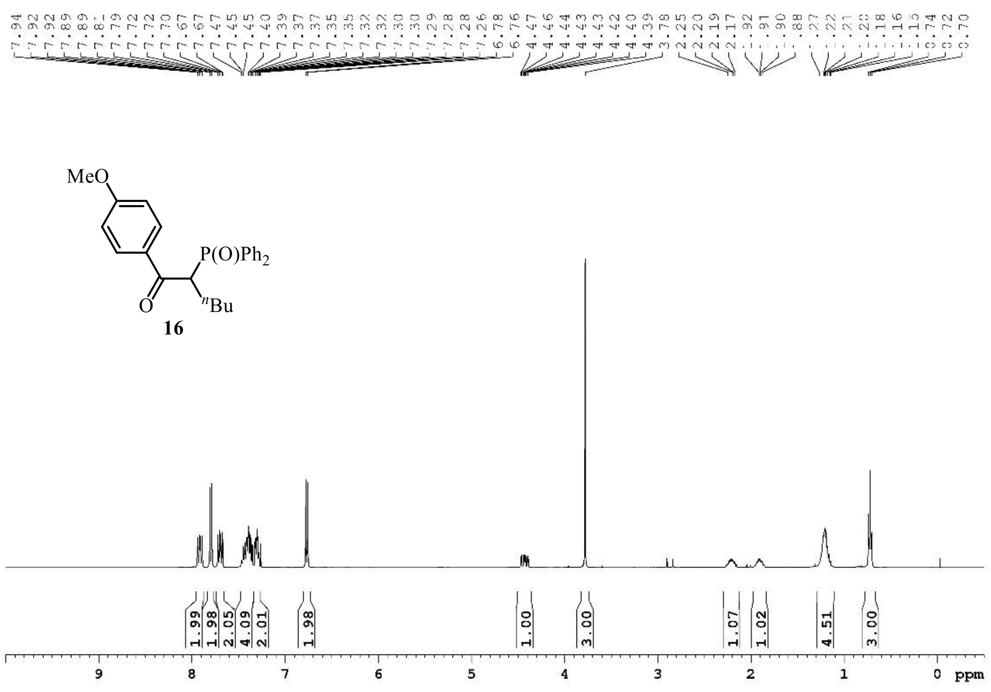

^{13}C NMR (101 MHz, CDCl$_3$) of 16

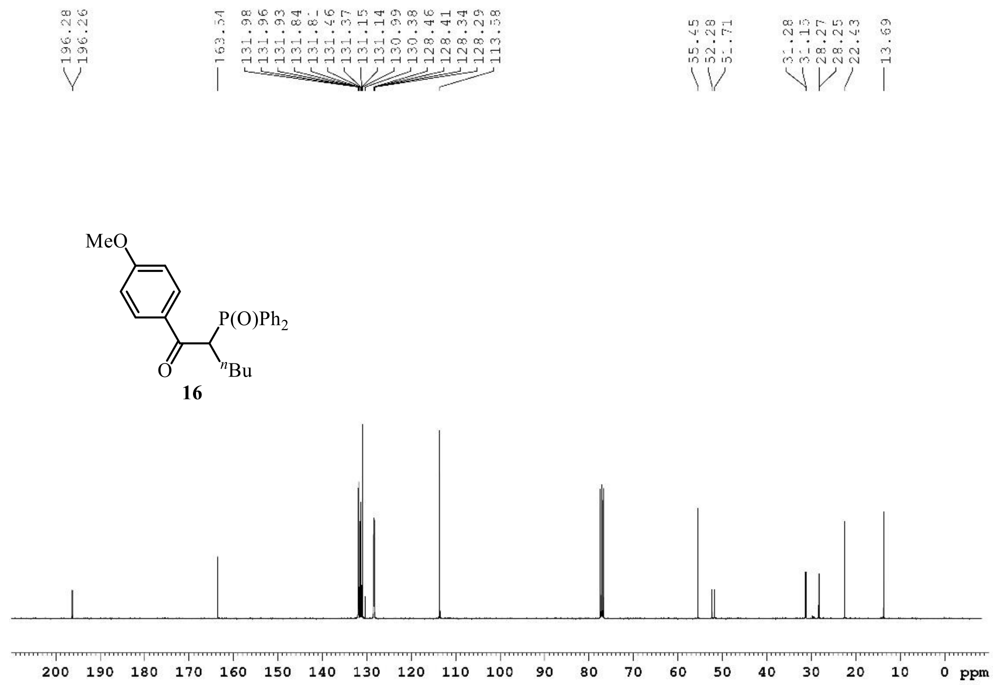

^{31}P NMR (162 MHz, CDCl$_3$) of 16

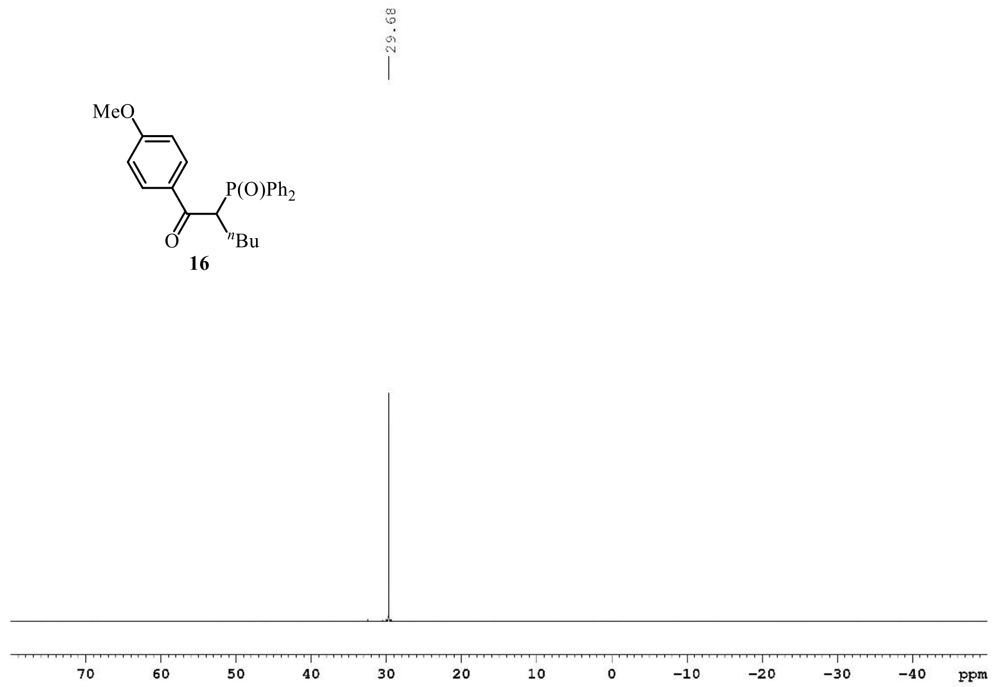

14. References

(1) Zhang, D. et al. Preparation of O-Protected Cyanohydrins by Aerobic Oxidation of α-Substituted Malononitriles in the Presence of Diarylphosphine Oxides. *Org. Lett.* **21**, 2597-2601 (2019).

(2) Liu, X.-T. et al. Ni-Catalyzed Asymmetric Hydrophosphination of Unactivated Alkynes. *J. Am. Chem. Soc.* **143**, 11309-11316 (2021).

(3) Gregori, B. J. et al. Stereoselective Chromium-Catalyzed Semi-Hydrogenation of Alkynes. *ChemCatChem* **12**, 5359-5363 (2020).

(4) Liu, J., Jia, X. & Huang, L. Sulfilimines as Transformable and Retainable Directing Groups in Rhodium-Catalyzed *ortho*-C-H Bond Functionalization. *Org. Lett.* **24**, 6772-6776 (2022).

(5) Wu, J. & Yoshikai, N. Cobalt-Catalyzed Alkenylzincation of Unfunctionalized Alkynes. *Angew. Chem. Int. Ed.* **55**, 336-340 (2016).

(6) Zhang, Z., Luo, Y., Du, H., Xu, J. & Li, P. Synthesis of α-Heterosubstituted Ketones Through Sulfur Mediated Difunctionalization of Internal Alkynes. *Chem. Sci.* **10**, 5156-5161 (2019).

(7) Guo, W.-H., Zhao, H.-Y., Luo, Z.-J., Zhang, S. & Zhang, X. Fluoroalkylation-Borylation of Alkynes: An Efficient Method To Obtain (*Z*)-Tri- and Tetrasubstituted Fluoroalkylated Alkenylboronates. *ACS Catal.* **9**, 38-43 (2019).

(8) Unoh, Y., Hirano, K. & Miura, M. Metal-Free Electrophilic Phosphination/Cyclization of Alkynes. *J. Am. Chem. Soc.* **139**, 6106-6109 (2017).

(9) Chu, X.-Q. et al. Defluorophosphorylation of fluoroalkyl peroxides for the synthesis of highly substituted furans. *Green Chem.* **25**, 2000–2010 (2023).

(10) Frisch, M. J. et al. Gaussian 09, Revision B.01; Gaussian, Inc., Wallingford, CT (2010).

(11) Zhao, Y. & Truhlar, D. G. The M06 Suite of Density Functionals for Main Group Thermochemistry, Thermochemical Kinetics, Noncovalent Interactions, Excited States, and Transition Elements: Two New Functionals and Systematic Testing of Four M06-Class Functionals and 12 Other Functionals. *Theor. Chem. Acc.* **120**, 215-241 (2008).

(12) Zhao, Y. & Truhlar, D. G. Exploring the Limit of Accuracy of The Global Hybrid Meta Density Functional for Main-group Thermochemistry, Kinetics, and Noncovalent Interactions. *J. Chem. Theory. Comput.* **4**, 1849-1868 (2008).

(13) Hehre, W. J., Ditchfield, R. & Pople, J. A. Self-consistent Molecular Orbital Methods. XLL. Further Extensions of Gaussian-type Basis Sets for Use in Molecular Orbital Studies of Organic Molecular. *J. Chem. Phys.* **56**, 2257-2261 (1972).

(14) Sang-Aroon, W. & Ruangpornvisuti, V. Determination of Aqueous Acid-dissociation Constants of Aspartic Acid Using PCM/DFT Method. *Int. J. Quantum Chem.* **108**, 1181-1188 (2007).

(15) Hay, P. J. & Wadt, W. R. Ab Initio Effective Core Potentials for Molecular Calculations. Potentials for The Transition Metal Atoms Sc to Hg. *J. Chem. Phys.* **82**, 270-283 (1985).

(16) Wadt, W. R. & Hay, P. J. Ab Initio Effective Core Potentials for Molecular Calculations. Potentials for Main Group Elements Na to Bi. *J. Chem. Phys.* **82**, 284-298 (1985).

(17) Barone, V. & Cossi, M. Quantum Calculation of Molecular Energies and Energy Gradients in Solution by A Conductor Solvent Model. *J. Phys. Chem. A.* **102**, 1995-2001 (1998).

(18) Quint, V. et al. Metal-Free, Visible Light-Photocatalyzed Synthesis of Benzo[*b*]phosphole Oxides: Synthetic and Mechanistic Investigations. *J. Am. Chem. Soc.* **138**, 7436-7441 (2016).
